# Placebo response in pharmacological and dietary supplement trials of autism spectrum disorder (ASD): systematic review and meta-regression analysis

**DOI:** 10.1186/s13229-020-00372-z

**Published:** 2020-08-26

**Authors:** Spyridon Siafis, Oğulcan Çıray, Johannes Schneider-Thoma, Irene Bighelli, Marc Krause, Alessandro Rodolico, Anna Ceraso, Giacomo Deste, Maximilian Huhn, David Fraguas, Dimitris Mavridis, Tony Charman, Declan G. Murphy, Mara Parellada, Celso Arango, Stefan Leucht

**Affiliations:** 1 Department of Psychiatry and Psychotherapy, School of Medicine, Technical University of Munich, Ismaningerstr. 22, 81675 Munich, Germany; 2 Department of Child and Adolescent Psychiatry, School of Medicine, Balçova Dokuz Eylul University, İzmir, Turkey; 3 Department of Experimental and Clinical Medicine, Psychiatric Clinic University Hospital ‘Gaspare Rodolico’, University of Catania, Catania, Italy; 4 Department of Psychiatry, Spedali Civili Hospital, Brescia, Italy; 5 Department of Psychiatry, Psychosomatic Medicine and Psychotherapy Social Foundation Bamberg, Teaching Hospital of the University of Erlangen, Erlangen, Germany; 6 Department of Child and Adolescent Psychiatry, Hospital General Universitario Gregorio Marañón, Institute of Psychiatry and Mental Health, IiSGM, CIBERSAM, Madrid, Spain; 7 School of Medicine, Universidad Complutense, Madrid, Spain; 8 Department of Primary Education, University of Ioannina, Ioannina, Greece; 9 Faculté de Médecine, Université Paris Descartes, Paris, France; 10 Department of Psychology, Institute of Psychiatry, Psychology & Neuroscience, King’s College London, London, UK; 11 Department of Forensic and Neurodevelopmental Sciences, Institute of Psychiatry, Psychology & Neuroscience, King’s College London, London, UK

**Keywords:** Autism spectrum disorder, Placebo, Trials

## Abstract

**Background:**

Placebo response in autism spectrum disorder (ASD) might dilute drug-placebo differences and hinder drug development. Therefore, this meta-analysis investigated placebo response in core symptoms.

**Methods:**

We searched ClinicalTrials.gov, CENTRAL, EMBASE, MEDLINE, PsycINFO, WHO-ICTRP (up to July 8, 2018), and PubMed (up to July 4, 2019) for randomized pharmacological and dietary supplement placebo-controlled trials (RCTs) with a minimum of seven days of treatment. Single-group meta-analyses were conducted using a random-effects model. Standardized mean changes (SMC) of core symptoms in placebo arms were the primary outcomes and placebo positive response rates were a secondary outcome. Predictors of placebo response were investigated with meta-regression analyses. The protocol was registered with PROSPERO ID CRD42019125317.

**Results:**

Eighty-six RCTs with 2360 participants on placebo were included in our analysis (87% in children/adolescents). The majority of trials were small, single-center with a duration of 8–12 weeks and published after 2009. Placebo response in social-communication difficulties was SMC = − 0.32, 95% CI [− 0.39, − 0.25], in repetitive behaviors − 0.23[− 0.32, − 0.15] and in scales measuring overall core symptoms − 0.36 [− 0.46, − 0.26]. Overall, 19%, 95% CI [16–22%] of participants were at least much improved with placebo. Caregiver (vs. clinician) ratings, lower risk of bias, flexible-dosing, larger sample sizes and number of sites, less recent publication year, baseline levels of irritability, and the use of a threshold of core symptoms at inclusion were associated with larger placebo response in at least a core symptom domain.

**Limitations:**

About 40% of the trials had an apparent focus on core symptoms. Investigation of the differential impact of predictors on placebo and drug response was impeded by the use of diverse experimental interventions with essentially different mechanisms of action. An individual-participant-data meta-analysis could allow for a more fine-grained analysis and provide more informative answers.

**Conclusions:**

Placebo response in ASD was substantial and predicted by design- and participant-related factors, which could inform the design of future trials in order to improve the detection of efficacy in core symptoms. Potential solutions could be the minimization and careful selection of study sites as well as rigorous participant enrollment and the use of measurements of change not solely dependent on caregivers.

## Background

Autism spectrum disorder (ASD) is a group of heterogeneous neurodevelopmental conditions, characterized by social-communication difficulties as well as repetitive-restricted behaviors and sensory abnormalities [1]. The prevalence is about 1–2% [2, 3], and lifetime costs are substantial (at US $1.4–2.44 million per individual) [4]. Behavioral interventions are the cornerstone of treatment and there is still no approved medication for the core symptoms [5]. Despite that, about half of the individuals with ASD, who might be more susceptible to side effects than neurotypical populations [5], receive psychotropic drugs [6]. Currently approved medications target associated symptoms, e.g., aripiprazole and risperidone for irritability [5]. Therefore, there is an unmet need to develop effective and safe treatments that target causal pathophysiological pathways, improve core symptoms and quality of life.

In spite of the recent advances in “translational” research, late-stage clinical trials for neurodevelopmental disorders have failed [7]. The low success rate could be explained by several factors, such as poor translational validity of preclinical models, true lack of drug efficacy, and suboptimal trial design [8]. One concern is also that placebo effects might dilute effect sizes. However, the magnitude and predictors of placebo response in core symptoms of ASD are still unknown; only investigated in post-hoc analyses of single trials [9, 10] and meta-analyses using aggregated outcome measures, potentially confounded by associated symptoms [11, 12]. In summary, placebo response may play an important role in the failure of clinical trials and the subsequent lack of approved medications for core symptoms. In order to improve the design and sensitivity of future trials, we meta-analyzed placebo response of core symptoms in pharmacological and dietary supplement ASD trials.

## Methods

This systematic review and meta-regression analysis was conducted according to PRISMA [13] (Additional file 3: eAppendix-1) with PROSPERO registration ID CRD42019125317 (Additional file 3: eAppendix-2).

### Participants and interventions

#### Participants

Participants with a diagnosis of ASD using standardized diagnostic criteria (e.g., DSM-III, ICD-10, or more recent versions) and/or validated diagnostic tools (e.g., ADI-R) [5]. There were no restrictions in terms of age, sex, ethnicity, setting, severity, or the presence of co-occurring conditions.

#### Interventions

Any pharmacological treatment or dietary supplement was eligible, when compared with placebo. We excluded psychological/behavioral and combination interventions (since placebo response might be confounded by the active component of the combination) as well as other interventions (e.g., elimination diets, milk formulations, or homeopathy). The minimum duration of treatment was 7 days, since we aimed to investigate a broad range of data but to exclude trials with a clearly very short duration, e.g., single-dose interventions. There was no restriction in terms of route of administration and dosing-schedule.

#### Type of studies

Blinded and unblinded randomized placebo-controlled trials (RCTs) were eligible. In case of cross-over studies, we used only data from the first phase of the crossover to avoid carryover effects [14]. We excluded studies with placebo-controlled discontinuation or cluster randomization [15], published before 1980 or smaller than ten participants [16]. Risk of bias of included studies was evaluated by at least two independent reviewers (SS, OC, AR) using the Cochrane Collaboration risk-of-bias tool [17]. Disagreements were resolved by discussion, and if needed, a third author was involved (SL, JST). Studies with a high risk of bias in sequence generation or allocation concealment were excluded (e.g., allocation by alternation or by an unblinded investigator). Studies were further classified as having an overall low, moderate, or high risk of bias [18].

### Search strategy and selection criteria

We searched (July 8, 2018) ClinicalTrials.gov, CENTRAL, EMBASE, MEDLINE, PsycINFO, PubMed (update on July 4, 2019), and WHO ICTRP. There was no date/time, language, document type, and publication status limitations (Additional file 3: eAppendix-3). Reference lists of included studies and previous reviews [5, 11, 12, 19–27] were also inspected.

### Outcome measures and data extraction

We investigated placebo response in core symptoms. The following primary outcomes, as measured by published scales, were analyzed: (1) social-communication difficulties (e.g., ABC-L/SW [28] or VABS-Socialization [29]), (2) repetitive-restricted behaviors (e.g., ABC-S [28] or CYBOC-PDD [30]), and (3) overall measures of core symptoms (e.g., SRS [31] or CARS [32]). There is no agreement on the optimal outcome measures to use in clinical trials of ASD and so preference was given to the aforementioned most frequently used scales (Additional file 3: eAppendix-5.3) [5, 33–36]. A higher score indicated more difficulties and when necessary, scores were minus-transformed. In the primary analysis, we pooled all studies by preferring ratings by clinicians (observations or interviews) to caregivers/teachers. Separate analyses by type of raters and positive response to treatment defined as at least much improvement in CGI-I, preferably anchored to global autism or core symptoms (when more than one CGI-I evaluations were reported), were analyzed as secondary outcomes. When the number of participants with a positive response was not reported, it was imputed from mean and standard deviation (SD) of CGI-I using a validated method (Additional file 3: eAppendix-2.2) [37, 38].

At least two independent reviewers/contributors selected relevant records and extracted data from eligible studies in an Access database (SS, OC, IB, AR, AC, GD, MK, YZ, and TF). Intention-to-treat data were preferred when available, and for a positive response to treatment, if the original authors presented only the results of completer population, we assumed that participants lost to follow-up did not have a positive response to treatment. Missing SDs were calculated according to the following hierarchy from available statistics (e.g., SE, *p* values, *t* tests) [39], median/range [40], pooling subscales (e.g., SRS subscales, assuming a correlation of 0.5) [41], or using a validated imputation method [39, 42]. Corresponding authors were contacted by e-mail for additional data, with a reminder e-mail in case of no response (complete list in Additional file 3: eAppendix-4).

### Statistical analysis

#### Synthesis of the results

Single-group meta-analyses of placebo arms were conducted using a random effects model [43]. The effect size for continuous outcomes (core symptoms) was the standardized mean change (SMC) with raw score standardization using the baseline SD of the placebo arm [44, 45]. When baseline SDs were not reported, change or follow-up SDs were used. In the primary analysis, a common pre-post correlation of 0.5 [41] was used for the calculation of variance of SMC [44]. Positive response rates were logit transformed, and back-transformed for presentation [46]. Heterogeneity was evaluated by visual inspection of forest plots and with the *χ*^2^ (*p* value < 0.1) and *I*^2^ statistics (considerable heterogeneity when > 50%); *χ*^2^ might detect small amounts of clinically unimportant heterogeneity; therefore, we based our evaluation on *I*^2^ [17].

#### Sensitivity analyses and publication bias

Predefined sensitivity analyses of the primary outcomes were conducted using a fixed-effects model or by exclusion of studies with genetic syndrome as inclusion criteria, using only diagnostic tools, single-blind, shorter than 4 weeks, presenting only completers data, with at least moderate overall risk of bias, with estimated SD (imputed, from medians/range, or pooled subscales). Post-hoc, we excluded studies without baseline SDs and we used the correlations of 0.25/0.75 for the calculation of variance of SMC [41]. Regarding responder rates, we post-hoc excluded studies with imputed responder rates [38]. We explored small study effects as proxy for publication bias with contour-enhanced funnel plots, Egger’s test [47], and trim-and-fill [48].

#### Meta-regression analyses

The dependent variable was SMC and the independent variable was selected from a list of covariates from the literature [9, 11, 12, 49–51]. First, we conducted univariable and then multivariable meta-regressions similar to our previous analyses in schizophrenia [51]: we used the factors that were significant in the univariable analysis and then a formal backward stepwise algorithm with a removal criterion of *p* = 0.15. Meta-regressions were not performed for categorical covariates with less than five data points per level. Spearman’s ρ were calculated post-hoc between SMCs of placebo and experimental intervention as well as between covariates.

##### Intervention-related factors

Intervention-related factors were route of administration (oral versus others) [52], type of experimental intervention (pharmacological versus dietary supplement), and dose-schedule (fixed versus flexible).

##### Study-related factors

Study-related factors were duration of treatment (weeks), publication year, washout from psychotropic medications (coded post-hoc as the presence of washout or not, because definitions varied), placebo lead-in with exclusion of those showing a positive response, type of rater (clinicians versus caregivers), total sample size, number of sites, %academic sites, number of arms and medications, %participants on placebo, sponsorship (industry-funded/patent application versus industry-independent), country of origin (US versus not only US), and risk of bias domains.

##### Participant-related factors

Participant-related factors were the presence of any associated conditions by inclusion criteria (i.e., irritability, ADHD, and other conditions apart from intellectual disability or genetic syndrome), mean age and age group (children/adolescent versus adults/mixed, post-hoc), %participants with intellectual disability (at least mild or IQ < 70), %female (post-hoc), ethnicity (%Caucasian/Hispanic, post-hoc), and baseline BMI (post-hoc) [9]. Due to inconsistent reporting of baseline severity [11, 12], we used CGI-Severity (ranging 1–7) as a measure of global severity and ABC-Irritability (ranging 0–45) as a measure of serious behavioral problems [53]. Baseline severity in core symptoms could not be investigated as a potential predictor due to the large diversity of scales and standardization methods (such as using the lower and upper limits of the measurement scale [54]) could not be utilized (trials reported raw and standard scores such as of VABS or T-scores of SRS). We also examined the use of a threshold of core symptom severity for inclusion (not only for the confirmation of diagnosis).

Analyses were conducted using metafor (v2.1-0) [45] and meta (v4.9-9) [55] in R (v3.6.2) [56]. Statistical threshold was set at two-sided alpha 5%. Due to the limited statistical power and exploratory (observational) nature of meta-regression analyses, alpha was not adjusted for multiple testing. Correction for multiple testing is not generally recommended by the Cochrane Handbook [39].

## Results

### Description of included studies

**Fig. 1:**
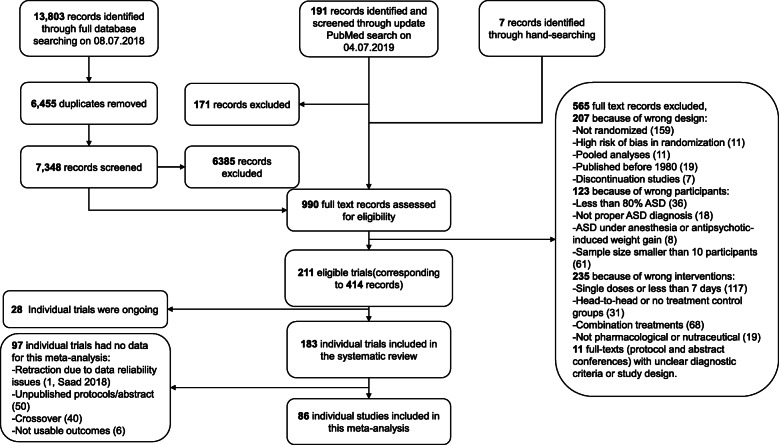
PRISMA flow chart of study selection. The list of included, ongoing and excluded records is displayed in Additional file 3: eAppendix-4.

The PRISMA flow diagram is presented in Fig. 1. In this analysis, 86 (*k*) studies were included, 71% comparing pharmacological treatments and 29% dietary supplements with placebo (eAppendix-5.1 and Table-S). Of the 86 studies, 75 were conducted in children/adolescents, eight in adults, and three included both age groups. The overall sample size (*n*) was 5365, 44% on placebo. The majority of studies were parallel (85%), single-center (60%, indicated in *k* = 78), and double-blind (only one single-blind [57] and none was open) with two arms (88%) and small sample sizes (median 45, interquartile-range [30–91]). About half of the studies (48%) had a duration of 12 weeks or more and three less than 4 weeks [57–59] (median 10 weeks [8–12]) as well as half used a fixed dose schedule (51%, *k* = 84) and had a washout from psychotropic drugs (55%, *k* = 75), yet definitions and duration varied. Placebo lead-in with exclusion of those with a positive response was used in five studies.

All of the studies used standardized diagnostic criteria, except five that used only diagnostic tools [60–64]. Associated conditions were the focus of and they were required for inclusion in 29 studies (irritability in 52% of them), and a genetic syndrome in one (neurofibromatosis-type-1) [60]. Core symptoms was the primary focus in 34 trials, while the focus was unclear in 23 studies. Nine studies included participants using a threshold of core symptom severity, using ABC-L/SW, CARS, RLRS, SRS, and YBOCS-versions (in five out of eight). Participants on placebo had a median age of 8.63 years ([6.55–10.16], *k* = 82), 17.16% were female ([0–20.9%], *k* = 80), and 54.2% had comorbid intellectual disability ([0–75%], *k* = 30). The median of baseline CGI-Severity was 4.73 ([4.39–5.00], *k* = 38) and ABC-Irritability was 17.18 ([13.71–22.70], *k* = 36).

Overall, 40% of the studies had an overall low risk of bias, 52% moderate and 8% high. Description of the methods was adequately reported in more than half of the studies for sequence generation (63%), allocation concealment (54%), and blinding (72%). Missing outcomes were adequately addressed in 60%, with a median overall dropout rate from placebo of 12.93% ([6.25–22.6%] and *k* = 70 trials out of 86 reported attrition rates). Of the studies, 23% had a high risk of selective reporting, and 13% high risk in other biases, mainly due to imbalances between groups (Additional file 3: eAppendix-5.2). Finally, 38% of the studies were industry-sponsored (including five in which investigators applied for a patent on the experimental intervention), and sponsorship was unclear in three studies.

### Primary outcomes

#### Social-communication difficulties

##### Primary analysis

**Fig. 2:**
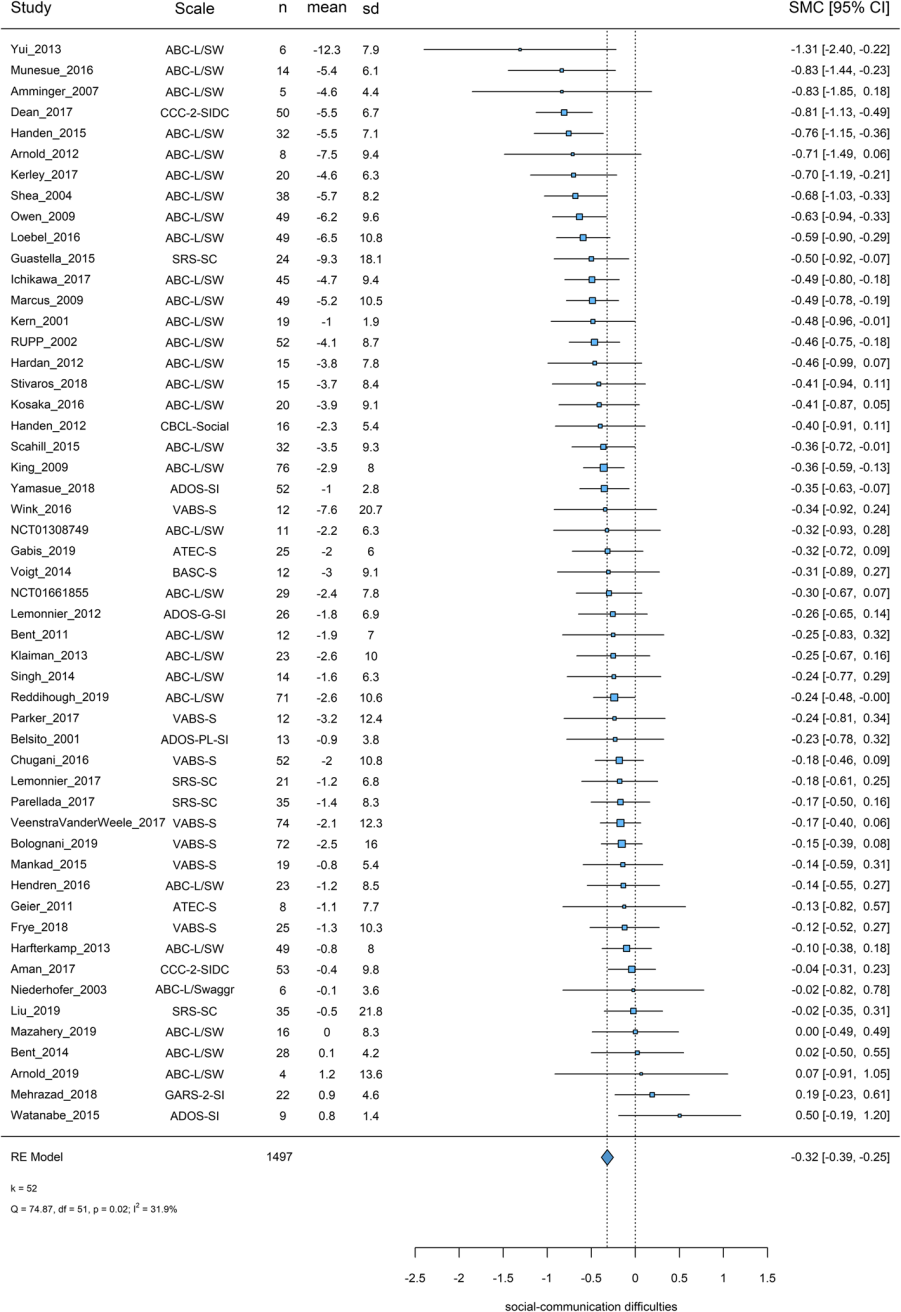
Placebo response in scales measuring social-communication difficulties. Squares and bars represent standardized mean changes (SMC) and 95% confidence intervals for each study. The size of the square is proportional to the weight of the study in the meta-analysis. The diamond represent the pooled SMC. Heterogeneity is quantified with a χ^2^ test (*Q*) and *I*^2^. *In Chugani 2016, standard errors might have been reported as SDs. Therefore, we calculated SDs from the reported values (no reply from the corresponding author). It should be noted that in Niederhofer 2003, an aggregated score of ABC-L/SW rated by both caregivers and teachers were reported, in Amminger 2007, ABC-L/SW was rated by clinicians of the day care center. Scale: the scale used (clinician rated scales based on observation or interviews were preferred in the primary analysis); *n*: the number of participants on placebo; mean: mean change from baseline to endpoint (negative values for improvement); sd: the standard deviation used for the standardization (baseline standard deviations were preferred); SMC: standardized mean changes, 95% CI: 95% confidence intervals, *k* = total number of studies included in the analysis

In the primary analysis, 52 studies with 1497 participants on placebo were included. Most of the scales were filled by caregivers (77%). ABC-L/SW was the most used scale (56%) followed by VABS-Socialization (13%). Pooled placebo response was SMC = − 0.32 [95%CI − 0.39, − 0.25], with moderate levels of heterogeneity (*I*^2^ = 31.88%, *χ*^2^ = 74.87, *p* = 0.02) (Fig. 2).

##### Sensitivity analysis and publication bias

The results did not change materially in sensitivity analyses (Additional file 3: eAppendix-6.1). There was no indication of small-study effects from a visual inspection of the funnel plot and Egger’s test or publication bias (*z* = − 0.38, *p* = 0.70) (Additional file 3: eAppendix-6.2). Also, fixed and random effects summaries were identical, an indication that smaller and larger studies give similar results.

##### Meta-regressions

The results of the univariable and multivariable meta-regression analyses are presented in Table 1, Figure-S, and eAppendix-6.3. Placebo response in social-communication difficulties decreased over years (by 0.016 [0.003, 0.030] SMC units per publication year). Larger placebo response was associated with caregiver ratings (−0.164 [−0.315, −0.012]), low risk of bias in other bias (− 0.160 [− 0.299, − 0.021]), and higher baseline ABC-Irritability (− 0.017 [− 0.028, − 0.006] per point). In the multivariable meta-regression, using the backward selection procedure, other bias, baseline ABC-Irritability, and the type of rater remained as covariates in the model, but the latter two were not significant due to their interaction with the other covariates. In a model without ABC-Irritability (available in 31 studies), publication year, other bias, and type of rater remained, the latter was not significant.

**Table 1:** Univariable meta-regression analyses

Covariate		Social-communication difficulties				Repetitive behaviors and restricted interests				Overall core symptoms			
		*k*, *n*	β [95% CI]	*p*	*R*^2^ (%)	*k*, *n*	β [95% CI]	*p*	*R*^2^ (%)	*k*, *n*	β [95% CI]	*p*	*R*^2^ (%)
Intervention	Route (ref. not oral)	52, 1497	0 [− 0.205, 0.206]	0.998	0	52, 1492	− 0.166 [− 0.391, 0.059]	0.148	8.13	45, 1063	− 0.042 [− 0.301, 0.217]	0.749	0
	Experimental intervention (ref. dietary supplement)	52, 1497	− 0.082 [− 0.229, 0.064]	0.268	2.61	52, 1492	− 0.081 − 0.262, 0.1]	0.382	0	45, 1063	− 0.023 [− 0.228, 0.181]	0.822	0
	Dose schedule (ref. fixed)**	51, 1491	− 0.095 [− 0.229, 0.038]	0.163	2.57	**51, 1486**	− **0.195 [**− **0.351,** − **0.038]**	**0.015**	27.03	44, 1057	− 0.044 [− 0.276, 0.189]	0.713	0
Study design	Publication year	**52, 1497**	**0.016 [0.003, 0.03]**	**0.017**	**31.53**	52, 1492	− 0.006 [− 0.022, 0.009]	0.416	0	45, 1063	− 0.004 [− 0.021, 0.013]	0.607	0
	Country (ref. outside US/mixed)	52, 1497	− 0.002 [− 0.139, 0.135]	0.973	0	52, 1492	− 0.024 [− 0.194, 0.145]	0.778	0	45, 1063	− 0.054 [− 0.257, 0.148]	0.598	0.03
	Sponsorship (ref. no)	50, 1469	0.078 [− 0.054, 0.21]	0.246	2.89	51, 1470	0.002 [− 0.172, 0.175]	0.984	0	43, 996	0.057 [− 0.152, 0.266]	0.590	0
	No. sites*	48, 1395	− 0.003 [− 0.008, 0.003]	0.295	0	48, 1427	− 0.004 [− 0.011, 0.003]	0.287	0	**41, 957**	− **0.025 [**− **0.045,** − **0.005]**	**0.015**	**31.62**
	% academic sites	46, 1340	− 0.196 [− 0.435, 0.044]	0.11	10.66	47, 1383	− 0.08 [− 0.397, 0.236]	0.619	0	41, 957	− 0.117 [− 0.461, 0.227]	0.505	0
	No. arms	52, 1497	− − 0.036 [− 0.208, 0.136]	0.684	0	52, 1492	0.035 [− 0.182, 0.252]	0.753	0	Insufficient data			
	Duration (weeks)	52, 1497	0.002 [− 0.007, 0.012]	0.669	0	52, 1492	− 0.009 [− 0.021, 0.003]	0.124	5.63	45, 1063	0.00 3[− 0.009, 0.015]	0.677	0
	Washout (ref. no)	46, 1404	− 0.064 [− 0.204, 0.076]	0.369	0	47, 1339	− 0.006 [− 0.165, 0.153]	0.943	0	39, 995	− 0.021 [− 0.248, 0.205]	0.855	0
	Sample size**	52, 1497	0 [− 0.001, 0.001]	0.967	0	**52, 1492**	− **0.002 [**− **0.003,** − **0.001]**	**0.004**	**29.38**	45, 1063	− 0.001 [− 0.004, 0.001]	0.213	9.89
	% participants on placebo	52, 1497	0.172 [− 0.625, 0.968]	0.673	0	52, 1492	− 0.333 [− 1.31, 0.643]	0.503	0	45, 1063	0.401 [− 0.981, 1.783]	0.569	0
	Rater (ref. clinician)***	**51, 1491**	− **0.164 [**− **0.315,** − **0.012]**	**0.034**	**21.48**	51, 1483	0.131 [− 0.033, 0.294]	0.117	12.20	43, 1009	− 0.148 [− 0.361, 0.065]	0.174	0
	Sequence generation (ref. unclear)	52, 1497	0.147 [− 0.047, 0.34]	0.138	6.46	52, 1492	− 0.043 [− 0.302, 0.216]	0.743	0	45, 1063	0.243[− 0.048, 0.533]	0.102	1.70
	Allocation concealment (ref. unclear)	52, 1497	− 0.045 [− 0.228, 0.138]	0.631	0	52, 1492	− 0.012 [− 0.233, 0.208]	0.912	0	**45, 1063**	− **0.252 [**− **0.485,** − **0.019]**	**0.034**	**17.23**
	Blinding (ref. unclear/high)	52, 1497	0.083 [− 0.068, 0.235]	0.28	0	52, 1492	0.006 [− 0.191, 0.202]	0.953	0	45, 1063	− 0.019 [− 0.269, 0.231]	0.881	0
	Missing outcome (ref. unclear/high)	52, 1497	− 0.141 [− 0.293, 0.01]	0.067	12.03	52, 1492	− 0.052 [− 0.247, 0.143]	0.6	0	45, 1063	− 0.162 [− 0.362, 0.038]	0.111	12.72
	Selective reporting (ref. unclear/high)	52, 1497	− 0.014 [− 0.254, 0.227]	0.912	0	52, 1492	− 0.156 [− 0.471, 0.160]	0.333	0.33	Insufficient data			
	Other bias (ref. unclear/high)	**52, 1497**	− **0.160 [**− **0.299,** − **0.021]**	**0.024**	**27.38**	52, 1492	− 0.091 [− 0.303, 0.120]	0.398	0	45, 1063	− 0.033 [− 0.254, 0.188]	0.768	0
Participant	Age group (ref. adults/mixed)	52, 1497	− 0.057 [− 0.27, 0.155]	0.597	0	52, 1492	− 0.107 [− 0.323, 0.109]	0.33	1.55	45, 1063	0.148 [− 0.124, 0.42]	0.287	9.35
	Mean age	51, 1478	0.002 [− 0.011, 0.014]	0.809	0	51, 1486	0.003 [− 0.008, 0.014]	0.547	0	45, 1063	− 0.007 [− 0.021, 0.007]	0.297	7.37
	% female	50, 1453	0.131 [− 0.65, 0.911]	0.743	0	50, 1482	− 0.282 − 1.114, 0.55]	0.507	0	45, 1063	0.277 [− 0.650, 1.203]	0.559	0
	% intellectual disability	17, 620	− 0.328 [− 0.672, 0.017]	0.063	42.21	21, 672	0.128 [− 0.335, 0.59]	0.588	0	14, 316	0.382 [− 0.105, 0.869]	0.124	20.87
	% Caucasian or Hispanic	29, 963	0.036 [− 0.351, 0.423]	0.855	0	29, 971	− 0.157 [− 0.715, 0.402]	0.583	0	21, 545	− 0.42 [− 1.204, 0.364]	0.294	0
	Associated conditions at baseline (ref. no)	52, 1497	− 0.068 [− 0.212, 0.075]	0.351	3.45	52, 1492	0.111 [− 0.065, 0.287]	0.216	5.83	45, 1063	− 0.012 [− 0.242, 0.218]	0.919	0
	Baseline mean BMI	12, 445	− 0.063 [− 0.156, 0.029]	0.179	19.01	12, 461	− 0.02 [− 0.132, 0.091]	0.720	0	8, 254	− 0.033 [− 0.122, 0.057]	0.476	0
	Baseline mean CGI− S	26, 917	− 0.042 [− 0.25, 0.167]	0.694	0	26, 925	0.033 [− 0.227, 0.292]	0.805	0	17, 513	− 0.077 [− 0.442, 0.288]	0.678	0
	Baseline mean ABC− Irritability	**31, 917**	− **0.017 [-0.028,** − **0.006]**	**0.002**	**100**	30, 884	− 0.005 [− 0.022, 0.013]	0.608	0	17, 340	− 0.006 [− 0.035, 0.023]	0.68	0
	Minimum threshold of core symptoms for inclusion (ref. no)**	52, 1497	0.085 [− 0.094, 0.264]	0.358	0.88	**52, 1492**	− **0.346 [**− **0.516,** − **0.175]**	**< 0.001**	**53.85**	Insufficient data			

A negative coefficient represent an increase of placebo response. For dichotomous covariates, the reference level is mentioned. Meta-regression with dichotomous covariates were not performed when there were less than five data points for a level of the covariate (e.g., placebo lead-in or number of medications). For continuous covariates, the covariate refer to a change of 1 point of the variable, e.g., per year for publication year: decrease of placebo response in social-communication difficulties by 0.016 per year, % percentage of intellectual disability: increase of placebo response in social-communication difficulties by − 0.328 from 0 to 1 (100%) of the participants had intellectual disability. *The effect of number of sites on placebo response in overall core symptoms was not significant (*k* = 40, coefficient 0.0182 [− 0.268, 0.0631], *p* = 0.4287), when one outlier study with 26 sites was excluded (Bolognani 2019). **Placebo response in repetitive behaviors was not found to be predicted by sample size (*k* = 49, coefficient − 0.001 [− 0.002, 0.000], *p* = 0.052), flexible-dosing (*k* = 48, coefficient − 0.073 [− 0.189, 0.043], *p* = 0.218) and using a minimum threshold of core symptoms (*k* = 49, coefficient − 0.083 [− 0.24, 0.073], *p* = 0.2950), when three antidepressant trials (Herscu 2019, King 2009, and Reddihough 2019) were excluded. ***In the meta-regression of type of rater, Niederhofer 2003 was not included in social-communication difficulties (aggregated caregiver/teacher rating of ABC-L/SW), Saad 2015 was not included in overall core symptoms (it was not clear if CARS was rated only by parents or also filled by clinicians), and Anagnostou_2012 was not included in overall core symptoms and repetitive behaviors (SRS and RBS-R might have been rated as self-reports)

#### Repetitive behaviors

##### Primary analysis

**Fig. 3:**
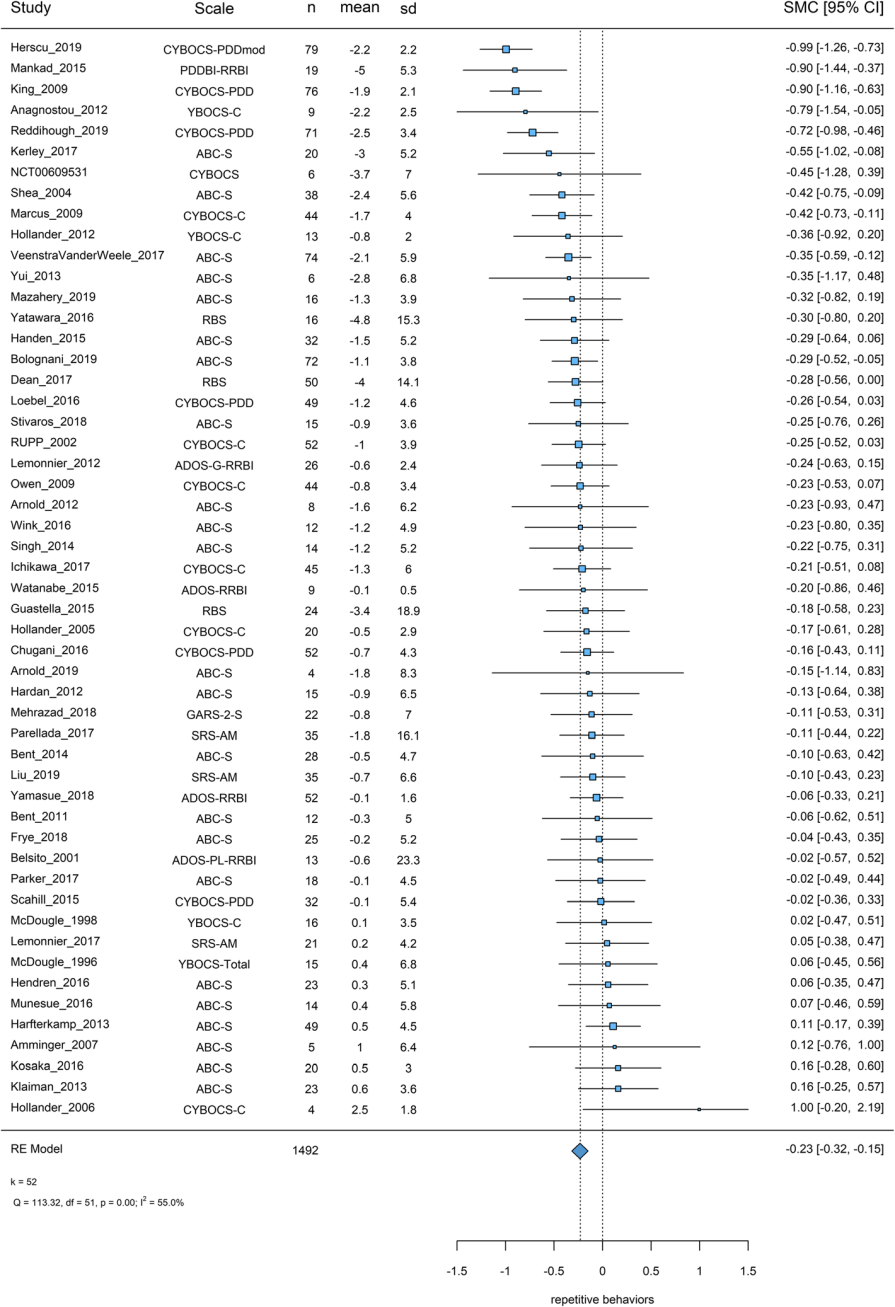
Placebo response in scales measuring repetitive behaviors. Squares and bars represent standardized mean changes (SMC) and 95% confidence intervals for each study. The size of the square is proportional to the weight of the study in the meta-analysis. The diamond represent the pooled SMC. Heterogeneity is quantified with a *χ*^2^ test (*Q*) and *I*^2^. *In Chugani 2016, standard errors might have been reported as SDs. Therefore, we calculated SDs from the reported values (no reply from the corresponding author). In Amminger 2007, ABC-S was rated by clinicians of the day care center. Scale: the scale used (clinician rated scales based on observation or interviews were preferred in the primary analysis); n: the number of participants on placebo; mean: mean change from baseline to endpoint (negative values for improvement); sd: the standard deviation used for the standardization (baseline standard deviations were preferred); SMC: standardized mean changes, 95% CI: 95% confidence intervals, *k* = total number of studies included in the analysis

Fifty-two studies were included in the primary analysis with 1492 participants. Caregivers filled about half of the scales (56%). The most frequently used scales were ABC-S (44%) and YBOCS-versions (33%). Overall placebo response was SMC = − 0.23 [− 0.32, − 0.15], and there was some heterogeneity (*I*^2^ = 55%, *χ*^2^ = 113.32, *p* < 0.001) (Fig. 3).

##### Sensitivity analysis and publication bias

Sensitivity analyses did not change the results materially, though there was a small difference between fixed and random-effects summary estimates, indicating possible small-study effects. Egger’s test was not significant and yielded a marginal *p* value (*z* = 1.71, *p* = 0.09); it has been suggested that for this test, a threshold of 0.1 should be employed. By visual inspection of the funnel plot, we detected a possible asymmetry (Additional file 3: eAppendix-6.2) and the trim-and-fill adjusted placebo response was − 0.33 [− 0.41, − 0.25].

##### Meta-regressions

Higher placebo response was associated with larger sample sizes (− 0.02 [− 0.03, − 0.01] SMC units per ten participants), flexible-dosing (− 0.195 [− 0.351, − 0.038]), and the use of a threshold of core symptoms at inclusion (− 0.346 [− 0.516, − 0.175]). These covariates remained in the multivariable model, but the use of a threshold of core symptoms at inclusion was not significant. Nevertheless, the findings might have been driven by three antidepressant trials in children/adolescents [65–67], with larger sample sizes (~ 150) and multiple sites (3, 6, and 18), as well as using flexible-dosing and a threshold of CYBOCS-PDD for inclusion (Table 1).

#### Overall core symptoms

##### Primary analysis

**Fig. 4:**
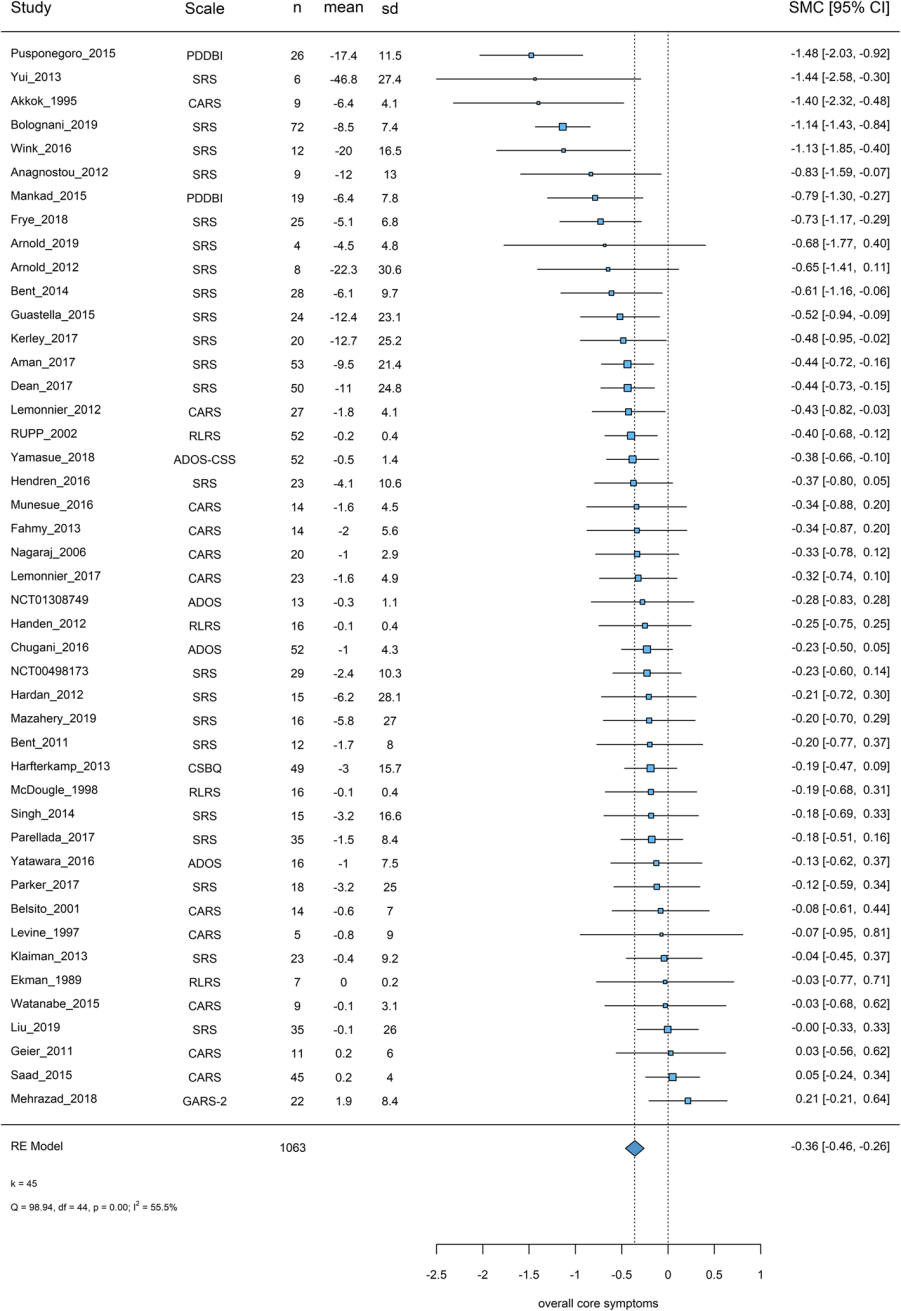
Placebo response in scales measuring overall core symptoms. Squares and bars represent standardized mean changes (SMC) and 95% confidence intervals for each study. The size of the square is proportional to the weight of the study in the meta-analysis. The diamond represent the pooled SMC. Heterogeneity is quantified with a *χ*^2^ test (*Q*) and *I*^2^. *In Anagnostou 2012, we reversed baseline and endpoint values of SRS: in the manuscript, original baseline values were lower than endpoint in both placebo and oxytocin arms, meaning an increase of severity of symptoms during the study, which is not consistent with the reported positive effect size and the other outcomes (no reply from the corresponding author), in Saad 2015, CARS was rated by caregivers but it was unclear if also filled by clinicians (no reply from the corresponding author), as well as in RUPP 2002 and Handen 2012 the Ritvo-Freeman Life Rating Scale was rated by caregivers. Scale: the scale used (clinician rated scales based on observation or interviews were preferred in the primary analysis); *n*: the number of participants on placebo; mean: mean change from baseline to endpoint (negative values for improvement); sd: the standard deviation used for the standardization (baseline standard deviations were preferred); SMC: standardized mean changes, 95% CI: 95% confidence intervals, k= total number of studies included in the analysis

Forty-five studies with 1063 participants were included in the primary analysis. Caregivers filled about half of the scales (51%). The most frequently used scales were SRS (49%) and CARS (24%). Overall placebo response was SMC = − 0.36 [− 0.46, − 0.26] and heterogeneity was considerable (*I*^2^ = 55.53%, *χ*^2^ = 98.94, *p* < 0.001) (Fig. 4).

##### Sensitivity analysis and publication bias

Sensitivity analyses did not change the results materially, no asymmetry was detected in the funnel plot, and Egger’s test yielded a marginal *p* value (*z* = − 1.82, *p* = 0.07).

##### Meta-regressions

Larger placebo response was associated with more trial sites (− 0.025 [− 0.045, − 0.005] SMC units per site, not significant when an outlier was removed) [68], and low risk of bias in allocation concealment (− 0.252 [− 0.485, − 0.019]). In the multivariable model, allocation concealment and number of sites were both significant.

Number of medications and the use of placebo lead-in had not sufficient data for all outcomes, while number of arms, selective reporting, and the use of the threshold of core symptoms did not have sufficient data for meta-regressions in overall core symptoms.

### Secondary outcomes

#### Placebo response by type of rater

Results based on scales filled by different type of raters (Additional file 3: eAppendix-6.4) were similar to those of meta-regressions by type of rater (one effect size per study, clinician ratings were preferred whenever available).

#### CGI-I positive response rates

**Fig. 5:**
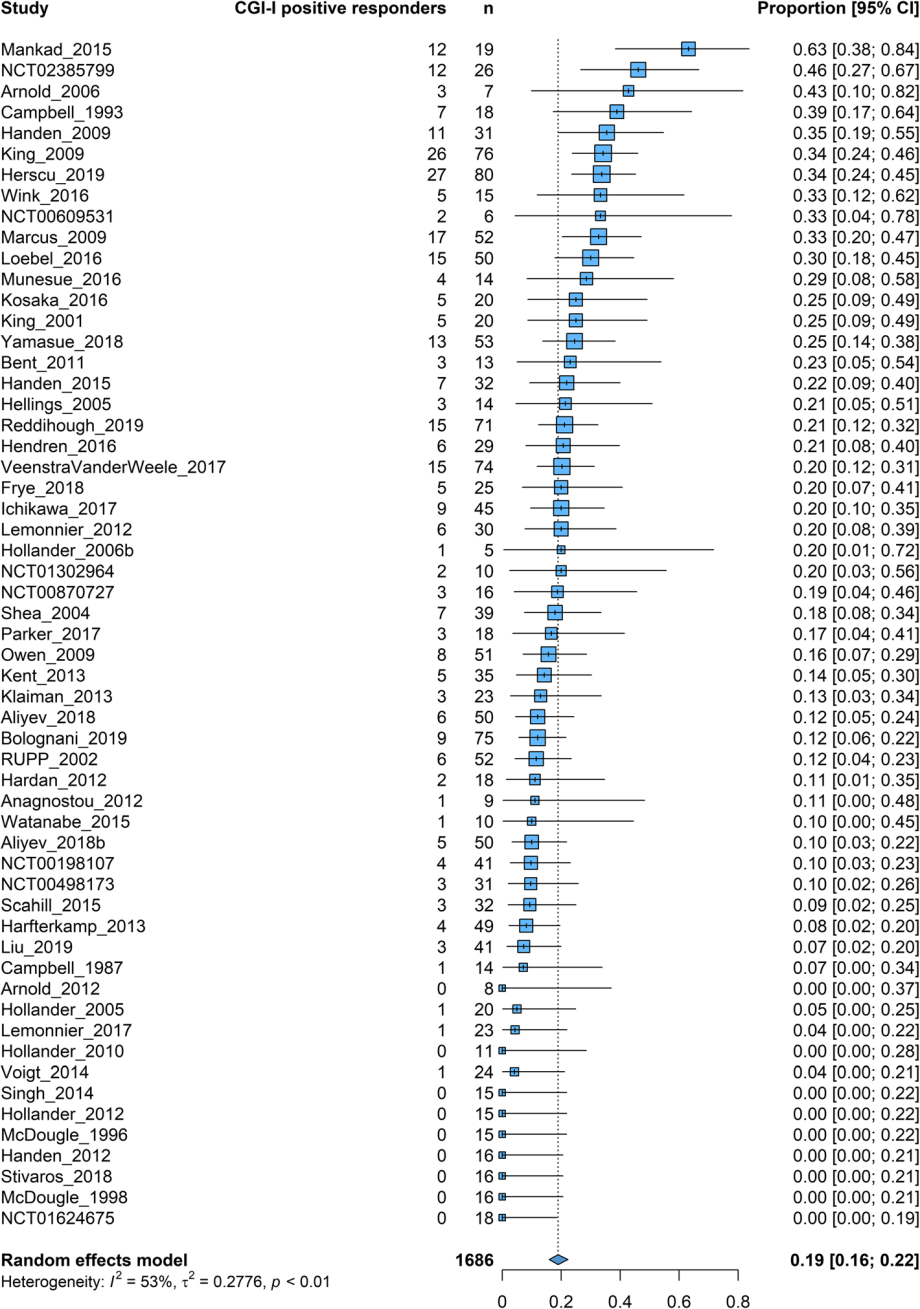
CGI-I positive placebo response. Square and bars represent the point estimate of the proportion of responders and its 95% confidence interval for each study. The size of the squares is proportional to the weight of the study. The diamonds represent the pooled proportion and its 95% confidence intervals for each subgroup and overall. Heterogeneity is quantified with a *χ*^2^ test (*Q*) and *I*^2^. CGI-I positive responders: number of participants with a positive response defined as at least much improvement in the CGI-I (if not reported, it was imputed using a validated method); Total: total number of participants on placebo

The overall positive response rate as defined by at least much improvement in the CGI-I was 19% [16–22%] (*k* = 57, *n* = 1686, *I*^2^ = 53%) (Fig. 5). The anchoring system of CGI was unclear in 35 studies, while seven considered both core and associated symptoms (three used OACIS [69]), three reported separate evaluations for global autism symptoms and for the trial target symptom, and three considered mainly core symptoms and nine associated symptoms (two reported the RUPP-framework [70]) (Table-S).

#### Post-hoc correlation analyses

##### Between covariates

Exploratory correlations between covariates are presented in Additional file 3: eAppendix-5.4. Significant correlations with a Spearman’s |ρ| > 0.5 were found between sample size and number of sites (*ρ* = 0.77), percentage of academic sites and sponsorship (*ρ* = − 0.52), as well as number of sites (*ρ* = − 0.51), risk of bias domain of sequence generation and the domain of selective reporting (*ρ* = 0.57), number of arms and percentage of participants on placebo (*ρ* = − 0.54), and between other covariates with a large proportion of missing data, e.g., baseline ABC-Irritability, BMI, CGI-S, and percentage of participants with intellectual disability (Additional file 3: eAppendix-5.3).

##### Between placebo and drug response

SMCs of placebo and experimental intervention were correlated in social-communication difficulties (Spearman’s *ρ* = 0.525, *p* < 0.001) and overall core symptoms (*ρ* = 0.539, *p* < 0.001), but no correlation was found in repetitive behaviors (*ρ* = 0.233, *p* = 0.096) (Additional file 3: eAppendix-6.5).

## Discussion

In pharmacological and dietary supplement ASD trials, placebo response was substantial and comparable among core symptoms; about 20% of the participants were at least much improved with placebo. We found potential predictors of larger placebo response in at least one symptom domain, i.e., baseline irritability, the use of a threshold of core symptoms at inclusion, caregiver ratings, larger sample size and number of sites, lower risk of bias, flexible-dosing, and less recent publication year.

### Predictors of placebo response

#### Participant-related factors

It has been argued that placebo response might be larger in children/adolescents than adults [71]. We did not find a difference between age groups or an effect of mean age. Nonetheless, extrapolations between age groups should be interpreted with caution because the majority of studies were in pediatric populations (87%). Other participant characteristics did not predict placebo response (e.g., sex, ethnicity, BMI, intellectual disability).

Low baseline severity has been found to predict placebo response in most psychiatric conditions [50]. We did not find an effect of baseline global severity (CGI-S), yet available data were sparse (baseline CGI-S was reported in less than half studies, *k* = 38, 44%) and narrowly ranged between 3.88 and 6 (Additional file 3: eAppendix-5.1); also because most of the studies required participants to be at least moderately ill (i.e., CGI-S ≥ 4). Baseline severity in core symptoms could not be analyzed as a potential predictor due to the large diversity of scales. On the other hand, we found that trials using a cut-off of core symptoms for inclusion might have a larger placebo response in repetitive behaviors, yet this association was not significant in a multivariable meta-regression and it might have been driven by three antidepressant trials that used a cut-off of the clinician-administered scale CYBOCS-PDD [65–67]. Trials that utilize a baseline score cut-off could be prone to regression to the mean effects as well as baseline score inflation, especially for clinician-administered scales and under participant recruitment pressure [72]. These effects could be partially avoided by using different scales at assessing participants for inclusion and as primary outcomes [73], yet this might be challenging given the lack of optimal scales in ASD. Centralized raters blind to inclusion criteria might also reduce baseline inflation and increase inter-rater reliability, yet the execution of the trial could become complicated [72]. Since inflated scores are usually very close to the inclusion cut-off, a potential solution could be that the primary analysis is conducted by including participants with a higher cut-off (that is blinded to the investigators) than the inclusion cut-off [74].

The presence of an associated condition was required as inclusion criteria in about one-third of the trials (29 out of 86), and it was not found to predict placebo response. Nevertheless and since co-occurring symptoms and diagnoses are highly prevalent in participants with ASD [5], it can be expected that participants in other studies had also associated symptoms of varying levels. Accordingly, the median of baseline ABC-Irritability was 17.18 IQR [13.71–22.70], while normative data suggested a mean of 12.8 [75]. Thus, our sample in general could be consisted of participants with somewhat higher levels of irritability. Indeed, the most frequently investigated associated condition in our sample was irritability (*k* = 15) and the presence of an associated condition was correlated to baseline ABC-Irritability (*ρ* = 0.49, *p* < 0.001, Additional file 3: eAppendix-5.4). We found that baseline ABC-Irritability was associated with a larger placebo response in social-communication difficulties, yet this association was not significant in a multivariable meta-regression. The contrary was found in a quite large trial (*n* = 149) investigating citalopram for repetitive behaviors, yet participants had lower levels of irritability (mean ABC-Irritability = 11.2) [9]. Additionally, a small 8-week observational study investigating the effects of participation in a study protocol suggested that placebo-effects may be mainly observed in children with higher levels of irritability [76]. Such participation effects could be decreased by a screening phase with adequate duration, which could also investigate the stability of symptoms and incorporate a potential washout of psychotropic drugs. However, no effect was found for the use of a washout phase and there were not enough data to investigate the use of a placebo lead-in phase, which is in general not recommended [72].

### Design- and intervention-related factors

Caregiver ratings seemed to be more prone to placebo response in social-communication difficulties, but the effect was not consistent in multivariable meta-regressions. It has been argued that placebo-by-proxy effects are important components of placebo response in child/adolescent psychiatry, since they can alter caregiver perception of symptoms (thus improving directly scores in caregiver scales), and/or modify caregiver behaviors toward children and subsequently improving symptoms (thus improving scores also in non-caregiver scales) [71, 77]. In addition, many of the existing scales were not designed to measure change but rather as screening (e.g., SRS [31]) or diagnostic tools (CARS [32] and ADOS [78]), and efforts have been made for their improvement and adaptation, such as the ADOS calibrated severity score [79]. Given the lack of optimal scales, CGI has been extensively used and it is recommended for all trials irrespective of their target in order to investigate global autism symptoms and incorporate both core and associated symptoms [80, 81]. However, the anchoring system of CGI should be clearly reported, since it could vary materially among trials with different target symptoms (Table-S).

Therefore, there is a critical need to develop standardized and sensitive measures of core symptoms, which do not solely depend on caregivers [82, 83]. The semi-structured interview of VABS might be a promising measure of change in social-communication difficulties [33], with potential sensitivity to detect efficacy [68, 84] and empirically derived cut-offs of minimal-clinical-important differences [85]. Recent instruments have also been developed, among others the Brief Observation of Social Communication Change (BOSCC) [86, 87], the Autism Behavior Inventory [88], and the Autism Impact Measure (AIM) [89], but their utilization is yet to be determined. Patient- (or parent-) reported outcomes have also gained recently greater attention [90], yet they should not be considered immune to placebo-effects [91]. The utilization of scales that require more extensive training and experience (e.g., ADOS, BOSCC, and VABS) might be challenging in larger scale trials, and thus a low inter-rater reliability could increase the variance of measurements and subsequently decrease drug-placebo differences. A notable example is the multi-center arbaclofen trial [84], in which VABS should have been completed by the same clinician and caregiver for each participant. However, there was quite low adherence to the protocol (rater change in about 25% of the participants), potentially because VABS-Socialization was a secondary outcome, not expected to be sensitive in the context of the trial. A post-hoc per-protocol analysis of no rater change found a significant improvement of arbaclofen in comparison to placebo, in contrast to the non-significant difference of the primary analysis [84]. Therefore, proper training of the raters and inter-rater reliability of the measurements as well as guidance and adherence to the protocol should be ensured, especially in multi-site trials.

Sample size and number of sites have been suggested as predictors of placebo response [50, 51, 92]. We also found that a larger sample size was associated with a larger placebo response in repetitive behaviors, yet the results might be driven by three antidepressant trials [65–67]. This association could also be explained by a potential publication bias and the small-study effects found in the funnel plot (see Additional file 3: eAppendix-6.2), since the results of less precise trials with larger placebo response in repetitive behaviors might have been not published. Additionally, sample size was closely related to the number of sites (Spearman’s *ρ* = 0.77, *p* < 0.001, see Additional file 3: eAppendix-6), which predicted placebo response in overall core symptoms, yet the latter was driven by another outlier study with 26 sites [68]. Trials with more sites were more frequently industry-sponsored (*ρ* = 0.27, *p* = 0.04) and consisted of less academic sites (*ρ* = − 0.51, *p* < 0.001). It should be noted though that the majority of included studies were single-center (median number of sites 1 IQR [1–4]), had academic sites (about 83% consisted only of academic sites), and small sample sizes (median 45 IQR [30–91]); therefore, the results could not be extrapolated to a wider range of potential values. Nevertheless, more sites and the recruitment of non-academic professional sites, which could have less experience and enroll competitively, might increase variability, be prone to less rigorous participant selection and baseline score inflation [73, 74, 92]. Therefore, trials should be well powered, yet extremely large sample sizes could be avoided, as well as sites should be carefully selected and their number should be kept at the minimum feasible.

Studies with low risk of bias in other biases (mainly baseline imbalance) and allocation concealment were associated with larger placebo response in social-communication and overall core symptoms, respectively. It is intriguing that studies with a better quality in terms of risk of bias might have a larger placebo response. However, the above risk of bias domains evaluate the randomization process, and in inadequately randomized trials, control groups might have a poorer prognosis [93].

The association between dosing schedule and placebo response can be puzzling, e.g., both flexible- [94] and fixed-dosing schedules [95] have been associated with larger placebo responses in depression. We found an association between flexible-dosing and larger placebo response in repetitive behaviors, yet it was driven by three antidepressant trials [65–67]. Flexible-dosing could allow dose optimization guided by clinical response and/or the occurrence of side effects. The dose titration schedule and criteria as well as the starting dose and dose ranges should be carefully selected in the context of large placebo responses. For example, in one the aforementioned antidepressant trials, large placebo responses (> 25% reduction from baseline in CYBOCS-PDD) might have impeded dose escalation from a low starting dose (2 mg of fluoxetine) to a stable appropriate dose (> 10 mg) for sufficient duration of treatment (> 4 weeks) [67]. On the other hand, dose-response studies are a special type of fixed-dosing studies that might be prone to larger placebo responses. They are multi-arm and participants have an increased chance to receive active medication, as well as larger sample sizes and multiple sites are usually required. These factors have been associated with a larger placebo response in psychiatry [50], yet not all of them were replicated in our analysis, probably due to the limited number of studies with those characteristics. A notable example is the dose-response study of aripiprazole [96], which had a placebo positive response rate of 33% in comparison to 16% in the similarly designed but flexible-dosing study [97] (Fig. 3). However, this has not always been observed, such as in risperidone trials, i.e., 14% in the dose-response study [98] in comparison to 12% [53] and 18% [99] in the flexible-dose studies.

Country of origin and type of experimental intervention (pharmacological or dietary supplement) was not found to predict placebo response, in contrast to a previous meta-analysis [11], which included also many Iranian trials with risperidone-combined treatments that were excluded from our review (combination treatments such as risperidone + placebo were excluded, see Additional file 3: eAppendix-4). Therefore, the findings in the previous meta-analysis could have been confounded by larger responses in combined placebo groups, i.e., response of risperidone + placebo.

There is no clear consensus about the adequate trial duration and half of the included studies had a duration of at least 12 weeks, yet the duration of the trial should be based on the mechanism of action of the experimental intervention and a longer duration could be required in order to observe sustained changes in core symptoms [100]. We did not find an effect of trial duration, yet shorter-term trials have been associated with larger placebo response in psychiatry [50]. However, in longer-term trials including young children, anticipated developmental trajectories could also explain placebo effects and subsequently mask drug-placebo differences [101]. Therefore, developmentally based scales might be necessary to overcome this challenge [82] as well as trial designs could include additional follow-up assessments in order to confirm stability of improvement [101].

In most psychiatric disorders, placebo response has increased over a period of 60 years [49, 50, 102], but this trend was not replicated in ASD trials, which were more recent, mainly published between 2009 and 2017. Even, placebo response in social-communication difficulties might have decreased over years. However, this effect was not found when ABC-Irritability was included in multivariable meta-regression. Temporal changes in the definition of ASD and research practices might play an important role per se, as differences between ASD and neurotypical populations might have been decreased over the years [103].

## Limitations

Our analysis has limitations. First, our analysis focused on placebo response in core symptoms of pharmacological and dietary supplement interventions. Therefore, we did not investigate placebo response in associated symptoms or of psychological/behavioral or multimodal interventions, which could also be of interest. However, core symptoms was the apparent focus in about 40% of the included trials, while many trials focused on associated symptoms, mainly irritability or ADHD symptoms. Second, there was a large diversity of scales used as well as a wide variability of their use, e.g., different CGI-I anchoring systems. Third, moderators of drug-placebo differences were not investigated and efforts to minimize placebo response could also affect drug response, since they were correlated in social-communication difficulties and overall core symptoms, but not in repetitive behaviors (Additional file 3: eAppendix-6.5). In addition, some predictors might have a different impact on placebo and drug response [51]. Nevertheless, a more fine-grained analysis was impeded by the use of diverse experimental interventions with essentially different mechanisms of action (Additional file 3: eAppendix-5.1), e.g., contrary to schizophrenia [49, 51, 102], for which antipsychotics are the cornerstone of treatment [104].

Fourth, a common estimated pre-post correlation was used, but effect sizes were not materially changed in sensitivity analyses (Additional file 3: eAppendix-6.1). Fifth, despite the large number of eligible studies, about half did not provide data in spite of our efforts (authors of 85% of included studies published after 1990 could be contacted, with a reminder e-mail in case of no response, and 17% of them provided additional data/clarifications, Additional file 3: eAppendix-4), and a priori we did not use data from the whole crossover period (in forty trials), in order to avoid carry-over effects [14]. Sixth, due to the fact that information for many predictors, especially for participant-related factors (Additional file 3: eAppendix-5), was missing in many studies, we could not employ a full multivariable meta-regression and we focused on a series of univariable meta-regressions. Therefore, we cannot exclude the possibility of omitted variable bias in the results, i.e., the fact that the effect of the omitted variables may be added to the predictor considered in the univariable meta-regression. It should be noted that meta-regressions of aggregate data have an observational nature and they are prone to ecological fallacy, thus our findings should be considered exploratory and hypothesis-generating, considering also that there was no adjustment to multiple testing. Accordingly, individual-participant-data meta-analysis could allow for a more fine-grained analysis and further elucidate the impact of participant-level factors, such as age, sex, as well as baseline severity of core/associated symptoms.

## Conclusions

In order to increase the detection of efficacy of experimental interventions for ASD, high-quality and adequately powered trials are required, and predictors of placebo response should be considered. Extremely large sample sizes could be avoided and when multiple sites are needed, they should be carefully selected, trained, and monitored as well as their number should be kept at the minimum feasible. This would also facilitate a more rigorous selection of participants and a higher inter-rater reliability of measurements. Furthermore, scales that do not solely depend on caregiver reports could be selected as primary outcomes, since placebo-by-proxy effects are expected. Nevertheless, our findings highlight the urgent need for optimal and developmentally-based measures of change in core symptoms [82, 83]. The mechanism of action of the experimental intervention could guide the selection of an appropriate, yet sufficiently long, trial duration as well as of the dose schedule and dose ranges. Participant-related factors, such as age, sex, and baseline severity of core/associated symptoms as well as factors that could differentially moderate drug response warrant further investigation. Last, in order to facilitate comparability between studies and synthesis of evidence, trials should better characterize their participants and improve their reporting, including the CGI anchoring system.

## Supplementary information

**Additional file 1.**

**Additional file 2.**

**Additional file 3.**

## Data Availability

All data generated during this study are included in this published article (and its supplementary information files). The datasets analyzed during the current study are available from the corresponding author on reasonable request.

## Abbreviations

ABC-L/SW: Aberrant Behavior Checklist-Lethargy/Social Withdrawal
ABC-S: Aberrant Behavior Checklist-Stereotypic Behavior
ADHD: Attention-deficit/hyperactivity disorder
ADI-R: Autism Diagnostic Interview-Revised
ADOS: Autism Diagnostic Observation Scale
ASD: Autism spectrum disorders
BOSCC: Brief Observation of Social Communication Change
BMI: Body mass index
CARS: Childhood Autism Rating Scale
CGI: Clinical global impression
(C)YBOCS-PDD: (Children) Yale Obsessive Compulsive Scale-Pervasive Developmental Disorders
DSM: Diagnostic and Statistical Manual of Mental Disorders
ICD: International Classification of Diseases
RLRS: Ritvo-Freeman Real Life Rating Scale
SD: Standard deviation
SMC: Standardized mean change
SRS: Social Responsiveness Scale
VABS: Vineland Adaptive Behavior Scale
