# Supplementary material for: Placebo response in pharmacological and dietary supplement trials of autism spectrum disorder (ASD): systematic review and meta-regression analysis
Source: Mol Autism. 2020 Aug 26;11:66. doi: 10.1186/s13229-020-00372-z (PMC7448339; doi:10.1186/s13229-020-00372-z)

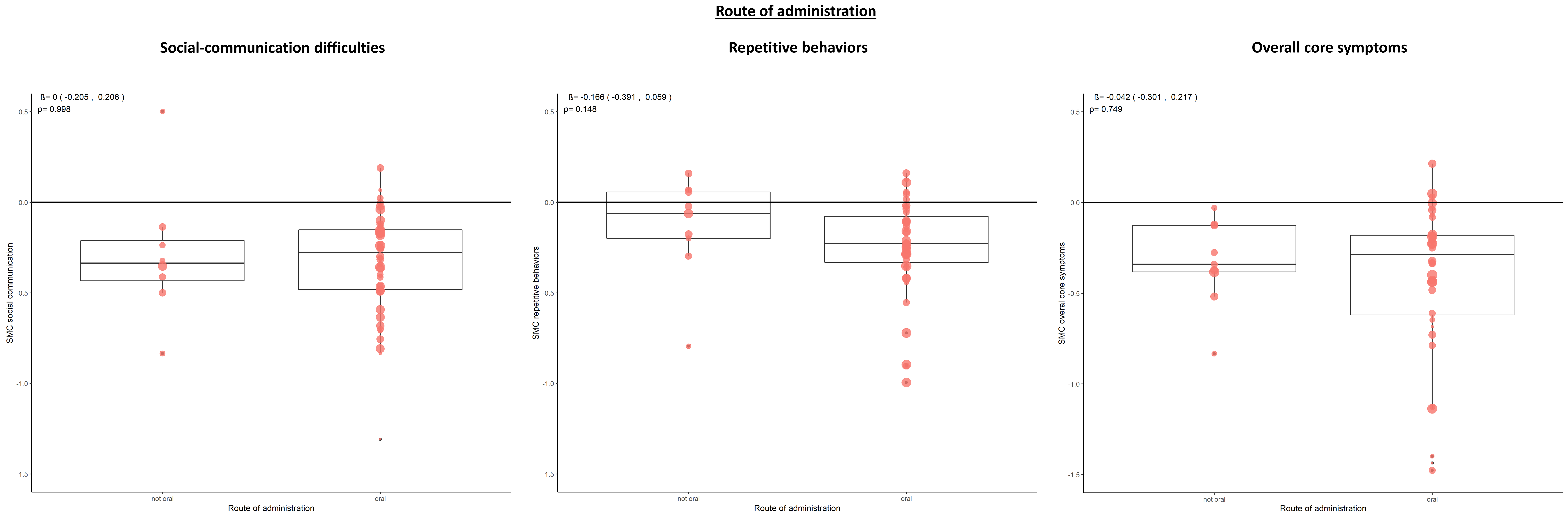

Route of administration
Social-communication difficulties
Repetitive behaviors
Overall core symptoms
ß= 0 (-0.205 , 0.206)
p= 0.998
ß= -0.166 (-0.391 , 0.059)
p= 0.148
ß= -0.042 (-0.301 , 0.217)
p= 0.749
SMC social communication
SMC repetitive behaviors
SMC overal core symptoms
0.5
0.0
-0.5
-1.0
-1.5
not oral
oral
Route of administration

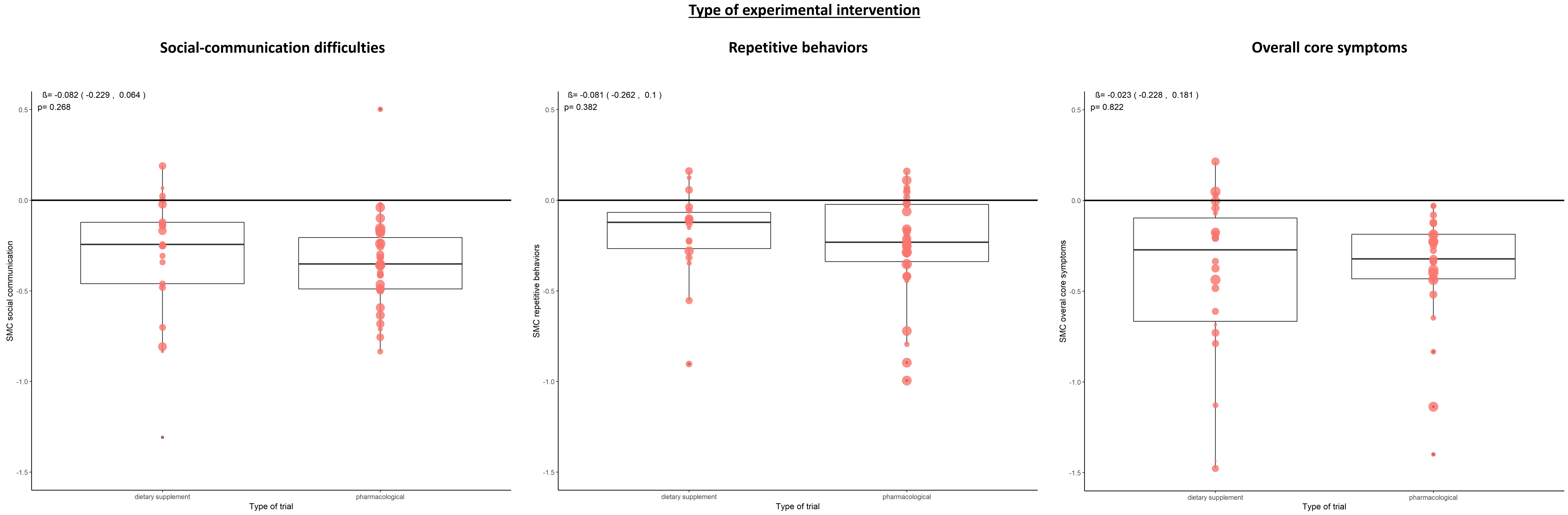

Type of experimental intervention
Social-communication difficulties
ß= -0.082 (-0.229 , 0.064)
p= 0.268
SMC social communication
dietary supplement
pharmacological
Type of trial
Repetitive behaviors
ß= -0.081 (-0.262 , 0.1)
p= 0.382
SMC repetitive behaviors
dietary supplement
pharmacological
Type of trial
Overall core symptoms
ß= -0.023 (-0.228 , 0.181)
p= 0.822
SMC overal core symptoms
dietary supplement
pharmacological
Type of trial

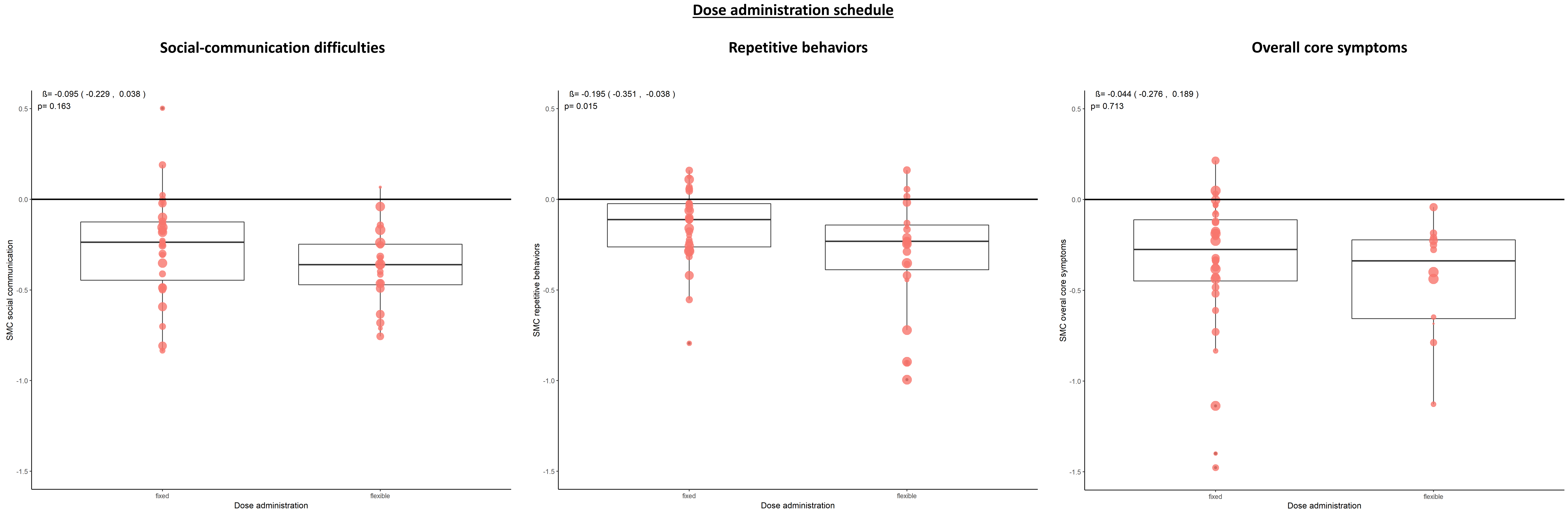

Dose administration schedule
Social-communication difficulties
Repetitive behaviors
Overall core symptoms
ß= -0.095 (-0.229 , 0.038)
p= 0.163
ß= -0.195 (-0.351 , -0.038)
p= 0.015
ß= -0.044 (-0.276 , 0.189)
p= 0.713
SMC social communication
SMC repetitive behaviors
SMC overal core symptoms
0.5
0.0
-0.5
-1.0
-1.5
fixed
flexible
Dose administration

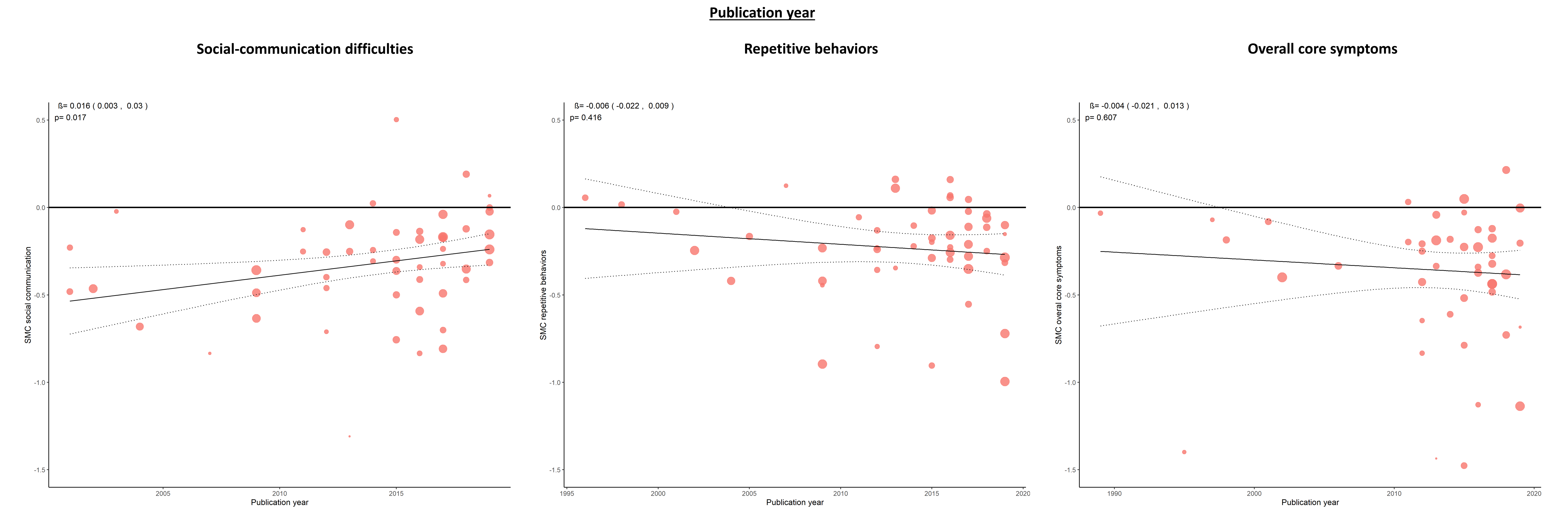
Publication year
Social-communication difficulties
ß= 0.016 (0.003 , 0.03)
p= 0.017
SMC social communication
Publication year
Repetitive behaviors
ß= -0.006 (-0.022 , 0.009)
p= 0.416
SMC repetitive behaviors
Publication year
Overall core symptoms
ß= -0.004 (-0.021 , 0.013)
p= 0.607
SMC overal core symptoms
Publication year

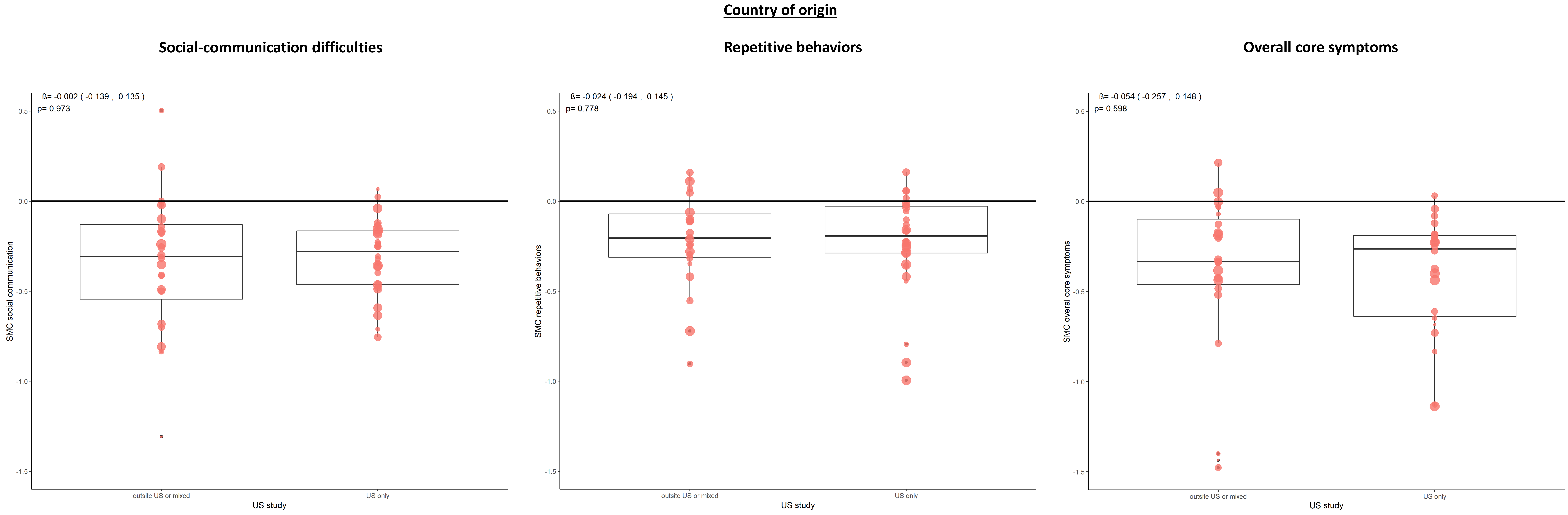
Country of origin
Social-communication difficulties
ß= -0.002 (-0.139 , 0.135)
p= 0.973
SMC social communication
0.5
0.0
-0.5
-1.0
-1.5
outside US or mixed
US only
US study
Repetitive behaviors
ß= -0.024 (-0.194 , 0.145)
p= 0.778
SMC repetitive behaviors
0.5
0.0
-0.5
-1.0
-1.5
outside US or mixed
US only
US study
Overall core symptoms
ß= -0.054 (-0.257 , 0.148)
p= 0.598
SMC overal core symptoms
0.5
0.0
-0.5
-1.0
-1.5
outside US or mixed
US only
US study

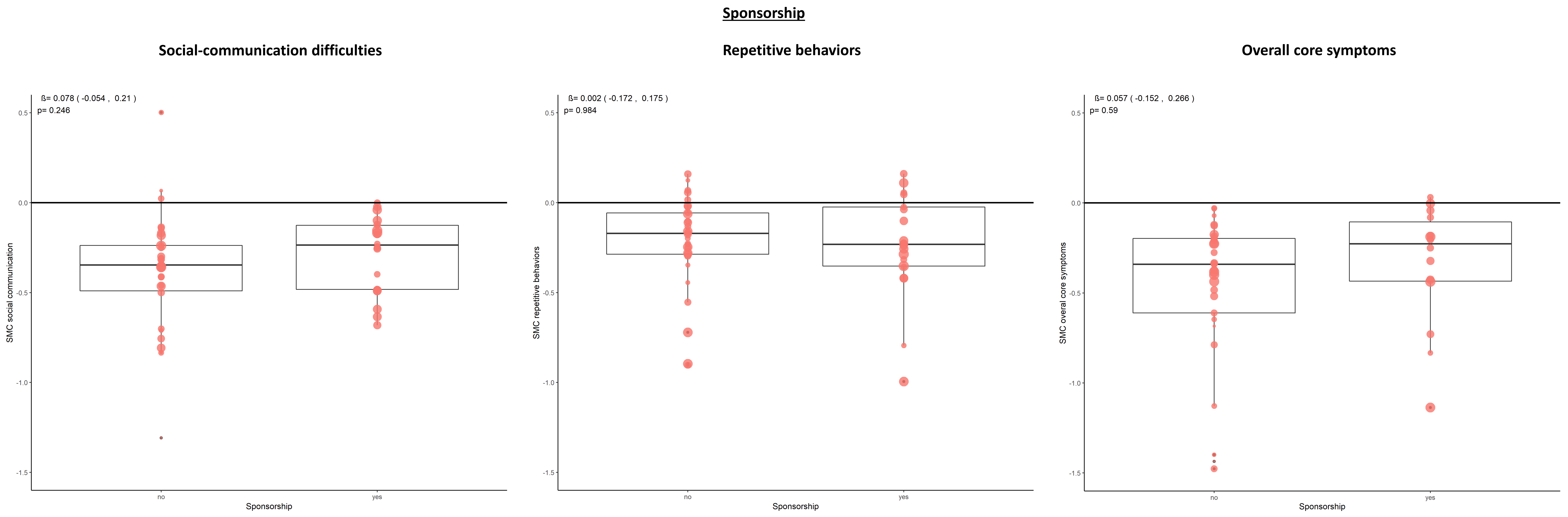

Sponsorship
Social-communication difficulties
ß= 0.078 (-0.054 , 0.21)
p= 0.246
SMC social communication
0.5
0.0
-0.5
-1.0
-1.5
no
yes
Sponsorship
Repetitive behaviors
ß= 0.002 (-0.172 , 0.175)
p= 0.984
SMC repetitive behaviors
0.5
0.0
-0.5
-1.0
-1.5
no
yes
Sponsorship
Overall core symptoms
ß= 0.057 (-0.152 , 0.266)
p= 0.59
SMC overal core symptoms
0.5
0.0
-0.5
-1.0
-1.5
no
yes
Sponsorship

Number of sites

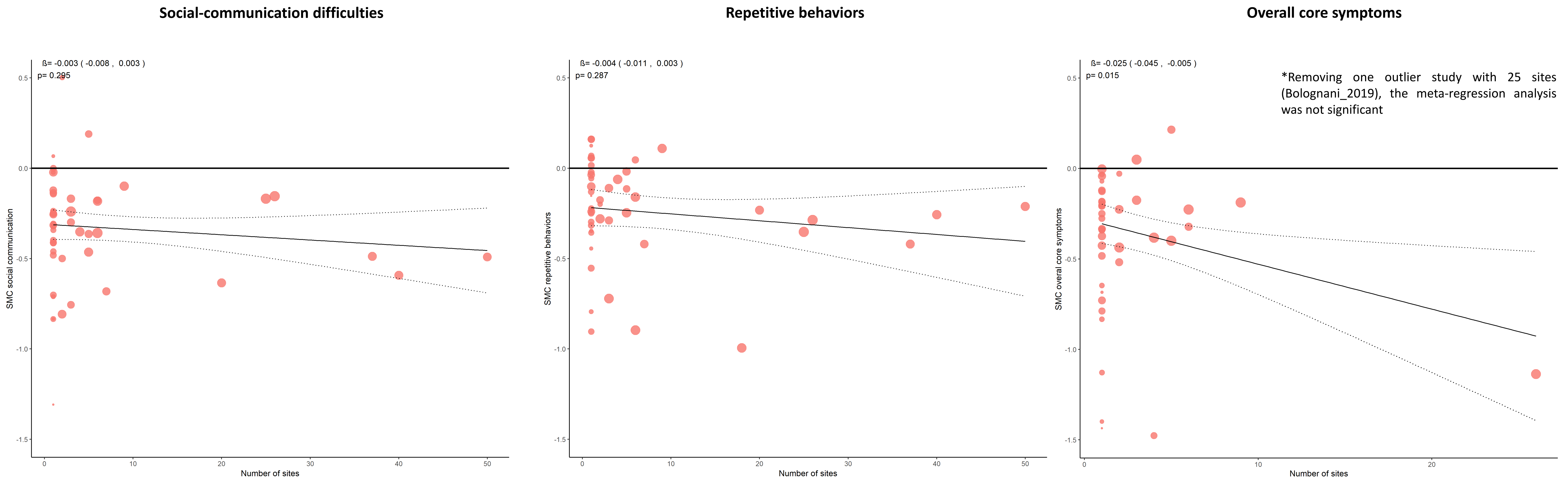

% academic sites

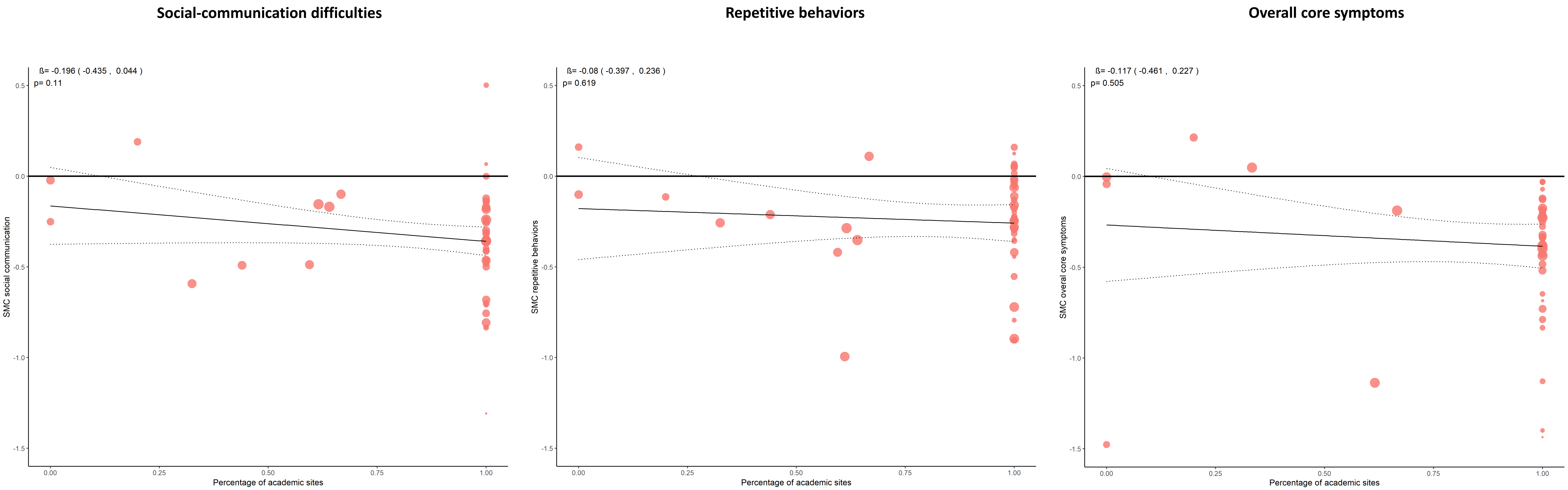

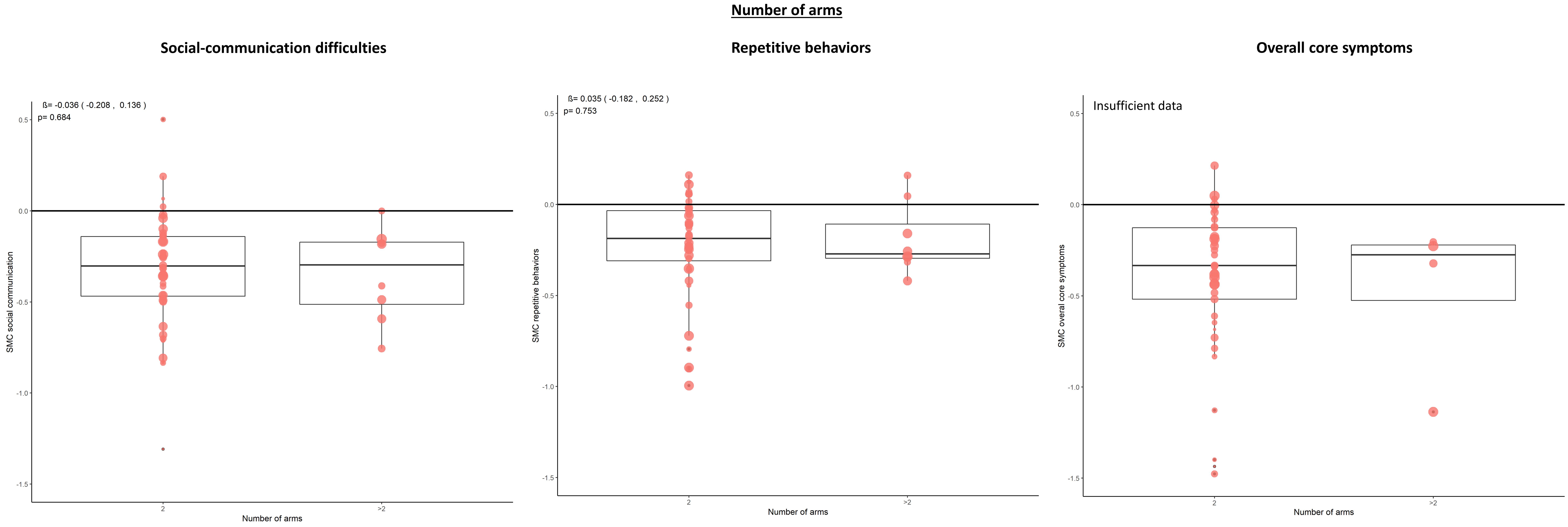

Number of arms
Social-communication difficulties
ß= -0.036 (-0.208 , 0.136)
p= 0.684
SMC social communication
Number of arms
Repetitive behaviors
ß= 0.035 (-0.182 , 0.252)
p= 0.753
SMC repetitive behaviors
Number of arms
Overall core symptoms
Insufficient data
SMC overal core symptoms
Number of arms

Number of medications

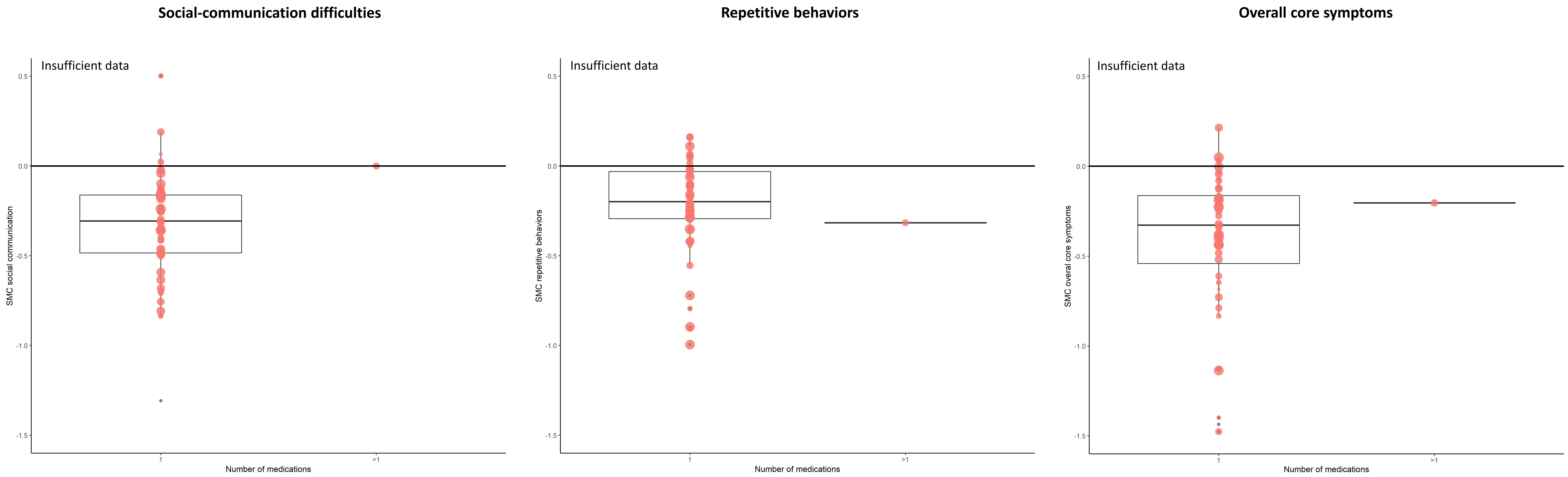

Duration (weeks)

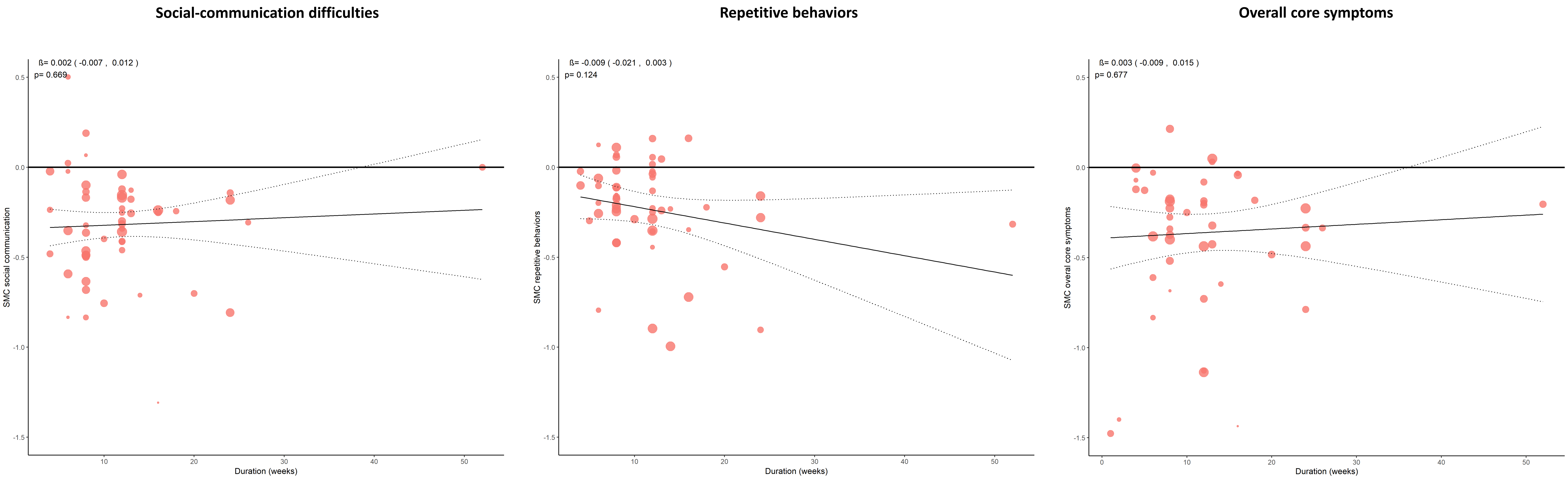

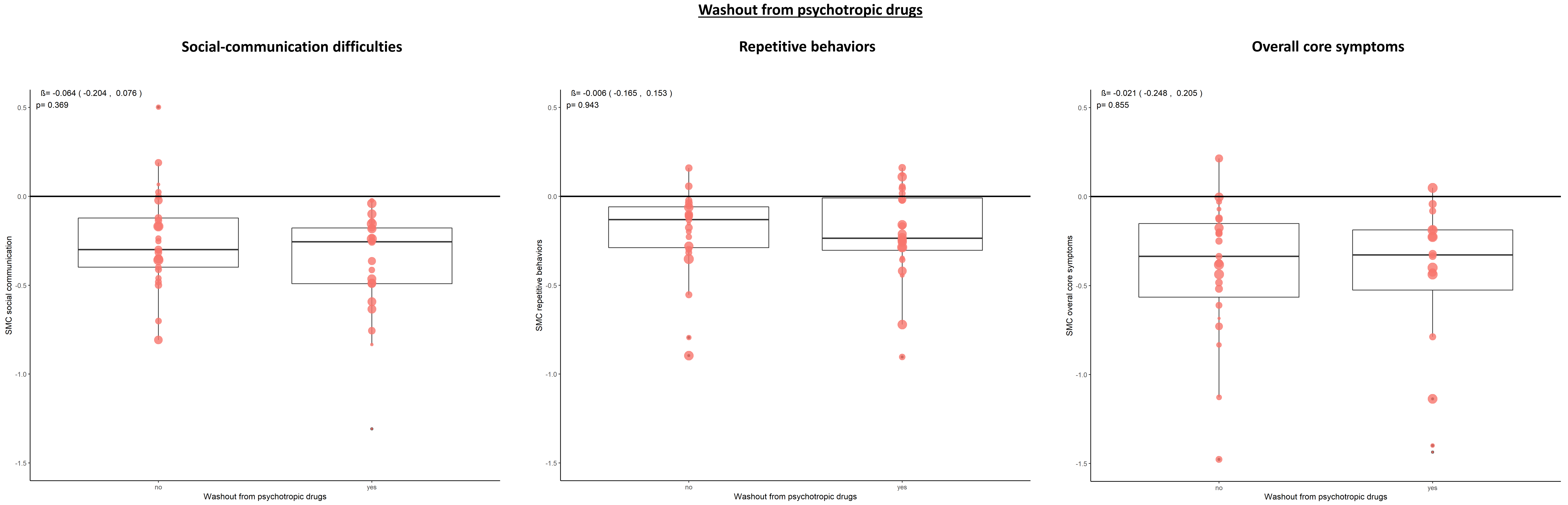

Washout from psychotropic drugs
Social-communication difficulties
Repetitive behaviors
Overall core symptoms
ß= -0.064 (-0.204 , 0.076)
p= 0.369
ß= -0.006 (-0.165 , 0.153)
p= 0.943
ß= -0.021 (-0.248 , 0.205)
p= 0.855
SMC social communication
SMC repetitive behaviors
SMC overal core symptoms
0.5
0.0
-0.5
-1.0
-1.5
no
yes
Washout from psychotropic drugs

Placebo lead-in with exclusion of placebo responders

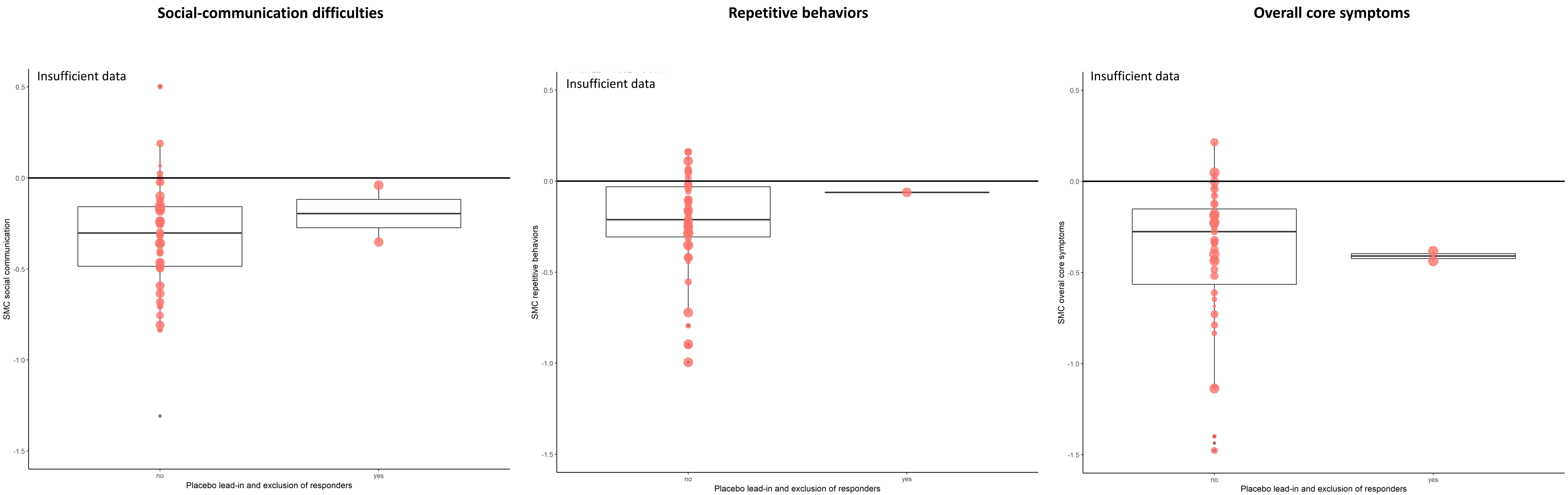

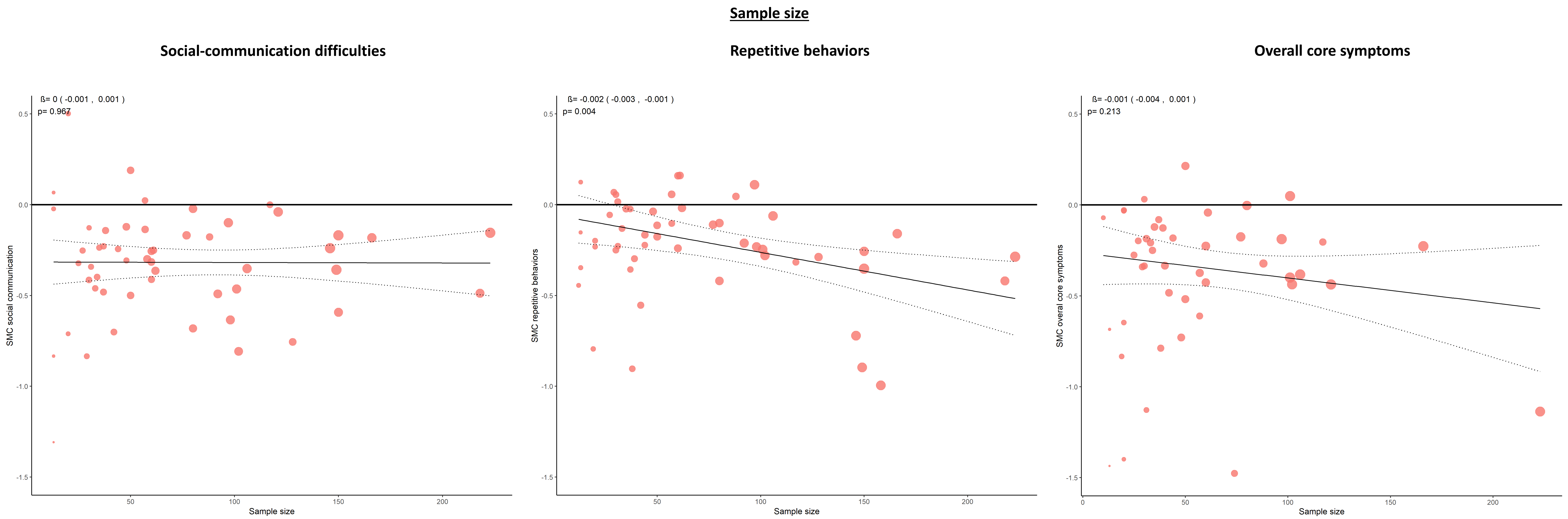

Sample size
Social-communication difficulties
Repetitive behaviors
Overall core symptoms
ß= 0 (-0.001 , 0.001)
p= 0.967
ß= -0.002 (-0.003 , -0.001)
p= 0.004
ß= -0.001 (-0.004 , 0.001)
p= 0.213
SMC social communication
SMC repetitive behaviors
SMC overal core symptoms
Sample size

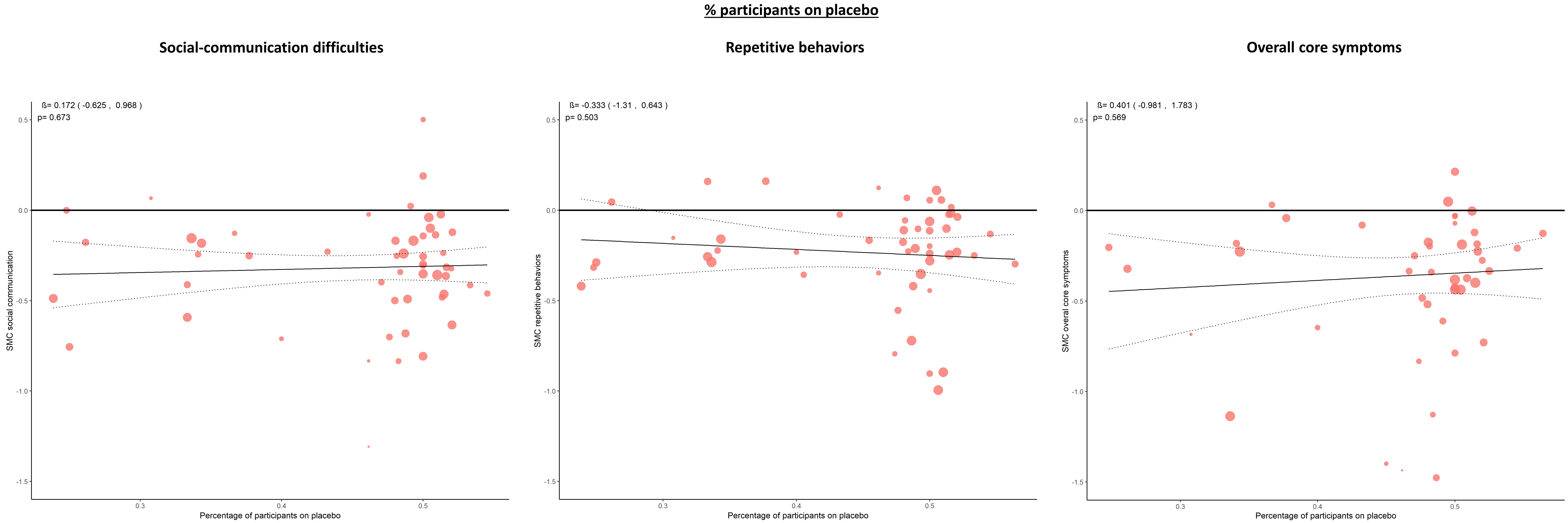
% participants on placebo
Social-communication difficulties
Repetitive behaviors
Overall core symptoms
ß= 0.172 (-0.625 , 0.968)
p= 0.673
ß= -0.333 (-1.31 , 0.643)
p= 0.503
ß= 0.401 (-0.981 , 1.783)
p= 0.569
SMC social communication
SMC repetitive behaviors
SMC overal core symptoms
Percentage of participants on placebo
0.5
0.0
-0.5
-1.0
-1.5
0.3
0.4
0.5

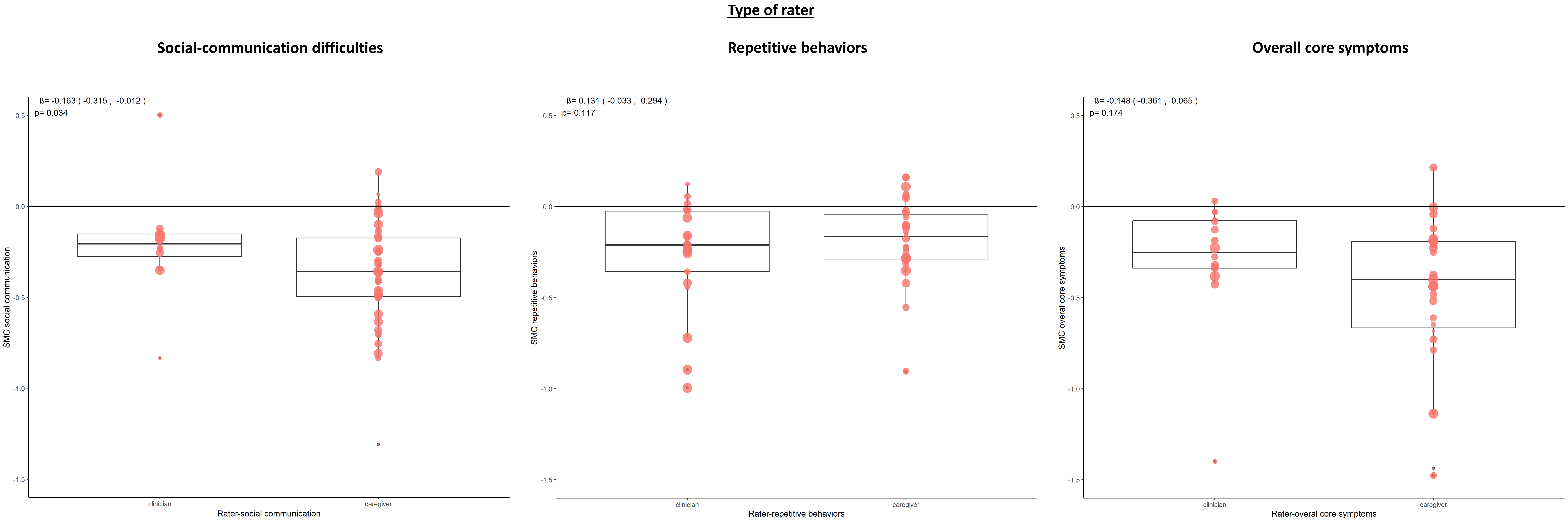
Type of rater
Social-communication difficulties
ß= -0.163 (-0.315 , -0.012)
p= 0.034
SMC social communication
clinician
caregiver
Rater-social communication
Repetitive behaviors
ß= 0.131 (-0.033 , 0.294)
p= 0.117
SMC repetitive behaviors
clinician
caregiver
Rater-repetitive behaviors
Overall core symptoms
ß= -0.148 (-0.361 , 0.065)
p= 0.174
SMC overal core symptoms
clinician
caregiver
Rater-overal core symptoms
0.5
0.0
-0.5
-1.0
-1.5

Random sequence generation

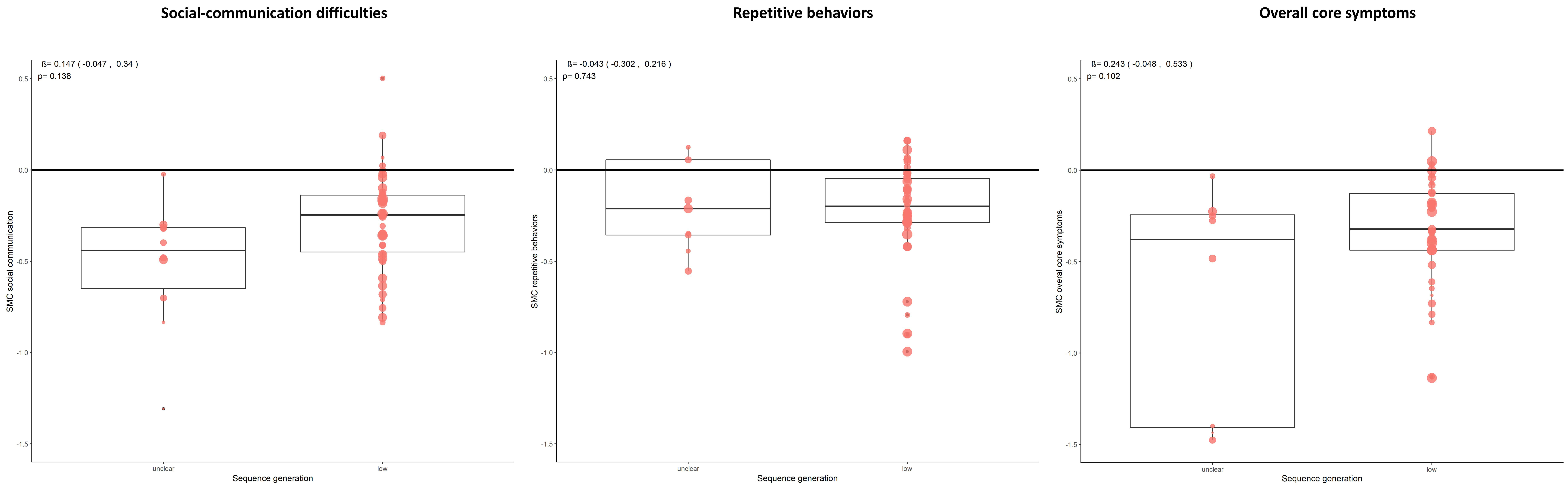

Allocation concealment

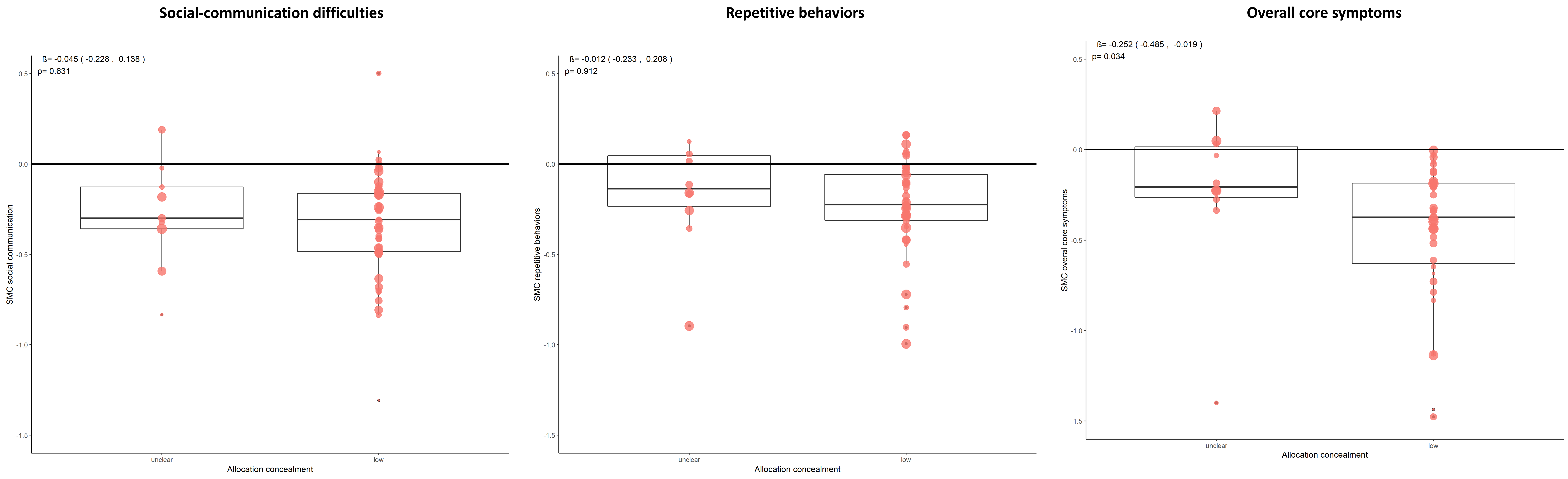

Blinding

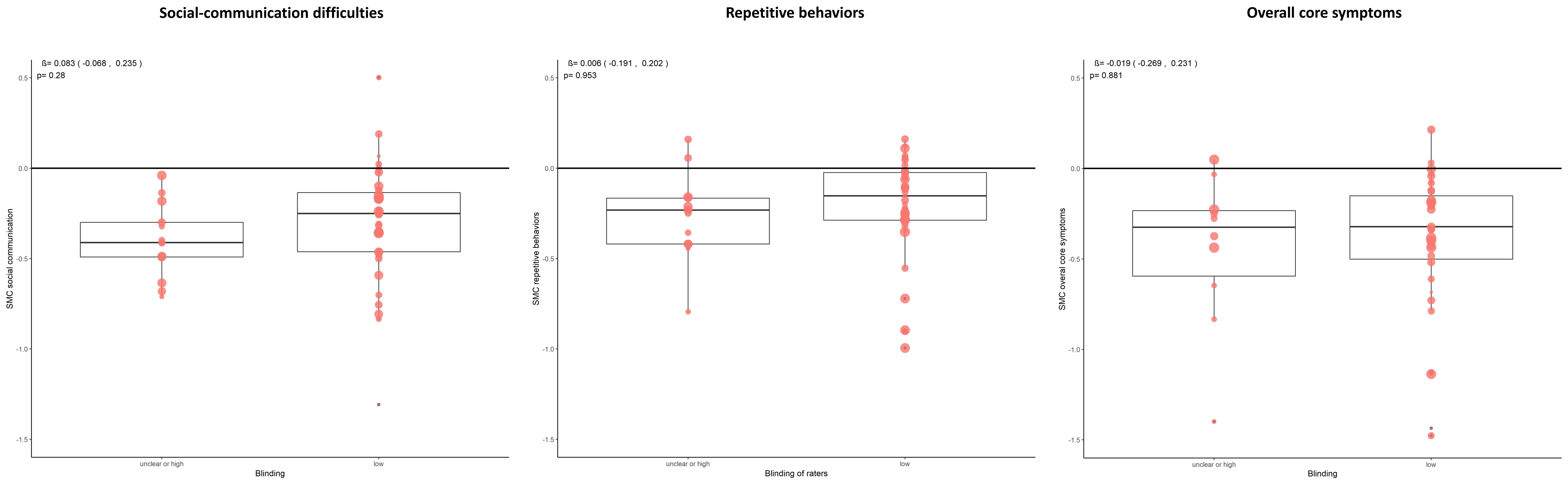
Social-communication difficulties
ß= 0.083 (-0.068 , 0.235)
p= 0.28
SMC social communication
Blinding
unclear or high
low
Repetitive behaviors
ß= 0.006 (-0.191 , 0.202)
p= 0.953
SMC repetitive behaviors
Blinding of raters
unclear or high
low
Overall core symptoms
ß= -0.019 (-0.269 , 0.231)
p= 0.881
SMC overal core symptoms
Blinding
unclear or high
low
0.5
0.0
-0.5
-1.0
-1.5

Missing outcome data

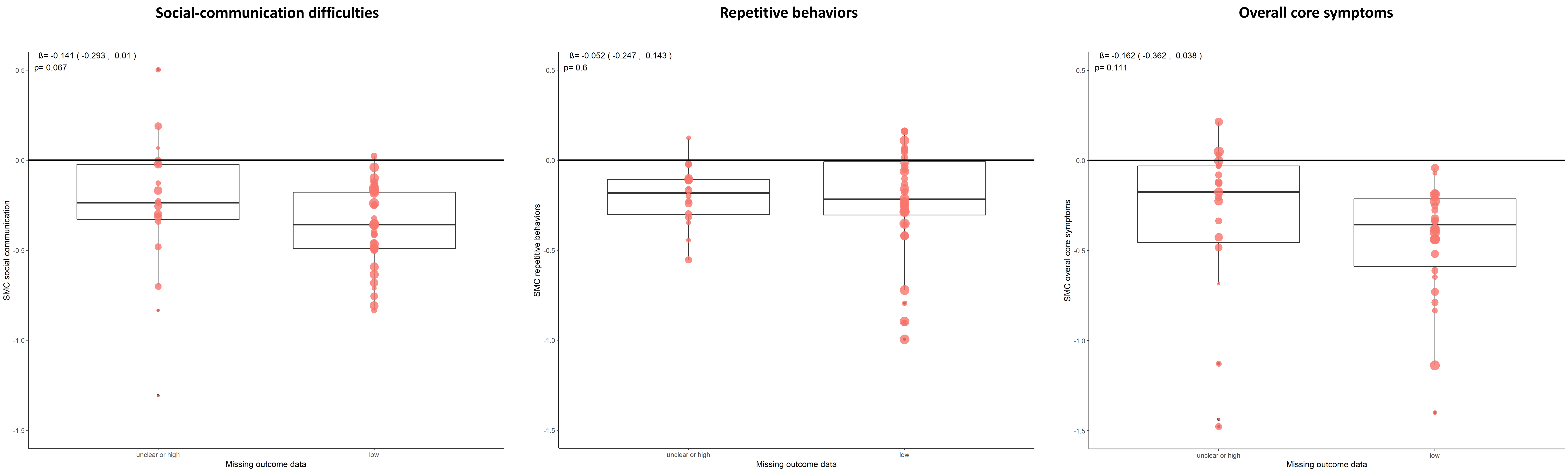

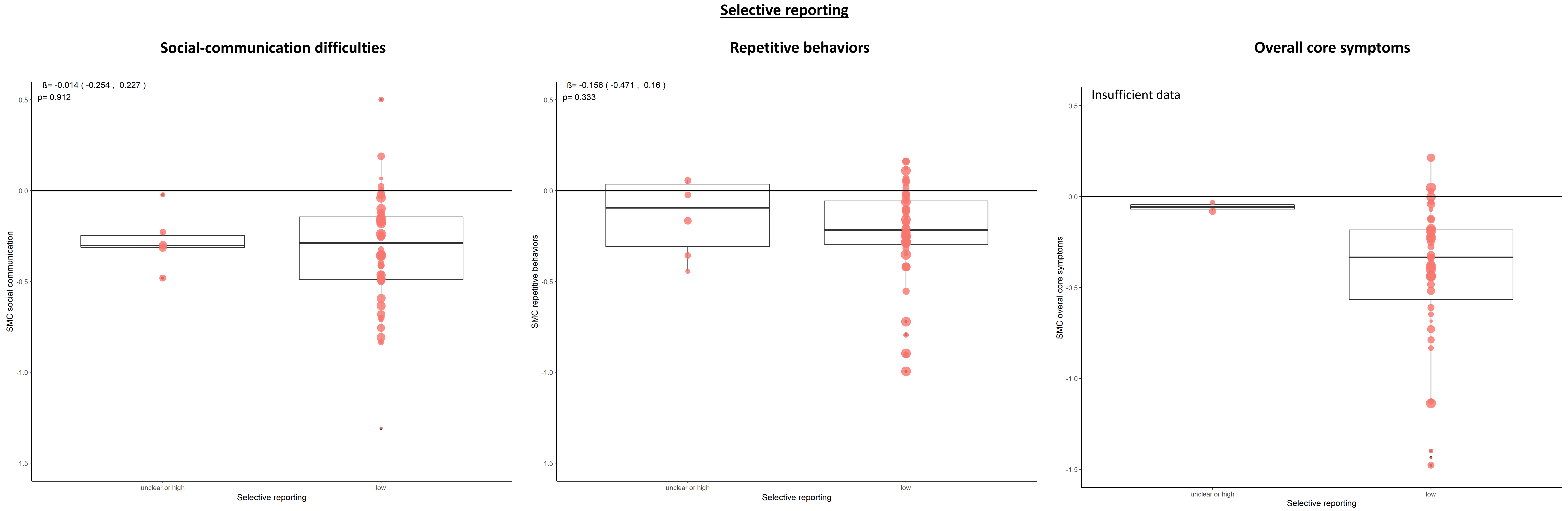
Selective reporting
Social-communication difficulties
ß= -0.014 (-0.254 , 0.227)
p= 0.912
SMC social communication
unclear or high
low
Selective reporting
Repetitive behaviors
ß= -0.156 (-0.471 , 0.16)
p= 0.333
SMC repetitive behaviors
unclear or high
low
Selective reporting
Overall core symptoms
Insufficient data
SMC overal core symptoms
unclear or high
low
Selective reporting

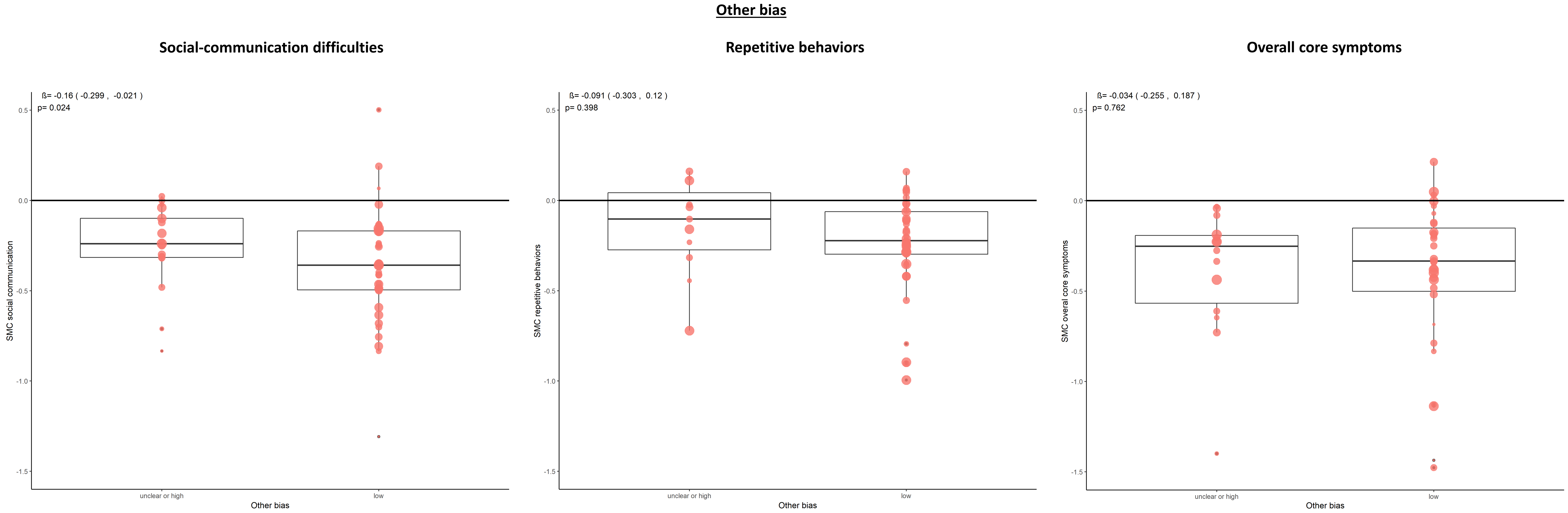
Other bias
Social-communication difficulties
ß= -0.16 (-0.299 , -0.021)
p= 0.024
SMC social communication
0.5
0.0
-0.5
-1.0
-1.5
unclear or high
low
Other bias
Repetitive behaviors
ß= -0.091 (-0.303 , 0.12)
p= 0.398
SMC repetitive behaviors
0.5
0.0
-0.5
-1.0
-1.5
unclear or high
low
Other bias
Overall core symptoms
ß= -0.034 (-0.255 , 0.187)
p= 0.762
SMC overal core symptoms
0.5
0.0
-0.5
-1.0
-1.5
unclear or high
low
Other bias

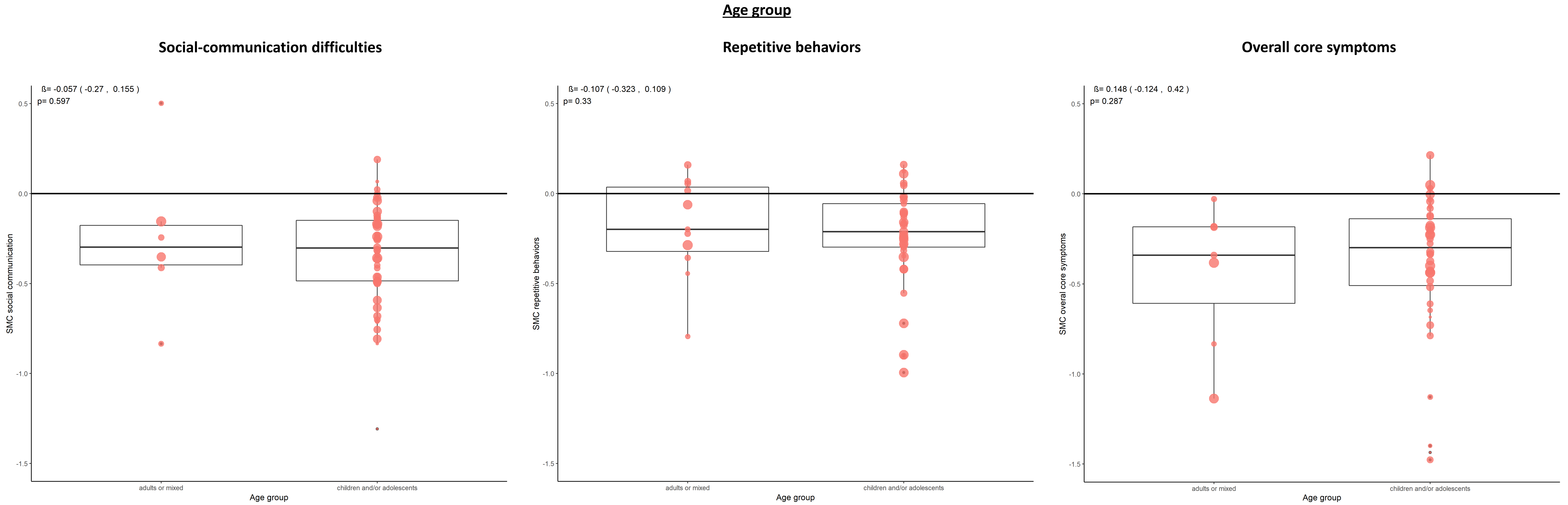

Age group
Social-communication difficulties
Repetitive behaviors
Overall core symptoms
ß= -0.057 (-0.27 , 0.155)
p= 0.597
ß= -0.107 (-0.323 , 0.109)
p= 0.33
ß= 0.148 (-0.124 , 0.42)
p= 0.287
SMC social communication
SMC repetitive behaviors
SMC overal core symptoms
0.5
0.0
-0.5
-1.0
-1.5
adults or mixed
children and/or adolescents
Age group

Mean age

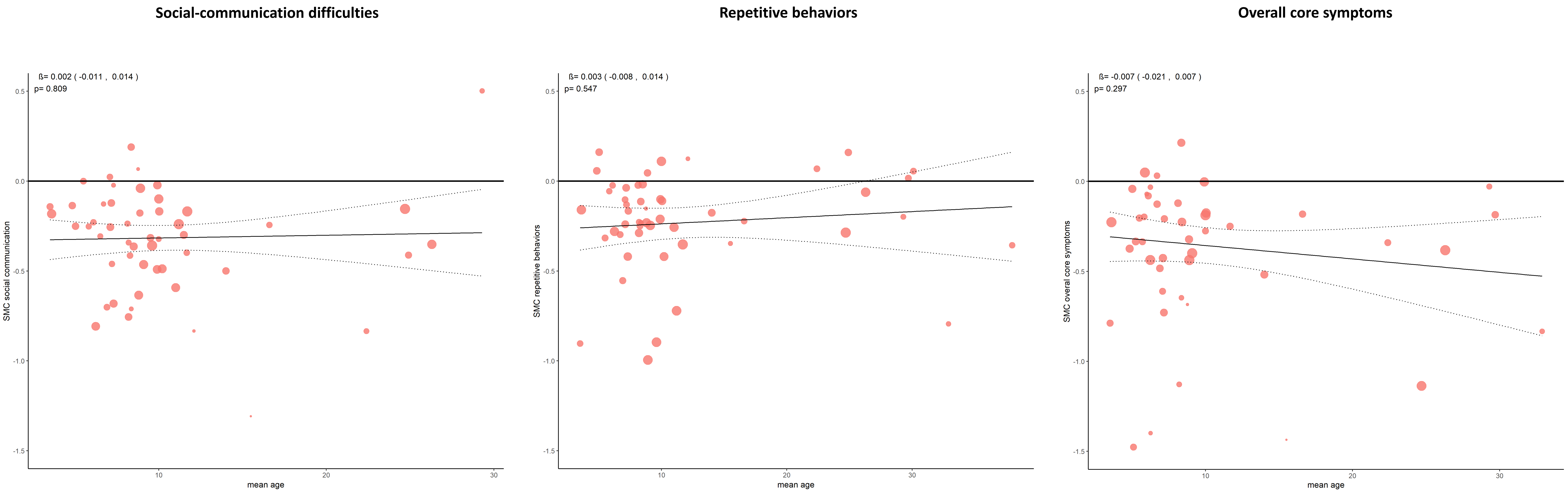

Social-communication difficulties
ß= 0.002 (-0.011 , 0.014)
p= 0.809
SMC social communication
mean age
Repetitive behaviors
ß= 0.003 (-0.008 , 0.014)
p= 0.547
SMC repetitive behaviors
mean age
Overall core symptoms
ß= -0.007 (-0.021 , 0.007)
p= 0.297
SMC overal core symptoms
mean age

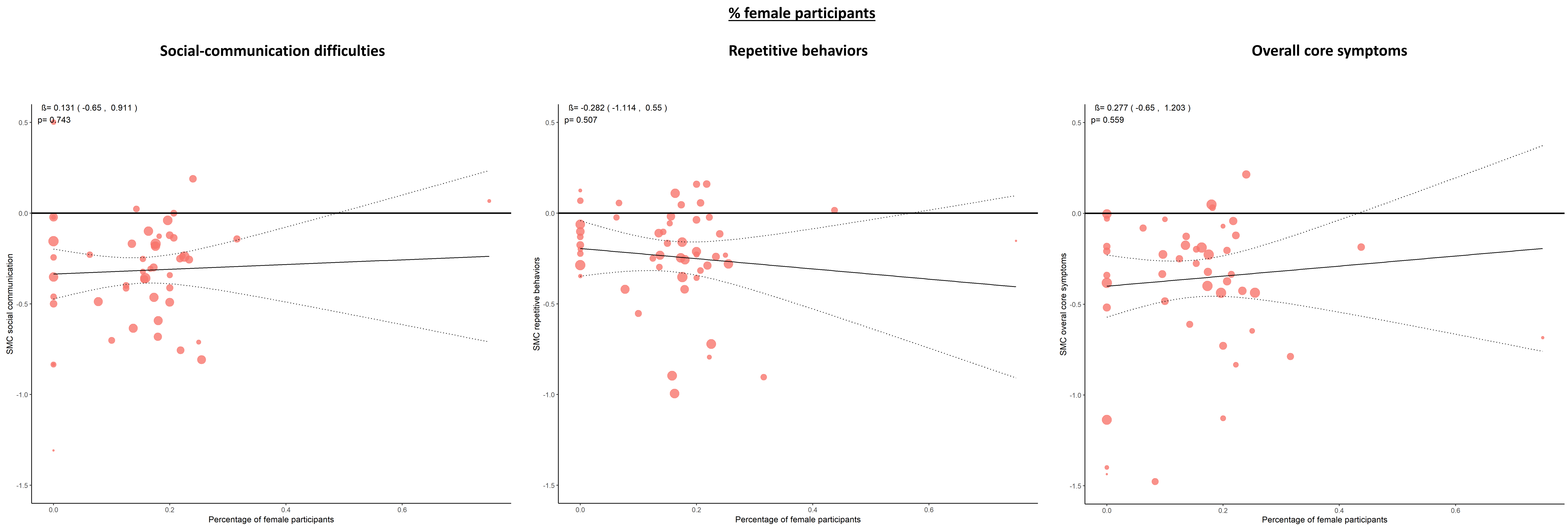
% female participants
Social-communication difficulties
ß= 0.131 (-0.65 , 0.911)
p= 0.743
SMC social communication
Percentage of female participants
Repetitive behaviors
ß= -0.282 (-1.114 , 0.55)
p= 0.507
SMC repetitive behaviors
Percentage of female participants
Overall core symptoms
ß= 0.277 (-0.65 , 1.203)
p= 0.559
SMC overal core symptoms
Percentage of female participants

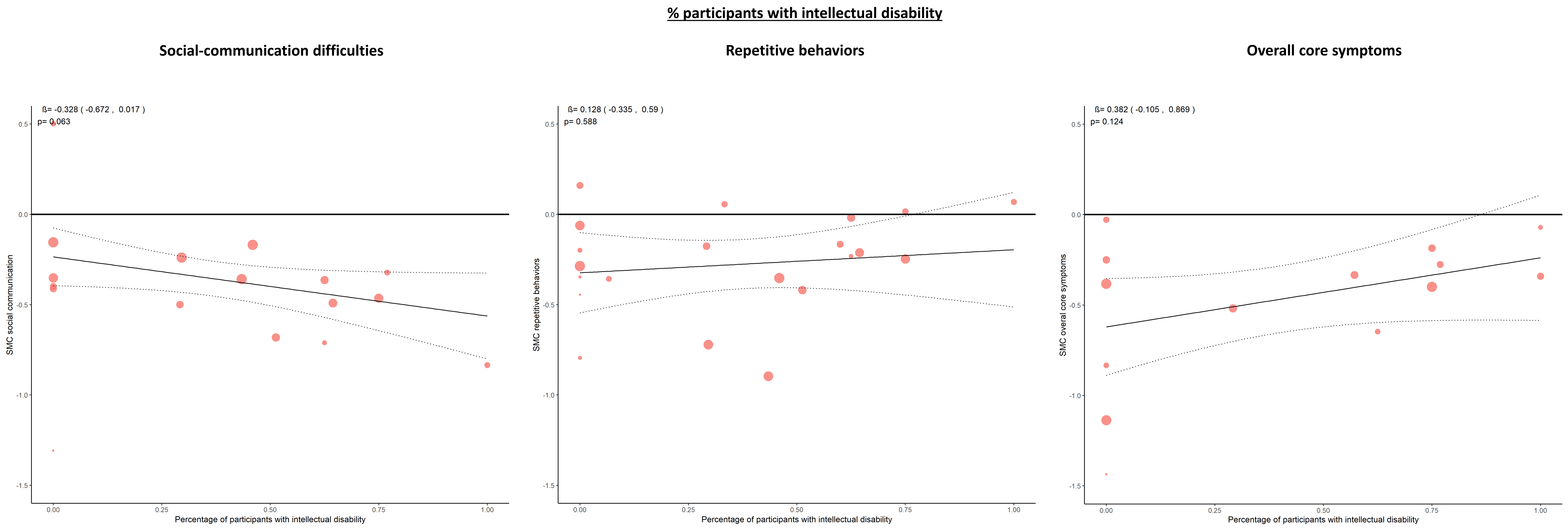
% participants with intellectual disability
Social-communication difficulties
ß= -0.328 (-0.672 , 0.017)
p= 0.063
SMC social communication
Percentage of participants with intellectual disability
Repetitive behaviors
ß= 0.128 (-0.335 , 0.59)
p= 0.588
SMC repetitive behaviors
Percentage of participants with intellectual disability
Overall core symptoms
ß= 0.382 (-0.105 , 0.869)
p= 0.124
SMC overal core symptoms
Percentage of participants with intellectual disability

% Caucasian or Hispanic participants

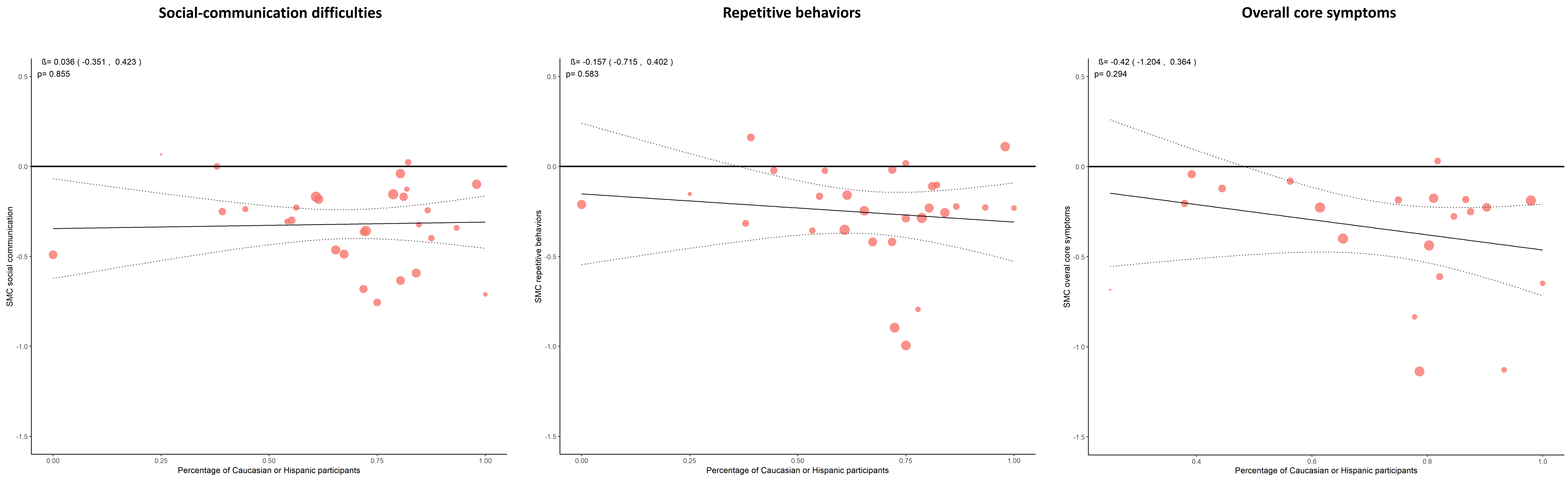

Associated conditions

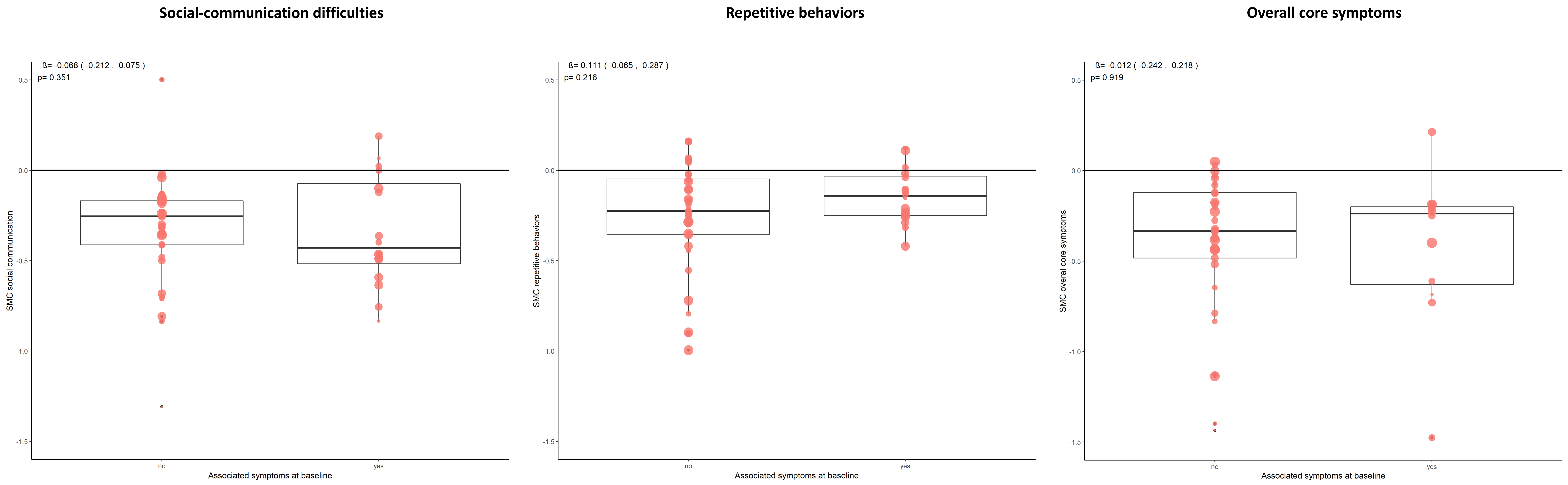

Baseline BMI

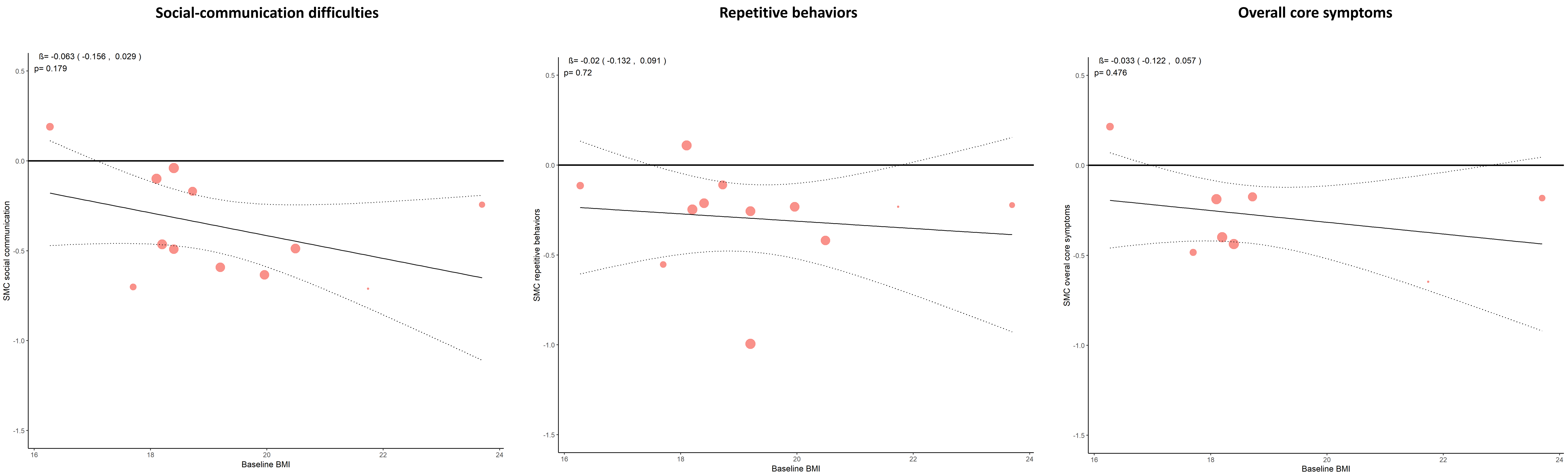

Baseline CGI-S

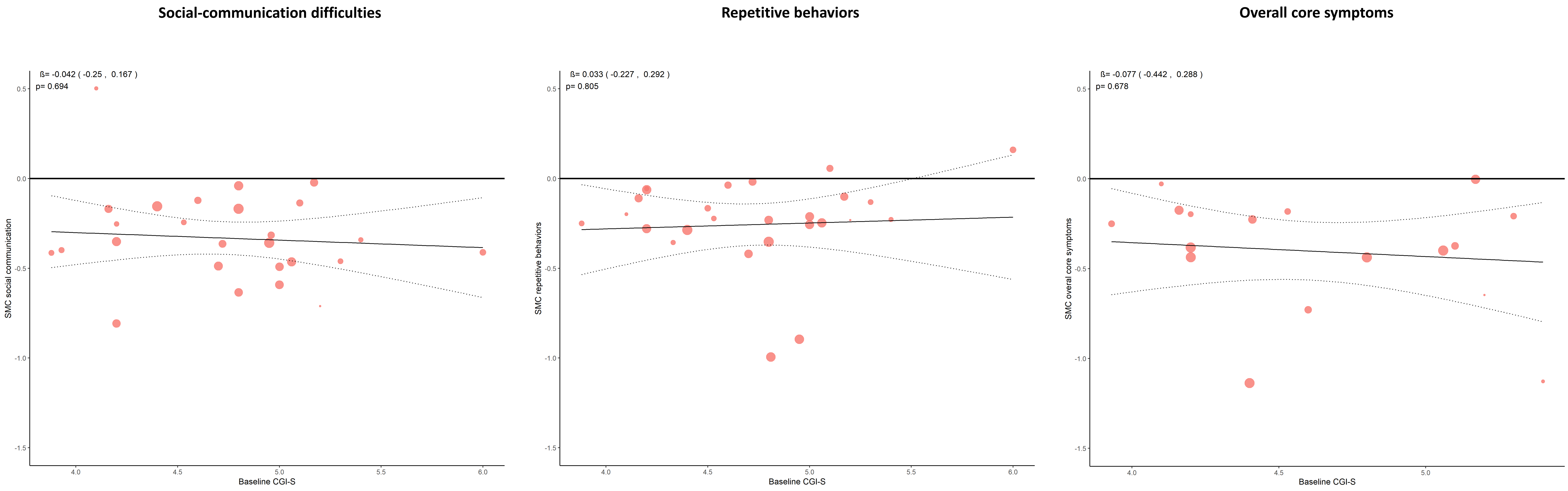

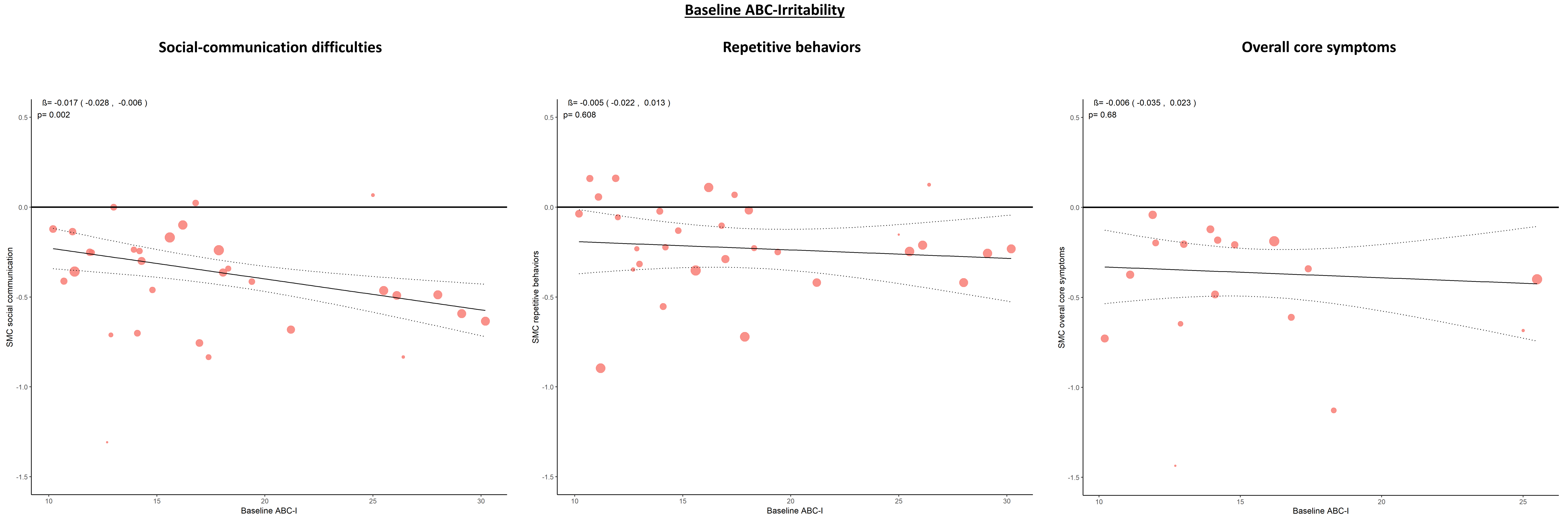

Baseline ABC-Irritability
Social-communication difficulties
ß= -0.017 (-0.028 , -0.006)
p= 0.002
SMC social communication
Baseline ABC-I
Repetitive behaviors
ß= -0.005 (-0.022 , 0.013)
p= 0.608
SMC repetitive behaviors
Baseline ABC-I
Overall core symptoms
ß= -0.006 (-0.035 , 0.023)
p= 0.68
SMC overal core symptoms
Baseline ABC-I

Minimum threshold of core symptoms at baseline

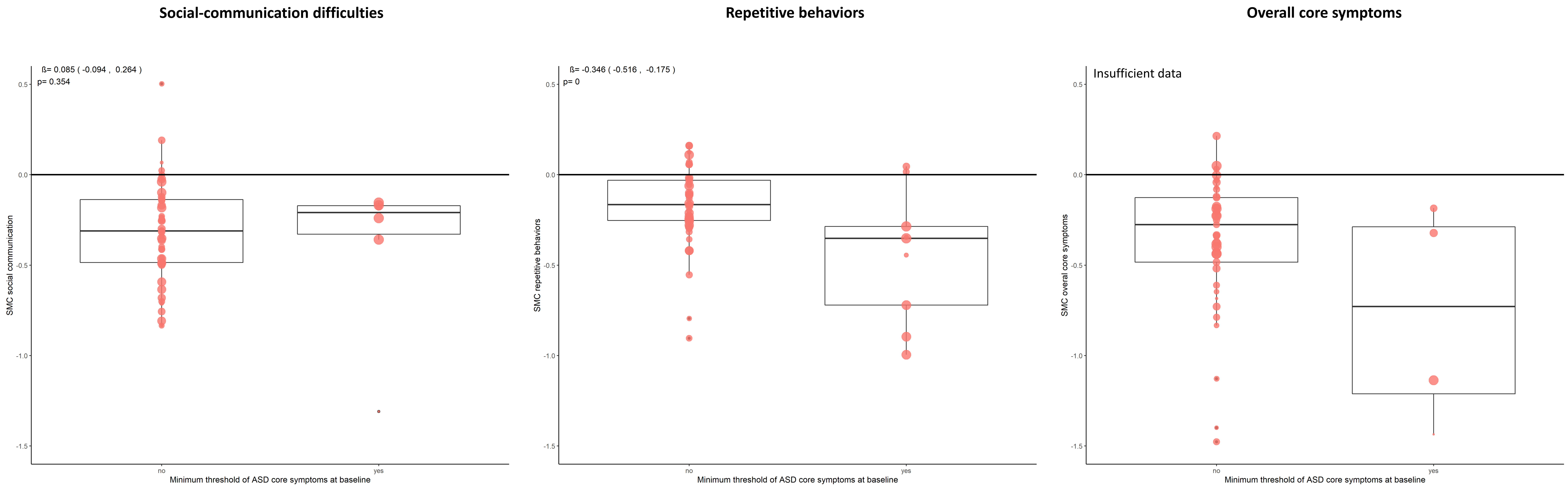

Supplement: Supplementary file 1 — Additional file 1. [file 13229_2020_372_MOESM1_ESM.pdf]
