# Supplementary material for: Placebo response in pharmacological and dietary supplement trials of autism spectrum disorder (ASD): systematic review and meta-regression analysis
Source: Mol Autism. 2020 Aug 26;11:66. doi: 10.1186/s13229-020-00372-z (PMC7448339; doi:10.1186/s13229-020-00372-z)
Supplement: Supplementary file 3 — Additional file 3. [file 13229_2020_372_MOESM3_ESM.docx]

**Appendix:**

**Placebo response in pharmacological and dietary supplement trials of autism spectrum disorder (ASD): systematic review and meta-regression analysis**

Spyridon Siafis,^1^ Oğulcan Çıray,^2^ Johannes Schneider-Thoma,^1^ Irene Bighelli,^1^ Marc Krause,^1^ Alessandro Rodolico,^3^ Anna Ceraso,^4^ Giacomo Deste,^4^ Maximilian Huhn,^1,5^ David Fraguas,^6,7^ Dimitris Mavridis,^8,9^ Tony Charman,^10^ Declan G Murphy,^11^ Mara Parellada,^6,7^ Celso Arango,^6,7^ Stefan Leucht^1^

^1^Department of Psychiatry and Psychotherapy, School of Medicine, Technical University of Munich, Munich, Germany; ^2^Dokuz Eylul University, School of Medicine, Department of Child and Adolescent Psychiatry Balçova, İzmir, Turkey; ^3^Department of Experimental and Clinical Medicine, Psychiatric Clinic University Hospital 'Gaspare Rodolico', University of Catania, Catania, Italy; ^4^Department of Psychiatry, Spedali Civili Hospital, Brescia, Italy; ^5^Department of Psychiatry, Psychosomatic Medicine and Psychotherapy Social Foundation Bamberg, Teaching Hospital of the University of Erlangen, Germany; ^6^Department of Child and Adolescent Psychiatry, Institute of Psychiatry and Mental Health, Hospital General Universitario Gregorio Marañón, Madrid, Spain; IiSGM, CIBERSAM, Spain; ^7^School of Medicine, Universidad Complutense, Madrid, Spain; ^8^Department of Primary Education, University of Ioannina, Ioannina, Greece; ^9^Faculté de Médecine, Université Paris Descartes, Paris, France; ^10^Department of Psychology, Institute of Psychiatry, Psychology & Neuroscience, King's College London, London, UK, ^11^Department of Forensic and Neurodevelopmental Sciences, Institute of Psychiatry, Psychology & Neuroscience, King's College London, London, UK

Corresponding author:

Spyridon Siafis,

Department of Psychiatry and Psychotherapy, School of Medicine, Technical University of Munich, Ismaningerstr. 22, 81675 Munich, Germany

Tel: +498941406415, Fax: +498941404888, e-mail: [spyridon.siafis@tum.de](mailto:spyridon.siafis@tum.de)

Contents:

[eAppendix-1 PRISMA checklist](#_eAppendix-1_PRISMA_checklist)

[eAppendix-2 Protocol](#_eAppendix-2_Protocol) and methods

[eAppendix-3 Database search strategy](#_eAppendix-3_Database_search)

[eAppendix-4 Study selection](#_eAppendix-4_Study_selection)

[eAppendix-5 Study characteristics](#_eAppendix-5_Study_characteristics)

[eAppendix-6 Results](#_eAppendix-6_Results)

# eAppendix-1 PRISMA checklist

1. PRISMA checklist 3

2. References 6

## PRISMA checklist

PISMA checklist according to Moher et al 2009.[1]

| **Section/topic** | **#** | **Checklist item** | **Reported on page #** |
| --- | --- | --- | --- |
| **TITLE** | | |  |
| Title | 1 | Identify the report as a systematic review, meta-analysis, or both. | 1 |
| **ABSTRACT** | | |  |
| Structured summary | 2 | Provide a structured summary including, as applicable: background; objectives; data sources; study eligibility criteria, participants, and interventions; study appraisal and synthesis methods; results; limitations; conclusions and implications of key findings; systematic review registration number. | 2 |
| **INTRODUCTION** | | |  |
| Rationale | 3 | Describe the rationale for the review in the context of what is already known. | 4 |
| Objectives | 4 | Provide an explicit statement of questions being addressed with reference to participants, interventions, comparisons, outcomes, and study design (PICOS). | 4 |
| **METHODS** | | |  |
| Protocol and registration | 5 | Indicate if a review protocol exists, if and where it can be accessed (e.g., Web address), and, if available, provide registration information including registration number. | 5, eAppendix-2.1. |
| Eligibility criteria | 6 | Specify study characteristics (e.g., PICOS, length of follow-up) and report characteristics (e.g., years considered, language, publication status) used as criteria for eligibility, giving rationale. | 5, eAppendix-2.2 |
| Information sources | 7 | Describe all information sources (e.g., databases with dates of coverage, contact with study authors to identify additional studies) in the search and date last searched. | 6, eAppendix-3 |
| Search | 8 | Present full electronic search strategy for at least one database, including any limits used, such that it could be repeated. | eAppendix-3 |
| Study selection | 9 | State the process for selecting studies (i.e., screening, eligibility, included in systematic review, and, if applicable, included in the meta-analysis). | 6 |
| Data collection process | 10 | Describe method of data extraction from reports (e.g., piloted forms, independently, in duplicate) and any processes for obtaining and confirming data from investigators. | 6 |
| Data items | 11 | List and define all variables for which data were sought (e.g., PICOS, funding sources) and any assumptions and simplifications made. | 5-7, eAppendix-2.2 |
| Risk of bias in individual studies | 12 | Describe methods used for assessing risk of bias of individual studies (including specification of whether this was done at the study or outcome level), and how this information is to be used in any data synthesis. | 5, 7-8 |
| Summary measures | 13 | State the principal summary measures (e.g., risk ratio, difference in means). | 7 |
| Synthesis of results | 14 | Describe the methods of handling data and combining results of studies, if done, including measures of consistency (e.g., I^2^) for each meta-analysis. | 7 |

| **Section/topic** | **#** | **Checklist item** | **Reported on page #** |
| --- | --- | --- | --- |
| Risk of bias across studies | 15 | Specify any assessment of risk of bias that may affect the cumulative evidence (e.g., publication bias, selective reporting within studies). | 5, 7-8 |
| Additional analyses | 16 | Describe methods of additional analyses (e.g., sensitivity or subgroup analyses, meta-regression), if done, indicating which were pre-specified. | 7-9 |
| **RESULTS** | | |  |
| Study selection | 17 | Give numbers of studies screened, assessed for eligibility, and included in the review, with reasons for exclusions at each stage, ideally with a flow diagram. | Figure 1, eAppendix-4 |
| Study characteristics | 18 | For each study, present characteristics for which data were extracted (e.g., study size, PICOS, follow-up period) and provide the citations. | Table S, eAppendix-4 |
| Risk of bias within studies | 19 | Present data on risk of bias of each study and, if available, any outcome level assessment (see item 12). | eAppendix-5.2 |
| Results of individual studies | 20 | For all outcomes considered (benefits or harms), present, for each study: (a) simple summary data for each intervention group (b) effect estimates and confidence intervals, ideally with a forest plot. | Figure 2- 5, eAppendix-6.4 |
| Synthesis of results | 21 | Present results of each meta-analysis done, including confidence intervals and measures of consistency. | 10-12, Figure 2-5, eAppendix-6.4 |
| Risk of bias across studies | 22 | Present results of any assessment of risk of bias across studies (see Item 15). | 11-13, eAppendix-5.2, eAppendix-6.2 |
| Additional analysis | 23 | Give results of additional analyses, if done (e.g., sensitivity or subgroup analyses, meta-regression [see Item 16]). | 11-14, eAppendix-6.1, 6.2 |
| **DISCUSSION** | | |  |
| Summary of evidence | 24 | Summarize the main findings including the strength of evidence for each main outcome; consider their relevance to key groups (e.g., healthcare providers, users, and policy makers). | 15-20 |
| Limitations | 25 | Discuss limitations at study and outcome level (e.g., risk of bias), and at review-level (e.g., incomplete retrieval of identified research, reporting bias). | 20-21 |
| Conclusions | 26 | Provide a general interpretation of the results in the context of other evidence, and implications for future research. | 21-22 |
| **FUNDING** | | |  |
| Funding | 27 | Describe sources of funding for the systematic review and other support (e.g., supply of data); role of funders for the systematic review. | 25 |

## References

1. Moher D, Liberati A, Tetzlaff J, Altman DG: **Preferred reporting items for systematic reviews and meta-analyses: the PRISMA statement**. *PLoS medicine* 2009, **6**(7):e1000097.

# eAppendix-2 Protocol and methods

1. PROSPERO protocol 8

2. Methods and post-hoc decisions 16

3. References 18

## 1. PROSPERO protocol

Placebo-controlled pharmacological and dietary supplement trials in autism spectrum disorders (ASD): systematic review, meta-analysis and meta-regression

Spyridon Siafis, Irene Bighelli, Johannes Schneider-Thoma, Stefan Leucht

Citation

Spyridon Siafis, Irene Bighelli, Johannes Schneider-Thoma, Stefan Leucht. Placebo-controlled pharmacological and dietary supplement trials in autism spectrum disorders (ASD): systematic review, meta-analysis and meta-regression. PROSPERO 2019 CRD42019125317 Available from: <https://www.crd.york.ac.uk/prospero/display_record.php?ID=CRD42019125317>

Review question

To investigate predictors of efficacy and placebo response in placebo-controlled pharmacological and dietary supplement trials in ASD.

Searches

Electronic databases: Comprehensive searches will be conducted in ClinicalTrials.gov, Cochrane Central Register of Controlled Trials (CENTRAL), EMBASE, MEDLINE, PsycINFO, PubMed, World Health Organization International Clinical Trials Registry Platform (WHO ICTRP). There will be no date/time, language, document type, and publication status limitations.

Reference searching: Reference lists of included records will be hand-searched for potentially relevant studies.

Previous reviews: Relevant reviews on pharmacological and dietary supplement treatments for ASD will be hand-searched for potentially relevant studies.

Personal contact: In addition, we will contact the first and/or corresponding author of each included study published in the last 30 years for missing information.

Search strategy <https://www.crd.york.ac.uk/PROSPEROFILES/125317_STRATEGY_20190213.pdf>

Types of study to be included

Randomized controlled trials (RCT) in which participants with ASD received pharmacological treatments or dietary supplements compared to placebo will be eligible.

Inclusion:

Both open and blinded RCTs.

Randomization will be implied if not explicitly reported, when the study is stated as double-blind.

In case of cross-over studies only data from the first phase before the crossover will be eligible, in order to avoid carry-over effects.

No restriction in terms of language or country of origin.

Exclusion:

Quasi-randomized trials and studies with high risk of bias in randomization as described in the Cochrane Handbook [1].

Cluster randomized trials.

Long-term studies with maintenance design, studies with placebo-controlled discontinuation or withdrawal design.

Studies published before 1980 (see participants/population).

Studies with less than 10 participants.

Condition or domain being studied

Autism spectrum disorders (ASD), including autistic disorder, Asperger’s syndrome and pervasive developmental disorder-not otherwise specified, as they were previously classified as independent categorical entities in DSM-IV.

Participants/population

Inclusion:

ASD as diagnosed by standardized diagnostic criteria (such as DSM-III, DSM-III-R, DSM-IV, DSM-IV-TR,DSM-5, ICD-10) and/or validated diagnostic tools.

Studies including participants with ASD and associated symptoms (e.g. ADHD symptoms, irritability) will be accepted.

Studies with all or some of the participants having a genetic syndrome (such as Fragile X syndrome) will be accepted, when all participants had also ASD (defined by inclusion criteria).

Trials in which less than 20% of participants had a developmental or psychiatric disorder other than ASD will be eligible.

There will be no restriction in terms of age, sex, ethnicity, setting, initial severity of ASD symptoms.

Exclusion:

Participants characterized as ‘autistic’ or with ‘autistic behavior’ and ‘autistic traits’ without using standardized diagnostic criteria or validated diagnostic tools for ASD. Studies published before 1980 will also be excluded, because ASD was clearly separated from childhood schizophrenia after the introduction of DSM-III (published in 1980). In addition, DSM-II and ICD-9 did not have standardized criteria.

Studies requiring all participants to have a genetic syndrome, but not all of the participants had ASD defined by inclusion criteria.

Studies focused on stable patients (controlled-discontinuation or withdrawal studies).

Intervention(s), exposure(s)

Any pharmacological treatment and dietary supplement will be eligible.

Inclusion:

Any application form or route of administration (e.g. oral, intramuscular, intravenous, intranasal) - Both fixed- and flexible-dose designs.

The minimum duration of treatment will be seven days.

Exclusion:

Other interventions, such as psychological/behavioral, traditional medicine, homeopathic, dietary interventions, such as elimination diets (gluten/casein-free, ketogenic diets) or milk formulations.

Augmentation treatments.

Studies using single doses.

Comparator(s)/control

Placebo.

Context

Main outcome(s)

Overall ASD core symptoms.

Social communication/interaction deficits.

Repetitive behaviors/restricted interests.

Timing and effect measures

Published and validated scales will be used. When scales filled by multiple informants are available, we will use the hierarchy: clinicians’, parents/caregivers’ and teachers’ rating. Regarding core symptoms, separate analyses will be conducted for rating scales filled by parent/caregivers, teachers and clinicians as secondary outcomes. Change scores will be preferred, but we will also use endpoint scores if the former are not available.

We will take the endpoint results and pool all studies. In addition, studies will be classified as shorter-term (1-12 weeks) and longer-term (13 or more weeks).

Additional outcome(s)

CGI-improvement and CGI-Severity.

Overall ASD core symptoms as measured by rating scales filled by clinicians, caregivers or teachers.

Social communication/interaction deficits as measured by rating scales filled by clinicians, caregivers or teachers.

Repetitive behaviors/restricted interests as measured by rating scales filled by clinicians, caregivers or teachers.

Internalizing associated symptoms (such as anxiety) as measured by appropriate scales.

Externalizing associated symptoms as well as ADHD symptoms and irritability, as measured by appropriate scales.

Number of participants with response to treatment, any study definition is eligible.

Quality of life of participants as measured by appropriate scales (e.g. PedsQL)

Global functioning of participants as measured by appropriate scales (e.g. CGAS)

Parental stress as measured by appropriate scales (e.g. PSI)

Number of participants who prematurely discontinued due to any cause (as a measure of overall acceptability), inefficacy (as a measure of global efficacy), adverse events (as a measure of overall tolerability).

Important side effects.

Timing and effect measures

We will take the endpoint results and pool all studies. In addition, studies will be classified as shorter-term (1-12 weeks) and longer-term (13 or more weeks).

Data extraction (selection and coding)

Selection of trials: At least two reviewers will independently inspect the titles and abstracts of non-duplicated references identified through the search and will exclude those not pertinent. Discrepancies between the two reviewers will be resolved by discussion reaching consensus. If doubts still remain, the full-text will be obtained and eligibility will be assessed. Full-texts of included references will be obtained and independently assessed by two reviewers for eligibility. Again, disagreements will be resolved by discussion and, if needed, a third senior author will be involved. When required, further information will be requested from study authors.

Data extraction: Two authors will independently extract data from all selected trials in a Microsoft Access database. When disagreement arises we will resolve it by discussion and, if needed, involving a third senior author. Where this is not sufficient we will contact the study authors.

Data extraction will include:

Study citation, registration number to trials registries, year of publication, location, setting, number of centers, sample size, and funding/sponsor (industry or academic).

Methodology (study design, number of arms and risk of bias).

Characteristics of study participants (age, sex, IQ, diagnosis, sample size).

Characteristics of intervention (name, dose, application form).

Outcome measures, including information on whether an intention-to-treat approach has been used and how it was defined.

Risk of bias (quality) assessment

Two independent review authors will assess the risk of bias in the selected studies using the Cochrane Collaboration ‘risk of bias’ tool. When disagreement arises we will resolve it by discussion and, if needed, involving a third senior author. The following domains will be considered (classified as low, moderate or high): sequence generation, allocation concealment, blinding, completeness of outcome data, selective reporting and other biases. Similar to the GRISELDA NMA [2, 3] studies will be classified as having an overall low (no domain with high risk of bias and three or less with unclear risk), moderate (one with high risk of bias or none with high risk of bias but four or more with unclear risk) and high risk of bias (all other cases). Quality of evidence for the primary outcome will be assessed by GRADE approach [4].

Strategy for data synthesis

The effect size for continuous outcomes will be the standardized mean difference as Hedges’ g, and for dichotomous outcomes the relative risk, accompanied by their 95% confidence intervals. Intention-to-treat data will be used whenever possible. For dichotomous outcomes, if the original authors presented only the results of completer population, we will assume that those participants lost to follow-up would not have changed for a given outcome. Missing standard deviations (SD) will be calculated from the following options and following order by the 1) standard error, 2) CIs, t-value or p-value, 3) contacting original authors 4) by SDs from other studies using a validated imputation method as described in the Cochrane Handbook.

Pairwise meta-analyses of studies, separately for children/adolescents and adults, that compared the same intervention with placebo will be conducted using random effects models [5]. Meta-regressions of predictors of efficacy and placebo response will be conducted by using all studies. Meta-regressions for individual interventions will not be conducted due to small statistical power.

Heterogeneity will be investigated by visual inspection of the forest plots, χ² test of homogeneity and I².

We will attempt to include unpublished studies. Small study and publication bias will be explored with funnel plot analyses if at least 10 studies are available for a comparison.

Sensitivity analyses of the primary outcomes will be performed:

Exclusion of studies with implied randomization.

Exclusion of studies including participants with associated symptoms or genetic syndrome.

Exclusion of studies with a diagnosis based only on diagnostic evaluation tools.

Exclusion of open and single-blinded trials.

Exclusion of studies lasting less than 4 weeks.

Using a fixed effects model.

Exclusion of studies presenting only completers data.

Exclusion of studies with imputed missing SD.

Exclusion of studies with an overall high or unclear risk of bias.

Analysis of subgroups or subsets

Meta-regressions of predictors of efficacy and placebo response will be conducted for the primary outcomes using a similar approach to our previous meta-regressions in acute schizophrenia [6-8]. The dependent variables in the analyses will be 1) placebo response, 2) drug response and 3) effect sizes for the comparisons of interventions with placebo. The independent variables will be the following potential moderators. A priori, we plan exploratory univariate meta-regressions. Multivariable meta-regression models will be conducted, if there are enough available data, because a higher statistical power is required.

Potential moderators will be assessed:

1. Drug-related factors:

Route of administration (oral versus other).

Type of intervention: pharmacological versus dietary supplements.

Fixed versus flexible designs.

2. Design-related factors:

Study duration (in weeks).

Target symptom of the study: associated symptoms versus not.

Publication year.

Duration of wash-out (in days).

Use of placebo-lead in phase with exclusion of placebo responders.

Type of informant: parent/caregiver versus clinician or teacher.

Sample size.

Number of sites and proportion of academic sites.

Number of arms and medications.

Percentage of participants on placebo.

Sponsorship (at least one site industry funded, no donation alone) versus not industry funded.

Risk of bias for each domain.

3. Participant-related factors:

a. Degree of placebo response (when efficacy or drug response are the dependent variables) b. Mean age.

US population versus not US or mixed populations.

Baseline severity

Intellectual impairment

Contact details for further information

Stefan Leucht

stefan.leucht@tum.de

Organisational affiliation of the review

Department of Psychiatry and Psychotherapy, Technische Universität München, Klinikum rechts der Isar <http://www.cfdm.de/>

Review team members and their organisational affiliations

Mr Spyridon Siafis. Department of Psychiatry and Psychotherapy, Technische Universität München, Klinikum rechts der Isar

Dr Irene Bighelli. Department of Psychiatry and Psychotherapy, Technische Universität München, Klinikum rechts der Isar

Dr Johannes Schneider-Thoma. Department of Psychiatry and Psychotherapy, Technische Universität München, Klinikum rechts der Isar

Professor Stefan Leucht. Department of Psychiatry and Psychotherapy, Technische Universität München, Klinikum rechts der Isar

Collaborators

Professor Celso Arango. Department of Child and Adolescent Psychiatry, Hospital General Universitario Gregorio Marañón, Madrid, Spain; IiSGM, CIBERSAM, Spain; School of Medicine, Universidad Complutense, Madrid, Spain.

Professor Mara Parellada. Department of Child and Adolescent Psychiatry, Hospital General Universitario

Gregorio Marañón, Madrid, Spain; IiSGM, CIBERSAM, Spain; School of Medicine, Universidad

Complutense, Madrid, Spain

Mr Farhad Shokraneh. Cochrane Schizophrenia Group, The Institute of Mental Health, the University of Nottingham and Nottinghamshire Healthcare NHS Trust, Nottingham, UK

Type and method of review

Intervention, Meta-analysis, Systematic review

Anticipated or actual start date

01 June 2018

Anticipated completion date

31 May 2020

Funding sources/sponsors

This project has received funding from the Innovative Medicines Initiative 2 Joint Undertaking under grant agreement No 777394. This Joint Undertaking receives support from the European Union’s Horizon 2020 research and innovation programme and EFPIA and AUTISM SPEAKS, Autistica, SFARI

Conflicts of interest

In the last 3 years, Stefan Leucht has received honoraria for consulting or lectures from LB Pharma, Lundbeck, Otsuka, TEVA, LTS Lohmann, Geodon Richter, Recordati, Boehringer Ingelheim, Sandoz, Janssen, Lilly, SanofiAventis, Servier and Sunovion.

David Fraguas has been a consultant and/or has received fees from Angelini, Eisai, IE4Lab, Janssen, Lundbeck, and Otsuka. He has also received grant support from Instituto de Salud Carlos III (Spanish Ministry of Science, Innovation and Universities) and from Fundación Alicia Koplowitz.

Mara Parellada has received educational honoraria from Otsuka, research grants from FAK and Fundación

Mutua Madrileña (FMM), Instituto de Salud Carlos III (Spanish Ministry of Science, Innovation and

Universities) and European ERANET and H2020 calls, travel grants from Otsuka and Janssen. Consultant for Exeltis and Servier.

Yes

Language

English

Country

Germany

Stage of review

Review Ongoing

Subject index terms status

Subject indexing assigned by CRD

Subject index terms

Autism Spectrum Disorder; Dietary Supplements; Humans

Date of registration in PROSPERO

15 March 2019

Date of publication of this version

15 March 2019

## 2. Methods and post-hoc decisions

- Augmentation treatments: Combination treatments, e.g. placebo + antipsychotic, placebo + psychological/behavioral treatments or experimental intervention + antipsychotic/psychological/behavioral intervention treatment, were excluded.
- High risk of bias in randomization: We excluded studies with a high risk of bias in randomization according to the Cochrane Handbook [1, 9]. Sequence generation and allocation concealment are both components of the randomization process and the associated selection bias [10]. Therefore, we excluded studies with a high risk of bias in sequence generation or allocation concealment according to the risk of bias tool [9].
- About descriptive characteristics (e.g. age, sex, intellectual disability, ethnicity, BMI), we preferred as randomized data per arm, but we also used completers data per arm, or data from the total sample.
- Responder rates: In this analysis, we supplemented the investigation of placebo response as continuous outcome with responder rates. Responder rates are easily interpretable in comparison to standardized mean changes of continuous outcomes. We used response as defined by at least much improvement in the CGI-I (CGI-I=1 or 2), which is a rather homogenous definition (despite the use of different anchors) and it was frequently used in clinical trials of autism [11, 12]. We also imputed the number of responders from mean and standard deviations of CGI-I, when they were not reported, using and validated method [13, 14]. In the imputation, a threshold of 2.4 was used instead of 2 to impute responders from an assumed normal distribution. CGI-I is a categorical scale with 7-points, and therefore we assumed that a participant with a value of 2.4 or less in the assumed underlying normal distribution would have been considered as at least much improved. Post-hoc, sensitivity analysis of exclusion of studies with imputed responders were conducted according to the suggestion of Samara et al 2013 [14].
- Mean and standard deviations: When median and ranges were reported, we estimated the mean and standard deviations as reported in Hozo et al 2005 [15]. When subscales rather than the eligible total scores were reported, we pooled mean and standard deviations of the subscales assuming a correlation of 0.5 [16]. In a predefined sensitivity analysis of excluded studies with imputed standard deviation [1, 17], we also excluded studies with estimated mean and standard deviation from medians/ranges and lumping subscales.
- Effect sizes: In the protocol, the effect sizes were Hedge’s g for the continuous outcomes and relative risk for dichotomous outcomes. In this analysis, we conducted single group meta-analyses, therefore we did not compare experimental and control investigations. For the continuous outcomes, we used standardized mean change with raw score standardization using the baseline standard deviation, which is a standardized measure and its point estimate is not affected by the pre-post correlation, which is rarely reported [16]. A similar measure has been used in a similar analysis of placebo response in schizophrenia [18]. For the dichotomous outcome, we conducted a meta-analysis of proportions, pooling the logit transformed response rates, which were back-transformed for presentations [19].
- Longer-term studies: Longer-term studies were classified when they had a duration of 12 weeks or more, rather than 13 weeks or more. Some studies had three-month duration (13 weeks) but the exact duration in weeks were inconsistently reported.
- Post-hoc sensitivity analyses: We added post-hoc sensitivity analyses of the primary outcomes using different pre-post correlations for the calculation of the variance of the standardized mean change. In the primary analysis, a common pre-post correlation 0.5 was used, which was the median of questionnaire/score outcomes in a meta-epidemiological study [20]. Sensitivity analysis using 0.25 (median of studies in psychiatry was 0.36) and 0.75 (median of inactive or no treatment arms was 0.73) were conducted [20].
- Baseline severity was inconsistently assessed and reported in trials. CGI-S and ABC-Irritability were used as measures of global severity and serious problem behaviors, as well as they were used in other meta-analyses as measures of baseline severity [21-23]. In addition, the use of a minimum threshold of core symptoms for inclusion (any domain, but not used only for diagnostic confirmation) was considered, as in other analyses [6-8].
- About blinding, we used an aggregate covariate using the highest risk of bias between blinding of participants/personnel and blinding of outcome assessors (the risk of bias between these two domains was very similar, see eAppendix-5.2).
- Post-hoc, we conducted Spearman’s ρ correlations in order to assess bivariate correlations between covariates as well as between SMCs of placebo and experimental intervention. SMC for experimental intervention was calculated similarly to placebo. In case of multi-arm studies, a weighted mean SMC was calculated according to the Cochrane Handbook. Arms with inappropriate to our review interventions were excluded such as psychological/behavioral or multimodal interventions (e.g. behavioral intervention + atomoxetine, or behavioral intervention + placebo).
- Post-hoc covariates in the meta-regressions:
  - The minimum duration of washout period in days would have been included as a covariate. However, due to the inconsistent reporting and definition of washout from psychotropic drugs, a categorical covariate of the presence or not of a washout period was used.
  - Studies in which the authors applied for a patent on the experimental intervention were categorized with “industry-sponsored” studies.
  - According to a secondary analysis of a trial [23], we supplemented the list of covariates with sex (% female participants), baseline mean BMI, ethnicity (% of Caucasian or Hispanic).

## 3. References

1. Higgins JP, Green S: **Cochrane handbook for systematic reviews of interventions**, vol. 4: John Wiley & Sons; 2011.

2. Cipriani A, Furukawa TA, Salanti G, Chaimani A, Atkinson LZ, Ogawa Y, Leucht S, Ruhe HG, Turner EH, Higgins JPT *et al*: **Comparative efficacy and acceptability of 21 antidepressant drugs for the acute treatment of adults with major depressive disorder: a systematic review and network meta-analysis**. *Lancet* 2018, **391**(10128):1357-1366.

3. Furukawa TA, Salanti G, Atkinson LZ, Leucht S, Ruhe HG, Turner EH, Chaimani A, Ogawa Y, Takeshima N, Hayasaka Y *et al*: **Comparative efficacy and acceptability of first-generation and second-generation antidepressants in the acute treatment of major depression: protocol for a network meta-analysis**. *BMJ Open* 2016, **6**(7):e010919.

4. Guyatt G, Oxman AD, Akl EA, Kunz R, Vist G, Brozek J, Norris S, Falck-Ytter Y, Glasziou P, DeBeer H *et al*: **GRADE guidelines: 1. Introduction-GRADE evidence profiles and summary of findings tables**. *Journal of clinical epidemiology* 2011, **64**(4):383-394.

5. DerSimonian R, Laird N: **Meta-analysis in clinical trials**. *Controlled clinical trials* 1986, **7**(3):177-188.

6. Leucht S, Chaimani A, Leucht C, Huhn M, Mavridis D, Helfer B, Samara M, Cipriani A, Geddes JR, Salanti G *et al*: **60years of placebo-controlled antipsychotic drug trials in acute schizophrenia: Meta-regression of predictors of placebo response**. *Schizophrenia research* 2018, **201**:315-323.

7. Leucht S, Chaimani A, Mavridis D, Leucht C, Huhn M, Helfer B, Samara M, Cipriani A, Geddes JR, Davis JM: **Disconnection of drug-response and placebo-response in acute-phase antipsychotic drug trials on schizophrenia? Meta-regression analysis**. *Neuropsychopharmacology* 2019.

8. Leucht S, Leucht C, Huhn M, Chaimani A, Mavridis D, Helfer B, Samara M, Rabaioli M, Bacher S, Cipriani A *et al*: **Sixty Years of Placebo-Controlled Antipsychotic Drug Trials in Acute Schizophrenia: Systematic Review, Bayesian Meta-Analysis, and Meta-Regression of Efficacy Predictors**. *Am J Psychiatry* 2017, **174**(10):927-942.

9. Higgins JP, Altman DG, Gotzsche PC, Juni P, Moher D, Oxman AD, Savovic J, Schulz KF, Weeks L, Sterne JA: **The Cochrane Collaboration's tool for assessing risk of bias in randomised trials**. *BMJ (Clinical research ed)* 2011, **343**:d5928.

10. Odgaard-Jensen J, Vist GE, Timmer A, Kunz R, Akl EA, Schunemann H, Briel M, Nordmann AJ, Pregno S, Oxman AD: **Randomisation to protect against selection bias in healthcare trials**. *The Cochrane database of systematic reviews* 2011(4):Mr000012.

11. Aman MG, Novotny S, Samango-Sprouse C, Lecavalier L, Leonard E, Gadow KD, King BH, Pearson DA, Gernsbacher MA, Chez M: **Outcome measures for clinical drug trials in autism**. *CNS Spectr* 2004, **9**(1):36-47.

12. Scahill L, Aman MG, Lecavalier L, Halladay AK, Bishop SL, Bodfish JW, Grondhuis S, Jones N, Horrigan JP, Cook EH *et al*: **Measuring repetitive behaviors as a treatment endpoint in youth with autism spectrum disorder**. *Autism* 2013, **19**(1):38-52.

13. Furukawa TA, Cipriani A, Barbui C, Brambilla P, Watanabe N: **Imputing response rates from means and standard deviations in meta-analyses**. *International clinical psychopharmacology* 2005, **20**(1):49-52.

14. Samara MT, Spineli LM, Furukawa TA, Engel RR, Davis JM, Salanti G, Leucht S: **Imputation of response rates from means and standard deviations in schizophrenia**. *Schizophrenia research* 2013, **151**(1-3):209-214.

15. Hozo SP, Djulbegovic B, Hozo I: **Estimating the mean and variance from the median, range, and the size of a sample**. *BMC Medical Research Methodology* 2005, **5**(1):13.

16. Morris SB, DeShon RP: **Combining effect size estimates in meta-analysis with repeated measures and independent-groups designs**. *Psychological methods* 2002, **7**(1):105-125.

17. Furukawa TA, Barbui C, Cipriani A, Brambilla P, Watanabe N: **Imputing missing standard deviations in meta-analyses can provide accurate results**. *Journal of clinical epidemiology* 2006, **59**(1):7-10.

18. Agid O, Siu CO, Potkin SG, Kapur S, Watsky E, Vanderburg D, Zipursky RB, Remington G: **Meta-regression analysis of placebo response in antipsychotic trials, 1970-2010**. *Am J Psychiatry* 2013, **170**(11):1335-1344.

19. Schwarzer G, Chemaitelly H, Abu-Raddad LJ, Rücker G: **Seriously misleading results using inverse of Freeman-Tukey double arcsine transformation in meta-analysis of single proportions**. *Research Synthesis Methods* 2019, **0**(0).

20. Balk EM, Earley A, Patel K, Trikalinos TA, Dahabreh IJ: **Empirical assessment of within-arm correlation imputation in trials of continuous outcomes**. 2012.

21. Masi A, Lampit A, DeMayo MM, Glozier N, Hickie IB, Guastella AJ: **A comprehensive systematic review and meta-analysis of pharmacological and dietary supplement interventions in paediatric autism: moderators of treatment response and recommendations for future research**. *Psychological medicine* 2017, **47**(7):1323-1334.

22. Masi A, Lampit A, Glozier N, Hickie IB, Guastella AJ: **Predictors of placebo response in pharmacological and dietary supplement treatment trials in pediatric autism spectrum disorder: a meta-analysis**. *Translational psychiatry* 2015, **5**:e640.

23. King BH, Dukes K, Donnelly CL, Sikich L, McCracken JT, Scahill L, Holl, er E, Bregman JD, Anagnostou E *et al*: **Baseline factors predicting placebo response to treatment in children and adolescents with autism spectrum disorders: a multisite randomized clinical trial**. *JAMA pediatrics* 2013, **167**(11):1045–1052.

# eAppendix-3 Database search strategy

1. Electronic search 21

1.1 Search on 8^th^ July 2018 21

1.2 PubMed update search on 4^th^ July 2019 21

2. Search Strategies 22

2.1 ClinicalTrials.Gov 22

2.2 Cochrane Central Register of Controlled Trials (CENTRAL) 22

2.3 EMBASE 25

2.4 MEDLINE 29

2.5 PsycINFO 34

2.6 PubMed 36

2.7 World Health Organization International Clinical Trials Registry Platform (WHO ICTRP) 42

3. References 44

## 1. Electronic search

### 1.1 Search on 8^th^ July 2018

We searched the following resources on 8^th^ July 2018 with no date/time, language, document type, and publication status limitations:

- ClinicalTrials.Gov (Until search date)
- Cochrane Central Register of Controlled Trials (CENTRAL) (Until search date)
- EMBASE (1974 to 2018 week 28)
- MEDLINE (1946 – search date)
- PsycINFO (1806 to July week 1 2018)
- PubMed (1946 – search date)
- World Health Organization International Clinical Trials Registry Platform (WHO ICTRP) (Until search date)

We followed the Cochrane Handbook [1] and MECIR [2] for conducting the search, PRISMA guideline [3] for reporting the search, and PRESS checklist for peer-reviewing the search strategies [4]. Keywords and search concepts were collected through experts’ opinion, existing recent survey[5], controlled vocabulary (APA Thesaurus, Medical Subject Headings = MeSH, and Excerpta Medica Tree = EMTREE), text mining using the Yale MeSH Analyzer [6], and reviewing the primary search results. In addition, we utilized the report from Canadian Agency for Drugs and Technologies in Health (CADTH) to focus the search employing EMTREE [7]. We used existing search filters such as Eisinga’s animal search filter to remove the non-human search results in EMBASE [8] and Cochrane’s Randomized Controlled Trials search filters for EMBASE, MEDLINE, and PubMed [1]. Because of poor reporting of outcomes in medical research [9-13], we did not limit the search adding specific outcomes so that we could have all the outcomes. Search strategies, developed by assistance of a medical information specialist, were reported in eAppendix-3.2. The duplicate search results were detected based on title match and deleted after approval through manual check in EndNote X8.

The search found 13,803 references. Removing 6,455 duplicate records, we screened 7,348 records.

### 1.2 PubMed update search on 4^th^ July 2019

An update search on PubMed was conducted on 4^th^ July 2019. The search found 191 new records, which were screened.

## 2. Search Strategies

### 2.1 ClinicalTrials.Gov

Advanced Search

Condition or Disease: Autism Spectrum Disorder OR Autistic Disorder OR Asperger Syndrome OR Rett Syndrome OR "Child Development Disorders, Pervasive" OR Childhood Disintegrative Disorder OR Hyperammonemia

Study Type: Interventional Studies (Clinical Trials)

### 2.2 Cochrane Central Register of Controlled Trials (CENTRAL)

([mh "Autistic Disorder"] OR [mh "Autism Spectrum Disorder"] OR [mh "Asperger Syndrome"] OR [mh "Rett Syndrome"] OR [mh "Child Development Disorders, Pervasive"] OR (Autis* OR Kanner* OR Asperger* OR "Pervasive Child Development Disorder" OR "Pervasive Child Development Disorders" OR "Pervasive Developmental Disorder" OR "Pervasive Developmental Disorders" OR "Pervasive Development Disorder" OR "Pervasive Development Disorders" OR "Childhood Disintegrative Disorder" OR "Childhood Disintegrative Disorders" OR Rett* OR "Cerebroatrophic Hyperammonemia" OR "Cerebroatrophic Hyperammonemias"):ti,ab) AND ([mh ^"Pharmaceutical Preparations"] OR [mh "Drug Combinations"] OR [mh "Drugs, Chinese Herbal"] OR [mh "Drugs, Essential"] OR [mh "Drugs, Generic"] OR [mh "Drugs, Investigational"] OR [mh "Nonprescription Drugs"] OR [mh "Plant Extracts"] OR [mh "Prescription Drugs"] OR [mh Prodrugs] OR [mh "Pharmacologic Actions"] OR [mh "Drug Therapy"] OR [mh "Therapeutic Uses"] OR [mh "Physiological Effects of Drugs"] OR [mh Aripiprazole] OR [mh Clozapine] OR [mh Haloperidol] OR [mh Loxapine] OR [mh "Lurasidone Hydrochloride"] OR [mh "Paliperidone Palmitate"] OR [mh "Quetiapine Fumarate"] OR [mh Risperidone] OR [mh Sulpiride] OR [mh Citalopram] OR [mh Clomipramine] OR [mh Desipramine] OR [mh Fluoxetine] OR [mh Fluvoxamine] OR [mh Imipramine] OR [mh Mianserin] OR [mh Nortriptyline] OR [mh Paroxetine] OR [mh Sertraline] OR [mh "Venlafaxine Hydrochloride"] OR [mh "Atomoxetine Hydrochloride"] OR [mh Dextromethorphan] OR [mh Fenfluramine] OR [mh "Lisdexamfetamine Dimesylate"] OR [mh "Lithium Carbonate"] OR [mh Methylphenidate] OR [mh "N-Methyl-3,4-methylenedioxyamphetamine"] OR [mh "Valproic Acid"] OR [mh Betahistine] OR [mh Bromocriptine] OR [mh Buspirone] OR [mh Cyproheptadine] OR [mh Famotidine] OR [mh Levodopa] OR [mh Sumatriptan] OR [mh Clonidine] OR [mh Guanfacine] OR [mh Dexmedetomidine] OR [mh Propranolol] OR [mh Acetylcysteine] OR [mh Amantadine] OR [mh Ketamine] OR [mh Memantine] OR [mh Riluzole] OR [mh Baclofen] OR [mh Bumetanide] OR [mh Flumazenil] OR [mh Galantamine] OR [mh Mecamylamine] OR [mh Pregnenolone] OR [mh Rivastigmine] OR [mh Varenicline] OR [mh Cannabidiol] OR [mh Celecoxib] OR [mh Everolimus] OR [mh "Fingolimod Hydrochloride"] OR [mh Fluconazole] OR [mh "Glatiramer Acetate"] OR ([mh Immunoglobulins] AND [mh "Administration, Oral"]) OR [mh Minocycline] OR [mh Naltrexone] OR [mh Pentoxifylline] OR [mh Sirolimus] OR [mh Staurosporine] OR [mh Suramin] OR [mh Tacrolimus] OR [mh "Insulin-Like Growth Factor I"] OR [mh "Adrenal Cortex Hormones"] OR [mh "Adrenocorticotropic Hormone"] OR [mh Angiotensins] OR [mh Carnitine] OR [mh "Diet Therapy"] OR [mh "Dietary Supplements"] OR [mh "Fatty Acids, Omega-3"] OR [mh "Gastrin-Releasing Peptide"] OR [mh Ghrelin] OR [mh Hydrocortisone] OR [mh Lovastatin] OR [mh Melanocortins] OR [mh Melatonin] OR [mh Metformin] OR [mh Minerals] OR [mh "Nutrition Therapy"] OR [mh Oligosaccharides] OR [mh Oxytocin] OR [mh Prednisone] OR [mh Probiotics] OR [mh Pyridoxine] OR [mh Secretin] OR [mh "Thyroid Hormones"] OR [mh Thyroxine] OR [mh Triiodothyronine] OR [mh Vasopressins] OR [mh "Vitamin E"] OR [mh "Arachidonic Acid"] OR [mh "Ascorbic Acid"] OR [mh Carnosine] OR [mh "Docosahexaenoic Acids"] OR [mh "Folic Acid"] OR [mh "Ginkgo biloba"] OR [mh Glutathione] OR [mh Glutens] OR [mh Inositol] OR [mh Leucovorin] OR [mh Magnesium] OR [mh "Magnesium Oxide"] OR [mh Milk] OR [mh Papain] OR [mh Succimer] OR [mh "Vitamin B 12"] OR [mh "Vitamin B 6"] OR [mh "Vitamin D"] OR [mh "Adrenergic alpha-2 Receptor Antagonists"] OR [mh "Antidepressive Agents, Second-Generation"] OR [mh "Anti-Dyskinesia Agents"] OR [mh Antiemetics] OR [mh "Antipsychotic Agents"] OR [mh "Dopamine Agonists"] OR [mh "Dopamine Antagonists"] OR [mh "Dopamine D2 Receptor Antagonists"] OR [mh "GABA Antagonists"] OR [mh "Serotonin 5-HT2 Receptor Antagonists"] OR [mh "Serotonin Agents"] OR [mh "Serotonin Antagonists"] OR [mh "Serotonin Uptake Inhibitors"] OR [mh "Adrenergic alpha-Antagonists"] OR [mh "Adrenergic Uptake Inhibitors"] OR [mh "Cytochrome P-450 CYP1A2 Inhibitors"] OR [mh "Cytochrome P-450 CYP2C19 Inhibitors"] OR [mh "Cytochrome P-450 CYP2D6 Inhibitors"] OR [mh "Enzyme Inhibitors"] OR [mh "Histamine H1 Antagonists"] OR [mh "Serotonin and Noradrenaline Reuptake Inhibitors"] OR [mh "Anti-Anxiety Agents"] OR [mh "Antidepressive Agents"] OR [mh "Antidepressive Agents, Tricyclic"] OR [mh "Psychotropic Drugs"] OR [mh Anticonvulsants] OR [mh "Antimanic Agents"] OR [mh "Calcium Channel Blockers"] OR [mh "Central Nervous System Stimulants"] OR [mh "Cytochrome P-450 CYP3A Inducers"] OR [mh "Dopamine Uptake Inhibitors"] OR [mh "Excitatory Amino Acid Antagonists"] OR [mh "GABA Agents"] OR [mh Hallucinogens] OR [mh "Neuroprotective Agents"] OR [mh "Nootropic Agents"] OR [mh "Serotonin Receptor Agonists"] OR [mh "Voltage-Gated Sodium Channel Blockers"] OR [mh "Antiparkinson Agents"] OR [mh "Dopamine Agents"] OR [mh "Histamine Agonists"] OR [mh "Histamine H2 Antagonists"] OR [mh "Hormone Antagonists"] OR [mh "Serotonin 5-HT1 Receptor Agonists"] OR [mh "Adrenergic alpha-2 Receptor Agonists"] OR [mh "Adrenergic beta-Antagonists"] OR [mh Sympatholytics] OR [mh "Excitatory Amino Acid Agonists"] OR [mh "Cholinesterase Inhibitors"] OR [mh "GABA Modulators"] OR [mh "GABA-B Receptor Agonists"] OR [mh "Ganglionic Blockers"] OR [mh "Nicotinic Agonists"] OR [mh "Nicotinic Antagonists"] OR [mh Parasympathomimetics] OR [mh "Sodium Potassium Chloride Symporter Inhibitors"] OR [mh "14-alpha Demethylase Inhibitors"] OR [mh "Adjuvants, Immunologic"] OR [mh "Anti-Bacterial Agents"] OR [mh "Antifungal Agents"] OR [mh "Cannabinoid Receptor Agonists"] OR [mh "Cannabinoid Receptor Antagonists"] OR [mh "Cannabinoid Receptor Modulators"] OR [mh "Central Nervous System Agents"] OR [mh "Cyclooxygenase 2 Inhibitors"] OR [mh "Cytochrome P-450 CYP2C9 Inhibitors"] OR [mh "Immunologic Factors"] OR [mh "Immunosuppressive Agents"] OR [mh "Narcotic Antagonists"] OR [mh "Neurotransmitter Agents"] OR [mh "Purinergic Agents"] OR [mh "Anti-Inflammatory Agents"] OR [mh Antioxidants] OR [mh "Central Nervous System Depressants"] OR [mh "Chelating Agents"] OR [mh Hormones] OR [mh Oxytocics] OR [mh "Vitamin B Complex"] OR [mh Vitamins] OR (Anticonvuls* OR Antiepilep* OR Antipsychotic* OR Psychotropic* OR "Anti-Anxiety" OR Anxiolytic* OR Antidepress* OR "Pharmaco-Therapy" OR "Pharmaco-Therapies" OR Chemotherapy OR Chemotherapies OR Pharmacotherapy OR Pharmacotherapies OR "Pharmacological Interventions" OR "Pharmacological Intervention" OR "Pharmacological Treatment" OR "Pharmacological Treatments" OR "Drug Therapy" OR "Drug Therapies" OR Amisulpride OR Aripiprazol* OR Abilify OR Brexpiprazole OR Clozapine OR Clozaril OR Leponex OR Haloperidol OR Haldol OR Loxapine OR Lurasidone OR Latuda OR Olanzapine OR Zyprexa OR Paliperidone OR Invega OR Quetiapine OR Seroquel OR Risperidone OR Risperdal OR Risperidal OR Sertindole OR Sulpiride OR Dogmatil OR Ziprasidone OR Geodon OR Ziprazidone OR Agomelatine OR Citalopram OR Clomipramine OR Desipramine OR Escitalopram OR Fluoxetine OR Prozac OR Fluvoxamine OR Imipramine OR Mianserin OR Milnacipran OR Mirtazapine OR Nortriptyline OR Paroxetine OR Sertraline OR Tianeptine OR Tianeptine OR Venlafaxine OR "m-chlorophenylpiperazine" OR "m-CPP" OR "1-(3-chlorophenyl)piperazine" OR Atomoxetine OR Strattera OR Dextromethorphan OR Fenfluramine OR Lamotrigine OR Levetiracetam OR Lisdexamfetamine OR Lithium OR MDMA OR "N-Methyl-3,4-methylenedioxyamphetamine" OR Ecstasy OR Methylenedioxymethamphetamine OR Methylphenidate OR Ritalin* OR Oxcarbazepine OR Topiramate OR Valproic Acid OR Divalproex OR Valproate OR Divalproate OR "(+)-5-FPT" OR "PRX-07034" OR Betahistin* OR Bromocriptine OR Buspirone OR Cyproheptadine OR Famotidine OR Levodopa OR "L-Dopa" OR "LP-211" OR "N-(4-cyanophenylmethyl)-4-(2-diphenyl)-1-piperazinehexanamide" OR Sumatriptan OR Volinanserin OR "M100907" OR Clonidine OR Guanfacine OR Dexmedetomidine OR Propranolol OR Acetylcysteine OR "ADX71149" OR "JNJ-40411813" OR "1-butyl-3-chloro-4-(4-phenyl-1-piperidinyl)-(1H)-pyridone" OR Amantadine OR "AZD8529" OR Basimglurant OR "2-chloro-4-(1-(4-fluorophenyl)-2,5-dimethyl-1H-imidazol-4-ylethynyl)pyridine" OR "CDPPB" OR "3-cyano-N-(1,3-diphenyl-1H-pyrazol-5-yl)benzamide" OR "CX516" OR "BDP 12" OR "1-(quinoxalin-6-ylcarbonyl)piperidine" OR "D-Cycloserine" OR Eglumetad OR Fenobam OR "GRN-529" OR Ketamine OR "LY 341495" OR "LY341495" OR "LY 379268" OR "LY379268" OR "LY 487379" OR "LY487379" OR Mavoglurant OR Memantine OR "MGS0039" OR "MPEP" OR "6-methyl-2-(phenylethynyl)pyridine" OR "MPX-004" OR "MPX-007" OR "MTEP" OR "NCFP" OR Riluzole OR "RO4491533" OR "TASP0433864" OR "A 867744" OR "4-(5-(4-chlorophenyl)-2-methyl-3-propionyl-1H-pyrrol-1-yl)benzenesulfonamide" OR Acamprosate OR "ADX71441" OR Arbaclofen OR "AZD7325" OR Baclofen OR Bumetanide OR "DMXB A" OR "DMXBA" OR "GTS 21" OR "3-(2,4-dimethoxybenzylidene)anabaseine" OR Donepezil OR "EVP-6124" OR "7-chloro-N-quinuclidin-3-yl-benzo(b)thiophene-2-carboxamide" OR Flumazenil OR Galantamin* OR Mecamylamine OR "PNU 120596" OR "PNU120596" OR "1-(5-chloro-2,4-dimethoxyphenyl)-3-(5-methylisoxazol-3-yl)urea" OR Pregnenolone OR Rivastigmine OR "SSR180711" OR Varenicline OR "AF38469" OR "AR-A014418" OR "N-(4-methoxybenzyl)-N'-(5-nitro-1,3-thiazol-2-yl)urea" OR Cannabidiol OR Cannabidivarin OR Celecoxib OR Everolimus OR Fingolimod OR Fluconazole OR Glatiramer OR Hydroxyfasudil OR Lenalidomide OR Minocycline OR Naltrexone OR "NVP-BKM120" OR Buparlisib OR "Oral Human Immunoglobulin" OR Pentoxifylline OR "SB 216763" OR "SB216763" OR Sirolimus OR Rapamycin OR Staurosporine OR Suramin OR Tacrolimus OR "TAK-242" OR "TAK242" OR Temsirolimus OR Tideglusib OR "NP031112" OR Amastatin OR Angiotensin* OR Carnitine OR Levocarnitine OR "CM-AT" OR "Diet Therapy" OR "Diet Therapies" OR "Dietary Supplements" OR "Dietary Supplement" OR Dimethylglycine OR "EPI-743" OR "alpha-Tocotrienol Quinone" OR "Food Supplement" OR "Food Supplements" OR "Gastrin-Releasing Peptide" OR Ghrelin OR "Herbal Supplement" OR "Herbal Supplements" OR Hormone* OR Corticosteroid* OR Corticoid* OR Hydrocortisone OR "IGF-1" OR "Insulin-Like Growth Factor I" OR Lovastatin OR Melanocortin* OR Melatonin OR Metformin OR Mineral* OR "NNZ-2566" OR "NNZ2566" OR "Nutrition Therapy" OR "Nutritional Therapy" OR Oligosaccharide* OR "Omega-3" OR "Omega3" OR "n-3 Fatty" OR "n-3 Polyunsaturated Fatty" OR "n-3 PUFA" OR "n3 PUFA" OR "n 3 Oils" OR "n 3 Oil" OR "n3 Fatty" OR "ORG-2766" OR Oxytocin OR Syntocinon OR Pioglitazone OR Prednisone OR Probiotic* OR Bifidobacter* OR Pyridoxine OR "RG7713" OR "Ro27 3225" OR Secretin OR Sulforaphane OR Sulforafan OR Tetrahydrobiopterin OR Sapropterin OR Thyroxine OR Triiodothyronine OR "T3" OR Trofinetide OR Vasopressin* OR Vitamin* OR "WAY-267464" OR "WAY267464" OR Arachidonic OR Arachidonate OR Ascorbic OR Ascorbate OR Carnosine OR Cyanocobalamin OR Cobalamin* OR Cobamide* OR Hydroxocobalamin OR Docosahexaenoic OR Docosahexaenoate OR Ferrous OR Folic OR Folate OR Ginkgo* OR Gingko* OR Ginko* OR Maidenhair OR Glutathione OR Gluten OR Inositol OR Leucovorin OR Folinic OR Magnesium OR Milk OR Papain OR Pepsin OR Pyridoxal OR Pyridoxamine OR Succimer OR Dimercaptosuccinic Acid OR DMSA OR "Trichuris Suis" OR Ubiquinol):ti,ab)

In Trials

### 2.3 EMBASE

1. Exp Autism/ OR "Asperger Syndrome"/ OR "Childhood Disintegrative Disorder"/ OR "Pervasive Developmental Disorder Not Otherwise Specified"/ OR "Rett Syndrome"/ OR (Autis* OR Kanner* OR Asperger* OR "Pervasive Child Development Disorder" OR "Pervasive Child Development Disorders" OR "Pervasive Developmental Disorder" OR "Pervasive Developmental Disorders" OR "Pervasive Development Disorder" OR "Pervasive Development Disorders" OR "Childhood Disintegrative Disorder" OR "Childhood Disintegrative Disorders" OR Rett* OR "Cerebroatrophic Hyperammonemia" OR "Cerebroatrophic Hyperammonemias").ti,ab.
2. Exp "Chemicals And Drugs"/ OR Exp *Drug/ OR *Behind the Counter Drug/ OR *Chinese Drug/ OR *Essential Drug/ OR *Generic Drug/ OR *Long Acting Drug/ OR *New Drug/ OR *Non Prescription Drug/ OR *Orphan Drug/ OR *Prescription Drug/ OR *Prodrug/ OR *Short Acting Drug/ OR *Unclassified Drug/ OR *Unindexed Drug/ OR Exp *Medicinal Plant/ OR Exp *Plant Extract/ OR Exp "Drug Combination"/ OR Exp *Drug Therapy/ OR Exp *Drug Effect/ OR Aripiprazole/ OR Clozapine/ OR Haloperidol/ OR Loxapine/ OR Lurasidone/ OR Paliperidone/ OR Quetiapine/ OR Risperidone/ OR Sulpiride/ OR Citalopram/ OR Clomipramine/ OR Desipramine/ OR Fluoxetine/ OR Fluvoxamine/ OR Imipramine/ OR Mianserin/ OR Nortriptyline/ OR Paroxetine/ OR Sertraline/ OR Venlafaxine/ OR Atomoxetine/ OR Dextromethorphan/ OR Fenfluramine/ OR Lisdexamfetamine/ OR "Lithium Carbonate"/ OR Methylphenidate/ OR Midomafetamine/ OR "Valproic Acid"/ OR Betahistine/ OR Bromocriptine/ OR Buspirone/ OR Cyproheptadine/ OR Famotidine/ OR Levodopa/ OR Sumatriptan/ OR Clonidine/ OR Guanfacine/ OR Dexmedetomidine/ OR Propranolol/ OR Acetylcysteine/ OR Amantadine/ OR Ketamine/ OR Memantine/ OR Riluzole/ OR Baclofen/ OR Bumetanide/ OR Flumazenil/ OR Galantamine/ OR Mecamylamine/ OR Pregnenolone/ OR Rivastigmine/ OR Varenicline/ OR Cannabidiol/ OR Celecoxib/ OR Everolimus/ OR Fingolimod/ OR Fluconazole/ OR Glatiramer/ OR (Immunoglobulin/ AND "Oral Drug Administration"/) OR Minocycline/ OR Naltrexone/ OR Pentoxifylline/ OR Rapamycin/ OR Staurosporine/ OR Suramin/ OR Tacrolimus/ OR "Somatomedin C"/ OR Exp *Corticosteroid/ OR Corticotropin/ OR Exp *Angiotensin Derivative/ OR Carnitine/ OR Exp *Diet Therapy/ OR *Diet Supplementation/ OR "Omega 3 Fatty Acid"/ OR "Gastrin Releasing Peptide"/ OR Ghrelin/ OR Hydrocortisone/ OR Mevinolin/ OR Melanocortin/ OR Melatonin/ OR Metformin/ OR Mineral/ OR Exp *Oligosaccharide/ OR Oxytocin/ OR Prednisone/ OR Probiotic Agent/ OR Pyridoxine/ OR Secretin/ OR Thyroid Hormone/ OR Thyroxine/ OR Liothyronine/ OR Exp *Vasopressin Derivative/ OR "alpha Tocopherol"/ OR "Arachidonic Acid"/ OR "Ascorbic Acid"/ OR Carnosine/ OR "Docosahexaenoic Acid"/ OR "Folic Acid"/ OR "Ginkgo biloba"/ OR Glutathione/ OR Gluten/ OR Inositol/ OR Folinic Acid/ OR Magnesium/ OR "Magnesium Oxide"/ OR Milk/ OR Papain/ OR Succimer/ OR Cyanocobalamin/ OR Pyridoxine/ OR "Vitamin D"/ OR Exp "alpha 2 Adrenergic Receptor Blocking Agent"/ OR Exp "Antidepressant Agent"/ OR Exp "Antiparkinson Agent"/ OR Exp "Antiemetic Agent"/ OR Exp "Neuroleptic Agent"/ OR Exp "Dopamine Receptor Stimulating Agent"/ OR Exp "Dopamine Receptor Blocking Agent"/ OR Exp "Dopamine 2 Receptor Blocking Agent"/ OR Exp "4 Aminobutyric Acid Receptor Blocking Agent"/ OR Exp "Serotonin 2 Antagonist"/ OR Exp "Serotonin Receptor Affecting Agent"/ OR Exp "Serotonin Antagonist"/ OR Exp "Serotonin Uptake Inhibitor"/ OR Exp "alpha Adrenergic Receptor Blocking Agent"/ OR Exp "Adrenergic Receptor Affecting Agent"/ OR Exp "Cytochrome P450 1A2 Inhibitor"/ OR Exp "Cytochrome P450 2C19 Inhibitor"/ OR Exp "Cytochrome P450 2D6 Inhibitor"/ OR Exp "Enzyme Inhibitor"/ OR Exp "Histamine H1 Receptor Antagonist"/ OR Exp Noradrenalin Uptake Inhibitor/ OR Exp "Serotonin Uptake Inhibitor"/ OR Exp "Anxiolytic Agent"/ OR Exp "Tricyclic Antidepressant Agent"/ OR Exp "Psychotropic Agent"/ OR Exp "Anticonvulsive Agent"/ OR Exp Tranquilizer/ OR Exp "Calcium Channel Blocking Agent"/ OR Exp "Central Stimulant Agent"/ OR Exp "Cytochrome P450 3A Inducer"/ OR Exp "Dopamine Uptake Inhibitor"/ OR Exp "Amino Acid Receptor Blocking Agent"/ OR Exp "GABAergic Receptor Affecting Agent"/ OR Exp "Psychedelic Agent"/ OR Exp "Neuroprotective Agent"/ OR Exp "Nootropic Agent"/ OR Exp "Serotonin Agonist"/ OR Exp "Voltage Gated Sodium Channel Blocking Agent"/ OR Exp "Histamine Agonist"/ OR Exp "Histamine H2 Receptor Antagonist"/ OR Exp "Hormone Antagonist"/ OR Exp "Serotonin 1 Agonist"/ OR Exp "alpha 2 Adrenergic Receptor Stimulating Agent"/ OR Exp "beta Adrenergic Receptor Blocking Agent"/ OR Exp "Adrenergic Receptor Blocking Agent"/ OR Exp "Amino Acid Receptor Stimulating Agent"/ OR Exp "Cholinesterase Inhibitor"/ OR Exp "Benzodiazepine Receptor Affecting Agent"/ OR Exp "4 Aminobutyric Acid B Receptor Stimulating Agent"/ OR Exp "Ganglion Blocking Agent"/ OR Exp "Nicotinic Agent"/ OR Exp "Nicotinic Receptor Blocking Agent"/ OR Exp "Cholinergic Receptor Stimulating Agent"/ OR Exp "Loop Diuretic Agent"/ OR Exp "Sterol 14alpha Demethylase Inhibitor"/ OR Exp "Immunological Adjuvant"/ OR Exp "Antiinfective Agent"/ OR Exp "Antifungal Agent"/ OR Exp "Cannabinoid Receptor Agonist"/ OR Exp "Cannabinoid Receptor Antagonist"/ OR Exp "Cannabinoid Receptor Affecting Agent"/ OR Exp "Central Nervous System Agents"/ OR Exp "Cyclooxygenase 2 Inhibitor"/ OR Exp "Cytochrome P450 2C9 Inhibitor"/ OR Exp "Immunologic Factor"/ OR Exp "Immunosuppressive Agent"/ OR Exp "Narcotic Antagonist"/ OR Exp "Agents Interacting With Transmitter, Hormone OR Drug Receptors"/ OR Exp "Purinergic Receptor Affecting Agent"/ OR Exp "Antiinflammatory Agent"/ OR Exp Antioxidant/ OR Exp "Central Depressant Agent"/ OR Exp "Chelating Agent"/ OR Exp Hormone/ OR Exp "Oxytocic Agent"/ OR Exp "Vitamin B Complex"/ OR Exp Vitamin/ OR (Anticonvuls* OR Antiepilep* OR Antipsychotic* OR Psychotropic* OR "Anti-Anxiety" OR Anxiolytic* OR Antidepress* OR "Pharmaco-Therapy" OR "Pharmaco-Therapies" OR Chemotherapy OR Chemotherapies OR Pharmacotherapy OR Pharmacotherapies OR "Pharmacological Interventions" OR "Pharmacological Intervention" OR "Pharmacological Treatment" OR "Pharmacological Treatments" OR "Drug Therapy" OR "Drug Therapies" OR Amisulpride OR Aripiprazol* OR Abilify OR Brexpiprazole OR Clozapine OR Clozaril OR Leponex OR Haloperidol OR Haldol OR Loxapine OR Lurasidone OR Latuda OR Olanzapine OR Zyprexa OR Paliperidone OR Invega OR Quetiapine OR Seroquel OR Risperidone OR Risperdal OR Risperidal OR Sertindole OR Sulpiride OR Dogmatil OR Ziprasidone OR Geodon OR Ziprazidone OR Agomelatine OR Citalopram OR Clomipramine OR Desipramine OR Escitalopram OR Fluoxetine OR Prozac OR Fluvoxamine OR Imipramine OR Mianserin OR Milnacipran OR Mirtazapine OR Nortriptyline OR Paroxetine OR Sertraline OR Tianeptine OR Tianeptine OR Venlafaxine OR "m-chlorophenylpiperazine" OR "m-CPP" OR "1-(3-chlorophenyl)piperazine" OR Atomoxetine OR Strattera OR Dextromethorphan OR Fenfluramine OR Lamotrigine OR Levetiracetam OR Lisdexamfetamine OR Lithium OR MDMA OR "N-Methyl-3,4-methylenedioxyamphetamine" OR Ecstasy OR Methylenedioxymethamphetamine OR Methylphenidate OR Ritalin* OR Oxcarbazepine OR Topiramate OR Valproic Acid OR Divalproex OR Valproate OR Divalproate OR "(+)-5-FPT" OR "PRX-07034" OR Betahistin* OR Bromocriptine OR Buspirone OR Cyproheptadine OR Famotidine OR Levodopa OR "L-Dopa" OR "LP-211" OR "N-(4-cyanophenylmethyl)-4-(2-diphenyl)-1-piperazinehexanamide" OR Sumatriptan OR Volinanserin OR "M100907" OR Clonidine OR Guanfacine OR Dexmedetomidine OR Propanolol OR Acetylcysteine OR "ADX71149" OR "JNJ-40411813" OR "1-butyl-3-chloro-4-(4-phenyl-1-piperidinyl)-(1H)-pyridone" OR Amantadine OR "AZD8529" OR Basimglurant OR "2-chloro-4-(1-(4-fluorophenyl)-2,5-dimethyl-1H-imidazol-4-ylethynyl)pyridine" OR "CDPPB" OR "3-cyano-N-(1,3-diphenyl-1H-pyrazol-5-yl)benzamide" OR "CX516" OR "BDP 12" OR "1-(quinoxalin-6-ylcarbonyl)piperidine" OR "D-Cycloserine" OR Eglumetad OR Fenobam OR "GRN-529" OR Ketamine OR "LY 341495" OR "LY341495" OR "LY 379268" OR "LY379268" OR "LY 487379" OR "LY487379" OR Mavoglurant OR Memantine OR "MGS0039" OR "MPEP" OR "6-methyl-2-(phenylethynyl)pyridine" OR "MPX-004" OR "MPX-007" OR "MTEP" OR "NCFP" OR Riluzole OR "RO4491533" OR "TASP0433864" OR "A 867744" OR "4-(5-(4-chlorophenyl)-2-methyl-3-propionyl-1H-pyrrol-1-yl)benzenesulfonamide" OR Acamprosate OR "ADX71441" OR Arbaclofen OR "AZD7325" OR Baclofen OR Bumetanide OR "DMXB A" OR "DMXBA" OR "GTS 21" OR "3-(2,4-dimethoxybenzylidene)anabaseine" OR Donepezil OR "EVP-6124" OR "7-chloro-N-quinuclidin-3-yl-benzo(b)thiophene-2-carboxamide" OR Flumazenil OR Galantamin* OR Mecamylamine OR "PNU 120596" OR "PNU120596" OR "1-(5-chloro-2,4-dimethoxyphenyl)-3-(5-methylisoxazol-3-yl)urea" OR Pregnenolone OR Rivastigmine OR "SSR180711" OR Varenicline OR "AF38469" OR "AR-A014418" OR "N-(4-methoxybenzyl)-N'-(5-nitro-1,3-thiazol-2-yl)urea" OR Cannabidiol OR Cannabidivarin OR Celecoxib OR Everolimus OR Fingolimod OR Fluconazole OR Glatiramer OR Hydroxyfasudil OR Lenalidomide OR Minocycline OR Naltrexone OR "NVP-BKM120" OR Buparlisib OR "Oral Human Immunoglobulin" OR Pentoxifylline OR "SB 216763" OR "SB216763" OR Sirolimus OR Rapamycin OR Staurosporine OR Suramin OR Tacrolimus OR "TAK-242" OR "TAK242" OR Temsirolimus OR Tideglusib OR "NP031112" OR Amastatin OR Angiotensin* OR Carnitine OR Levocarnitine OR "CM-AT" OR "Diet Therapy" OR "Diet Therapies" OR "Dietary Supplements" OR "Dietary Supplement" OR Dimethylglycine OR "EPI-743" OR "alpha-Tocotrienol Quinone" OR "Food Supplement" OR "Food Supplements" OR "Gastrin-Releasing Peptide" OR Ghrelin OR "Herbal Supplement" OR "Herbal Supplements" OR Hormone* OR Corticosteroid* OR Corticoid* OR Hydrocortisone OR "IGF-1" OR "Insulin-Like Growth Factor I" OR Lovastatin OR Melanocortin* OR Melatonin OR Metformin OR Mineral* OR "NNZ-2566" OR "NNZ2566" OR "Nutrition Therapy" OR "Nutritional Therapy" OR Oligosaccharide* OR "Omega-3" OR "Omega3" OR "n-3 Fatty" OR "n-3 Polyunsaturated Fatty" OR "n-3 PUFA" OR "n3 PUFA" OR "n 3 Oils" OR "n 3 Oil" OR "n3 Fatty" OR "ORG-2766" OR Oxytocin OR Syntocinon OR Pioglitazone OR Prednisone OR Probiotic* OR Bifidobacter* OR Pyridoxine OR "RG7713" OR "Ro27 3225" OR Secretin OR Sulforaphane OR Sulforafan OR Tetrahydrobiopterin OR Sapropterin OR Thyroxine OR Triiodothyronine OR "T3" OR Trofinetide OR Vasopressin* OR Vitamin* OR "WAY-267464" OR "WAY267464" OR Arachidonic OR Arachidonate OR Ascorbic OR Ascorbate OR Carnosine OR Cyanocobalamin OR Cobalamin* OR Cobamide* OR Hydroxocobalamin OR Docosahexaenoic OR Docosahexaenoate OR Ferrous OR Folic OR Folate OR Ginkgo* OR Gingko* OR Ginko* OR Maidenhair OR Glutathione OR Gluten OR Inositol OR Leucovorin OR Folinic OR Magnesium OR Milk OR Papain OR Pepsin OR Pyridoxal OR Pyridoxamine OR Succimer OR Dimercaptosuccinic Acid OR DMSA OR "Trichuris Suis" OR Ubiquinol).ti,ab.
3. Crossover Procedure/ OR Double Blind Procedure/ OR Randomized Controlled Trial/ OR Single Blind Procedure/ OR (Random* OR Factorial* OR Crossover* OR "Cross Over*" OR Placebo* OR (Doubl* adj Blind*) OR (Singl* adj Blind*) OR Assign* OR Allocat* OR Volunteer*).ti,ab.
4. 1 AND 2 AND 3
5. Exp Animals/ OR Exp Invertebrate/ OR Animal Experiment/ OR Animal Model/ OR Animal Tissue/ OR Animal Cell/ OR Nonhuman/
6. Human/ OR Normal Human/ OR Human Cell/
7. 5 AND 6
8. 5 NOT 7
9. 4 NOT 8

### 2.4 MEDLINE

1. "Autistic Disorder"/ OR "Autism Spectrum Disorder"/ OR "Asperger Syndrome"/ OR "Rett Syndrome"/ OR "Child Development Disorders, Pervasive"/ OR (Autis* OR Kanner* OR Asperger* OR "Pervasive Child Development Disorder" OR "Pervasive Child Development Disorders" OR "Pervasive Developmental Disorder" OR "Pervasive Developmental Disorders" OR "Pervasive Development Disorder" OR "Pervasive Development Disorders" OR "Childhood Disintegrative Disorder" OR "Childhood Disintegrative Disorders" OR Rett* OR "Cerebroatrophic Hyperammonemia" OR "Cerebroatrophic Hyperammonemias").ti,ab.
2. "Pharmaceutical Preparations"/ OR Exp "Drug Combinations"/ OR "Drugs, Chinese Herbal"/ OR "Drugs, Essential"/ OR "Drugs, Generic"/ OR "Drugs, Investigational"/ OR Exp "Nonprescription Drugs"/ OR Exp "Plant Extracts"/ OR "Prescription Drugs"/ OR Prodrugs/ OR "Pharmacologic Actions"/ OR Exp "Pharmacological Actions (Non MeSH)"/ OR Exp "Drug Therapy"/ OR Exp "Therapeutic Uses"/ OR Exp "Physiological Effects of Drugs"/ OR Aripiprazole/ OR Clozapine/ OR Haloperidol/ OR Loxapine/ OR "Lurasidone Hydrochloride"/ OR "Paliperidone Palmitate"/ OR "Quetiapine Fumarate"/ OR Risperidone/ OR Sulpiride/ OR Citalopram/ OR Clomipramine/ OR Desipramine/ OR Fluoxetine/ OR Fluvoxamine/ OR Imipramine/ OR Mianserin/ OR Nortriptyline/ OR Paroxetine/ OR Sertraline/ OR "Venlafaxine Hydrochloride"/ OR "Atomoxetine Hydrochloride"/ OR Dextromethorphan/ OR Fenfluramine/ OR "Lisdexamfetamine Dimesylate"/ OR "Lithium Carbonate"/ OR Methylphenidate/ OR "N-Methyl-3,4-methylenedioxyamphetamine"/ OR "Valproic Acid"/ OR Betahistine/ OR Bromocriptine/ OR Buspirone/ OR Cyproheptadine/ OR Famotidine/ OR Levodopa/ OR Sumatriptan/ OR Clonidine/ OR Guanfacine/ OR Dexmedetomidine/ OR Propranolol/ OR Acetylcysteine/ OR Amantadine/ OR Ketamine/ OR Memantine/ OR Riluzole/ OR Baclofen/ OR Bumetanide/ OR Flumazenil/ OR Galantamine/ OR Mecamylamine/ OR Pregnenolone/ OR Rivastigmine/ OR Varenicline/ OR Cannabidiol/ OR Celecoxib/ OR Everolimus/ OR "Fingolimod Hydrochloride"/ OR Fluconazole/ OR "Glatiramer Acetate"/ OR (Immunoglobulins/ AND "Administration, Oral"/) OR Minocycline/ OR Naltrexone/ OR Pentoxifylline/ OR Sirolimus/ OR Staurosporine/ OR Suramin/ OR Tacrolimus/ OR "Insulin-Like Growth Factor I"/ OR "Adrenal Cortex Hormones"/ OR "Adrenocorticotropic Hormone"/ OR Angiotensins/ OR Carnitine/ OR "Diet Therapy"/ OR "Dietary Supplements"/ OR "Fatty Acids, Omega-3"/ OR "Gastrin-Releasing Peptide"/ OR Ghrelin/ OR Hydrocortisone/ OR Lovastatin/ OR Melanocortins/ OR Melatonin/ OR Metformin/ OR Minerals/ OR "Nutrition Therapy"/ OR Oligosaccharides/ OR Oxytocin/ OR Prednisone/ OR Probiotics/ OR Pyridoxine/ OR Secretin/ OR "Thyroid Hormones"/ OR Thyroxine/ OR Triiodothyronine/ OR Vasopressins/ OR "Vitamin E"/ OR "Arachidonic Acid"/ OR "Ascorbic Acid"/ OR Carnosine/ OR "Docosahexaenoic Acids"/ OR "Folic Acid"/ OR "Ginkgo biloba"/ OR Glutathione/ OR Glutens/ OR Inositol/ OR Leucovorin/ OR Magnesium/ OR "Magnesium Oxide"/ OR Milk/ OR Papain/ OR Succimer/ OR "Vitamin B 12"/ OR "Vitamin B 6"/ OR "Vitamin D"/ OR "Adrenergic alpha-2 Receptor Antagonists"/ OR "Antidepressive Agents, Second-Generation"/ OR "Anti-Dyskinesia Agents"/ OR Antiemetics/ OR "Antipsychotic Agents"/ OR "Dopamine Agonists"/ OR "Dopamine Antagonists"/ OR "Dopamine D2 Receptor Antagonists"/ OR "GABA Antagonists"/ OR "Serotonin 5-HT2 Receptor Antagonists"/ OR "Serotonin Agents"/ OR "Serotonin Antagonists"/ OR "Serotonin Uptake Inhibitors"/ OR "Adrenergic alpha-Antagonists"/ OR "Adrenergic Uptake Inhibitors"/ OR "Cytochrome P-450 CYP1A2 Inhibitors"/ OR "Cytochrome P-450 CYP2C19 Inhibitors"/ OR "Cytochrome P-450 CYP2D6 Inhibitors"/ OR "Enzyme Inhibitors"/ OR "Histamine H1 Antagonists"/ OR "Serotonin and Noradrenaline Reuptake Inhibitors"/ OR "Anti-Anxiety Agents"/ OR "Antidepressive Agents"/ OR "Antidepressive Agents, Tricyclic"/ OR "Psychotropic Drugs"/ OR Anticonvulsants/ OR "Antimanic Agents"/ OR "Calcium Channel Blockers"/ OR "Central Nervous System Stimulants"/ OR "Cytochrome P-450 CYP3A Inducers"/ OR "Dopamine Uptake Inhibitors"/ OR "Excitatory Amino Acid Antagonists"/ OR "GABA Agents"/ OR Hallucinogens/ OR "Neuroprotective Agents"/ OR "Nootropic Agents"/ OR "Serotonin Receptor Agonists"/ OR "Voltage-Gated Sodium Channel Blockers"/ OR "Antiparkinson Agents"/ OR "Dopamine Agents"/ OR "Histamine Agonists"/ OR "Histamine H2 Antagonists"/ OR "Hormone Antagonists"/ OR "Serotonin 5-HT1 Receptor Agonists"/ OR "Adrenergic alpha-2 Receptor Agonists"/ OR "Adrenergic beta-Antagonists"/ OR Sympatholytics/ OR "Excitatory Amino Acid Agonists"/ OR "Cholinesterase Inhibitors"/ OR "GABA Modulators"/ OR "GABA-B Receptor Agonists"/ OR "Ganglionic Blockers"/ OR "Nicotinic Agonists"/ OR "Nicotinic Antagonists"/ OR Parasympathomimetics/ OR "Sodium Potassium Chloride Symporter Inhibitors"/ OR "14-alpha Demethylase Inhibitors"/ OR "Adjuvants, Immunologic"/ OR "Anti-Bacterial Agents"/ OR "Antifungal Agents"/ OR "Cannabinoid Receptor Agonists"/ OR "Cannabinoid Receptor Antagonists"/ OR "Cannabinoid Receptor Modulators"/ OR "Central Nervous System Agents"/ OR "Cyclooxygenase 2 Inhibitors"/ OR "Cytochrome P-450 CYP2C9 Inhibitors"/ OR "Immunologic Factors"/ OR "Immunosuppressive Agents"/ OR "Narcotic Antagonists"/ OR "Neurotransmitter Agents"/ OR "Purinergic Agents"/ OR "Anti-Inflammatory Agents"/ OR Antioxidants/ OR "Central Nervous System Depressants"/ OR "Chelating Agents"/ OR Hormones/ OR Oxytocics/ OR "Vitamin B Complex"/ OR Vitamins/ OR "Diet Therapy".sh. OR (Brexpiprazole OR Olanzapine OR Sertindole OR Ziprasidone OR Agomelatine OR Milnacipran OR Mirtazapine OR Tianeptine OR Tianeptine OR "1-(3-chlorophenyl)piperazine" OR Lamotrigine OR Levetiracetam OR Oxcarbazepine OR Topiramate OR "N-(4-cyanophenylmethyl)-4-(2-diphenyl)-1-piperazinehexanamide" OR Volinanserin OR "1-(quinoxalin-6-ylcarbonyl)piperidine" OR "1-butyl-3-chloro-4-(4-phenyl-1-piperidinyl)-(1H)-pyridone" OR "2-((4-tert-butylphenoxy)methyl)-5-methyl-2,3-dihydroimidazo(2,1-b)(1,3)oxazole-6-carboxamide" OR "2-amino-3-(3,4-dichlorobenzyloxy)-6-fluorobicyclo(3.1.0)hexane-2,6-dicarboxylic acid" OR "2-chloro-4-(1-(4-fluorophenyl)-2,5-dimethyl-1H-imidazol-4-ylethynyl)pyridine" OR "3-cyano-N-(1,3-diphenyl-1H-pyrazol-5-yl)benzamide" OR "4-(3-(2,6-dimethylpyridin-4-yl)phenyl)-7-methyl-8-trifluoromethyl-1,3-dihydrobenzo(b)(1,4)diazepin-2-one" OR "6-methyl-2-(phenylethynyl)pyridine" OR "AZD8529" OR Eglumetad OR Fenobam OR "GRN-529" OR "LY 341495" OR "LY 379268" OR Mavoglurant OR "MPX-004" OR "MPX-007" OR "N-(4-(2-methoxyphenoxy)phenyl)-N-(2,2,2-trifluoroethylsulfonyl)pyrid-3-ylmethylamine" OR "1-(5-chloro-2,4-dimethoxyphenyl)-3-(5-methylisoxazol-3-yl)urea" OR "3-(2,4-dimethoxybenzylidene)anabaseine" OR "4-(5-(4-chlorophenyl)-2-methyl-3-propionyl-1H-pyrrol-1-yl)benzenesulfonamide" OR "4-amino-8-(2-fluoro-6-methoxy-phenyl)-N-propylcinnoline-3-carboxamide" OR "7-chloro-N-quinuclidin-3-yl-benzo(b)thiophene-2-carboxamide" OR Acamprosate OR "ADX71441" OR "Arbaclofen Placarbil" OR Donepezil OR "SSR180711" OR "ethyl 6-(N-(2-chloro-4-fluorophenyl)sulfamoyl)cyclohex-1-ene-1-carboxylate" OR "N-(4-methoxybenzyl)-N'-(5-nitro-1,3-thiazol-2-yl)urea" OR "AF38469" OR Cannabidivarin OR Hydroxyfasudil OR Lenalidomide OR "NVP-BKM120" OR "SB 216763" OR Temsirolimus OR "NP 031112" OR "(6-chloro-1-(2-(dimethylamino)ethyl)indol-3-yl)-spiro(1H-isobenzofuran-3,4'-piperidine)-1'-yl-methanone" OR "4-(3,5-dihydroxybenzyl)-N-(2-methyl-4-((1-methyl-4,10-dihydropyrazolo(3,4-b)(1,5)benzodiazepin-5(1H)-yl)carbonyl)benzyl)piperazine-1-carboxamide" OR "alpha-tocotrienol quinone" OR "butir-His-Phe-Arg-Trp-Sar-NH2" OR Amastatin OR Dimethylglycine OR "NNZ 2566" OR "ORG 2766" OR Pioglitazone OR Sapropterin OR Sulforafan OR "Ferrous Sulfate" OR Ubiquinol).rn. OR (Anticonvuls* OR Antiepilep* OR Antipsychotic* OR Psychotropic* OR "Anti-Anxiety" OR Anxiolytic* OR Antidepress* OR "Pharmaco-Therapy" OR "Pharmaco-Therapies" OR Chemotherapy OR Chemotherapies OR Pharmacotherapy OR Pharmacotherapies OR "Pharmacological Interventions" OR "Pharmacological Intervention" OR "Pharmacological Treatment" OR "Pharmacological Treatments" OR "Drug Therapy" OR "Drug Therapies" OR Amisulpride OR Aripiprazol* OR Abilify OR Brexpiprazole OR Clozapine OR Clozaril OR Leponex OR Haloperidol OR Haldol OR Loxapine OR Lurasidone OR Latuda OR Olanzapine OR Zyprexa OR Paliperidone OR Invega OR Quetiapine OR Seroquel OR Risperidone OR Risperdal OR Risperidal OR Sertindole OR Sulpiride OR Dogmatil OR Ziprasidone OR Geodon OR Ziprazidone OR Agomelatine OR Citalopram OR Clomipramine OR Desipramine OR Escitalopram OR Fluoxetine OR Prozac OR Fluvoxamine OR Imipramine OR Mianserin OR Milnacipran OR Mirtazapine OR Nortriptyline OR Paroxetine OR Sertraline OR Tianeptine OR Tianeptine OR Venlafaxine OR "m-chlorophenylpiperazine" OR "m-CPP" OR "1-(3-chlorophenyl)piperazine" OR Atomoxetine OR Strattera OR Dextromethorphan OR Fenfluramine OR Lamotrigine OR Levetiracetam OR Lisdexamfetamine OR Lithium OR MDMA OR "N-Methyl-3,4-methylenedioxyamphetamine" OR Ecstasy OR Methylenedioxymethamphetamine OR Methylphenidate OR Ritalin* OR Oxcarbazepine OR Topiramate OR Valproic Acid OR Divalproex OR Valproate OR Divalproate OR "(+)-5-FPT" OR "PRX-07034" OR Betahistin* OR Bromocriptine OR Buspirone OR Cyproheptadine OR Famotidine OR Levodopa OR "L-Dopa" OR "LP-211" OR "N-(4-cyanophenylmethyl)-4-(2-diphenyl)-1-piperazinehexanamide" OR Sumatriptan OR Volinanserin OR "M100907" OR Clonidine OR Guanfacine OR Dexmedetomidine OR Propranolol OR Acetylcysteine OR "ADX71149" OR "JNJ-40411813" OR "1-butyl-3-chloro-4-(4-phenyl-1-piperidinyl)-(1H)-pyridone" OR Amantadine OR "AZD8529" OR Basimglurant OR "2-chloro-4-(1-(4-fluorophenyl)-2,5-dimethyl-1H-imidazol-4-ylethynyl)pyridine" OR "CDPPB" OR "3-cyano-N-(1,3-diphenyl-1H-pyrazol-5-yl)benzamide" OR "CX516" OR "BDP 12" OR "1-(quinoxalin-6-ylcarbonyl)piperidine" OR "D-Cycloserine" OR Eglumetad OR Fenobam OR "GRN-529" OR Ketamine OR "LY 341495" OR "LY341495" OR "LY 379268" OR "LY379268" OR "LY 487379" OR "LY487379" OR Mavoglurant OR Memantine OR "MGS0039" OR "MPEP" OR "6-methyl-2-(phenylethynyl)pyridine" OR "MPX-004" OR "MPX-007" OR "MTEP" OR "NCFP" OR Riluzole OR "RO4491533" OR "TASP0433864" OR "A 867744" OR "4-(5-(4-chlorophenyl)-2-methyl-3-propionyl-1H-pyrrol-1-yl)benzenesulfonamide" OR Acamprosate OR "ADX71441" OR Arbaclofen OR "AZD7325" OR Baclofen OR Bumetanide OR "DMXB A" OR "DMXBA" OR "GTS 21" OR "3-(2,4-dimethoxybenzylidene)anabaseine" OR Donepezil OR "EVP-6124" OR "7-chloro-N-quinuclidin-3-yl-benzo(b)thiophene-2-carboxamide" OR Flumazenil OR Galantamin* OR Mecamylamine OR "PNU 120596" OR "PNU120596" OR "1-(5-chloro-2,4-dimethoxyphenyl)-3-(5-methylisoxazol-3-yl)urea" OR Pregnenolone OR Rivastigmine OR "SSR180711" OR Varenicline OR "AF38469" OR "AR-A014418" OR "N-(4-methoxybenzyl)-N'-(5-nitro-1,3-thiazol-2-yl)urea" OR Cannabidiol OR Cannabidivarin OR Celecoxib OR Everolimus OR Fingolimod OR Fluconazole OR Glatiramer OR Hydroxyfasudil OR Lenalidomide OR Minocycline OR Naltrexone OR "NVP-BKM120" OR Buparlisib OR "Oral Human Immunoglobulin" OR Pentoxifylline OR "SB 216763" OR "SB216763" OR Sirolimus OR Rapamycin OR Staurosporine OR Suramin OR Tacrolimus OR "TAK-242" OR "TAK242" OR Temsirolimus OR Tideglusib OR "NP031112" OR Amastatin OR Angiotensin* OR Carnitine OR Levocarnitine OR "CM-AT" OR "Diet Therapy" OR "Diet Therapies" OR "Dietary Supplements" OR "Dietary Supplement" OR Dimethylglycine OR "EPI-743" OR "alpha-Tocotrienol Quinone" OR "Food Supplement" OR "Food Supplements" OR "Gastrin-Releasing Peptide" OR Ghrelin OR "Herbal Supplement" OR "Herbal Supplements" OR Hormone* OR Corticosteroid* OR Corticoid* OR Hydrocortisone OR "IGF-1" OR "Insulin-Like Growth Factor I" OR Lovastatin OR Melanocortin* OR Melatonin OR Metformin OR Mineral* OR "NNZ-2566" OR "NNZ2566" OR "Nutrition Therapy" OR "Nutritional Therapy" OR Oligosaccharide* OR "Omega-3" OR "Omega3" OR "n-3 Fatty" OR "n-3 Polyunsaturated Fatty" OR "n-3 PUFA" OR "n3 PUFA" OR "n 3 Oils" OR "n 3 Oil" OR "n3 Fatty" OR "ORG-2766" OR Oxytocin OR Syntocinon OR Pioglitazone OR Prednisone OR Probiotic* OR Bifidobacter* OR Pyridoxine OR "RG7713" OR "Ro27 3225" OR Secretin OR Sulforaphane OR Sulforafan OR Tetrahydrobiopterin OR Sapropterin OR Thyroxine OR Triiodothyronine OR "T3" OR Trofinetide OR Vasopressin* OR Vitamin* OR "WAY-267464" OR "WAY267464" OR Arachidonic OR Arachidonate OR Ascorbic OR Ascorbate OR Carnosine OR Cyanocobalamin OR Cobalamin* OR Cobamide* OR Hydroxocobalamin OR Docosahexaenoic OR Docosahexaenoate OR Ferrous OR Folic OR Folate OR Ginkgo* OR Gingko* OR Ginko* OR Maidenhair OR Glutathione OR Gluten OR Inositol OR Leucovorin OR Folinic OR Magnesium OR Milk OR Papain OR Pepsin OR Pyridoxal OR Pyridoxamine OR Succimer OR Dimercaptosuccinic Acid OR DMSA OR "Trichuris Suis" OR Ubiquinol).ti,ab.
3. Randomized Controlled Trial.pt. OR Controlled Clinical Trial.pt. OR (Randomi?ed OR Placebo OR Randomly OR Trial OR Groups).ti,ab. OR Drug Therapy.fs. NOT (Exp Animals/ NOT Humans.sh.)
4. 1 AND 2 AND 3

### 2.5 PsycINFO

1. Autism Spectrum Disorders/ OR Rett Syndrome/ OR (Autis* OR Kanner* OR Asperger* OR "Pervasive Child Development Disorder" OR "Pervasive Child Development Disorders" OR "Pervasive Developmental Disorder" OR "Pervasive Developmental Disorders" OR "Pervasive Development Disorder" OR "Pervasive Development Disorders" OR "Childhood Disintegrative Disorder" OR "Childhood Disintegrative Disorders" OR Rett* OR "Cerebroatrophic Hyperammonemia" OR "Cerebroatrophic Hyperammonemias").ti,ab.
2. Exp Drugs/ OR Exp Drug Therapy/ OR Exp Adrenergic Blocking Drugs/ OR Exp Adrenergic Drugs/ OR Exp Anti Inflammatory Drugs/ OR Antiandrogens/ OR Exp Antibiotics/ OR Exp Anticonvulsive Drugs/ OR Exp Antidepressant Drugs/ OR Exp Antiemetic Drugs/ OR Exp Antihistaminic Drugs/ OR Exp Antitremor Drugs/ OR Channel Blockers/ OR Exp Cholinergic Blocking Drugs/ OR Exp Cholinergic Drugs/ OR Exp Cholinomimetic Drugs/ OR Exp CNS Affecting Drugs/ OR Exp Dopamine Agonists/ OR Exp Enzyme Inhibitors/ OR Exp Ganglion Blocking Drugs/ OR Generic Drugs/ OR Exp Hallucinogenic Drugs/ OR Exp Narcotic Antagonists/ OR Exp Neurotransmitter Uptake Inhibitors/ OR Nonprescription Drugs/ OR Exp Nootropic Drugs/ OR Prescription Drugs/ OR Exp Psychotomimetic Drugs/ OR Exp Serotonin Agonists/ OR Exp Serotonin Antagonists/ OR Exp Sympatholytic Drugs/ OR Exp Sympathomimetic Drugs/ OR Exp Vitamins/ OR Exp Hormones/ OR Exp "Medicinal Herbs and Plants"/ OR Tricyclic Antidepressant Drugs/ OR Exp Neuroleptic Drugs/ OR Exp Dopamine Antagonists/ OR Exp Gamma Aminobutyric Acid Antagonists/ OR Exp Serotonin Reuptake Inhibitors/ OR Exp Serotonin Norepinephrine Reuptake Inhibitors/ OR Exp CNS Stimulating Drugs/ OR Exp Gamma Aminobutyric Acid Agonists/ OR Exp Cholinesterase Inhibitors/ OR Exp Immunologic Factors/ OR Exp Neurotransmitters/ OR Exp Antioxidants/ OR Exp CNS Depressant Drugs/ OR Aripiprazole/ OR Clozapine/ OR Haloperidol/ OR Loxapine/ OR Quetiapine/ OR Risperidone/ OR Sulpiride/ OR Citalopram/ OR Chlorimipramine/ OR Desipramine/ OR Fluoxetine/ OR Fluvoxamine/ OR Imipramine/ OR Mianserin/ OR Nortriptyline/ OR Paroxetine/ OR Sertraline/ OR Venlafaxine/ OR Atomoxetine/ OR Fenfluramine/ OR "Lithium Carbonate"/ OR Methylphenidate/ OR Methylenedioxymethamphetamine/ OR "Valproic Acid"/ OR Bromocriptine/ OR Buspirone/ OR Clonidine/ OR Propranolol/ OR Amantadine/ OR Ketamine/ OR Baclofen/ OR Galanthamine/ OR Mecamylamine/ OR (Immunoglobulins/ AND Oral*.ti,ab.) OR Naltrexone/ OR "Insulin-Like Growth Factor"/ OR "Adrenal Cortex Hormones"/ OR Corticotropin/ OR Angiotensin/ OR Diets/ OR "Dietary Supplements"/ OR Fatty Acids/ OR Ghrelin/ OR Hydrocortisone/ OR Melatonin/ OR Nutrition/ OR Oxytocin/ OR "Thyroid Hormones"/ OR Thyroxine/ OR Triiodothyronine/ OR Vasopressin/ OR Vitamin Therapy/ OR "Arachidonic Acid"/ OR "Ascorbic Acid"/ OR "Folic Acid"/ OR Magnesium/ OR (Anticonvuls* OR Antiepilep* OR Antipsychotic* OR Psychotropic* OR "Anti-Anxiety" OR Anxiolytic* OR Antidepress* OR "Pharmaco-Therapy" OR "Pharmaco-Therapies" OR Chemotherapy OR Chemotherapies OR Pharmacotherapy OR Pharmacotherapies OR "Pharmacological Interventions" OR "Pharmacological Intervention" OR "Pharmacological Treatment" OR "Pharmacological Treatments" OR "Drug Therapy" OR "Drug Therapies" OR Amisulpride OR Aripiprazol* OR Abilify OR Brexpiprazole OR Clozapine OR Clozaril OR Leponex OR Haloperidol OR Haldol OR Loxapine OR Lurasidone OR Latuda OR Olanzapine OR Zyprexa OR Paliperidone OR Invega OR Quetiapine OR Seroquel OR Risperidone OR Risperdal OR Risperidal OR Sertindole OR Sulpiride OR Dogmatil OR Ziprasidone OR Geodon OR Ziprazidone OR Agomelatine OR Citalopram OR Clomipramine OR Desipramine OR Escitalopram OR Fluoxetine OR Prozac OR Fluvoxamine OR Imipramine OR Mianserin OR Milnacipran OR Mirtazapine OR Nortriptyline OR Paroxetine OR Sertraline OR Tianeptine OR Tianeptine OR Venlafaxine OR "m-chlorophenylpiperazine" OR "m-CPP" OR "1-(3-chlorophenyl)piperazine" OR Atomoxetine OR Strattera OR Dextromethorphan OR Fenfluramine OR Lamotrigine OR Levetiracetam OR Lisdexamfetamine OR Lithium OR MDMA OR "N-Methyl-3,4-methylenedioxyamphetamine" OR Ecstasy OR Methylenedioxymethamphetamine OR Methylphenidate OR Ritalin* OR Oxcarbazepine OR Topiramate OR Valproic Acid OR Divalproex OR Valproate OR Divalproate OR "(+)-5-FPT" OR "PRX-07034" OR Betahistin* OR Bromocriptine OR Buspirone OR Cyproheptadine OR Famotidine OR Levodopa OR "L-Dopa" OR "LP-211" OR "N-(4-cyanophenylmethyl)-4-(2-diphenyl)-1-piperazinehexanamide" OR Sumatriptan OR Volinanserin OR "M100907" OR Clonidine OR Guanfacine OR Dexmedetomidine OR Propranolol OR Acetylcysteine OR "ADX71149" OR "JNJ-40411813" OR "1-butyl-3-chloro-4-(4-phenyl-1-piperidinyl)-(1H)-pyridone" OR Amantadine OR "AZD8529" OR Basimglurant OR "2-chloro-4-(1-(4-fluorophenyl)-2,5-dimethyl-1H-imidazol-4-ylethynyl)pyridine" OR "CDPPB" OR "3-cyano-N-(1,3-diphenyl-1H-pyrazol-5-yl)benzamide" OR "CX516" OR "BDP 12" OR "1-(quinoxalin-6-ylcarbonyl)piperidine" OR "D-Cycloserine" OR Eglumetad OR Fenobam OR "GRN-529" OR Ketamine OR "LY 341495" OR "LY341495" OR "LY 379268" OR "LY379268" OR "LY 487379" OR "LY487379" OR Mavoglurant OR Memantine OR "MGS0039" OR "MPEP" OR "6-methyl-2-(phenylethynyl)pyridine" OR "MPX-004" OR "MPX-007" OR "MTEP" OR "NCFP" OR Riluzole OR "RO4491533" OR "TASP0433864" OR "A 867744" OR "4-(5-(4-chlorophenyl)-2-methyl-3-propionyl-1H-pyrrol-1-yl)benzenesulfonamide" OR Acamprosate OR "ADX71441" OR Arbaclofen OR "AZD7325" OR Baclofen OR Bumetanide OR "DMXB A" OR "DMXBA" OR "GTS 21" OR "3-(2,4-dimethoxybenzylidene)anabaseine" OR Donepezil OR "EVP-6124" OR "7-chloro-N-quinuclidin-3-yl-benzo(b)thiophene-2-carboxamide" OR Flumazenil OR Galantamin* OR Mecamylamine OR "PNU 120596" OR "PNU120596" OR "1-(5-chloro-2,4-dimethoxyphenyl)-3-(5-methylisoxazol-3-yl)urea" OR Pregnenolone OR Rivastigmine OR "SSR180711" OR Varenicline OR "AF38469" OR "AR-A014418" OR "N-(4-methoxybenzyl)-N'-(5-nitro-1,3-thiazol-2-yl)urea" OR Cannabidiol OR Cannabidivarin OR Celecoxib OR Everolimus OR Fingolimod OR Fluconazole OR Glatiramer OR Hydroxyfasudil OR Lenalidomide OR Minocycline OR Naltrexone OR "NVP-BKM120" OR Buparlisib OR "Oral Human Immunoglobulin" OR Pentoxifylline OR "SB 216763" OR "SB216763" OR Sirolimus OR Rapamycin OR Staurosporine OR Suramin OR Tacrolimus OR "TAK-242" OR "TAK242" OR Temsirolimus OR Tideglusib OR "NP031112" OR Amastatin OR Angiotensin* OR Carnitine OR Levocarnitine OR "CM-AT" OR "Diet Therapy" OR "Diet Therapies" OR "Dietary Supplements" OR "Dietary Supplement" OR Dimethylglycine OR "EPI-743" OR "alpha-Tocotrienol Quinone" OR "Food Supplement" OR "Food Supplements" OR "Gastrin-Releasing Peptide" OR Ghrelin OR "Herbal Supplement" OR "Herbal Supplements" OR Hormone* OR Corticosteroid* OR Corticoid* OR Hydrocortisone OR "IGF-1" OR "Insulin-Like Growth Factor I" OR Lovastatin OR Melanocortin* OR Melatonin OR Metformin OR Mineral* OR "NNZ-2566" OR "NNZ2566" OR "Nutrition Therapy" OR "Nutritional Therapy" OR Oligosaccharide* OR "Omega-3" OR "Omega3" OR "n-3 Fatty" OR "n-3 Polyunsaturated Fatty" OR "n-3 PUFA" OR "n3 PUFA" OR "n 3 Oils" OR "n 3 Oil" OR "n3 Fatty" OR "ORG-2766" OR Oxytocin OR Syntocinon OR Pioglitazone OR Prednisone OR Probiotic* OR Bifidobacter* OR Pyridoxine OR "RG7713" OR "Ro27 3225" OR Secretin OR Sulforaphane OR Sulforafan OR Tetrahydrobiopterin OR Sapropterin OR Thyroxine OR Triiodothyronine OR "T3" OR Trofinetide OR Vasopressin* OR Vitamin* OR "WAY-267464" OR "WAY267464" OR Arachidonic OR Arachidonate OR Ascorbic OR Ascorbate OR Carnosine OR Cyanocobalamin OR Cobalamin* OR Cobamide* OR Hydroxocobalamin OR Docosahexaenoic OR Docosahexaenoate OR Ferrous OR Folic OR Folate OR Ginkgo* OR Gingko* OR Ginko* OR Maidenhair OR Glutathione OR Gluten OR Inositol OR Leucovorin OR Folinic OR Magnesium OR Milk OR Papain OR Pepsin OR Pyridoxal OR Pyridoxamine OR Succimer OR Dimercaptosuccinic Acid OR DMSA OR "Trichuris Suis" OR Ubiquinol).ti,ab.
3. Exp Treatment Effectiveness Evaluation/ OR Clinical Trials/ OR Mental Health Program Evaluation/ OR Placebo/ OR (Random* OR Factorial* OR Crossover* OR Cross Over* OR Placebo* OR ((Singl* OR Doubl* OR Trebl* or Tripl*) adj (Mask* OR Blind*)) OR Assign* OR Allocat* OR Volunteer* OR Groups OR Trial*).ti,ab.
4. 1 AND 2 AND 3

### 2.6 PubMed

("Autistic Disorder"[MH] OR "Autism Spectrum Disorder"[MH] OR "Asperger Syndrome"[MH] OR "Rett Syndrome"[MH] OR "Child Development Disorders, Pervasive"[MH] OR Autis*[TIAB] OR Kanner*[TIAB] OR Asperger*[TIAB] OR "Pervasive Child Development Disorders"[TIAB] OR "Pervasive Developmental Disorder"[TIAB] OR "Pervasive Developmental Disorders"[TIAB] OR "Pervasive Development Disorder"[TIAB] OR "Pervasive Development Disorders"[TIAB] OR "Childhood Disintegrative Disorder"[TIAB] OR Rett*[TIAB]) AND ("Adrenergic alpha-2 Receptor Antagonists"[PA] OR "Antidepressive Agents, Second-Generation"[PA] OR "Anti-Dyskinesia Agents"[PA] OR Antiemetics[PA] OR "Antipsychotic Agents"[PA] OR "Dopamine Agonists"[PA] OR "Dopamine Antagonists"[PA] OR "Dopamine D2 Receptor Antagonists"[PA] OR "GABA Antagonists"[PA] OR "Serotonin 5-HT2 Receptor Antagonists"[PA] OR "Serotonin Agents"[PA] OR "Serotonin Antagonists"[PA] OR "Serotonin Uptake Inhibitors"[PA] OR "Adrenergic alpha-Antagonists"[PA] OR "Adrenergic Uptake Inhibitors"[PA] OR "Cytochrome P-450 CYP1A2 Inhibitors"[PA] OR "Cytochrome P-450 CYP2C19 Inhibitors"[PA] OR "Cytochrome P-450 CYP2D6 Inhibitors"[PA] OR "Enzyme Inhibitors"[PA] OR "Histamine H1 Antagonists"[PA] OR "Serotonin and Noradrenaline Reuptake Inhibitors"[PA] OR "Anti-Anxiety Agents"[PA] OR "Antidepressive Agents"[PA] OR "Antidepressive Agents, Tricyclic"[PA] OR "Psychotropic Drugs"[PA] OR Anticonvulsants[PA] OR "Antimanic Agents"[PA] OR "Calcium Channel Blockers"[PA] OR "Central Nervous System Stimulants"[PA] OR "Cytochrome P-450 CYP3A Inducers"[PA] OR "Dopamine Uptake Inhibitors"[PA] OR "Excitatory Amino Acid Antagonists"[PA] OR "GABA Agents"[PA] OR Hallucinogens[PA] OR "Neuroprotective Agents"[PA] OR "Nootropic Agents"[PA] OR "Serotonin Receptor Agonists"[PA] OR "Voltage-Gated Sodium Channel Blockers"[PA] OR "Antiparkinson Agents"[PA] OR "Dopamine Agents"[PA] OR "Histamine Agonists"[PA] OR "Histamine H2 Antagonists"[PA] OR "Hormone Antagonists"[PA] OR "Serotonin 5-HT1 Receptor Agonists"[PA] OR "Adrenergic alpha-2 Receptor Agonists"[PA] OR "Adrenergic beta-Antagonists"[PA] OR Sympatholytics[PA] OR "Excitatory Amino Acid Agonists"[PA] OR "Cholinesterase Inhibitors"[PA] OR "GABA Modulators"[PA] OR "GABA-B Receptor Agonists"[PA] OR "Ganglionic Blockers"[PA] OR "Nicotinic Agonists"[PA] OR "Nicotinic Antagonists"[PA] OR Parasympathomimetics[PA] OR "Sodium Potassium Chloride Symporter Inhibitors"[PA] OR "14-alpha Demethylase Inhibitors"[PA] OR "Adjuvants, Immunologic"[PA] OR "Anti-Bacterial Agents"[PA] OR "Antifungal Agents"[PA] OR "Cannabinoid Receptor Agonists"[PA] OR "Cannabinoid Receptor Antagonists"[PA] OR "Cannabinoid Receptor Modulators"[PA] OR "Central Nervous System Agents"[PA] OR "Cyclooxygenase 2 Inhibitors"[PA] OR "Cytochrome P-450 CYP2C9 Inhibitors"[PA] OR "Immunologic Factors"[PA] OR "Immunosuppressive Agents"[PA] OR "Narcotic Antagonists"[PA] OR "Neurotransmitter Agents"[PA] OR "Purinergic Agents"[PA] OR "Anti-Inflammatory Agents"[PA] OR Antioxidants[PA] OR "Central Nervous System Depressants"[PA] OR "Chelating Agents"[PA] OR Hormones[PA] OR Oxytocics[PA] OR "Vitamin B Complex"[PA] OR Vitamins[PA] OR "Pharmaceutical Preparations"[MH:NoExp] OR "Drug Combinations"[MH] OR "Drugs, Chinese Herbal"[MH] OR "Drugs, Essential"[MH] OR "Drugs, Generic"[MH] OR "Drugs, Investigational"[MH] OR "Nonprescription Drugs"[MH] or "Plant Extracts"[MH] OR "Prescription Drugs"[MH] OR Prodrugs[MH] OR "Pharmacologic Actions"[MH] OR "Drug Therapy"[MH] OR "Therapeutic Uses"[MH] OR "Physiological Effects of Drugs"[MH] OR Aripiprazole[MH] OR Clozapine[MH] OR Haloperidol[MH] OR Loxapine[MH] OR "Lurasidone Hydrochloride"[MH] OR "Paliperidone Palmitate"[MH] OR "Quetiapine Fumarate"[MH] OR Risperidone[MH] OR Sulpiride[MH] OR Citalopram[MH] OR Clomipramine[MH] OR Desipramine[MH] OR Fluoxetine[MH] OR Fluvoxamine[MH] OR Imipramine[MH] OR Mianserin[MH] OR Nortriptyline[MH] OR Paroxetine[MH] OR Sertraline[MH] OR "Venlafaxine Hydrochloride"[MH] OR "Atomoxetine Hydrochloride"[MH] OR Dextromethorphan[MH] OR Fenfluramine[MH] OR "Lisdexamfetamine Dimesylate"[MH] OR "Lithium Carbonate"[MH] OR Methylphenidate[MH] OR "N-Methyl-3,4-methylenedioxyamphetamine"[MH] OR "Valproic Acid"[MH] OR Betahistine[MH] OR Bromocriptine[MH] OR Buspirone[MH] OR Cyproheptadine[MH] OR Famotidine[MH] OR Levodopa[MH] OR Sumatriptan[MH] OR Clonidine[MH] OR Guanfacine[MH] OR Dexmedetomidine[MH] OR Propranolol[MH] OR Acetylcysteine[MH] OR Amantadine[MH] OR Ketamine[MH] OR Memantine[MH] OR Riluzole[MH] OR Baclofen[MH] OR Bumetanide[MH] OR Flumazenil[MH] OR Galantamine[MH] OR Mecamylamine[MH] OR Pregnenolone[MH] OR Rivastigmine[MH] OR Varenicline[MH] OR Cannabidiol[MH] OR Celecoxib[MH] OR Everolimus[MH] OR "Fingolimod Hydrochloride"[MH] OR Fluconazole[MH] OR "Glatiramer Acetate"[MH] OR (Immunoglobulins[MH] AND "Administration, Oral"[MH]) OR Minocycline[MH] OR Naltrexone[MH] OR Pentoxifylline[MH] OR Sirolimus[MH] OR Staurosporine[MH] OR Suramin[MH] OR Tacrolimus[MH] OR "Insulin-Like Growth Factor I"[MH] OR"Adrenal Cortex Hormones"[MH] OR "Adrenocorticotropic Hormone"[MH] OR Angiotensins[MH] OR Carnitine[MH] OR "Diet Therapy"[MH] OR "Dietary Supplements"[MH] OR "Fatty Acids, Omega-3"[MH] OR "Gastrin-Releasing Peptide"[MH] OR Ghrelin[MH] OR Hydrocortisone[MH] OR Lovastatin[MH] OR Melanocortins[MH] OR Melatonin[MH] OR Metformin[MH] OR Minerals[MH] OR "Nutrition Therapy"[MH] OR Oligosaccharides[MH] OR Oxytocin[MH] OR Prednisone[MH] OR Probiotics[MH] OR Pyridoxine[MH] OR Secretin[MH] OR "Thyroid Hormones"[MH] OR Thyroxine[MH] OR Triiodothyronine[MH] OR Vasopressins[MH] OR "Vitamin E"[MH] OR "Arachidonic Acid"[MH] OR "Ascorbic Acid"[MH] OR Carnosine[MH] OR "Docosahexaenoic Acids"[MH] OR "Folic Acid"[MH] OR "Ginkgo biloba"[MH] OR Glutathione[MH] OR Glutens[MH] OR Inositol[MH] OR Leucovorin[MH] OR Magnesium[MH] OR "Magnesium Oxide"[MH] OR Milk[MH] OR Papain[MH] OR Succimer[MH] OR "Vitamin B 12"[MH] OR "Vitamin B 6"[MH] OR "Vitamin D"[MH] OR "Adrenergic alpha-2 Receptor Antagonists"[MH] OR "Antidepressive Agents, Second-Generation"[MH] OR "Anti-Dyskinesia Agents"[MH] OR Antiemetics[MH] OR "Antipsychotic Agents"[MH] OR "Dopamine Agonists"[MH] OR "Dopamine Antagonists"[MH] OR "Dopamine D2 Receptor Antagonists"[MH] OR "GABA Antagonists"[MH] OR "Serotonin 5-HT2 Receptor Antagonists"[MH] OR "Serotonin Agents"[MH] OR "Serotonin Antagonists"[MH] OR "Serotonin Uptake Inhibitors"[MH] OR "Adrenergic alpha-Antagonists"[MH] OR "Adrenergic Uptake Inhibitors"[MH] OR "Cytochrome P-450 CYP1A2 Inhibitors"[MH] OR "Cytochrome P-450 CYP2C19 Inhibitors"[MH] OR "Cytochrome P-450 CYP2D6 Inhibitors"[MH] OR "Enzyme Inhibitors"[MH] OR "Histamine H1 Antagonists"[MH] OR "Serotonin and Noradrenaline Reuptake Inhibitors"[MH] OR "Anti-Anxiety Agents"[MH] OR "Antidepressive Agents"[MH] OR "Antidepressive Agents, Tricyclic"[MH] OR "Psychotropic Drugs"[MH] OR Anticonvulsants[MH] OR "Antimanic Agents"[MH] OR "Calcium Channel Blockers"[MH] OR "Central Nervous System Stimulants"[MH] OR "Cytochrome P-450 CYP3A Inducers"[MH] OR "Dopamine Uptake Inhibitors"[MH] OR "Excitatory Amino Acid Antagonists"[MH] OR "GABA Agents"[MH] OR Hallucinogens[MH] OR "Neuroprotective Agents"[MH] OR "Nootropic Agents"[MH] OR "Serotonin Receptor Agonists"[MH] OR "Voltage-Gated Sodium Channel Blockers"[MH] OR "Antiparkinson Agents"[MH] OR "Dopamine Agents"[MH] OR "Histamine Agonists"[MH] OR "Histamine H2 Antagonists"[MH] OR "Hormone Antagonists"[MH] OR "Serotonin 5-HT1 Receptor Agonists"[MH] OR "Adrenergic alpha-2 Receptor Agonists"[MH] OR "Adrenergic beta-Antagonists"[MH] OR Sympatholytics[MH] OR "Excitatory Amino Acid Agonists"[MH] OR "Cholinesterase Inhibitors"[MH] OR "GABA Modulators"[MH] OR "GABA-B Receptor Agonists"[MH] OR "Ganglionic Blockers"[MH] OR "Nicotinic Agonists"[MH] OR "Nicotinic Antagonists"[MH] OR Parasympathomimetics[MH] OR "Sodium Potassium Chloride Symporter Inhibitors"[MH] OR "14-alpha Demethylase Inhibitors"[MH] OR "Adjuvants, Immunologic"[MH] OR "Anti-Bacterial Agents"[MH] OR "Antifungal Agents"[MH] OR "Cannabinoid Receptor Agonists"[MH] OR "Cannabinoid Receptor Antagonists"[MH] OR "Cannabinoid Receptor Modulators"[MH] OR "Central Nervous System Agents"[MH] OR "Cyclooxygenase 2 Inhibitors"[MH] OR "Cytochrome P-450 CYP2C9 Inhibitors"[MH] OR "Immunologic Factors"[MH] OR "Immunosuppressive Agents"[MH] OR "Narcotic Antagonists"[MH] OR "Neurotransmitter Agents"[MH] OR "Purinergic Agents"[MH] OR "Anti-Inflammatory Agents"[MH] OR Antioxidants[MH] OR "Central Nervous System Depressants"[MH] OR "Chelating Agents"[MH] OR Hormones[MH] OR Oxytocics[MH] OR "Vitamin B Complex"[MH] OR Vitamins[MH] OR "Diet Therapy"[sh] OR Brexpiprazole[NM] OR Olanzapine[NM] OR Sertindole[NM] OR Ziprasidone[NM] OR Agomelatine[NM] OR Milnacipran[NM] OR Mirtazapine[NM] OR Tianeptine[NM] OR Tianeptine[NM] OR "1-(3-chlorophenyl)piperazine"[NM] OR Lamotrigine[NM] OR Levetiracetam[NM] OR Oxcarbazepine[NM] OR Topiramate[NM] OR "N-(4-cyanophenylmethyl)-4-(2-diphenyl)-1-piperazinehexanamide"[NM] OR Volinanserin[NM] OR "1-(quinoxalin-6-ylcarbonyl)piperidine"[NM] OR "1-butyl-3-chloro-4-(4-phenyl-1-piperidinyl)-(1H)-pyridone"[NM] OR "2-((4-tert-butylphenoxy)methyl)-5-methyl-2,3-dihydroimidazo(2,1-b)(1,3)oxazole-6-carboxamide"[NM] OR "2-amino-3-(3,4-dichlorobenzyloxy)-6-fluorobicyclo(3.1.0)hexane-2,6-dicarboxylic acid"[NM] OR "2-chloro-4-(1-(4-fluorophenyl)-2,5-dimethyl-1H-imidazol-4-ylethynyl)pyridine"[NM] OR "3-cyano-N-(1,3-diphenyl-1H-pyrazol-5-yl)benzamide"[NM] OR "4-(3-(2,6-dimethylpyridin-4-yl)phenyl)-7-methyl-8-trifluoromethyl-1,3-dihydrobenzo(b)(1,4)diazepin-2-one"[NM] OR "6-methyl-2-(phenylethynyl)pyridine"[NM] OR "AZD8529"[NM] OR Eglumetad[NM] OR Fenobam[NM] OR "GRN-529"[NM] OR "LY 341495"[NM] OR "LY 379268"[NM] OR Mavoglurant[NM] OR "MPX-004"[NM] OR "MPX-007"[NM] OR "N-(4-(2-methoxyphenoxy)phenyl)-N-(2,2,2-trifluoroethylsulfonyl)pyrid-3-ylmethylamine"[NM] OR "1-(5-chloro-2,4-dimethoxyphenyl)-3-(5-methylisoxazol-3-yl)urea"[NM] OR "3-(2,4-dimethoxybenzylidene)anabaseine"[NM] OR "4-(5-(4-chlorophenyl)-2-methyl-3-propionyl-1H-pyrrol-1-yl)benzenesulfonamide"[NM] OR "4-amino-8-(2-fluoro-6-methoxy-phenyl)-N-propylcinnoline-3-carboxamide"[NM] OR "7-chloro-N-quinuclidin-3-yl-benzo(b)thiophene-2-carboxamide"[NM] OR Acamprosate[NM] OR "ADX71441"[NM] OR "Arbaclofen Placarbil"[NM] OR Donepezil[NM] OR "SSR180711"[NM] OR "ethyl 6-(N-(2-chloro-4-fluorophenyl)sulfamoyl)cyclohex-1-ene-1-carboxylate"[NM] OR "N-(4-methoxybenzyl)-N'-(5-nitro-1,3-thiazol-2-yl)urea"[NM] OR "AF38469"[NM] OR Cannabidivarin[NM] OR Hydroxyfasudil[NM] OR Lenalidomide[NM] OR "NVP-BKM120"[NM] OR "SB 216763"[NM] OR Temsirolimus[NM] OR "NP 031112"[NM] OR "(6-chloro-1-(2-(dimethylamino)ethyl)indol-3-yl)-spiro(1H-isobenzofuran-3,4'-piperidine)-1'-yl-methanone"[NM] OR "4-(3,5-dihydroxybenzyl)-N-(2-methyl-4-((1-methyl-4,10-dihydropyrazolo(3,4-b)(1,5)benzodiazepin-5(1H)-yl)carbonyl)benzyl)piperazine-1-carboxamide"[NM] OR "alpha-tocotrienol quinone"[NM] OR "butir-His-Phe-Arg-Trp-Sar-NH2"[NM] OR Amastatin[NM] OR Dimethylglycine[NM] OR "NNZ 2566"[NM] OR "ORG 2766"[NM] OR Pioglitazone[NM] OR Sapropterin[NM] OR Sulforafan[NM] OR "Ferrous Sulfate"[NM] OR Ubiquinol[NM] OR Anticonvuls*[TIAB] OR Antiepilep*[TIAB] OR Antipsychotic*[TIAB] OR Psychotropic*[TIAB] OR "Anti-Anxiety"[TIAB] OR Anxiolytic*[TIAB] OR Antidepress*[TIAB] OR "Pharmaco-Therapy"[TIAB] OR "Pharmaco-Therapies"[TIAB] OR Chemotherapy[TIAB] OR Chemotherapies[TIAB] OR Pharmacotherapy[TIAB] OR Pharmacotherapies[TIAB] OR "Pharmacological Interventions"[TIAB] OR "Pharmacological Intervention"[TIAB] OR "Pharmacological Treatment"[TIAB] OR "Pharmacological Treatments"[TIAB] OR "Drug Therapy"[TIAB] OR "Drug Therapies"[TIAB] OR Amisulpride[TIAB] OR Aripiprazol*[TIAB] OR Abilify[TIAB] OR Brexpiprazole[TIAB] OR Clozapine[TIAB] OR Clozaril[TIAB] OR Leponex[TIAB] OR Haloperidol[TIAB] OR Haldol[TIAB] OR Loxapine[TIAB] OR Lurasidone[TIAB] OR Latuda[TIAB] OR Olanzapine[TIAB] OR Zyprexa[TIAB] OR Paliperidone[TIAB] OR Invega[TIAB] OR Quetiapine[TIAB] OR Seroquel[TIAB] OR Risperidone[TIAB] OR Risperdal[TIAB] OR Risperidal[TIAB] OR Sertindole[TIAB] OR Sulpiride[TIAB] OR Dogmatil[TIAB] OR Ziprasidone[TIAB] OR Geodon[TIAB] OR Ziprazidone[TIAB] OR Agomelatine[TIAB] OR Citalopram[TIAB] OR Clomipramine[TIAB] OR Desipramine[TIAB] OR Escitalopram[TIAB] OR Fluoxetine[TIAB] OR Prozac[TIAB] OR Fluvoxamine[TIAB] OR Imipramine[TIAB] OR Mianserin[TIAB] OR Milnacipran[TIAB] OR Mirtazapine[TIAB] OR Nortriptyline[TIAB] OR Paroxetine[TIAB] OR Sertraline[TIAB] OR Tianeptine[TIAB] OR Tianeptine[TIAB] OR Venlafaxine[TIAB] OR "m-chlorophenylpiperazine"[TIAB] OR "m-CPP"[TIAB] OR "1-(3-chlorophenyl)piperazine"[TIAB] OR Atomoxetine[TIAB] OR Strattera[TIAB] OR Dextromethorphan[TIAB] OR Fenfluramine[TIAB] OR Lamotrigine[TIAB] OR Levetiracetam[TIAB] OR Lisdexamfetamine[TIAB] OR Lithium[TIAB] OR MDMA[TIAB] OR "N-Methyl-3,4-methylenedioxyamphetamine"[TIAB] OR Ecstasy[TIAB] OR Methylenedioxymethamphetamine[TIAB] OR Methylphenidate[TIAB] OR Ritalin*[TIAB] OR Oxcarbazepine[TIAB] OR Topiramate[TIAB] OR Valproic Acid[TIAB] OR Divalproex[TIAB] OR Valproate[TIAB] OR Divalproate[TIAB] OR "(+)-5-FPT"[TIAB] OR "PRX-07034"[TIAB] OR Betahistin*[TIAB] OR Bromocriptine[TIAB] OR Buspirone[TIAB] OR Cyproheptadine[TIAB] OR Famotidine[TIAB] OR Levodopa[TIAB] OR "L-Dopa"[TIAB] OR "LP-211"[TIAB] OR "N-(4-cyanophenylmethyl)-4-(2-diphenyl)-1-piperazinehexanamide"[TIAB] OR Sumatriptan[TIAB] OR Volinanserin[TIAB] OR "M100907"[TIAB] OR Clonidine[TIAB] OR Guanfacine[TIAB] OR Dexmedetomidine[TIAB] OR Propranolol[TIAB] OR Acetylcysteine[TIAB] OR "ADX71149"[TIAB] OR "JNJ-40411813"[TIAB] OR "1-butyl-3-chloro-4-(4-phenyl-1-piperidinyl)-(1H)-pyridone"[TIAB] OR Amantadine[TIAB] OR "AZD8529"[TIAB] OR Basimglurant[TIAB] OR "2-chloro-4-(1-(4-fluorophenyl)-2,5-dimethyl-1H-imidazol-4-ylethynyl)pyridine"[TIAB] OR "CDPPB"[TIAB] OR "3-cyano-N-(1,3-diphenyl-1H-pyrazol-5-yl)benzamide"[TIAB] OR "CX516"[TIAB] OR "BDP 12"[TIAB] OR "1-(quinoxalin-6-ylcarbonyl)piperidine"[TIAB] OR "D-Cycloserine"[TIAB] OR Eglumetad[TIAB] OR Fenobam[TIAB] OR "GRN-529"[TIAB] OR Ketamine[TIAB] OR "LY 341495"[TIAB] OR "LY341495"[TIAB] OR "LY 379268"[TIAB] OR "LY379268"[TIAB] OR "LY 487379"[TIAB] OR "LY487379"[TIAB] OR Mavoglurant[TIAB] OR Memantine[TIAB] OR "MGS0039"[TIAB] OR "MPEP"[TIAB] OR "6-methyl-2-(phenylethynyl)pyridine"[TIAB] OR "MPX-004"[TIAB] OR "MPX-007"[TIAB] OR "MTEP"[TIAB] OR "NCFP"[TIAB] OR Riluzole[TIAB] OR "RO4491533"[TIAB] OR "TASP0433864"[TIAB] OR "A 867744"[TIAB] OR "4-(5-(4-chlorophenyl)-2-methyl-3-propionyl-1H-pyrrol-1-yl)benzenesulfonamide"[TIAB] OR Acamprosate[TIAB] OR "ADX71441"[TIAB] OR Arbaclofen[TIAB] OR "AZD7325"[TIAB] OR Baclofen[TIAB] OR Bumetanide[TIAB] OR "DMXB A"[TIAB] OR "DMXBA"[TIAB] OR "GTS 21"[TIAB] OR "3-(2,4-dimethoxybenzylidene)anabaseine"[TIAB] OR Donepezil[TIAB] OR "EVP-6124"[TIAB] OR "7-chloro-N-quinuclidin-3-yl-benzo(b)thiophene-2-carboxamide"[TIAB] OR Flumazenil[TIAB] OR Galantamin*[TIAB] OR Mecamylamine[TIAB] OR "PNU 120596"[TIAB] OR "PNU120596"[TIAB] OR "1-(5-chloro-2,4-dimethoxyphenyl)-3-(5-methylisoxazol-3-yl)urea"[TIAB] OR Pregnenolone[TIAB] OR Rivastigmine[TIAB] OR "SSR180711"[TIAB] OR Varenicline[TIAB] OR "AF38469"[TIAB] OR "AR-A014418"[TIAB] OR "N-(4-methoxybenzyl)-N'-(5-nitro-1,3-thiazol-2-yl)urea"[TIAB] OR Cannabidiol[TIAB] OR Cannabidivarin[TIAB] OR Celecoxib[TIAB] OR Everolimus[TIAB] OR Fingolimod[TIAB] OR Fluconazole[TIAB] OR Glatiramer[TIAB] OR Hydroxyfasudil[TIAB] OR Lenalidomide[TIAB] OR Minocycline[TIAB] OR Naltrexone[TIAB] OR "NVP-BKM120"[TIAB] OR Buparlisib[TIAB] OR "Oral Human Immunoglobulin"[TIAB] OR Pentoxifylline[TIAB] OR "SB 216763"[TIAB] OR "SB216763"[TIAB] OR Sirolimus[TIAB] OR Rapamycin[TIAB] OR Staurosporine[TIAB] OR Suramin[TIAB] OR Tacrolimus[TIAB] OR "TAK-242"[TIAB] OR "TAK242"[TIAB] OR Temsirolimus[TIAB] OR Tideglusib[TIAB] OR "NP031112"[TIAB] OR Amastatin[TIAB] OR Angiotensin*[TIAB] OR Carnitine[TIAB] OR Levocarnitine[TIAB] OR "CM-AT"[TIAB] OR "Diet Therapy"[TIAB] OR "Diet Therapies"[TIAB] OR "Dietary Supplements"[TIAB] OR "Dietary Supplement"[TIAB] OR Dimethylglycine[TIAB] OR "EPI-743"[TIAB] OR "alpha-Tocotrienol Quinone"[TIAB] OR "Food Supplement"[TIAB] OR "Food Supplements"[TIAB] OR "Gastrin-Releasing Peptide"[TIAB] OR Ghrelin[TIAB] OR "Herbal Supplement"[TIAB] OR "Herbal Supplements"[TIAB] OR Hormone*[TIAB] OR Corticosteroid*[TIAB] OR Corticoid*[TIAB] OR Hydrocortisone[TIAB] OR "IGF-1"[TIAB] OR "Insulin-Like Growth Factor I"[TIAB] OR Lovastatin[TIAB] OR Melanocortin*[TIAB] OR Melatonin[TIAB] OR Metformin[TIAB] OR Mineral*[TIAB] OR "NNZ-2566"[TIAB] OR "NNZ2566"[TIAB] OR "Nutrition Therapy"[TIAB] OR "Nutritional Therapy"[TIAB] OR Oligosaccharide*[TIAB] OR "Omega-3"[TIAB] OR "Omega3"[TIAB] OR "n-3 Fatty"[TIAB] OR "n-3 Polyunsaturated Fatty"[TIAB] OR "n-3 PUFA"[TIAB] OR "n3 PUFA"[TIAB] OR "n 3 Oils"[TIAB] OR "n 3 Oil"[TIAB] OR "n3 Fatty"[TIAB] OR "ORG-2766"[TIAB] OR Oxytocin[TIAB] OR Syntocinon[TIAB] OR Pioglitazone[TIAB] OR Prednisone[TIAB] OR Probiotic*[TIAB] OR Bifidobacter*[TIAB] OR Pyridoxine[TIAB] OR "RG7713"[TIAB] OR "Ro27 3225"[TIAB] OR Secretin[TIAB] OR Sulforaphane[TIAB] OR Sulforafan[TIAB] OR Tetrahydrobiopterin[TIAB] OR Sapropterin[TIAB] OR Thyroxine[TIAB] OR Triiodothyronine[TIAB] OR "T3"[TIAB] OR Trofinetide[TIAB] OR Vasopressin*[TIAB] OR Vitamin*[TIAB] OR "WAY-267464"[TIAB] OR "WAY267464"[TIAB] OR Arachidonic[TIAB] OR Arachidonate[TIAB] OR Ascorbic[TIAB] OR Ascorbate[TIAB] OR Carnosine[TIAB] OR Cyanocobalamin[TIAB] OR Cobalamin*[TIAB] OR Cobamide*[TIAB] OR Hydroxocobalamin[TIAB] OR Docosahexaenoic[TIAB] OR Docosahexaenoate[TIAB] OR Ferrous[TIAB] OR Folic[TIAB] OR Folate[TIAB] OR Ginkgo*[TIAB] OR Gingko*[TIAB] OR Ginko*[TIAB] OR Maidenhair[TIAB] OR Glutathione[TIAB] OR Gluten[TIAB] OR Inositol[TIAB] OR Leucovorin[TIAB] OR Folinic[TIAB] OR Magnesium[TIAB] OR Milk[TIAB] OR Papain[TIAB] OR Pepsin[TIAB] OR Pyridoxal[TIAB] OR Pyridoxamine[TIAB] OR Succimer[TIAB] OR Dimercaptosuccinic Acid[TIAB] OR DMSA[TIAB] OR "Trichuris Suis"[TIAB] OR Ubiquinol[TIAB]) AND ("Randomized Controlled Trial"[PT] OR "Controlled Clinical Trial"[PT] OR Randomized[TIAB] OR Randomised[TIAB] OR Placebo*[TIAB] OR "Drug Therapy"[SH] OR Randomly[TIAB] OR Trial[TIAB] OR Groups[TIAB]) NOT (Animals[MH] NOT Humans[MH])

### 2.7 World Health Organization International Clinical Trials Registry Platform (WHO ICTRP)

Including:

- Australian New Zealand Clinical Trials Registry, last data file imported on 2 July 2018
- Chinese Clinical Trial Registry, last data file imported on 2 July 2018
- ClinicalTrials.gov, last data file imported on 2 July 2018
- EU Clinical Trials Register (EU-CTR), last data file imported on 25 June 2018
- ISRCTN, last data file imported on 2 July 2018
- The Netherlands National Trial Register, last data file imported on 2 July 2018
- Brazilian Clinical Trials Registry (ReBec), last data file imported on 20 June 2018
- Clinical Trials Registry - India, last data file imported on 18 June 2018
- Clinical Research Information Service - Republic of Korea, last data file imported on 18 June 2018
- Cuban Public Registry of Clinical Trials, last data file imported on 18 June 2018
- German Clinical Trials Register, last data file imported on 18 June 2018
- Iranian Registry of Clinical Trials, last data file imported on 20 June 2018
- Japan Primary Registries Network, last data file imported on 20 June 2018
- Pan African Clinical Trial Registry, last data file imported on 22 May 2018
- Sri Lanka Clinical Trials Registry, last data file imported on 18 June 2018
- Thai Clinical Trials Registry (TCTR), last data file imported on 20 June 2018
- Peruvian Clinical Trials Registry (REPEC), last data file imported on 18 June 2018

Advanced Search

Autism OR Autistic OR Asperger OR Rett OR Pervasive OR Disintegrative OR Hyperammonemia OR Hyperammonaemia in the Condition

Recruitment status is ALL

## 3. References

1. Lefebvre C, Manheimer E, Glanville J: **Chapter 6: Searching for studies**. In: *Cochrane Handbook for Systematic Reviews of Interventions.* Version 5.1.0 [updated March 2011] edn. Edited by Higgins JPT, Green S: The Cochrane Collaboration; 2011.

2. Higgins JPT, Lasserson T, Chandler J, Tovey D, Churchill R: **Methodological Expectations of Cochrane Intervention Reviews**. London: Cochrane; 2016.

3. Moher D, Liberati A, Tetzlaff J, Altman DG: **Preferred reporting items for systematic reviews and meta-analyses: the PRISMA statement**. *PLoS medicine* 2009, **6**(7):e1000097.

4. McGowan J, Sampson M, Salzwedel DM, Cogo E, Foerster V, Lefebvre C: **PRESS peer review of electronic search strategies: 2015 guideline statement**. *Journal of Clinical Epidemiology* 2016, **75**:40-46.

5. Tromans S, Adams C: **Brief Report: Autism Spectrum Disorder: A Comprehensive Survey of Randomized Controlled Trials**. *Journal of Autism and Developmental Disorders* 2018.

6. **The Yale MeSH Analyzer [Internet]** [<http://mesh.med.yale.edu/>]

7. **Pruning Emtree: does focusing Embase subject headings impact search strategy precision and sensitivity? [Internet]** [<https://www.cadth.ca/pruning-emtree-embase>]

8. **Embase animal filter** [<https://bit.ly/2IRTda1>]

9. Agarwal A, Johnston BC, Vernooij RW, Carrasco-Labra A, Brignardello-Petersen R, Neumann I, Akl EA, Sun X, Briel M, Busse JW *et al*: **Authors seldom report the most patient-important outcomes and absolute effect measures in systematic review abstracts**. *Journal of Clinical Epidemiology* 2017, **81**:3-12.

10. Chalmers I, Glasziou P: **Avoidable waste in the production and reporting of research evidence**. *Lancet (London, England)* 2009, **374**(9683):86-89.

11. Chan AW, Hrobjartsson A, Haahr MT, Gotzsche PC, Altman DG: **Empirical evidence for selective reporting of outcomes in randomized trials: comparison of protocols to published articles**. *Jama* 2004, **291**(20):2457-2465.

12. Glasziou P, Altman DG, Bossuyt P, Boutron I, Clarke M, Julious S, Michie S, Moher D, Wager E: **Reducing waste from incomplete or unusable reports of biomedical research**. *Lancet (London, England)* 2014, **383**(9913):267-276.

13. Mantziari S, Demartines N: **Poor outcome reporting in medical research; building practice on spoilt grounds**. *Annals of translational medicine* 2017, **5**(Suppl 1):S15.

# eAppendix-4 Study selection

1. Excluded records 46

1.1. Excluded records 46

1.2. References of excluded records 46

2. Eligible trials, included and ongoing 81

2.1. Included records in the systematic review 81

2.2. Ongoing trials 106

3. Contacting corresponding authors for additional data/clarifications 109

## 1. Excluded records

### 1.1. Excluded records

Wrong design (k=207):

- not randomized (k=159):^1–159^
- high risk of bias in randomization (sequence generation or allocation concealment, k=11):^160–170^
- pooled analyses (k=11):^171–181^
- placebo-discontinuation studies (k=7):^182–188^
- published before 1980 (k=19):^189–207^

Wrong participants (k=123):

- Less than 80% had ASD (k=36):^208–243^
- Inappropriate ASD diagnosis (standardized diagnostic criteria or validated diagnostic tools were not used for diagnosis, k=18):^244–261^
- ASD under anesthesia or antipsychotic-induced weight gain (combination treatment) (k=8):^262–269^
- Sample size smaller than 10 participants (including withdrawn studies, k=61): ^270–328, 460, 576^

Wrong interventions (k=235):

- Single dose interventions or less than seven days of treatments (k=117): ^329–445^
- Head-to-head or no treatment control groups (k=31): ^446–459, 461-477^
- Combination treatments (k=68): ^478–545^
- Not pharmacological or dietary supplement interventions (k=19): ^546–564^

Full-texts (protocol and abstract conferences) with unclear diagnostic criteria or study design (k=11): ^565–575^

### 1.2. References of excluded records

1. Aman M. The use of methylphenidate in autism. Journal of the American Academy of Child and Adolescent Psychiatry. 1988;27(6):821–822.

2. Acosta MT. Pharmacotherapy in autism: where to start? Drug Discovery Today. 2004;9(11):474.

3. Anonymous. Single dose secretin ’no more effective than placebo’ for autism. Pharmaceutical Journal. 2000;264(7077):8.

4. Anonymous. Children with autism may benefit from risperidone. The Pharmaceutical Journal. 2002;269(7210):184.

5. Anonymous. Autism: a new treatment seems unsuccessful. Child Health Alert. 2004;22:1–2.

6. Anonymous. Drug fails to subdue repetitive behavior in children with autism spectrum disorders. Harv Ment Health Lett. 2009;26(1057-5022 (Linking)):4.

7. Bates G, Willson SW. ’Use of selective serotonin reuptake inhibitors in children with pervasive developmental disorder: risk of treatment emergent mania’. Developmental Medicine & Child Neurology. 2003;45(5):359; author reply 360.

8. Bent S, Bertoglio K, Hendren RL. Regarding omega-3 fatty acids in severe autism. Archives of Medical Research. 2009;40(1):64; author reply 65.

9. Bou Khalil R. Would some cannabinoids ameliorate symptoms of autism? European Child and Adolescent Psychiatry. 2012;21(4):237–238.

10. Brulotte J, Bukutu C FVohra, Sunita, Vohra S. Complementary, holistic, and integrative medicine: fish oils and neurodevelopmental disorders.

11. Buitelaar JK, Willemsen-Swinkels S, Engel, H. Treatment of autism and self-injury with naltrexone. Xth world congress of psychiatry; 1996 aug 23-28; madrid, spain. 1996.

12. Buitelaar JK, Willemsen-Swinkels S, van Engel, H. Naltrexone in children with autism. Journal of the American Academy of Child and Adolescent Psychiatry. 1998;37(8):800–802.

13. Caicedo C, Williams SH. Risperidone improves behavior in children with autism. Journal of family practice. 2002;51(11):915.

14. Campbell M. The effect of neuroleptics on cognition and diagnosis, and their influence on stereotypies. Journal of Mental Deficiency Research. 1987;31:220–225.

15. Campbell M. Fenfluramine treatment of autism. Journal of Child Psychology & Psychiatry & Allied Disciplines. 1988;29(1):1–10.

16. Campbell M. Resolved: Autistic children should have a trial of naltrexone": Affirmative rebuttal. Journal of the American Academy of Child and Adolescent Psychiatry. 1996;35(2):249–250.

17. Campbell M, Harris JC. Resolved: autistic children should have a trial of naltrexone. Journal of the American Academy of Child and Adolescent Psychiatry. 1996;35(2):246-9; discussion 249-51.

18. Campbell M, Palij M. Behavioral and cognitive measures used in psychopharmacological studies of infantile autism. Psychopharmacology Bulletin. 1985;21(4):1047–1053.

19. Connors SL, Crowell DE. Secretin and autism: the role of cysteine. Journal of the American Academy of Child and Adolescent Psychiatry. 1999;38(7):795–796.

20. Corey R. Hopkins. ACS Chemical Neuroscience Molecule Spotlight on STX209 (Arbaclofen). ACS Chem Neurosci. 2011;2(8):381. doi:10.1021/cn200019z.

21. Cysneiros RM, Terra VC, Machado HR, et al. May the best friend be an enemy if not recognized early: possible role of omega-3 against cardiovascular abnormalities due to antipsychotics in the treatment of autism. Arquivos de Neuro-Psiquiatria. 2009;67(3):922–926.

22. Farber JM. Fenfluramine and autism. Developmental Medicine & Child Neurology. 1986;28(6):817–818.

23. Fenfluramine in Autism. New England Journal of Medicine. 1982;307(23):1450–1451. doi:10.1056/NEJM198212023072314.

24. Fox NS, Roman AS. Beta 2 adrenergic agents and autism. American Journal of Obstetrics & Gynecology. 2010;203(4):e15. doi:10.1016/j.ajog.2010.06.064.

25. Ghanizadeh A. Methionine sulfoximine may improve inflammation in autism, a novel hypothesized treatment for autism. Archives of Medical Research. 2010;41(8):651–652.

26. Ghanizadeh A. Ghrelin as a promising therapeutic target for co-occurring autism and epilepsy. Epilepsy & Behavior. 2011;20(2):420–421.

27. Gordon D. Early negative results not the last word on secretin/autism story. Gastroenterology. 2000;118(2):250.

28. Goulden KJ. In children with autism, is intravenous secretin more effective than placebo in improving social skills, communication, behaviour or global functioning? Part B: Clinical commentary. Paediatrics and child health;9(4):246.

29. Heisler MA, Guidry JR, McQueen JM, Heck AM. Comment: Secretin for autism: Unproven treatment or ineffective treatment? [5] (mulitiple letters). Annals of Pharmacotherapy. 2002;36(7):1294–1295.

30. Holl, er E. Translational experimental therapeutics of inflammation and fever in autism spectrum disorder: Hot tubs, locus coeruleus modulation and helminth therapy. Neuropsychopharmacology;2:S92-S93.

31. Jayach, ra S. Is secretin effective in treatment for autism spectrum disorders (ASD)? International Journal of Psychiatry in Medicine. 2005;35(1):99–101.

32. Johnson KP, Malow BA. Assessment and pharmacologic treatment of sleep disturbance in autism.

33. Johnson SM, Holl, er E. Evidence that eicosapentaenoic acid is effective in treating autism. J Clin Psychiatry;64(7):848–849.

34. Jorgensen M, Thomsen PH, Henriksen JH. [Secretin treatment of autism?]. Ugeskrift for Laeger. 2002;164(12):1676.

35. Lensing P, Klingler D, Panksepp J, et al. [Opiate hypothesis of the origin of early childhood autism and sequelae for psychopharmacotherapy]. Zeitschrift fur Kinder- und Jugendpsychiatrie. 1992;20(3):185–196.

36. Leventhal BL, Cook, Edwin H., Jr., Lord C. The irony of autism. Archives of General Psychiatry. 1998;55(7):643–644.

37. Levitas A, Zarcone JR, Hellings JA, Schroeder SR. Reader response to Zarcone et al. (2001), "Effects of risperidone on aberrant behavior in persons with developmental disabilities: I. A double-blind crossover study using multiple measures" (multiple letters). American Journal on Mental Retardation. 1;108(3):212–216.

38. Lightdale JR, Heyman MB. Secretin: cure or snake oil for autism in the new millennium? Journal of Pediatric Gastroenterology & Nutrition. 1999;29(2):114–115.

39. Linday LA. Saccharomyces boulardii: potential adjunctive treatment for children with autism and diarrhea. Journal of child neurology. 2001;16(5):387.

40. Longhurst JG, Potenza MN, McDougle CJ. Autism. New England Journal of Medicine. 1997;337(21):1555–1556.

41. Mehlinger R, Scheftner WA, Poznanski E. Fluoxetine and Autism. Journal of the American Academy of Child and Adolescent Psychiatry. 1990;29(6):985. doi:10.1097/00004583-199011000-00032.

42. Meyer-Lindenberg A. Impact of prosocial neuropeptides on human brain function. Advances in Vasopressin and Oxytocin - From Genes to Behaviour to Disease. 2008:463–470.

43. Munarriz R, Bennett L, Goldstein I. Risperidone in children with autism and serious behavioral problems. New England Journal of Medicine. 2002;347(23):1890-1; author reply 1890-1.

44. Nau JY. The effectiveness of bumetanide in the management of autism. Revue Medicale Suisse. 2017;13(556):722–723.

45. Niederhofer H. Also Topiramate might have some benefit in psychopharmacological treatment of autism.

46. Niederhofer H. Treating autism pharmacologically: also tacrine might improve symptomatology in some cases.

47. Paczynski M. Piracetam: a novel therapy for autism? Journal of Autism & Developmental Disorders. 1997;27(5):628–630.

48. Parsonson BS. Using psychoactive medication to intervene in children's behaviour: an evidence-based practice? J Prim Health Care. 2009;1(1):6–10.

49. Petryk S. In children with autism, is intravenous secretin more effective than placebo in improving social skills, communication, behaviour or global functioning? Part A: Evidence-based answer and summary. Paediatrics and child health;9(4):244–245.

50. Pretest for January 2004. CNS Spectrums. 2003;8(12):962–964. doi:10.1017/S1092852900028741.

51. Rickards EH, Prendergast M. Fluoxetine and serotonin in autism. The American Journal of Psychiatry. 1992;149(6):851.

52. Riml, B. High dose vitamin B6 and magnesium in treating autism: response to study by Findling et al. Journal of Autism & Developmental Disorders. 1998;28(6):581–582.

53. Riml, B. Secretin: real therapeutic potential (response). Journal of Pediatric Gastroenterology & Nutrition. 2000;30(2):113; author reply 113-4.

54. Said SI, Bodanszky M. Secretin treatment for autism. New England Journal of Medicine. 2000;342(16):1217–1218.

55. Stokstad E. Stalled Trial for Autism Highlights Dilemma of Alternative Treatments. Science. 2008;321(5887):326. doi:10.1126/science.321.5887.326.

56. Strayhorn J. More on methylphenidate in autism. Journal of the American Academy of Child and Adolescent Psychiatry. 1989;28(2):299.

57. Theoharides TC, Asadi S. Unwanted interactions among psychotropic drugs and other treatments for autism spectrum disorders. Journal of Clinical Psychopharmacology. 2012;32(4):437–440. doi:10.1097/JCP.0b013e31825e00e4.

58. Volkmar FR. Lessons from secretin. New England Journal of Medicine. 1999;341(24):1842–1844.

59. Wink LK, Erickson CA, Stigler KA, McDougle CJ. Riluzole in autistic disorder. Journal of Child and Adolescent Psychopharmacology. 2011;21(4):375–379. doi:10.1089/cap.2010.0154.

60. Tufan AE, Kutlu H. Adjunctive quetiapine may help depression comorbid with pervasive developmental disorders. Progress in Neuro-Psychopharmacology and Biological Psychiatry. 2009;33(8):1570–1571. doi:10.1016/j.pnpbp.2009.09.009.

61. Todd RD. Fluoxetine in autism. The American Journal of Psychiatry. 1991;148(8):1089.

62. Szabo CP, Bracken C. Imipramine and Asperger’s. Journal of the American Academy of Child and Adolescent Psychiatry. 1994;33(3):431–432.

63. Stigler KA, Erickson CA, Mullett JE, Posey DJ, McDougle CJ. Paliperidone for irritability in autistic disorder. Journal of Child & Adolescent Psychopharmacology. 2010;20(1):75–78.

64. Sporn A, Pinsker H. Use of stimulant medication in treating pervasive developmental disorder. The American Journal of Psychiatry. 1981;138(7):997.

65. Snead RW, Boon F, Presberg J. Paroxetine for self-injurious behavior. Journal of the American Academy of Child and Adolescent Psychiatry. 1994;33(6):909–910.

66. Shahani L. Use of lithium for sexual obsessions in Asperger's disorder. J Neuropsychiatry Clin Neurosci. 2012;24(4):E17. doi:10.1176/appi.neuropsych.11090232.

67. Realmuto GM, August GJ, Garfinkel BD. Clinical effect of buspirone in autistic children. Journal of Clinical Psychopharmacology. 1989;9(2):122–125.

68. Posey DI, Litwiller M, Koburn A, McDougle CJ. Paroxetine in autism. Journal of the American Academy of Child and Adolescent Psychiatry. 1999;38(2):111–112.

69. Pardini M, Guida S, Gialloreti LE. Aripiprazole treatment for coprophagia in autistic disorder. J Neuropsychiatry Clin Neurosci. 2010;22(4):451-s.e33-451.e33. doi:10.1176/jnp.2010.22.4.451.e33.

70. Ozbayrak KR. Sertraline in PDD. Journal of the American Academy of Child and Adolescent Psychiatry. 1997;36(1):7–8.

71. Niederhofer H FDamodharan, Senthil Kumar, Damodharan SK FJoji, Rekha, Joji R FCorfield, Alison, Corfield A. Atomoxetine treating patients with Autistic disorder.

72. McCracken JT, Martin W. Clonidine side effect. Journal of the American Academy of Child and Adolescent Psychiatry. 1997;36(2):160–161.

73. Malek-Ahmadi P, Simonds JF. Olanzapine for autistic disorder with hyperactivity. Journal of the American Academy of Child and Adolescent Psychiatry. 1998;37(9):902.

74. Magen J. Negative results with clomipramine. Journal of the American Academy of Child and Adolescent Psychiatry. 1993;32(5):1079–1080.

75. Kapetanovic S. Oxcarbazepine in youths with autistic disorder and significant disruptive behaviors.

76. Leboyer M, Bouvard MP, Dugas M. Effects on naltrexone on infantile autism. Lancet. 1988;1(8587):715.

77. Horrigan JP, Barnhill LJ. More on melatonin. Journal of the American Academy of Child and Adolescent Psychiatry;36(8):1014.

78. Gudarzi SS, Yasamy M, Akhondzadeh S. Cyproheptadine in treatment of autism. European Psychiatry: the Journal of the Association of European Psychiatrists. 2002;17(4):230–231.

79. Fisman S, Steele M, Pipher B. Risperidone in PDD. Journal of the American Academy of Child and Adolescent Psychiatry. 1998;37(1):15–16.

80. Erickson CA, Chambers JE. Memantine for disruptive behavior in autistic disorder. J Clin Psychiatry;67(6):1000.

81. Duggal HS. Ziprasidone for maladaptive behavior and attention-deficit/hyperactivity disorder symptoms in autistic disorder.

82. Duggal HS. Mood stabilizers in Asperger’s syndrome. Australian and New Zealand Journal of Psychiatry. 2001;35(3):390–391.

83. Albertini G, Majolini L, Di Gennaro G, Quarato P, Scoppetta C, Onorati P. Oral dyskinesia induced by fluoxetine therapy for infantile autism. Pediatric Neurology. 2004;31(1):76.

84. Alessi NE. Ziprasidone in autism. Journal of the American Academy of Child and Adolescent Psychiatry. 2003;42(6):622–623.

85. Alessi N, Alkhouri I, Fluent T, Quinlan P, Williams K. Haloperidol decanoate in children. Journal of the American Academy of Child and Adolescent Psychiatry. 2001;40(8):865–866.

86. Bernhardt EB, Walsh KH, Posey DJ, McDougle CJ. Memantine for comorbid obsessive-compulsive disorder and Asperger disorder suggests a link in glutamatergic dysregulation. Journal of Clinical Psychopharmacology. 2011;31(5):673–675.

87. Caixeta M, Caixeta L. [Topiramate reduces irritability and self-injuries in autistic children]. Revista Brasileira de Psiquiatria. 2005;27(4):345–346.

88. Craven-Thuss B, Nicolson R. Amoxapine treatment of interfering behaviors in autistic disorder. Journal of the American Academy of Child and Adolescent Psychiatry. 2003;42(5):515–516.

89. Decocq G, K, elaft N, Compagnon M. [Effects of naltrexone on automutilation behavior in autistic psychosis]. Presse Medicale. 1996;25(7):305.

90. Demb HB. Risperidone in young children with pervasive developmental disorders and other developmental disabilities. Journal of Child & Adolescent Psychopharmacology. 1996;6(1):79–80.

91. Doan RJ. Risperidone for insomnia in PDDs. Canadian Journal of Psychiatry - Revue Canadienne de Psychiatrie. 1998;43(10):1050–1051.

92. Aman MG, Armstrong SA. Regarding secretin for treating autistic disorder. Journal of Autism & Developmental Disorders. 2000;30(1):71–72.

93. Arnold LE, Aman MG, Li X, et al. Research Units of Pediatric Psychopharmacology (RUPP) autism network randomized clinical trial of parent training and medication: one-year follow-up. Journal of the American Academy of Child and Adolescent Psychiatry. 2012;51(11):1173–1184.

94. August GJ, Raz N, Baird TD. Effects of fenfluramine on behavioral, cognitive, and affective disturbances in autistic children. Journal of Autism & Developmental Disorders. 1985;15(1):97–107.

95. Awad GA. The use of selective serotonin reuptake inhibitors in young children with pervasive developmental disorders: some clinical observations. Canadian Journal of Psychiatry - Revue Canadienne de Psychiatrie. 1996;41(6):361–366. doi:10.1177/070674379604100606.

96. Bent S, Ailarov A, Dang KT, Widjaja F, Lawton BL, Hendren RL. Open-Label Trial of Vitamin D3 Supplementation in Children with Autism Spectrum Disorder. Journal of Alternative & Complementary Medicine. 2017;23(5):394–395.

97. Bent S, Lawton B, Warren T, et al. Identification of urinary metabolites that correlate with clinical improvements in children with autism treated with sulforaphane from broccoli. Molecular Autism. 2018;9:35.

98. Birmaher B, Quintana H, Greenhill LL. Methylphenidate treatment of hyperactive autistic children. Journal of the American Academy of Child and Adolescent Psychiatry. 1988;27(2):248–251.

99. Campbell M, Adams P, Small AM, Tesch LM, Curren EL. Naltrexone in infantile autism. Psychopharmacology Bulletin. 1988;24(1):135–139.

100. Campbell M, Deutsch SI, Perry R, Wolsky BB, Palij M. Short-term efficacy and safety of fenfluramine in hospitalized preschool-age autistic children: an open study. Psychopharmacology Bulletin. 1986;22(1):141–147.

101. Campbell M, Perry R, Polonsky BB, Deutsch SI, Palij M, Lukashok D. An open study of fenfluramine in hospitalized young autistic children. Journal of Autism & Developmental Disorders. 1986;16(4):495–506.

102. ChiCTR1800016113, Children’s Hospital of Chongqing Medical, University. Prospective Study of Vitamin A and Vitamin D Treatment in Children with Autism Spectrum Disorders. 2018.

103. ChiCTR1800016473, Xuanwu Hospital, Capital Medical University. The effect of improving gut microbiota for treating children with Autism Spectrum Disorder(ASD). 2018.

104. ChiCTR-CCC-13004498, Bethune First Hospital of Jilin, University. The associate of polymorphisms of vitamin D metabolism-related genes with autism, and the treatment of autism with vitamin D. 2013.

105. ChiCTR-ROC-14005442, Children’s Hospital, Chongqing Medical University. The roles of vitamin A and its nuclear receptors in the pathogenesis of autism spectrum disorder. 2014.

106. CTRI/2018/06/014379, All India Institute of, Ayurveda. Effect of Abhaya Ghrita and Panchabhautika Taila Nasya in treating Autism Spectrum Disorders in children. 2018.

107. Desousa A. An Open-label Trial of Risperidone and Fluoxetine in Children with Autistic Disorder. Indian Journal of Psychological Medicine. 2010;32(1):17–21.

108. Deutsch SI, Milstoc M, Platovsky G, Wolsky BB, Perry R, Green WH. Cholinesterase activities in blood in infantile autism. Biological Psychiatry. 1987;22(2):234–236.

109. Erickson CA, Ray B, Maloney B, et al. Impact of acamprosate on plasma amyloid-beta precursor protein in youth: a pilot analysis in fragile X syndrome-associated and idiopathic autism spectrum disorder suggests a pharmacodynamic protein marker. Journal of Psychiatric Research. 2014;59:220–228.

110. Erickson CA, Wink LK, Early MC, et al. Brief report: pilot single-blind placebo lead-in study of acamprosate in youth with autistic disorder. Journal of autism and developmental disorders. 2014;44(4):981–987.

111. EUCTR2012-001616-33-GB, Forest Research Institute I. An Open-Label Study Of The Safety And Tolerability Of Memantine In Pediatric Patients With Autism, Asperger’s Disorder, Or Pervasive Developmental Disorder Not Otherwise Specified (PDD-NOS). 2012.

112. Frye RE, Sequeira JM, Quadros EV, James SJ, Rossignol DA. Cerebral folate receptor autoantibodies in autism spectrum disorder. Molecular Psychiatry. 2013;18(3):369–381.

113. Geier DA, Geier MR. A clinical trial of combined anti-androgen and anti-heavy metal therapy in autistic disorders. Neuroendocrinology Letters. 2006;27(6):833–838.

114. Ghaziuddin M, Tsai L, Ghaziuddin N. Fluoxetine in autism with depression. Journal of the American Academy of Child and Adolescent Psychiatry. 1991;30(3):508–509.

115. Gupta S. Treatment of children with autism with intravenous immunoglobulin. Journal of child neurology. 1999;14(3):203–205.

116. Gvozdjakova A, Kucharska J, Ostatnikova D, Babinska K, Nakladal D, Crane FL. Ubiquinol improves symptoms in children with autism. Oxidative medicine & cellular longevity. 2014;2014:798957.

117. IRCT20130504013215N2, Shahid Beheshti University of Medical, Sciences. Probiotics Effect in Reducing Behavioral Symptoms and Severity of Autism. 2018.

118. IRCT2015100424337N1, Vice chancellor for research, Tehran University of Medical Sciences. Cerebrolysin effect On cognitive and verbal aspects of children suffering from autism spectrum disorder. 2015.

119. James SJ, Cutler P, Melnyk S, et al. Metabolic biomarkers of increased oxidative stress and impaired methylation capacity in children with autism. American Journal of Clinical Nutrition. 2004;80(6):1611–1617.

120. Joshi G, Biederman J, Wozniak J, et al. Response to second generation antipsychotics in youth with comorbid bipolar disorder and autism spectrum disorder. CNS Neuroscience & Therapeutics. 2012;18(1):28–33.

121. JPRN-UMIN000006558, Department of Neuropsychiatry, Faculty of Medical Sciences University of Fukui. A research of efficacy and safety of aripiprazole treatment for the behavioral symptoms in subjects with pervasive developmental disordes. 2011.

122. JPRN-UMIN000016770, Environmental M, Council for N. Vitamin D Status in Autism Spectrum Disorder and the Efficacy of Vitamin D Supplementation in Autistic Children. 2015.

123. JPRN-UMIN000021433, Assiut u. Ketogenic diet versus gluten free casein free diet in autistic children: a case-control study. 2016.

124. Julie Hess, Johnny Matson, Daniene Neal, et al. A Comparison of Psychotropic Drug Side Effect Profiles in Adults Diagnosed With Intellectual Disabilities and Autism Spectrum Disorders. Journal of Mental Health Research in Intellectual Disabilities. 2010;3(2):85–96. doi:10.1080/19315861003690588.

125. Kaluzna-Czaplinska J, Jozwik-Pruska J, Chirumbolo S, Bjorklund G. Tryptophan status in autism spectrum disorder and the influence of supplementation on its level. Metabolic Brain Disease. 2017;32(5):1585–1593.

126. Kaluzna-Czaplinska J, Michalska M, Rynkowski J. Vitamin supplementation reduces the level of homocysteine in the urine of autistic children. Nutrition Research. 2011;31(4):318–321.

127. Kaluzna-Czaplinska J, Socha E, Rynkowski J. B vitamin supplementation reduces excretion of urinary dicarboxylic acids in autistic children. Nutrition Research. 2011;31(7):497–502.

128. Legido A, Goldenthal M, Garvin B, et al. Effect of a combination of carnitine, coenzyme q10 and alpha-lipoic acid (mitococktail) on mitochondrial function and neurobehavioral performance in children with autism spectrum disorder. Neurology. Conference: 70th Annual Meeting of the American Academy of Neurology, AAN. 2018;90(15).

129. Martineau J, Barthelemy C, Jouve J, Muh J-P, LeLord G. Monoamines (serotonin and catecholamines) and their derivatives in infantile autism: Age-related changes and drug effects. Developmental Medicine & Child Neurology. 1992;34(7):593–603.

130. Martsenkovsky I. Open-label atomoxetine for attention-deficit/ hyperactivity disorder symptoms associated with high-functioning autism spectrum disorders. European Child and Adolescent Psychiatry;1:S221.

131. Max Horovitz, Johnny L. Matson, Alyse Barker. The relationship between symptoms of autism spectrum disorders and psychotropic medication use in infants and toddlers. Research in autism spectrum disorders. 2012;6(4):1406–1411. doi:10.1016/j.rasd.2011.05.013.

132. Martsenkovsky I, Bikshaieva I, Vashenko O, Martsenkovsky D. Memantine therapy of cognitive, behavioral, and social dysfunction in children witch autism spectrum disorders (ASD). International Journal of Neuropsychopharmacology;1:138.

133. Meguid NA, Hashish AF, Anwar M, Sidhom G. Reduced serum levels of 25-hydroxy and 1,25-dihydroxy vitamin D in Egyptian children with autism. Journal of alternative and complementary medicine (new york, N.Y.). 2010;16(6):641–645. doi:10.1089/acm.2009.0349.

134. Milin R, Simeon JG, Batth S, Thatte S, Dare GJ, Walker S. An open trial of olanzapine in children and adolescents with Asperger Disorder. Journal of Clinical Psychopharmacology. 2006;26(1):90–92.

135. Min Guo, Jiang Zhu, Ting Yang, et al. Vitamin A and vitamin D deficiencies exacerbate symptoms in children with autism spectrum disorders. Nutritional neuroscience. 2018;0(0):1–11. doi:10.1080/1028415X.2017.1423268.

136. Minderaa RB FAnderson, G M, Anderson GM FVolkmar, F R, Volkmar FR FAkkerhuis, G W, Akkerhuis GW FCohen, D J, Cohen DJ. Urinary 5-hydroxyindoleacetic acid and whole blood serotonin and tryptophan in autistic and normal subjects.

137. NCT00325572, Thrasher Research F, Penn State U. Evaluation and Treatment of Copper/Zinc Imbalance in Children With Autism. 2006.

138. NCT00549562, Ortho-McNeil Janssen Scientific Affairs, L. L. C., Indiana University School of, Medicine. Study of Paliperidone ER in Adolescents and Young Adults With Autism. 2007.

139. NCT00619190, Bristol-Myers S, University of North Carolina, Chapel Hill. Study of Aripiprazole to Treat Children and Adolescents With Autism. 2008.

140. NCT01050582, amp, Johnson Pharmaceutical R, Development LLC, Johnson. A Study to Evaluate the Safety and the Effects of Risperidone Compared With Other Atypical Antipsychotic Drugs on the Growth and Sexual Maturation in Children. 2010.

141. NCT01205282, Holl, Bloorview Kids Rehabilitation H, Evdokia A. Dose Finding Study of Pioglitazone in Children With Autism Spectrum Disorders (ASD) (PIO). 2010.

142. NCT01352611, University of M-C. Open Label Treatment of Severe Tactile Defensiveness With Intrathecal Baclofen. 2011.

143. NCT01731119, Foundation of Hope, North Carolina, University of North Carolina, Chapel Hill. Study of Lurasidone in Treating Antipsychotic Naive or Quasi-Naive Children and Adolescents. 2012.

144. NCT01881737, Stanford U. A Study of Pregnenolone in the Treatment of Individuals With Autism. 2011.

145. NCT03432065, Massachusetts General H. A Pilot Study of Buspirone for the Treatment of Anxiety in Youth With Autism Spectrum Disorders. 2018.

146. Perry R, Bangaru BS. Secretin in autism. Journal of Child & Adolescent Psychopharmacology. 1998;8(4):247–248.

147. Perry R, Campbell M, Green WH, et al. Neuroleptic-related dyskinesias in autistic children: a prospective study. Psychopharmacology Bulletin. 1985;21(1):140–143.

148. Plioplys AV. Intravenous immunoglobulin treatment in autism. Journal of Autism & Developmental Disorders. 2000;30(1):73–74.

149. Ritvo ER, Freeman BJ, Yuwiler A, et al. Study of fenfluramine in outpatients with the syndrome of autism. Journal of pediatrics. 1984;105(5):823–828.

150. Simon-Soret C, Borenstein P. [A trial of bromocriptine in the treatment of infantile autism]. Presse Medicale. 1987;16(26):1286.

151. Stubbs EG, Budden SS, Jackson RH, Terdal LG, Ritvo ER. Effects of fenfluramine on eight outpatients with the syndrome of autism. Developmental medicine and child neurology. 1986;28(2):229–235.

152. Tachibana M, Kagitani-Shimono K, Mohri I, et al. Long-term administration of intranasal oxytocin to early adolescents with autistic spectrum disorder. Developmental medicine and child neurology. 2012;54:66.

153. Valdovinos MG, Bailey L, Taylor SL. Examining risperidone use in those diagnosed with autism 1 year after FDA approval. J Clin Psychiatry. 2010;71(5):651–652.

154. Zeiner P, Gjevik E, Weidle B. Response to atomoxetine in boys with high-functioning autism spectrum disorders and attention deficit/hyperactivity disorder. Acta Paediatr. 2011;100(9):1258–1261. doi:10.1111/j.1651-2227.2011.02263.x.

155. Ritvo ER, Freeman BJ, Yuwiler A, et al. Fenfluramine treatment of autism: UCLA collaborative study of 81 patients at nine medical centers. Psychopharmacology Bulletin. 1986;22(1):133–140.

156. Malow BA, Adkins KW, McGrew SG, Surdyka K, Goldman SE, Wofford D. Impact of supplemental melatonin on sleep and behavior in children with autism spectrum disorders. Sleep. 2009:A64.

157. Malow BA, Adkins KW, McGrew SG, Surdyka K, Wofford D. Supplemental melatonin improves sleep in children with autism spectrum disorders. Annals of Neurology. 2009;1:S31.

158. Malow B, Adkins KW, McGrew SG, et al. Melatonin for sleep in children with autism: a controlled trial examining dose, tolerability, and outcomes. Journal of autism and developmental disorders. 2012;42(8):1729-37; author reply 1738.

159. NCT00927030, Eunice Kennedy Shriver National Institute of Child, Health, Human D, V, erbilt U. Melatonin for Sleep in Children With Autism. 2009.

160. Chez MG, Buchanan CP, Aimonovitch MC, et al. Double-blind, placebo-controlled study of L-carnosine supplementation in children with autistic spectrum disorders. Journal of child neurology. 2002;17(11):833–837.

161. Buitelaar JK, Dekker ME, Ree JM, Engel, H. A controlled trial with ORG 2766, an ACTH-(4-9) analog, in 50 relatively able children with autism. European neuropsychopharmacology. 1996;6(1):13–19.

162. Verbaten MN, Kemner C, Buitelaar JK, et al. Effects of ORG-2766 on brain event-related potentials of autistic children. Psychiatry research. 1996;63(1):33–45.

163. Klykylo WM, Feldis D, O’Grady D, Ross DL, Halloran C. Clinical effects of fenfluramine in ten autistic subjects. Journal of Autism & Developmental Disorders. 1985;15(4):417–423.

164. NCT01962870, National Institute of Mental, Health, Stanford U. The Role of Vasopressin in the Social Deficits of Autism. 2013.

165. Parker K, Oztan O, Libove R, et al. Intranasal vasopressin treatment improves social abilities in children with Autism. Neuropsychopharmacology. Conference: 55th annual meeting of the american college of neuropsychopharmacology, ACNP 2016. United states. Conference start: 20161204. Conference end: 20161208. 2016;41:S341.

166. Parker KJ, Oztan O, Libove RA, et al. A randomized placebo-controlled pilot trial shows that intranasal vasopressin improves social deficits in children with autism. Sci Transl Med. 2019;11(491).

167. Luby J, Mrakotsky C, Stalets MM, et al. Risperidone in preschool children with autistic spectrum disorders: an investigation of safety and efficacy. Journal of Child & Adolescent Psychopharmacology. 2006;16(5):575–587.

168. Luby JL, Mrakotsky C, Stalets MM, et al. Risperidone in preschool children with autistic spectrum disorders: An investigation of safety and efficacy. Luby, Joan L [Ed]. 2009.

169. NCT00374764, Washington University School of, Medicine. Comparison of Applied Behavioral Analysis (ABA) Versus ABA and Risperidone. 2006.

170. Chez MG, Buchanan TM, Becker M, Kessler J, Aimonovitch MC, Mrazek SR. Donepezil hydrochloride: A double-blind study in autistic children. Journal of Pediatric Neurology. 2003;1(2):83–88.

171. Aman MG, Kasper W, Manos G, et al. Line-item analysis of the Aberrant Behavior Checklist: results from two studies of aripiprazole in the treatment of irritability associated with autistic disorder. Journal of Child & Adolescent Psychopharmacology. 2010;20(5):415–422.

172. Calarge CA, Ziegler EE, Castillo N, et al. Iron homeostasis during risperidone treatment in children and adolescents. Journal of clinical psychiatry. 2015;76(11):1500–1505.

173. Ernst M, Devi L, Silva RR, et al. Plasma beta-endorphin levels, naltrexone, and haloperidol in autistic children. Psychopharmacology Bulletin. 1993;29(2):221–227.

174. L, sberg W, Loze JY, et al. Safety and tolerability of aripiprazole in the treatment of irritability associated with autistic disorder in pediatric patients: Results from a 52-week open-label study. European Psychiatry. 2011;26.

175. Lewis D, Owen R, Couch DM. Efficacy and safety of aripiprazole for the treatment of irritability associated with autistic disorder in children and adolescents (6-17 years): results from two 8-week, randomized, double-blind, placebo-controlled trials. Neurology. 2009;72(11):A428, Abstract no: S50.005.

176. Locascio JJ, Malone RP, Small AM, et al. Factors related to haloperidol response and dyskinesias in autistic children. Psychopharmacology Bulletin. 1991;27(2):119–126.

177. NCT00211770, Mount Sinai School of, Medicine. Use of Functional Behavioral Assessments to Evaluate Stereotypy and Repetitive Behaviors in a Double-blind, Placebo Controlled Trials of Various Medications Used to Treat Children With Autism. 2005.

178. NCT00399698, Ohio State U. Study to Determine Whether There Are Any Cognitive or Motor Effects From Taking the Medicine Risperidone. 2006.

179. Owada K, Okada T, Munesue T, et al. Quantitative facial expression analysis revealed the efficacy and time course of oxytocin in autism. Brain. 2019.

180. Robb AS, Andersson C, Bellocchio EE, et al. Safety and tolerability of aripiprazole in the treatment of irritability associated with autistic disorder in pediatric subjects (6-17 years Old): results from a pooled analysis of 2 studies. Primary care companion to the journal of clinical psychiatry. 2011;13(1):e1-e9.

181. Varni JW, H, en BL, et al. Effect of Aripiprazole 2 to 15 mg/d on Health-Related Quality of Life in the Treatment of Irritability Associated with Autistic Disorder in Children: a Post Hoc Analysis of Two Controlled Trials. Clinical therapeutics. 2012;34(4):980–992.

182. EUCTR2006-005346-37-NL, Company L, Eli L. A Randomized, Double-Blind, Placebo-Controlled Maintenance of Effect Study of Olanzapine in the Treatment of Disruptive Behavioral Symptoms in Children and Adolescents with Pervasive Developmental Disorders - HGMR. 2006.

183. Dolske MC, Spollen J, McKay S, Lancashire E, Tolbert L. A preliminary trial of ascorbic acid as supplemental therapy for autism. Progress in neuro-psychopharmacology & biological psychiatry. 1993;17(5):765–774.

184. Findling RL, Mankoski R, Timko K, et al. A randomized controlled trial investigating the safety and efficacy of aripiprazole in the long-term maintenance treatment of pediatric patients with irritability associated with autistic disorder. Journal of clinical psychiatry. 2014;75(1):22–30.

185. NCT01227668. Phase IV Long-term Maintenance Study of Aripiprazole in the Treatment of Irritability Associated With Autistic Disorder.

186. NTR294, Adolescent P, Accare, Division University Center for Child. Risperidone in Children and Adolescents with severe disruptive behavior problems. 2005.

187. Tolbert L, Haigler T, Waits MM, Dennis T. Brief report: lack of response in an autistic population to a low dose clinical trial of pyridoxine plus magnesium. Journal of autism and developmental disorders. 1993;23(1):193–199.

188. EUCTR2012-001568-31-GB, Forest Research Institute I. A Double-Blind, Placebo-Controlled, Randomized Withdrawal Study of the Safety and Efficacy of Memantine in Pediatric Patients with Autism, Asperger’s Disorder, or Pervasive Developmental Disorder Not Otherwise Specified (PDD-NOS) Previously Treated with Memantine. 2012.

189. Bonisch E. [Experiences with pyrithioxin in brain-damaged children with autistic syndrome]. Praxis der Kinderpsychologie und Kinderpsychiatrie. 1968;17(8):308–310.

190. Bruce AG. Lucidril for autism. New Zealand Medical Journal. 1971;73(466):173.

191. Buck RP de. Antiautistic effect of flupentixol. Acta Psychiatrica Belgica. 1974;74(5):520–525.

192. Campbell M, Fish B, Korein J, Shapiro T, Collins P, Koh C. Lithium and chlorpromazine: a controlled crossover study of hyperactive severely disturbed young children. Journal of autism and childhood schizophrenia. 1972;2(3):234–263.

193. Campbell M, Small AM, Holl, et al. A controlled crossover study of triiodothyronine in autistic children. Journal of autism and childhood schizophrenia. 1978;8(4):371–381.

194. Collard J. [Sulpiride, an unusual antiautistic and thymanaleptic neuroleptic agent]. Therapeutique. 1970;46(5):503–506.

195. Collard J, Dufrasne M, Fraipont J. The place of sulpiride (Dogmatil) in chemotherapy of autism. Acta Psychiatrica Belgica. 1971;71(1):42–55.

196. Collard J, Fraipont J, Dufrasne M. [Sulpiride, autism and course under MMPI]. Lille Medical. 1972;17:Suppl 1:33-6.

197. Hoshino Y, Yashima Y, Ishige K, Kaneko M, Kumashiro H. Effects of small doses of haloperidol on autistic children. Fukushima Journal of Medical Science. 1979;26(1):43–54.

198. Kehrer HE. [Infantile autism and drug therapy]. Bibliotheca Psychiatrica. 1978(157):91–97.

199. Kurtis LB. Clinical study of the response to nortriptyline on autistic children. International Journal of Neuropsychiatry. 1966;2(4):298–301.

200. Luyckx A. [Clinical experimentation with a long-acting neuroleptic fluspirilene (R 6218)]. Acta Psychiatrica Belgica. 1972;72(6):748–755.

201. Miller B, Wallis H. The mode of action of sulpiride in autistic children. A double blind study. [German] Uber Die Wirkungsweise Von Sulpirid Bei Autistischen Kindern. Eine Doppelblinduntersuchungen. Munchener Medizinische Wochenschrift. 1979;121(19):667–669.

202. Moss N, Boverman H. Megavitamin therapy for autistic children. The American Journal of Psychiatry. 1978;135(11):1425–1426.

203. Rimland B., Callaway E, Dreyfus P. The effect of high doses of vitamin B6 on autistic children: a double-blind crossover study. American journal of psychiatry. 1978;135(4):472–475.

204. Ritvo ER, Yuwiler A, Geller E, et al. Effects of L-dopa in autism. Journal of autism and developmental disorders. 1971;1(2):190–205. doi:10.1007/BF01537957.

205. Simmons JQ, Leiken SJ, Lovaas OI, Schaeffer B, Perloff B. Modification of autistic behavior with LSD-25. The American Journal of Psychiatry. 1966;122(11):1201–1211.

206. Campbell M, Anderson LT, Meier M, et al. A comparison of haloperidol and behavior therapy and their interaction in autistic children. Journal of the American Academy of Child Psychiatry. 1978;17(4):640–655.

207. Campbell M, Anderson LT, Meier M. A comparison of haloperidol, behavior therapy, and their interaction in autistic children [proceedings]. Psychopharmacology Bulletin. 1979;15(2):84–86.

208. Crewther D, Bauer I, Crewther S, Pipingas A. Non-linear visual evoked potentials - A sensitive assay for nutraceutical effects. Clinical EEG and Neuroscience. 2011;42:127–128.

209. Dager SR, Corrigan NM, Richards T, Dunner D, Lyoo IK, Renshaw PF. Imaging brain metabolism: evidence for altered brain bioenergetics in bipolar disorder. Bipolar disorders. 2014;16:25.

210. Feng C, Hackett PD, DeMarco AC, et al. Oxytocin and vasopressin effects on the neural response to social cooperation are modulated by sex in humans. Brain Imaging and Behavior. 2015;9(4):754–764.

211. Goldstein R, Joja O, Psatta DM, Petrescu M, Paraschiv I, Popa M. Vasotocin improves intelligence and attention in mentally retarded children. Physiology & behavior. 1989;46(6):967–970.

212. H, en BL, Sahl R, Hardan AY. Guanfacine in children with autism and/or intellectual disabilities. Journal of developmental and behavioral pediatrics. 2008;29(4):303–308.

213. Mancini J, Dubus JC, Jouve E, et al. Effect of desipramine on patients with breathing disorders in RETT syndrome. Annals of Clinical and Translational Neurology. 2018;5(2):118–127.

214. NCT00205699, National Institute of Mental, Health, Washington University School of, Medicine. Metabolic Effects of Antipsychotics in Children. 2005.

215. NCT02149823, James, J. Peters Veterans Affairs Medical Center, Visn 3 Mental Illness Research, Education, Clinical C, Maria de las Mercedes Perez, Rodriguez. Examining Dose-Related Effects of Oxytocin on Social Cognition Across Populations. 2014.

216. SLCTR/2009/006, Habib J. Effectiveness of Omega-3 and Omega-6 in childhood behaviour disorders. 2009.

217. Appleton RE, Gringras P. Mends: The use of melatonin in children with neuro-developmental disorders and impaired sleep-A randomised, double-blind, placebocontrolled, parallel trial. Archives of disease in childhood;1:A1.

218. Uillemsen Svinkels, S. Kh N., Bautelaar Ia K, Neikhof GI, Engel, Kh. Failure of naltrexone hydrochloride to reduce self-injurious and autistic behavior in mentally retarded adults: double-blind placebo-controlled studies. Sotsialnaia I klinicheskaia psikhiatriia. 1997:63–74.

219. Willemsen-Swinkels SH, Buitelaar JK, Nijhof GJ, Engl, H. Failure of naltrexone hydrochloride to reduce self-injurious and autistic behavior in mentally retarded adults. Double-blind placebo-controlled studies. Archives of General Psychiatry. 1995;52(9):766–773.

220. Boone KM, Gracious B, Klebanoff MA, et al. Omega-3 and -6 fatty acid supplementation and sensory processing in toddlers with ASD symptomology born preterm: A randomized controlled trial.[Erratum appears in Early Hum Dev. 2018 Mar 2;:; PMID: 29506901]. Early Human Development. 2017;115:64–70.

221. Keim SA, Gracious B, Boone KM, et al. omega-3 and omega-6 Fatty Acid Supplementation May Reduce Autism Symptoms Based on Parent Report in Preterm Toddlers. Journal of Nutrition. 2018;148(2):227–235.

222. Sheppard KW, Boone KM, Gracious B, et al. Effect of Omega-3 and -6 Supplementation on Language in Preterm Toddlers Exhibiting Autism Spectrum Disorder Symptoms. Journal of autism and developmental disorders. 2017;47(11):3358–3369.

223. Wasdell MB, Jan JE, Bomben MM, et al. A randomized, placebo-controlled trial of controlled release melatonin treatment of delayed sleep phase syndrome and impaired sleep maintenance in children with neurodevelopmental disabilities. Journal of pineal research. 2008;44(1):57–64.

224. Simonoff E, Taylor E, Baird G, Bernard S. Commentary: RCT of optimal dose methylphenidate in children and adolescents with severe ADHD and ID‐a reply to Arnold (2013). Journal of Child Psychology & Psychiatry & Allied Disciplines. 2013;54(6):703–704.

225. Simonoff E, Taylor E, Baird G, et al. Randomized controlled double-blind trial of optimal dose methylphenidate in children and adolescents with severe attention deficit hyperactivity disorder and intellectual disability. Journal of child psychology and psychiatry, and allied disciplines. 2013;54(5):527–535.

226. Joseph L, Grant P, Swedo S. A placebo-controlled trial of riluzole for treatment of childhood-onset obsessive compulsive disorder. Neuropsychopharmacology. 2011;36:S228-s229.

227. NCT00251303, National Institute of Mental, Health. Riluzole to Treat Child and Adolescent Obsessive-Compulsive Disorder With or Without Autism Spectrum Disorders. 2005.

228. Sandman CA. Opiate Control of Self-Injury in Mental Retardation. 8th european college of neuropsychopharmacology congress. 1995.

229. Sandman CA. Opiate Control of Self-Injury in Mental Retardation CONFERENCE ABSTRACT. 8th european college of neuropsychopharmacology congress. Venice, italy. 30th september - 4th october, 1995. 1995.

230. Sandman CA, Hetrick W, Talyor D, Marion S, Chicz-Demet A. Uncoupling of proopiomelanocortin (POMC) fragments is related to self- injury. Peptides. 2000;21(6):785–791.

231. Tyrer P, Oliver-Africano P, Romeo R, et al. Neuroleptics in the treatment of aggressive challenging behaviour for people with intellectual disabilities: A randomised controlled trial (NACHBID). Health Technology Assessment;13(21):1–54.

232. Overwater IE, Rietman AB, Mous SE, et al. A randomized controlled trial with everolimus for IQ and autism in tuberous sclerosis complex. Neurology. Conference: 65th American Academy of Neurology Annual Meeting. San Diego, CA United States. Conference Publication. 2019.

233. CTRI/2017/01/007738, Newron Pharmaceuticals Sp A. A 6 Month Study to Evaluate the Efficacy, Safety and Tolerability of drug â??Sarizotanâ?? in Patients with Rett Syndrome having difficulty in breathing. This is a Randomized, Double-Blind (the doctor and the patient not knowing the treatment), Placebo (inactive substance) -Controlled study. 2017.

234. Diego-Otero Y de, Calvo-Medina R, Quintero-Navarro C, et al. A combination of ascorbic acid and alpha-tocopherol to test the effectiveness and safety in the fragile X syndrome: study protocol for a phase II, randomized, placebo-controlled trial. Trials;15:345.

235. NCT03569631. A 2-Period Crossover Study of BPN14770 in Adults Males With Fragile X Syndrome. https://ClinicalTrials.gov/show/NCT03569631.

236. O’Leary HM, Kaufmann WE, Barnes KV, et al. Placebo-controlled crossover assessment of mecasermin for the treatment of Rett syndrome. Annals of Clinical and Translational Neurology;5(3):323–332.

237. Percy A, Glaze D, Neul J, et al. Trofinetide, a novel IGF-1 related treatment for neurodevelopmental disorders, demonstrates efficacy for children and adolescents with rett syndrome. Annals of Neurology;82:S342-S343.

238. Percy A, Glaze DG, Neul JL, et al. Trofinetide, a novel IGF-1 related treatment for neurodevelopmental disorders, demonstrates efficacy for children and adolescents with Rett syndrome. Journal of the american academy of child and adolescent psychiatry. Conference: 64th annual meeting american academy of child and adolescent psychiatry, AACAP 2017. United states. 2017;56(10):S168-s169.

239. Sahu JK, Gulati S, Sapra S, et al. Effectiveness and safety of donepezil in boys with fragile x syndrome: a double-blind, randomized, controlled pilot study. Journal of child neurology. 2013;28(5):570–575.

240. Smith-Hicks CL, Gupta S, Ewen JB, et al. Randomized open-label trial of dextromethorphan in Rett syndrome. Neurology. 2017;89(16):1684–1690.

241. Siper P, Tavassoli T, George-Jones J, et al. The sensory domain as a target for treatment in ASD clinical trials: Electrophysiological and behavioral markers of therapeutic change. Biological Psychiatry;83:S370.

242. Al Olaby RR, Hagerman R, Abbeduto L, Tassone F. Identification of molecular biomarkers predictive of response to targeted treatment in fragile X syndrome and autism spectrum disorder. Journal of Intellectual Disability Research;61:828.

243. Greiss Hess L, Fitzpatrick SE, Nguyen DV, et al. A Randomized, Double-Blind, Placebo-Controlled Trial of Low-Dose Sertraline in Young Children With Fragile X Syndrome. Journal of developmental and behavioral pediatrics. 2016;37(8):619–628.

244. Jung SS, Lee YC. A double blind study of dimethylglycine treatment in children with autism. [Korean]. Tzu Chi Medical Journal. 2000;12(2):111–121.

245. Grimaldi R, Gibson GR, Vulevic J, et al. A prebiotic intervention study in children with autism spectrum disorders (ASDs). Microbiome. 2018;6(1):133.

246. NCT02720900, University of R, Clasado L. Prebiotic Intervention for Autism Spectrum Disorders. 2015.

247. Naruse H, Hayashi T, Takesada M, Nakane A, Yamazaki K. Metabolic changes in aromatic amino acids and monoamines in infantile autism and development of new treatment related to the finding. No to hattatsu = brain and development. 1989;21(2):181–189.

248. Naruse H, Takesada M, Nakane Y, et al. Clinical Evaluation of R-Tetrahydrobiopterin (SUN 0588) on Infantile Autism: a Double-Blind Comparative Study Using Placebo as a Control. Rinsho iyaku (journal of clinical therapeutics and medicines). 1990;6(7):1343–1368.

249. A multi centered double blind trial of pimozide (Orap), haloperidol and placebo for abnormal behavior in children using crossover design. Rinsho hyoka /clinical evaluation. 1980;8(3):629–673.

250. Naruse H, Nagahata M, Nakane Y. A multi-center double-blind trial of pimozide (Orap), haloperidol and placebo in children with behavioral disorders, using crossover design. Acta Paedopsychiatrica. 1982;48(4):173–184.

251. Moorthy MP, Srinivasan AV, Bhanu K, Mugundan K, Sivakumar S. L-Carnosine in pediatric cognitive disorders. Neurorehabilitation and Neural Repair;32:372–373.

252. Parracho, H. M. R. T., Gibson GR, Knott F, Bosscher D, Kleerebezem M, McCartney AL. A double-blind, placebo-controlled, crossover-designed probiotic feeding study in children diagnosed with autistic spectrum disorders. International Journal of Probiotics and Prebiotics. 2010;5(2):69–74.

253. Kohler JA, Shortl, G., Rolles CJ. Effect of fenfluramine on autistic symptoms. British medical journal (clinical research ed.). 1987;295(6603):885.

254. Adams JB, Holloway C. Pilot study of a moderate dose multivitamin/mineral supplement for children with autistic spectrum disorder.[Erratum appears in J Altern Complement Med. 2005 Aug;11(4):749]. Journal of Alternative & Complementary Medicine. 2004;10(6):1033–1039.

255. Adams JB, Audhya T, McDonough-Means S, et al. Effect of a vitamin/mineral supplement on children and adults with autism. BMC pediatrics. 2011;11:111.

256. NCT01225198, Autism Research I, Legacy F, Arizona State U. Vitamin/Mineral Supplement for Children and Adults With Autism. 2010.

257. Barthelemy C, Garreau B, Leddet I, et al. Relevance of behavior scales and dosage levels of homovanillic acid in the urine to controlling the effects of a treatment combining vitamin B6 and magnesium administered to children with autistic behavior. [French] Interet des echelles de comportement et des dosages de l’acide homovanilique urinaire pour le controle des effets d’un traitement associant vitamine B6 et magnesium chez des enfants ayant un comportement autistique. Neuropsychiatrie de l’Enfance et de l’Adolescence. 1983;31(5):289–301.

258. Barthelemy C, Garreau B, Leddet I, et al. Value of behavior scales and urinary homovanillic acid determinations in monitoring the combined treatment with vitamin B6 and magnesium of children displaying autistic behavior. Neuropsychiatrie de l’Enfance et de l’Adolescence. 1983;31(5):289–301.

259. Lelord G, Callaway E, Muh JP. Clinical and biological effects of high doses of vitamin B6 and magnesium on autistic children. Acta vitaminologica ET enzymologica. 1982;4(1):27–44.

260. Lelord G, Muh JP, Barthelemy C, Martineau J, Garreau B, Callaway E. Effects of pyridoxine and magnesium on autistic symptoms: Initial observations. Journal of Autism & Developmental Disorders. 1981;11(2):219–230.

261. Garstang J, Wallis M. Randomized controlled trial of melatonin for children with autistic spectrum disorders and sleep problems. Child Care Health Dev. 2006;32(5):585–589.

262. NCT00709202, Stanley Medical Research I, Nathan Kline Institute for Psychiatric, Research. Efficacy and Tolerability Study of Betahistine to Ameliorate Antipsychotic Associated Weight Gain in Adolescents and Young Adults. 2008.

263. Reeves GM, Keeton C, Correll CU, et al. Improving metabolic parameters of antipsychotic child treatment (IMPACT) study: rationale, design, and methods. Child Adolesc Psychiatry Ment Health;7(1):31.

264. Aman MG, Hollway JA, Veenstra-V, et al. Effects of Metformin on Spatial and Verbal Memory in Children with ASD and Overweight Associated with Atypical Antipsychotic Use. Journal of Child and Adolescent Psychopharmacology. 2018;28(4):266–273.

265. Anagnostou E, Aman MG, H, et al. Metformin for Treatment of Overweight Induced by Atypical Antipsychotic Medication in Young People With Autism Spectrum Disorder: a Randomized Clinical Trial. JAMA psychiatry. 2016;73(9):928–937.

266. Anonymous. Erratum: metformin for treatment of overweight induced by atypical antipsychotic medication in young people with autism spectrum disorder: a randomized clinical trial (JAMA Psychiatry (2016) 73: 9 (928-937)). JAMA psychiatry. 2016;73(12):1295.

267. NCT01825798, Massachusetts General H, V, et al. Treatment of Overweight Induced by Antipsychotic Medication in Young People With Autism Spectrum Disorders (ASD). 2013.

268. Larr AS, Vakhrusheva J, Marino P, Maayan L. A double-blind, placebo controlled trial of betahistine to ameliorate antipsychotic associated weight gain in adolescents and young adults: Preliminary safety and efficacy data. Schizophrenia Research;1:S355.

269. Li BL, Yuen V-Y, Zhang N, et al. Intranasal dexmedetomidine with and without buccal midazolam for procedural sedation in autistic children: a double-blind randomised controlled trial. The lancet. Conference: chinese academy of medical sciences health summit, CAMS 2017. China. 2017;390:26.

270. NCT00318162, Jerusalem Institute for Child, Development, Hadassah Medical O. Trial of Low-Dose Naltrexone for Children With Pervasive Developmental Disorder (PDD). 2006.

271. NCT02007447, University of Sao Paulo General, Hospital. Oxytocin in Adolescents With Autism Spectrum Disorders. 2013.

272. Jonas C, Etienne T, Barthelemy C, Jouve J, Mariotte N. [Clinical and biochemical value of Magnesium + vitamin B6 combination in the treatment of residual autism in adults]. Therapie. 1984;39(6):661–669.

273. Stigler K, Wang Y, McDonald B, et al. Effects of aripiprazole on brain circuitry in youth with pervasive developmental disorders. Neuropsychopharmacology;1:S367.

274. ISRCTN72571312, Coventry U. A placebo controlled pilot study to explore the affects of GABA tea on children with autistic spectrum conditions. 2017.

275. Jaselskis CA, Cook EH, Fletcher KE, Leventhal BL. Clonidine treatment of hyperactive and impulsive children with autistic disorder. Journal of Clinical Psychopharmacology. 1992;12(5):322–327.

276. Woodard C, Groden J, Goodwin M, Bodfish J. A placebo double-blind pilot study of dextromethorphan for problematic behaviors in children with autism. Autism. 2007;11(1):29–41.

277. Wirojanan J, Jacquemont S, Diaz R, et al. The efficacy of melatonin for sleep problems in children with autism, fragile X syndrome, or autism and fragile X syndrome.[Erratum appears in J Clin Sleep Med. 2010 Aug 15;6(4):preceding 311]. Journal of Clinical Sleep Medicine. 2009;5(2):145–150.

278. Williams PG, Allard AM, Sears L, Dalrymple N, Bloom AS. Brief report: Case reports on naltexone use in children with autism: Controlled observations regarding benefits and practical issues of medication management. Journal of autism and developmental disorders. 2001;31(1):103–108.

279. Sanchez LE, Adams PB, Uysal S, Hallin A, Campbell M, Small AM. A comparison of live and videotape ratings: clomipramine and haloperidol in autism. Psychopharmacology Bulletin. 1995;31(2):371–378.

280. S, man CA. Beta-endorphin disregulation in autistic and self-injurious behavior: a neurodevelopmental hypothesis. Synapse (new york, N.Y.). 1988;2(3):193–199.

281. Sandman CA. B-endorphin disregulation in autistic and self-injurious behavior: A neurodevelopmental hypothesis. Synapse. 1988;2(3):193–199.

282. Ross DL, Klykylo WM, Hitzemann R. Reduction of elevated CSF beta-endorphin by fenfluramine in infantile autism. Pediatric Neurology. 1987;3(2):83–86.

283. Reiss AL, Egel AL, Feinstein C, Goldsmith B, Borengasser-Caruso MA. Effects of fenfluramine on social behavior in autistic children. Journal of autism and developmental disorders. 1988;18(4):617–625.

284. Pritchard WS, Raz N, August GJ. No effect of chronic fenfluramine on the P300 component of the event-related potential. International journal of neuroscience. 1987;35(1):105–110.

285. Piggott LR, Gdowski CL, Villanueva D, Fischhoff J, Frohman CF. Side effects of fenfluramine in autistic children. Journal of the American Academy of Child Psychiatry. 1986;25(2):287–289.

286. Ney P, Neal T, Manku MS. Double blind cross-over trial with fenfluramine. Canadian Journal of Psychiatry - Revue Canadienne de Psychiatrie. 1988;33(6):574.

287. NCT02812368, Autism Treatment N, Autism Intervention Research Network on Physical, Health, Ohio State U. Clonidine for Sleep Disturbances in Children With Autism Spectrum Disorder. 2016.

288. NCT02552147, Autism S, Yale U. Nicotinic Cholinergic Modulation as a Novel Treatment Strategy for Aggression Associated With Autism. 2015.

289. NCT02414451, University of M-C. Trial of Propranolol in Adults and Adolescents With ASD and Predictors of Response. 2015.

290. NCT02111551, University of Colorado D. Phase I Nicotinic Agonist Treatment Trial for Autism. 2014.

291. NCT02094651, University of L, Boston Children’s H. Treatment of Children With Autism Spectrum Disorders and Epileptiform EEG With Divalproex Sodium. 2014.

292. NCT01887132, National Institute of Mental, Health. A Trial of the Drug Donepezil for Sleep Enhancement and Behavioral Change in Children With Autism. 2013.

293. NCT01734941, Hadassah Medical O. TSO in Pediatric Autistic Spectrum Disorders. 2012.

294. NCT01337687, National Alliance for Research on, Schizophrenia, Depression, Montefiore Medical C. Intranasal Oxytocin for the Treatment of Autism Spectrum Disorders. 2010.

295. NCT01248130, Massachusetts General H. Omega-3 Fatty Acids Monotherapy in Children and Adolescents With Autism Spectrum Disorders. 2010.

296. NCT01078844, Forest L, Johns Hopkins U. Memantine in Adult Autism Spectrum Disorder. 2010.

297. NCT00467753, Dentistry of New J, National Alliance for Research on, Schizophrenia, Depression, University of M. Oxcarbazepine Versus Placebo in Childhood Autism. 2007.

298. NCT02081027, Children’s Hospital Medical Center, Cincinnati. Pilot Study of Riluzole for Drug-Refractory Irritability in Autism Spectrum Disorders. 2013.

299. Wink LK, Adams R, Horn PS, et al. A randomized placebo-controlled cross-over pilot study of riluzole for drug-refractory irritability in autism spectrum disorder. Journal of autism and developmental disorders. 2018.

300. Wink LK, Adams R, Horn PS, et al. A Randomized Placebo-Controlled Cross-Over Pilot Study of Riluzole for Drug-Refractory Irritability in Autism Spectrum Disorder. Journal of Autism & Developmental Disorders. 2018;48(9):3051–3060.

301. Linday L, Tsiouris JA, Cohen IL, DeCresce R. Famotidine treatment of young children with autistic spectrum disorders. 152nd annual meeting of the american psychiatric association; 1999 may 15-20; washington, DC. 1999.

302. Linday LA, Tsiouris JA, Cohen IL, Shindledecker R, DeCresce R. Famotidine treatment of children with autistic spectrum disorders: pilot research using single subject research design. Journal of neural transmission (vienna, austria : 1996). 2001;108(5):593–611.

303. Stephenson MB. Famotidine (Pepcid) and Autistic Spectrum Disorders: A Reason for Optimism, or for Heartburn? The Scientific Review of Mental Health Practice: Objective Investigations of Controversial and Unorthodox Claims in Clinical Psychology, Psychiatry, and Social Work. 2002;1(2):184–188.

304. Kamiyama M, Kuriyama S, Watanabe M. A clinical study of pyridoxine treatment for pervasive developmental disorders with hypersensitivity to sound. [Japanese]. No to Hattatsu [Brain & Development]. 2006;38(4):277–282.

305. Kuriyama, Kamiyama, Watanabe, et al. Pyridoxine treatment in a subgroup of children with pervasive developmental disorders. Developmental Medicine & Child Neurology. 2002;44(4):284–286.

306. Lewis AS, van Schalkwyk GI, Lopez MO, Volkmar FR, Picciotto MR, Sukhodolsky DG. An Exploratory Trial of Transdermal Nicotine for Aggression and Irritability in Adults with Autism Spectrum Disorder. Journal of autism and developmental disorders. 2018;48:1–10.

307. Beeghly JH, Kuperman S, Perry PJ, Wright GJ, Tsai LY. Fenfluramine treatment of autism: relationship of treatment response to blood levels of fenfluramine and norfenfluramine. Journal of autism and developmental disorders. 1987;17(4):541–548.

308. Black SL. Naltrexone in infantile autism. Journal of Autism & Developmental Disorders. 1994;24(2):236–239.

309. Leboyer M, Bouvard MP, Lensing P, et al. Opioid excess hypothesis of autism: A double-blind study of naltrexone. Brain Dysfunction. 1990;3(5):285–298.

310. Leboyer M, Bouvard MP, Launay J-M, et al. A double-blind study of naltrexone in infantile autism. Journal of Autism & Developmental Disorders. 1992;22(2):309–319.

311. Leboyer M, Bouvard MP, Launay JM, et al. Brief report: a double-blind study of naltrexone in infantile autism. Journal of autism and developmental disorders. 1992;22(2):309–319.

312. Leboyer M, Bouvard MP, Launay JM, et al. Opiate hypothesis in infantile autism? Therapeutic trials with naltrexone. Encephale. 1993;19(2):95–102.

313. Buchsbaum MS, Holl, er E, et al. Effect of fluoxetine on regional cerebral metabolism in autistic spectrum disorders: a pilot study. The international journal of neuropsychopharmacology. 2001;4(2):119–125.

314. Coggins TE, Morisset C, Krasney L, Frederickson R, Holm VA, Raisys VA. Brief Report: Does fenfluramine treatment enhance the cognitive and communicative functioning of autistic children. Journal of Autism & Developmental Disorders. 1988;18(3):425–434.

315. Dollfus S, Petit M, Menard JF, Lesieur P. Amisulpride versus bromocriptine in infantile autism: a controlled crossover comparative study of two drugs with opposite effects on dopaminergic function. Journal of autism and developmental disorders. 1992;22(1):47–60.

316. Dollfus S, Petit P, Menard JF. Pharmacoclinical study of an agonist and an antagonist of dopamine in early infantile autism. Neuropsychiatrie de l’Enfance et de l’Adolescence. 1992;40(5):300–309.

317. Dollfus S, Petit M, Launay JM, et al. Platelet serotonin in infantile autism. Cross-over effects of a dopamine agonist and an antagonist. Encephale. 1992;18(6):605–610.

318. Dollfus S, Petit M, Garnier JP, et al. Catecholamines in autistic disorder: Effects of amisulpride and bromocriptine in a controlled crossover study. Journal of Child and Adolescent Psychopharmacology. 1993;3(3):145–156.

319. Ho HH, Lockitch G, Eaves L, Jacobson B. Blood serotonin concentrations and fenfluramine therapy in autistic children. Journal of pediatrics. 1986;108(3):465–469.

320. Zingarelli G, Ellman G, Hom A, Wymore M, Heidorn S, Chicz-Demet A. Clinical effects of naltrexone on autistic behavior. American Journal on Mental Retardation. 1992;97(1):57–63.

321. NCT01170325, National Institute of Mental, Health. A Study of Divalproex Sodium in Children With ASD and Epileptiform EEG. 2010.

322. Fankhauser MP, Karumanchi VC, German ML, Yates A, Karumanchi SD. A double-blind, placebo-controlled study of the efficacy of transdermal clonidine in autism. Journal of clinical psychiatry. 1992;53(3):77–82.

323. NCT00936182, Related D, The International Child Development Resource, Center, Thoughtful H, The Center for A. Study of Fluconazole in Children With Autism Spectrum Disorder. 2009.

324. Cohen IL, Campbell M, Posner D. A study of haloperidol in young autistic children: a within-subjects design using objective rating scales. Psychopharmacology Bulletin. 1980;16:63–65.

325. EUCTR2008-003712-36-FR, Assistance Publique, Hopitaux De Paris. Etude de la réponse clinique et neurofonctionnelle à la fluoxétine dans l’autisme infantile - FAIR. 2008.

326. NCT00873834, Assistance Publique - Hôpitaux de, Paris. Fluoxetine Essay in Children With Autism. 2009.

327. EUCTR2016-000106-11-FR. EVALUATION OF THE EFFICIENCY OF TREATMENT BY BUMETANIDE ON AUTISTIC CHILDREN WITH A KNOWN ETIOLOGY: MULTICENTER AND DOUBLE-BLIND STUDY WITH RANDOMIZED PARALLEL GROUP, AGAINST PLACEBO. https://www.clinicaltrialsregister.eu/ctr-search/trial/2016-000106-11/FR/.

328. NCT02947880, University Hospital L. Evaluation of the Efficiency of Treatment by BUMETANIDE on Autistic Children With a Known Ethiology. 2016.

329. NCT00889538, Cumberl, Pharmaceuticals, Norton H, University of L. Study of Glutathione, Vitamin C and Cysteine in Children With Autism and Severe Behavior Problems. 2009.

330. NCT02140112, Coronado Biosciences I. Efficacy and Safety of Trichuris Suis Ova (TSO) as Compared to Placebo in Autism Spectrum Disorder. 2014.

331. NCT00376194, National Institute of Mental, Health. Mercury Chelation to Treat Autism. 2006.

332. Holl, er E, Ferretti CJ, et al. Trichuris suis ova (TSO) as an immuneinflammatory treatment for repetitive behaviors in ASD. Neuropsychopharmacology. 2013;38:S391-s392.

333. Holl, er E, Ferretti CJ, Taylor BP, Noone RH, Racine E. Trichuris Suis Ova (TSO) as an immuneinflammatory treatment for repetitive behaviors in autism spectrum disorders (ASD). European neuropsychopharmacology. 2015;2:S723.

334. Hollander E, Uzunova G, Taylor BP, et al. Randomized crossover feasibility trial of helminthic Trichuris suis ova versus placebo for repetitive behaviors in adult autism spectrum disorder. World J Biol Psychiatry. 2018:1–9.

335. NCT01040221, Simons F, Montefiore Medical C. Trichuris Suis Ova in Autism Spectrum Disorders. 2009.

336. ACTRN12609000784213, University University of New South, Wales. Oxytocin and social interactions in young people with autism spectrum disorders. 2009.

337. Dadds MR, MacDonald E, Cauchi A, Williams K, Levy F, Brennan J. Nasal oxytocin for social deficits in childhood autism: a randomized controlled trial. Journal of autism and developmental disorders. 2014;44(3):521–531.

338. Wray, Wilkins S, O’Connor, et al. Lack of communication and behavioural response of children with autism from single dose of intravenous porcine secretin. Cochrane Developmental, Psychosocial and Learning Problems Group. 2000.

339. Strathearn L, Kim S, Bastian DA, et al. Visual systemizing preference in children with autism: A randomized controlled trial of intranasal oxytocin. Dev Psychopathol;30(2):511–521.

340. Sponheim E, Oftedal G, Helverschou SB. Multiple doses of secretin in the treatment of autism: a controlled study. Acta paediatrica. 2002;91(5):540–545.

341. Sirigu A. How oxytocin affects the human brain and behavior. Hormone Research in Paediatrics;2:7.

342. Saklayen SS. Effects of propranolol on cognition and eye contact in autism spectrum disorder (ASD). Dissertation Abstracts International: Section B: The Sciences and Engineering. 2011;71(11):6601.

343. S, ler AD, Sutton KA, et al. Lack of benefit of a single dose of synthetic human secretin in the treatment of autism and pervasive developmental disorder. New England Journal of Medicine. 1999;341(24):1801–1806.

344. Roseman B, Schneider E, Crimmins D, et al. What to Measure in Autism Drug Trials. Journal of autism and developmental disorders. 2001;31(3):361–362.

345. Roberts W, Weaver L, Brian J, et al. Repeated doses of porcine secretin in the treatment of autism: a randomized, placebo-controlled trial. Pediatrics. 2001;107(5):E71.

346. Rahman A, Freedman R, Holl, er E. Alpha-7 nicotinic acetylcholine receptor positive allosteric modulator galantamine in autism spectrum disorder. Biological psychiatry. Conference: 73rd annual scientific convention and meeting of the society of biological psychiatry, SOBP 2018. United states. 2018;83(9):S369-s370.

347. Quintana DS, Westlye LT, Hope S, et al. Dose-dependent social-cognitive effects of intranasal oxytocin delivered with novel Breath Powered device in adults with autism spectrum disorder: a randomized placebo-controlled double-blind crossover trial. Translational psychiatry. 2017;7(5):e1136.

348. Quintana D, Westlye L, Hope S, et al. Dose-dependent social-cognitive effects of intrana-sal oxytocin delivered with novel breath powered device in adults with autism spectrum disorder: a randomized placebo-controlled double-blind crossover trial. Biological psychiatry. Conference: 72nd annual scientific convention and meeting of the society of biological psychiatry, SOBP 2017. United states. 2017;81(10):S167.

349. Pelphrey K. Oxytocin engages target neural systems for social motivation and social cognition. Neuropsychopharmacology. 2014;39:S63-s64.

350. Peled L, Wagner S, Perry A, Shamay-Tsoory SG. Get in touch: The role of oxytocin in social touch. Journal of Molecular Neuroscience. 2013;1:S90.

351. Owley T, Steele E, Corsello C, Risi S, McKaig K, Lord C. A double-blind, placebo-controlled trial of secretin for the treatment of autistic disorder. Medscape general medicine. 1999;1(3):e1006.

352. Owley T, McMahon W, Cook EH, et al. Multisite, double-blind, placebo-controlled trial of porcine secretin in autism. Journal of the American Academy of Child and Adolescent Psychiatry. 2001;40(11):1293–1299.

353. Novotny S, Holl, er E, et al. Increased repetitive behaviours and prolactin responsivity to oral m-chlorophenylpiperazine in adults with autism spectrum disorders. The international journal of neuropsychopharmacology. 2004;7(3):249–254.

354. Novotny S, Holl, er E, et al. Increased growth hormone response to sumatriptan challenge in adult autistic disorders. Psychiatry research. 2000;94(2):173–177.

355. Niederhofer H, Staffen W, Mair A. Immunoglobulins as an alternative strategy of psychopharmacological treatment of children with autistic disorder. Neuropsychopharmacology : official publication of the American College of Neuropsychopharmacology. 2003;28(5):1014–1015.

356. NCT03537950, King’s College L. Shifting Brain Excitation-Inhibition Balance in Autism Spectrum Disorder. 2018.

357. NCT03183674, University of Sao Paulo General, Hospital. Oxytocin in Spectrum Autism Disorders. 2017.

358. NCT03033784, National Institute of Mental, Health, Emory U. Autism Oxytocin Brain Project. 2017.

359. NCT02874690, Children’s Hospital Medical Center, Cincinnati. Stimulant Autism Test. 2016.

360. NCT02611921, Roivant Sciences I, Cures Within R, Children’s Hospital Medical Center, Cincinnati. Study of Intranasal Ketamine for Social Impairment in Autism Spectrum Disorder. 2015.

361. NCT02493426, Translational Science I, University of Minnesota C. Single Dose Intranasal Oxytocin and Cognitive Effects in Autism. 2015.

362. NCT02302209, University of California, San Francisco. Dyad Oxytocin Study (DOS). 2014.

363. NCT02278328, Simons F, Clinical Research Associates, L. L. C., Children’s Hospital of P. MEG Study of STX209. 2014.

364. NCT02090829, Translational Science I, Children’s Hospital of P, University of Minnesota C. Intranasal Oxytocin and Learning in Autism. 2014.

365. Kruppa JA, Gossen A, Oberwelland Weiss E, et al. Neural modulation of social reinforcement learning by intranasal oxytocin in male adults with high-functioning autism spectrum disorder: a randomized trial. Neuropsychopharmacology. 2019;44(4):749–756.

366. NCT01712464. Modulation of Reinforcement Learning. https://ClinicalTrials.gov/show/NCT01712464.

367. NCT01417026, Robert S. Intranasal Oxytocin and Learning in Autism. 2011.

368. NCT01183221, Bartz JPD. The Effects of Oxytocin on Complex Social Cognition in Autism Spectrum Disorders. 2010.

369. NCT01093768, National Institute of Mental, Health. Brain Imaging Study of Adults With Autism Spectrum Disorders. 2010.

370. Zamzow RM, Christ SE, Saklayen SS, et al. Effect of propranolol on facial scanning in autism spectrum disorder: a preliminary investigation. Journal of clinical and experimental neuropsychology. 2014;36(4):431–445.

371. Zamzow RM, Ferguson BJ, Ragsdale AS, Lewis ML, Beversdorf DQ. Effects of acute beta-adrenergic antagonism on verbal problem solving in autism spectrum disorder and exploration of treatment response markers. Journal of clinical and experimental neuropsychology. 2017;39(6):596–606.

372. Yamada T, Ohta H, Watanabe H, et al. Intranasal oxytocin restrictively improves emotion recognition for men with autism spectrum disorders. Neuropsychiatrie de l’Enfance et de l’Adolescence. 2012;60(5):S219.

373. NCT00263796, Anagnostou EMD. An fMRI Study of the Effect of Intravenous Oxytocin vs. Placebo on Response Inhibition and Face Processing in Autism. 2005.

374. NCT00065962, Human D, National Institute on D, Other Communication D, Eunice Kennedy Shriver National Institute of Child, Health. Secretin for the Treatment of Autism. 2003.

375. Unis AS, Munson JA, Rogers SJ, et al. A randomized, double-blind, placebo-controlled trial of porcine versus synthetic secretin for reducing symptoms of autism. Journal of the American Academy of Child and Adolescent Psychiatry. 2002;41(11):1315–1321.

376. Naviaux RK, Curtis B, Li K, et al. Low-dose suramin in autism spectrum disorder: a small, phase I/II, randomized clinical trial. Annals of Clinical and Translational Neurology. 2017;4(7):491–505.

377. NCT02508259, University of California, San Diego. University of California, San Diego (UCSD) Suramin Treatment Trial for Autism. 2015.

378. Narayanan A. Pharmacological modulation of functional connectivity in neuropsychological disorders. Dissertation Abstracts International: Section B: The Sciences and Engineering. 2013;74(5).

379. Molloy CA, Manning-Courtney P, Swayne S, et al. Lack of benefit of intravenous synthetic human secretin in the treatment of autism. Journal of autism and developmental disorders. 2002;32(6):545–551.

380. Marchezan J, Becker M, Schwartsmann G, et al. A Placebo-Controlled Crossover Trial of Gastrin-Releasing Peptide in Childhood Autism. Clinical neuropharmacology. 2017;40(3):108–112.

381. Lefevre A, Mottolese R, Redoute J, et al. Oxytocin Fails to Recruit Serotonergic Neurotransmission in the Autistic Brain. Cereb Cortex:1–10.

382. Krusch DA. Effects of repeated secretin administration on a subset of children with pervasive developmental disorder. Dissertation Abstracts International: Section B: The Sciences and Engineering. 2004;65(2).

383. Khan K, Corbett B, Czapansky-Beilman D, et al. The effect of intravenous secretin on gastrointestinal symptoms in autistic children: a double-blind placebo-controlled trial. Pediatric research. 2001;49(4):117a.

384. Kern JK, Miller S, Evans PA, Trivedi MH. Efficacy of porcine secretin in children with autism and pervasive developmental disorder. Journal of autism and developmental disorders. 2002;32(3):153–160.

385. JPRN-UMIN000016389, University of Fukui, Research Center for Child Mental Development Age Division. A research of efficacy and safety of oxytocin administration to detect others altruism in children and adolescents with reactive attachment disorder. 2015.

386. Honomichl RD, Goodlin-Jones BL, Burnham MM, Hansen RL, Anders TF. Secretin and sleep in children with autism. Child psychiatry and human development. 2002;33(2):107–123.

387. Holl, er E, Novotny S, et al. The relationship between repetitive behaviors and growth hormone response to sumatriptan challenge in adult autistic disorder. Neuropsychopharmacology. 2000;22(2):163–167.

388. Holl, er E, Novotny S, et al. Oxytocin infusion reduces repetitive behaviors in adults with autistic and Asperger’s disorders. Neuropsychopharmacology. 2003;28(1):193–198.

389. Holl, er E, Bartz J, et al. Oxytocin increases retention of social cognition in autism. Biological Psychiatry. 2007;61(4):498–503.

390. Novotny Sherie L. Decreased repetitive behaviors in response to oxytocin challenge in adult autistic disorders. 155th annual meeting of the american psychiatric association. 2002.

391. Holl, er E. V1A antagonist (RG7713) proof of mechanism study in high functioning autism spectrum disorder: clinical, biomarker and social learning effects. Neuropsychopharmacology. 2014;39:S63.

392. Holl, er E, Valle Rubido M, et al. Affective speech recognition clinical biomarker effects of a novel vasopressin 1a receptor antagonist vs placebo in adult autism. Biological Psychiatry. 2014;75(9):324s-325s.

393. Holl, er E, Valle Rubido M, et al. Clinical and biomarker effects of a novel vasopressin 1a receptor antagonist (RG7713) vs. Placebo in high functioning adult autism. Neuropsychopharmacology. 2014;39:S374-s375.

394. NCT01474278, Hoffmann-La R. A Study of RO5028442 in Adult Male High-Functioning Autistic Patients. 2011.

395. Umbricht D, Valle Rubido M, Shik F, et al. Deficient olfaction is associated with impaired ability to recognize emotions in high functioning autistic subjects and may be improved by a vasopressin 1a receptor antagonist. Biological Psychiatry. 2014;75(9):388s.

396. Umbricht D, Valle Rubido M, Shic F, et al. Olfaction is associated with ability to recognize emotions in high functioning autistic subjects. Neuropsychopharmacology. 2014;39:S584.

397. Valle Rubido M, Holl, er E, et al. A multi-center, observational study to explore the relationship between exploratory biomarkers and functional dimensions in adults with autistic spectrum disorders. European neuropsychopharmacology. Conference: 29th european college of neuropsychopharmacology congress, ECNP 2016. Austria. Conference start: 20160917. Conference end: 20160920. 2016;26:S193.

398. Valle Rubido M, Umbricht D, Shic F, et al. Results from a phase I proof-of-mechanism study with a vasopressin 1A receptor antagonist in autism spectrum disorder. European neuropsychopharmacology. 2015;25:S646-s647.

399. Hall SS, Lightbody AA, McCarthy BE, Parker KJ, Reiss AL. Effects of intranasal oxytocin on social anxiety in males with fragile X syndrome. Psychoneuroendocrinology. 2012;37(4):509–518.

400. Greene RK, Spanos M, Alderman C, et al. The effects of intranasal oxytocin on reward circuitry responses in children with autism spectrum disorder. J Neurodev Disord;10(1):12.

401. Gordon I, Jack A, Pretzsch CM, et al. Intranasal Oxytocin Enhances Connectivity in the Neural Circuitry Supporting Social Motivation and Social Perception in Children with Autism. Scientific reports. 2016;6:35054.

402. Francis SM, Kirkpatrick MG, Wit H, Jacob S. Urinary and plasma oxytocin changes in response to MDMA or intranasal oxytocin administration. Psychoneuroendocrinology. 2016;74:92–100.

403. EUCTR2012-003750-89-DE, Philipps-University M. Empathy, Autism and Oxytocin – an investigation by means of functional magnetic resonance imaging and moleculargenetic analyses. 2012.

404. EUCTR2010-022511-18-DE, Personality P, Albert-Ludwig University Freiburg, Laboratory for Biological. Behavioral effects and neural correlates of oxytocin on social attention [Verhaltenseffekte und neuronales Korrelat von Oxytocin im Kontext sozialer Aufmerksamkeit]. 2010.

405. Kanat M, Heinrichs M, Domes G. Intranasal oxytocin enhances neural correlates of face processing in autism. Journal of Intellectual Disability Research. 2015;59:117.

406. Kanat M, Spenthof I, Riedel A, Elst LT, Heinrichs M, Domes G. Restoring effects of oxytocin on the attentional preference for faces in autism. Translational psychiatry. 2017;7(4):e1097.

407. JPRN-UMIN000005809, Department of Neuropsychiatry, Showa University School of Medicine. A single-blind and crossover study examining the efficacy of intranasal oxytocin administration for social impairments in subjects with pervasive developmental disorders. 2011.

408. Lin IF, Kashino M, Ohta H, et al. The effect of intranasal oxytocin versus placebo treatment on the autonomic responses to human sounds in autism: a single-blind, randomized, placebo-controlled, crossover design study. Mol Autism;5(1):20.

409. Corbett BA, Bales KL, Swain D, et al. Comparing oxytocin and cortisol regulation in a double-blind, placebo-controlled, hydrocortisone challenge pilot study in children with autism and typical development. J Neurodev Disord. 2016;8:32.

410. Daly EM, Deeley Q, Ecker C, et al. Serotonin and the neural processing of facial emotions in adults with autism: An fMRI study using acute tryptophan depletion. Archives of General Psychiatry. 2012;69(10):1003–1013.

411. Daly E, Ecker C, Hallahan B, et al. Response inhibition and serotonin in autism: A functional MRI study using acute tryptophan depletion. Brain. 2014;137(9):2600–2610.

412. ACTRN12609000368235, University University of S. The effect of Oxytocin (OT) on social cognition and behaviour in youth with Autism Spectrum Disorders (ASD). 2009.

413. Guastella AJ, Einfeld SL, Gray KM, et al. Intranasal oxytocin improves emotion recognition for youth with autism spectrum disorders. Biological Psychiatry. 2010;67(7):692–694.

414. Ajram L. Pharmacological modulation of excitatory/ inhibitory balance in autism spectrum disorder. European neuropsychopharmacology. 2015;25:S127.

415. Ajram L, Horder J, Mendez MA, et al. Pharmacological modulation of excitatory/inhibitory balance in autism spectrum disorder. European neuropsychopharmacology. 2015;25:S61.

416. ACTRN12614000747628, University University of S. A Within-Subject Randomized Controlled Trial on the Effects of Phenytoin on Social Cognition and Behaviour in Males aged 16 Years and Older with Autism Spectrum Disorders. 2014.

417. ACTRN12615001059550, University of S. A Within-Subject Single Dose Trial on the Effects of Bremelanotide on Social Cognition and Behaviour. 2015.

418. Althaus M, Groen Y, Wijers AA, Noltes H, Tucha O, Hoekstra PJ. Oxytocin enhances orienting to social information in a selective group of high-functioning male adults with autism spectrum disorder. Neuropsychologia. 2015;79:53–69.

419. Andari E, Duhamel JR, Zalla T, Herbrecht E, Leboyer M, Sirigu A. Promoting social behavior with oxytocin in high-functioning autism spectrum disorders. Proceedings of the national academy of sciences of the united states of america. 2;107(9):4389–4394.

420. Andari E, Schneider F, Vindras P, Mottolese R, Leboyer M, Sirigu A. Oxytocin’s fingerprints in social deficits of autism spectrum disorders. [French, English] Le role de l’ocytocine dans l’autisme. Encephale;1:S18.

421. Aoki Y, Watanabe T, Abe O, et al. Oxytocin’s neurochemical effects in the medial prefrontal cortex underlie recovery of task-specific brain activity in autism: a randomized controlled trial. Molecular Psychiatry. 2015;20(4):447–453.

422. Aoki Y, Yahata N, Watanabe T, et al. Oxytocin improves behavioural and neural deficits in inferring others’ social emotions in autism. Brain. 2014;137:3073–3086.

423. JPRN-UMIN000002241, Department of Neuropsychiatry, Graduate School of Medicine University of Tokyo. Pilot study searching neural correlates of changes in social impairments induced by intranasal oxytocin administration in subjects with autism spectrum disorder. 2009.

424. JPRN-UMIN000004393, Department of Neuropsychiatry, Graduate School of Medicine University of Tokyo. Searching neural correlates of changes in social impairments induced by intranasal oxytocin administration and its association with genotypes related tooxytocin in subjects with autism spectrum disorder. 2010.

425. Watanabe T, Abe O, Kuwabara H, et al. Mitigation of sociocommunicational deficits of autism through oxytocin-induced recovery of medial prefrontal activity: a randomized trial. JAMA psychiatry. 2014;71(2):166–175.

426. Auyeung B, Lombardo MV, Heinrichs M, et al. Oxytocin increases eye contact during a real-time, naturalistic social interaction in males with and without autism. Translational psychiatry. 2015;5:e507.

427. Beversdorf D, Zamzow R, Ferguson B, Martin T, Lewis M, Stichter J. Predictors of response to propranolol for social functioning in autism spectrum disorder. Neurology. Conference: 66th American Academy of Neurology Annual Meeting, AAN. 2014;82(10).

428. Carey T, Ratliff-Schaub K, Funk J, Weinle C, Myers M, Jenks J. Double-blind placebo-controlled trial of secretin: effects on aberrant behavior in children with autism. Journal of autism and developmental disorders. 2002;32(3):161–167.

429. Chantiluke K, Barrett N, Giampietro V, Brammer M, Simmons A, Rubia K. Disorder-dissociated effects of fluoxetine on brain function of working memory in attention deficit hyperactivity disorder and autism spectrum disorder. Psychological medicine. 2015;45(6):1195–1205.

430. Chantiluke K, Barrett N, Giampietro V, et al. Inverse Effect of Fluoxetine on Medial Prefrontal Cortex Activation During Reward Reversal in ADHD and Autism. Cerebral cortex (new york, N.Y. : 1991). 2015;25(7):1757–1770.

431. Chantiluke K, Barrett N, Giampietro V, et al. Inverse fluoxetine effects on inhibitory brain activation in non-comorbid boys with ADHD and with ASD. Psychopharmacology. 2015;232(12):2071–2082.

432. Chez MG, Buchanan CP, Bagan BT, et al. Secretin and autism: a two-part clinical investigation. Journal of autism and developmental disorders. 2000;30(2):87–94.

433. Clark CE. Re: Secretin and autism: a two-part clinical investigation. Journal of autism and developmental disorders. 2001;31(2):248–249.

434. Riml, B. Comments on "Secretin and autism: a two-part clinical investigation" by M.G. Chez et al. Journal of autism and developmental disorders. 2000;30(2):95; discussion 97-8.

435. Coniglio SJ, Lewis JD, Lang C, et al. A randomized, double-blind, placebo-controlled trial of single-dose intravenous secretin as treatment for children with autism. Journal of pediatrics. 2001;138(5):649–655.

436. Coplan J, Souders MC, Mulberg AE, et al. Children with autistic spectrum disorders. II: parents are unable to distinguish secretin from placebo under double-blind conditions. Archives of disease in childhood. 2003;88(8):737–739.

437. Levy SE, Souders MC, Wray J, et al. Children with autistic spectrum disorders. I: comparison of placebo and single dose of human synthetic secretin. Archives of disease in childhood. 2003;88(8):731–736.

438. Corbett B, Khan K, Czapansky-Beilman D, et al. A double-blind, placebo-controlled crossover study investigating the effect of porcine secretin in children with autism. Clinical pediatrics. 2001;40(6):327–331.

439. DRKS00008984, Philipps-Universität Marburg vertreten durch das Koordinierungszentrum für Klinische, Studien. A placebo-controlled, double blind, randomised trial with crossover-design investigating the effect of oxytocin nasal spray on neuronal processes of empathy. 2015.

440. DRKS00010053, Max-Planck-Institut für Kognitions- und, Neurowissenschaften. Pacebo-controlled, double-blind, randomised phase II study with crossover-design investigating the modulatory effects of intranasal Oxytocin on social cognition in patients with Autism-Spectrum-Disorder. 2016.

441. Domes G, Heinrichs M, Kumbier E, Grossmann A, Hauenstein K, Herpertz SC. Effects of intranasal oxytocin on the neural basis of face processing in autism spectrum disorder. Biological Psychiatry. 2013;74(3):164–171.

442. Domes G, Kumbier E, Heinrichs M, Herpertz SC. Oxytocin promotes facial emotion recognition and amygdala reactivity in adults with asperger syndrome. Neuropsychopharmacology. 2014;39(3):698–706.

443. Dunn-Geier J, Ho HH, Auersperg E, et al. Effect of secretin on children with autism: a randomized controlled trial. Developmental medicine and child neurology. 2000;42(12):796–802.

444. Gordon I, Wyk BCV, Lucas MV, et al. The neural attunement effects of oxytocin in children with autism disorders. Biological Psychiatry. 2014;75(9):84s.

445. H, en BL, Hofkosh D. Secretin in Children with Autistic Disorder: A Double-Blind, Placebo-Controlled Trial. Journal of Developmental and Physical Disabilities. 2005;17(2):95–106.

446. Urbano MR, Okwara L, Manser P, Hartmann K, Deutsch S. A trial of d-cycloserine to treat the social deficit in older adolescents and young adults with autism spectrum disorders. Neuropsychopharmacology;2:S505-S506.

447. Urbano M, Okwara L, Manser P, Hartmann K, Herndon A, Deutsch SI. A trial of D-cycloserine to treat stereotypies in older adolescents and young adults with autism spectrum disorder. Clinical neuropharmacology. 2014;37(3):69–72.

448. Urbano M, Okwara L, Manser P, Hartmann K, Deutsch SI. A trial of d-cycloserine to treat the social deficit in older adolescents and young adults with autism spectrum disorders. Journal of neuropsychiatry and clinical neurosciences. 2015;27(2):133–138.

449. Carmen Galán de Isla, Soledad Sánchez Mateos, Lourdes Franco Hernández, Rafael Bravo Santos, Montserrat Rivero Urgell, Ana Beatriz Rodríguez Moratinos, Carmen Barriga Ibars. Tryptophan-enriched antioxidant cereals improve sleep in children with autistic spectrum and attention deficit hyperactivity disorders. J Cell Neurosci Oxid Stress. 2017;9(1):608–616.

450. Galan C, Sanchez S, Franco L, Bravo R, Rodriguez A, Barriga C. Intake of tryptophanenriched cereals and its influence on the sleep of children with neurological disorders. Acta Physiologica. 2017;698:73.

451. TCTR20180414001, No. Efficacy and adverse drug reaction betweenRisperidone solution local made and original drug in treatment of autism spectrum disorders. 2018.

452. NCT02255565, Pfizer, Seattle Children’s H. Dose Response Effects of Quillivant XR in Children With ADHD and Autism: A Pilot Study. 2014.

453. Adams JB, Baral M, Geis E, et al. Safety and efficacy of oral DMSA therapy for children with autism spectrum disorders: part A‐medical results. BMC clinical pharmacology. 2009;9:16.

454. Adams JB, Baral M, Geis E, et al. Safety and efficacy of oral DMSA therapy for children with autism spectrum disorders: part B - behavioral results. BMC clinical pharmacology. 2009;9:17.

455. NCT00811083, Southwest College of Naturopathic, Medicine. Dimercaptosuccinic Acid (DMSA) Treatment of Children With Autism and Heavy Metal Toxicity. 2008.

456. NCT02086110, University of California D. Effect of Milk Oligosaccharides and Bifidobacteria on the Intestinal Microflora of Children With Autism. 2014.

457. Li YC, Ma J, Xu HM, Yang GY, Zhang JC. Efficacy and safety of paliperidone and aripiprazole in the treatment of autism. [Chinese]. Chinese Journal of New Drugs. 30;25(16):1893–1897.

458. DeVane CL, Charles JM, Abramson RK, et al. Pharmacotherapy of Autism Spectrum Disorder: Results from the Randomized BAART Clinical Trial. Pharmacotherapy. 2019;39(6):626–635.

459. NCT01333072, Medical University of South, Carolina. Biomarkers in Autism of Aripiprazole and Risperidone Treatment (BAART). 2010.

460. NCT01395953, Massachusetts General H. Double-blind Trial of Buspirone for the Treatment of Anxiety in Youth With Autism Spectrum Disorders. 2011.

461. Miral S, Gencer O, Inal-Emiroglu FN, Baykara B, Baykara A, Dirik E. Risperidone versus haloperidol in children and adolescents with AD : a randomized, controlled, double-blind trial. European child & adolescent psychiatry. 2008;17(1):1–8.

462. Martsenkovsky I. Divalproex sodium and risperidone in the treatment of cognitive, behavioral and social dysfunction in preschool children with PDD and ADHD. European neuropsychopharmacology;2:S721-S722.

463. Malone RP, Cater J, Sheikh RM, Choudhury MS, Delaney MA. Olanzapine versus haloperidol in children with autistic disorder: an open pilot study. Journal of the American Academy of Child and Adolescent Psychiatry. 2001;40(8):887–894.

464. EUCTR2009-012102-39-IT, Azienda Ospedaliera Maggiore Della Carita‘ Di, Novara. CLINICAL STUDIES ON THE EFFECTIVENESS OF THE GLUTEN-FREE DIET AND CASEIN AND THERAPY ANTI-INFLAMMATORY BOWEL CHANGE IN PSYCHIATRIC SYMPTOMS INTESTINAL AND IN PATIENTS WITH CHILDHOOD AUTISMO. 2009.

465. Ghanizadeh A, Sahraeizadeh A, Berk M. A head-to-head comparison of aripiprazole and risperidone for safety and treating autistic disorders, a randomized double blind clinical trial. Child psychiatry and human development. 2014;45(2):185–192.

466. IRCT201110233930N15, Vice chancellor for research, Shiraz University of Medical sciences. Aripiprazole versus risperidone for treatment of autism. 2011.

467. IRCT201204037202N5, Tehran University of Medical, Sciences. Comparing efficacy and side effects of Memantine and Risperidone in treating autistic patients. 2012.

468. Nikvarz N, Alaghb, -Rad J, Tehrani-Doost M, Alimadadi A, Ghaeli P. Comparing efficacy and side effects of memantine vs. risperidone in the treatment of autistic disorder. Pharmacopsychiatry. 2017;50(1):19–25.

469. Lamberti M, Siracusano R, Italiano D, et al. Head-to-Head Comparison of Aripiprazole and Risperidone in the Treatment of ADHD Symptoms in Children with Autistic Spectrum Disorder and ADHD: a Pilot, Open-Label, Randomized Controlled Study. Paediatric drugs. 2016;18(4):319–329.

470. NCT02383758, Organization for Autism R, Emory U. An Interdisciplinary Approach to the Treatment of Encopresis in Children With Autism Spectrum Disorders. 2015.

471. NCT03408886, Arizona State U. Microbiota Transfer Therapy for Adults With Autism Spectrum Disorder (ASD) Who Have Gastrointestinal Disorders. 2018.

472. Chan AS, Sze SL, Han YMY. An intranasal herbal medicine improves executive functions and activates the underlying neural network in children with autism. Research in autism spectrum disorders. 2014;8(6):681–691.

473. ChiCTR-TRC-12001857, The Chinese University of Hong, Kong. Herbal Nose Drop for Patients with Brain Dysfunction: A Pilot Study. 2012.

474. NCT03115671, Institute of Mental Health, Singapore. Efficacy Study of Vayarin in Children With Autism and Comorbid Attention Deficit Hyperactivity Disorder (ADHD). 2017.

475. NCT02059577, Autism Research I, Arizona State U. Nutritional and Dietary Treatment Study for Children/Adults With Autism. 2014.

476. Goodarzi M, Hemayattalab R. Bone mineral density accrual in students with autism spectrum disorders: effects of calcium intake and physical training. Research in autism spectrum disorders. 2012;6(2):690–695.

477. Johnson CR, H, en BL, Zimmer M, Sacco K. Polyunsaturated fatty acid supplementation in young children with autism. Journal of Developmental and Physical Disabilities. 2010;22(1):1–10.

478. Carminati GG, Gerber F, Darbellay B, et al. Using venlafaxine to treat behavioral disorders in patients with autism spectrum disorder. Progress in neuro-psychopharmacology & biological psychiatry. 2016;65:85–95.

479. Akhondzadeh S, Asadabadi M. Risperidone plus celecoxib in children with autistic disorder: a double-blind, randomized trial. British journal of clinical pharmacology. 2012;73(6):983–984.

480. Asadabadi M, Mohammadi MR, Ghanizadeh A, et al. Celecoxib as adjunctive treatment to risperidone in children with autistic disorder: a randomized, double-blind, placebo-controlled trial. Psychopharmacology. 2013;225(1):51–59.

481. IRCT138711091556N2, Tehran University of Medical, Sciences. Celecoxib and Autism. 2009.

482. IRCT2017041333406N1, Vice Chancellor for Research of Mashhad University of Medical, Sciences. The efficacy of augmentation Donepezil to risperidon in treatment of autism spectrum disorders. 2017.

483. IRCT201702171556N96, Tehran University of Medical, Sciences. Palmitoylethanolamide as adjunctive treatment of Autism: A double blind and placebo controlled trial. 2017.

484. Khalaj M, Saghazadeh A, Shirazi E, et al. Palmitoylethanolamide as adjunctive therapy for autism: Efficacy and safety results from a randomized controlled trial. Journal of Psychiatric Research. 2018;103:104–111.

485. IRCT2017013132326N1, Vice Chancellor for Research of Mashhad University of Medical, Sciences. The efficacy ofaugmentation Flavonoid Quercetin torisperidon in treatment of autism spectrum disorders. 2017.

486. IRCT201701131556N95, Tehran University of Medical, Sciences. Baclofenin the treatment of Autism. 2017.

487. IRCT2016022826802N1, Vice Chancellor for Research of Mashhad University of Medical, Sciences. Assessment the efficacy of atomoxetin(stramox) in autism spectrum disorders. 2016.

488. IRCT201602041556N86, Tehran University of Medical, Sciences. Simvastatinin the treatment of Autism. 2016.

489. Moazen-Zadeh E, Shirzad F, Karkhaneh-Yousefi MA, Khezri R, Mohammadi MR, Akhondzadeh S. Simvastatin as an Adjunctive Therapy to Risperidone in Treatment of Autism: a Randomized, Double-Blind, Placebo-Controlled Clinical Trial. Journal of Child and Adolescent Psychopharmacology. 2018;28(1):82–89.

490. IRCT201405273930N34, Vice chancellor for research, Shiraz University of Medical sciences. Vitamin D for treating autism. 2014.

491. IRCT201402043930N33, Vice chancellor for research, Shiraz University of Medical sciences. short-term co-administration of acid folicfor treating children and adolescents with autism. 2014.

492. IRCT201110281556N29, Tehran University of Medical, Sciences. N-acetyl cysteine in the treatment of autism. 2011.

493. Nikoo M, Radnia H, Farokhnia M, Mohammadi MR, Akhondzadeh S. N-acetylcysteine as an adjunctive therapy to risperidone for treatment of irritability in autism: a randomized, double-blind, placebo-controlled clinical trial of efficacy and safety. Clinical neuropharmacology. 2015;38(1):11–17.

494. IRCT201106101556N25, Tehran University of Medical, Sciences. Amantadine in the treatment of autism. 2011.

495. Mohammadi MR, Yadegari N, Hassanzadeh E, et al. Double-blind, placebo-controlled trial of risperidone plus amantadine in children with autism: a 10-week randomized study. Clinical neuropharmacology. 2013;36(6):179–184.

496. IRCT201108155280N5, Mashhad University of Medical, Scinces. A comparative study on the effectiveness of Risperidone versus Risperidone plus naltrexone in treatment of autistic spectrum disorder in children with 6-12 years old. 2013.

497. IRCT20090117001556N107, Tehran University of Medical, Sciences. Sulforaphane as adjunctive treatment of irritability in children with Autism spectrum disorder. 2018.

498. IRCT20090117001556N104, Tehran University of Medical, Sciences. Resveratrolin treatment of autism. 2017.

499. IRCT20090117001556N102, Tehran University of Medical, Sciences. Prednisolone inautism spectrum disorders. 2017.

500. IRCT138901141556N9, Tehran University of Medical, Sciences. Tpoiramate in the treatment of autism. 2010.

501. Rezaei V, Mohammadi MR, Ghanizadeh A, et al. Double-blind, placebo-controlled trial of risperidone plus topiramate in children with autistic disorder. Progress in neuro-psychopharmacology & biological psychiatry. 2010;34(7):1269–1272.

502. Hasanzadeh E, Mohammadi MR, Ghanizadeh A, et al. A double-blind placebo controlled trial of Ginkgo biloba added to risperidone in patients with autistic disorders. Child psychiatry and human development. 2012;43(5):674–682.

503. IRCT201012031556N19, Kurdistan University of Medical, Sciences, Tehran University of Medical, Sciences. Ginkgo biloba in the treatment of autistic disorder. 2010.

504. Akhondzadeh S, Erfani S, Mohammadi MR, et al. Cyproheptadine in the treatment of autistic disorder: a double-blind placebo-controlled trial. Journal of clinical pharmacy and therapeutics. 2004;29(2):145–150.

505. Akhondzadeh S, Fallah J, Mohammadi MR, et al. Double-blind placebo-controlled trial of pentoxifylline added to risperidone: effects on aberrant behavior in children with autism. Progress in neuro-psychopharmacology & biological psychiatry. 2010;34(1):32–36.

506. IRCT138711161556N7, Tehran University of Medical, Sciences. Pentoxifylline in the treatment of autism. 2009.

507. Akhondzadeh S, Tajdar H, Mohammadi MR, et al. A double-blind placebo controlled trial of piracetam added to risperidone in patients with autistic disorder. Child psychiatry and human development. 2008;39(3):237–245.

508. Ghaleiha A, Alikhani R, Kazemi MR, et al. Minocycline as Adjunctive Treatment to Risperidone in Children with Autistic Disorder: a Randomized, Double-Blind Placebo-Controlled Trial. Journal of Child and Adolescent Psychopharmacology. 2016;26(9):784–791.

509. IRCT201302201556N50, Tehran University of Medical, Sciences. Minocycline in the treatment of autism. 2013.

510. Ghaleiha A, Asadabadi M, Mohammadi MR, et al. Memantine as adjunctive treatment to risperidone in children with autistic disorder: a randomized, double-blind, placebo-controlled trial. The international journal of neuropsychopharmacology. 2013;16(4):783–789.

511. IRCT1138901151556N10, Tehran University of Medical, Sciences. memantine in the treatment of autism. 2010.

512. Ghaleiha A, Ghyasv, M., et al. Galantamine efficacy and tolerability as an augmentative therapy in autistic children: a randomized, double-blind, placebo-controlled trial. Journal of psychopharmacology (oxford, england). 2014;28(7):677–685.

513. IRCT201204081556N40, Tehran University of Medical, Sciences. Galantamine in the treatment of autism. 2012.

514. Ghaleiha A, Mohammadi E, Mohammadi MR, et al. Riluzole as an adjunctive therapy to risperidone for the treatment of irritability in children with autistic disorder: a double-blind, placebo-controlled, randomized trial. Paediatric drugs. 2013;15(6):505–514.

515. IRCT201107281556N27, Tehran University of Medical, Sciences. Riluzole in the treatment of autism. 2011.

516. Ghaleiha A, Rasa SM, Nikoo M, Farokhnia M, Mohammadi MR, Akhondzadeh S. A pilot double-blind placebo-controlled trial of pioglitazone as adjunctive treatment to risperidone: effects on aberrant behavior in children with autism. Psychiatry research. 2015;229(1):181–187.

517. IRCT201202281556N37, Tehran University of Medical, Sciences. pioglitazone in the treatment of autism. 2012.

518. Ghanizadeh A, Ayoobzadehshirazi A. A randomized double-blind placebo-controlled clinical trial of adjuvant buspirone for irritability in autism. Pediatric Neurology. 2015;52(1):77–81.

519. IRCT201307303930N28, Shiraz University of Medical, sciences. A randomized double blind placebo controlled clinical trial of buspirone for treating autism spectrum disorders. 2014.

520. Ghanizadeh A, Moghimi-Sarani E. A randomized double blind placebo controlled clinical trial of N-Acetylcysteine added to risperidone for treating autistic disorders. BMC Psychiatry. 2013;13:196.

521. IRCT201106103930N6, Vice chancellor for research, Shiraz University of Medical sciences. N-Acetylcysteine augmentation with Rispridone in treatment of Autism in children. 2011.

522. Hajizadeh-Zaker R, Ghajar A, Mesgarpour B, Afarideh M, Mohammadi MR, Akhondzadeh S. L-Carnosine As an Adjunctive Therapy to Risperidone in Children with Autistic Disorder: a Randomized, Double-Blind, Placebo-Controlled Trial. Journal of Child and Adolescent Psychopharmacology. 2018;28(1):74–81.

523. IRCT201512081556N83, Tehran University of Medical, Sciences. L Carnosinein the treatment of Autism. 2015.

524. IRCT201101105280N3, Mashhad University of Medical, Sciences. Cyproheptadin plus Risperidon in treatment of children with Autistic Disorder: a double blind, placebo controlled study. 2011.

525. ACTRN12618001029280, None, University of S. Oxytocin in Preschoolers with Autism receiving Social Learning Therapy. 2018.

526. DRKS00008952, Zentralinstitut für seelische Gesundheit, Klinik für Psychiatrie und Psychotherapie des Kindes-und Jugendalters. Oxytocin-induced enhancement of Social Skills Training in Adolescents with ASD. 2015.

527. EUCTR2010-024202-34-DE, Klinik für Psychiatrie und Psychotherapie des Kindes- und Jugendalters am Zentralinstitut für Seelische, Gesundheit. group-therapy, autism and oxytocin - an investigation with the question "Does oxytocin (OT) enhance therapy effects in autism?“. 2013.

528. Kamp-Becker I, Poustka L, Bachmann C, et al. Study protocol of the ASD-Net, the German research consortium for the study of Autism Spectrum Disorder across the lifespan: from a better etiological understanding, through valid diagnosis, to more effective health care. BMC Psychiatry. 2017;17(1):206.

529. Preckel K, Kanske P, Singer T, Paulus FM, Krach S. Clinical trial of modulatory effects of oxytocin treatment on higher-order social cognition in autism spectrum disorder: a randomized, placebo-controlled, double-blind and crossover trial. BMC Psychiatry;16(1):329.

530. Wang M, Jiang L, Tang X. Levetiracetam is associated with decrease in subclinical epileptiform discharges and improved cognitive functions in pediatric patients with autism spectrum disorder. Neuropsychiatric disease and treatment. 2017;13:2321–2326.

531. Scahill L, McDougle CJ, Aman MG, et al. Effects of risperidone and parent training on adaptive functioning in children with pervasive developmental disorders and serious behavioral problems. Journal of the American Academy of Child and Adolescent Psychiatry. 2012;51(2):136–146.

532. Rezaei M, Moradi A, Tehrani-Doost M, Hassanabadi H, Khosroabadi R. Effects of Combining Medication and Pivotal Response Treatment on Aberrant Behavior in Children with Autism Spectrum Disorder. Children. 2018;5(2):30.

533. NCT03370510, Yale U. Translating Neuroprediction Into Precision Medicine Via Brain Priming. 2017.

534. NCT03242772, Eunice Kennedy Shriver National Institute of Child, Health, Human D, Duke U. Impact of Combined Medication and Behavioral Treatment for ASD & ADHD. 2017.

535. NCT02574741, University of California, Los Angeles. Combination Treatment for Augmenting Language in Children With ASD. 2015.

536. NCT02428205, Autism Science F, University of M-C. Combined Effects of Early Behavioral Intervention and Propranolol on ASD. 2015.

537. NCT02008396. Placebo-controlled, Randomized, Blinded, Dose Finding Phase 2 Pilot Safety Study of MDMA-assisted Therapy for Social Anxiety in Autistic Adults. Http://clinicaltrials.gov/show/nct02008396. 2014.

538. NCT02008396, Los Angeles Biomedical Research, Institute, Multidisciplinary Association for Psychedelic, Studies. MDMA-assisted Therapy for Social Anxiety in Autistic Adults. 2013.

539. NCT01914939, Massachusetts Institute of T, Massachusetts General H. A Randomized, Controlled Trial of Intranasal Oxytocin as an Adjunct to Behavioral Therapy for Autism Spectrum Disorder. 2013.

540. Minshawi NF, Wink LK, Shaffer R, et al. A randomized, placebo-controlled trial of D-cycloserine for the enhancement of social skills training in autism spectrum disorders. Molecular Autism. 2016;7:2.

541. NCT01086475, United States Department of, Defense, Indiana U. D-Cycloserine and Social Skills Training in Autism Spectrum Disorders. 2010.

542. Wink LK, Minshawi NF, Shaffer RC, et al. d-Cycloserine enhances durability of social skills training in autism spectrum disorder. Molecular Autism. 2017;8:2.

543. Du L, Shan L, Wang B, et al. A Pilot Study on the Combination of Applied Behavior Analysis and Bumetanide Treatment for Children with Autism. Journal of Child & Adolescent Psychopharmacology. 2015;25(7):585–588.

544. Zhou Y-y, Huang C-j, Liu J, Luo X-r. Efficacy of medication combined with sensory integration therapy for children with comorbid high-functioning autism spectrum disorder and attention deficit and hyperactivity disorder. [Chinese]. Chinese Journal of Clinical Psychology. 2014;22(6):1137–1140.

545. Karahmadi M, Tarrahi MJ, Vatankhah Ardestani SS, Omranifard V, Farzaneh B. Efficacy of Memantine as Adjunct Therapy for Autism Spectrum Disorder in Children Aged <14 Years. Adv Biomed Res. 2018;7:131.

546. Pedroza Garcia KA, Ronquillo D, Palacios Delgado JR, Anaya-Loyola MA, Rosado JL. Consumption of milk products with 100% b-casein a2 improves overall gastrointestinal tolerance but had no effect on behavior of Mexican children with autism spectrum disorder. Annals of nutrition and metabolism. Conference: 21st international congress of nutrition, ICN 2017. Argentina. 2017;71:389.

547. Papadaki OUR, Lykogeorgou MAR, Pap, reou THA, Lianou LOU, Chrousos GEO. Elemental formula diet in autistic children SAV Karkelis. Paediatrics and child health. 2010;15:42a.

548. NCT02911194, Research E, a2 Milk Company L, Northumbria U. a2 Milk for Autism and Attention-deficit Hyperactivity Disorder (ADHD). 2016.

549. Navarro F, Pearson DA, Fatheree N, Mansour R, Hashmi SS, Rhoads JM. Are ’leaky gut’ and behavior associated with gluten and dairy containing diet in children with autism spectrum disorders? Nutritional neuroscience. 2015;18(4):177–185.

550. Navarro FA, Pearson D, Lovel, et al. Intestinal permeability and behavior in children with autism spectrum disorder (ASD) on gluten and dairy-containing diet (GD). Journal of pediatric gastroenterology and nutrition;1:E16-E17.

551. McColl E, Adams S, Burton N, et al. Development of double blind gluten & casein free (GFCF) test foods for autism trial. Trials. 2013;14:151dummy.

552. Karkelis S, Papadaki-Pap, reou O, Lykogeorgou M, Chrousos G. Fecal calprotectin in autistic children before and after the use of elemental diet. Journal of pediatric gastroenterology and nutrition. 2010;2:E198.

553. IRCT201404212017N20, Tabriz University of Medical, Sciences. The effect of gluten free diet on gastrointestinal and behavioral indices in children with ASD. 2014.

554. ChiCTR-OON-14005638, The first Hospital of Jilin, University. The therapeutic effect and mechanism of ketogenic diet for children with autism. 2014.

555. Bashir S, Al-Ayadhi LY. Effect of camel milk on thymus and activation-regulated chemokine in autistic children: double-blind study. Pediatric research. 2014;75(4):559–563.

556. Al-Ayadhi LY, Halepoto DM, Al-Dress AM, Mitwali Y, Zainah R. Behavioral Benefits of Camel Milk in Subjects with Autism Spectrum Disorder. Journal of the college of physicians and surgeons‐pakistan : JCPSP. 2015;25(11):819–823.

557. JPRN-UMIN000015708, Shimane University School of, Medicine. Efficacy and Safety of Yokukansan in Autism Spectrum disorder: A Randomized, Multi-center, Double-Blind, Placebo-Controlled Trial. 2014.

558. Chan AS, Sze SL, Han YMY, Cheung MC. A Chan dietary intervention enhances executive functions and anterior cingulate activity in autism spectrum disorders: A randomized controlled trial. Evidence-based Complementary and Alternative Medicine. 2012;2012(262136).

559. CTRI/2015/10/006284, Ayush CCo. Study on purified and standardized UNANI Brahmi preparation to improve brain function in Autism. 2015.

560. CTRI/2016/04/006856, Central Council for Research in, Homoeopathy. Effectiveness of Homoeopathic medicines in Autism. 2016.

561. CTRI/2018/05/014017, Scsvmv U. AYURVEDA FOR AUTISM. 2018.

562. NCT03426826, Children’s Hospital Los A. The Gut-Brain Study. 2018.

563. NCT00065936. Self-Injury: diagnosis and Treatment. Https://clinicaltrials.gov/show/nct00065936. 2003.

564. ChiCTR-IIR-16008468, Chinese, P. L. A. General Hospital. Fecal Microbiota Preparation Treatment for Autism Spectrum Disorder: A Prospective, Open-label, Randomized, Controlled Trial. 2016.

565. IRCT2012111011421N1, Tehran University of Medical, Sciences. Effect of omega-3 supplementation on Autistic patients. 2013.

566. IRCT2015122625699N1, Food Technology Research I, National N. The effect of Omega-3 on Autism. 2016.

567. NTR6325, University Medical Center U. Bumetanide for the Autism Spectrum Clinical Effectiveness Trial. 2017.

568. NCT00054730, Foundation F, Cortex P. Effects of CX516 on Functioning in Fragile X Syndrome and Autism. 2003.

569. NCT00036231, Repligen C. Synthetic Human Secretin in Children With Autism and Gastrointestinal Dysfunction. 2002.

570. NCT00036244. A phase III, randomized, double-blind, placebo-controlled, multiple dose study to assess the efficacy, safety and tolerability of RG1068 (synthetic human secretin) in children with autism. Https://clinicaltrials.gov/ct2/show/nct00036244. 2002.

571. NCT00036244, Repligen C. Synthetic Human Secretin in Children With Autism. 2002.

572. EUCTR2006-006126-25-FR, Michel, Cnrs Van Der Rest. Emotional and Social deficits in Asperger syndrome - Asperger and Oxytocin. 2007.

573. EUCTR2009-009475-35-NL, jeudpsychiatrie, Karakter universitair centrum voor kinder- en. Lack of Empathy as a Symptom in various Psychiatric Disorders - Psychopathology and the Lack of Empathy. 2009.

574. Kanmani VK, Kumar S, Doshi V, Sivalingam, Nambi S. A randomised double blind placebo control study of joint attention, language, social responsiveness, behaviour and epileptic discharge following 8 weeks of levo-carnosine in children with autism spectrum disorder. Indian journal of psychiatry. Conference: 70th annual national conference of indian psychiatric society, ANCIPS 2018. India. 2018;60(5):S87.

575. Stigler KA, Hummer TA, Wang Y, McDonald BC, Saykin AJ. Social impairment is related to frontolimbic structural connectivity and functional activity in autism spectrum disorders. Neuropsychopharmacology;2:S200-S201.

576. NCT00965068, Human D, Eunice Kennedy Shriver National Institute of Child, Health. Cholesterol in ASD: Characterization and Treatment. 2009.

## 2. Eligible trials, included and ongoing

### 2.1. Included records in the systematic review

From the 183 eligible trials, 86 provided data for this analysis (underlined). Reasons for not inclusion were: retraction due to data reliability issues (k=1), not relevant scales or outcomes (k=6), crossover without relevant data before the crossover (k=40), unpublished protocols or abstracts without relevant data (k=50).

1. ACTRN12613000334707^1^ *(unpublished protocol)*

- ACTRN12613000334707, Telethon Institute for Child Health Research, University of Western Australia, Hospital Princess Margaret H. A randomized controlled trial of fish-oil supplementation for children with autism spectrum disorder. 2013.

1. ACTRN12617000441314^2^ *(unpublished protocol)*

- ACTRN12617000441314, University of Western A, University of S. A Course of Oxytocin to Improve Social Communication in Young Children with Autism. 2017.

1. Akkok 1995^3^

- Akkok F, Gokler B, Oktem F, Reid LD, Sucuoglu B. Behavioral and biochemical papameters of naltrexone in the treatment of autism. [Turkish] Otizm’de naltrekson sagaltiminin davranissal ve biyokimyasal boyutlari. Turk Psikiyatri Dergisi. 1995;6(4):251–262.

1. Aliyev_2018a^4^

- Aliyev NA. A Double-Blind Placebo-Controlled Trial of Acediprol (Valproate Sodium) For Global Severity in Child Autism Spectrum Disorders. OJNBD. 2018;2(1). doi:10.32474/OJNBD.2018.02.000127.

1. Aliyev_2018b^5^

- N. Aliyev, Z. Aliyev. A Double-Blind Placebo-Controlled Trial of levetiracetam for Global Severity in Child Autism Spectrum Disorders. ijirms. 2018;3(10). doi:10.23958/ijirms/vol03-i10/455.

1. Aman 2017^6–9^

- Aman MG, Findling RL, Hardan AY, et al. Safety and Efficacy of Memantine in Children with Autism: randomized, Placebo-Controlled Study and Open-Label Extension. Journal of Child and Adolescent Psychopharmacology. 2017;27(5):403–412.
- EUCTR2012-001630-33-GB, Forest Research Institute I. An Open-Label Extension Study of the Safety and Tolerability of Memantine in Pediatric Patients with Autism, Asperger’s Disorder or Pervasive Developmental Disorder Not Otherwise Specified (PDD-NOS). 2012.
- Katz E, Lateiner J, Spera A, Palmer R, Graham S. Memantine for the treatment of autism spectrum disorder: overview of the phase II clinical development program. Neurology. 2014;82(10).
- NCT00872898, Merz Pharmaceuticals Gmb H, Forest L. Study of Pharmacokinetics, Safety, Efficacy, and Tolerability of Memantine in Children With Autism. 2009.

1. Amminger 2008^10, 11^

- Amminger GP, Berger GE, Schäfer MR, Klier C, Friedrich MH, Feucht M. Omega-3 fatty acids supplementation in children with autism: a double-blind randomized, placebo-controlled pilot study. Biological Psychiatry. 2007;61(4):551–553.
- Gilbert DL. Regarding "Omega-3 Fatty Acids Supplementation in Children with Autism: A Double-Blind Randomized, Placebo-Controlled Pilot Study. Biological Psychiatry. 15;63(2):e13.

1. Anagnostou 2012^12, 13^

- Anagnostou E, Soorya L, Chaplin W, et al. Intranasal oxytocin versus placebo in the treatment of adults with autism spectrum disorders: a randomized controlled trial. Mol Autism. 2012;3(1):16.
- NCT00490802, Icahn School of Medicine at Mount, Sinai, Evdokia A. Intranasal Oxytocin in the Treatment of Autism. 2007.

1. Anderson 1984^14–16^ *(crossover; no relevant data before the first crossover phase)*

- Anderson LT, Campbell M, Grega DM. Haloperidol in the treatment of infantile autism: effects on learning and behavioral symptoms. American journal of psychiatry. 1984;141(10):1195–1202.
- Campbell M, Anderson LT, Small AM, Perry R, Green WH, Caplan R. The effects of haloperidol on learning and behavior in autistic children. Journal of autism and developmental disorders. 1982;12(2):167–175.
- Ornitz EM. Should autistic children be treated with haloperidol? The American Journal of Psychiatry. 1985;142(7):883–884.

1. Anderson 1989^17^

- Anderson LT, Campbell M, Adams P, Small AM, Perry R, Shell J. The effects of haloperidol on discrimination learning and behavioral symptoms in autistic children. Journal of autism and developmental disorders. 1989;19(2):227–239.

1. Arnold 2006^18^

- Arnold LE, Aman MG, Cook AM, et al. Atomoxetine for hyperactivity in autism spectrum disorders: placebo-controlled crossover pilot trial. Journal of the American Academy of Child and Adolescent Psychiatry. 2006;45(10):1196–1205.

1. Arnold 2012^19, 20^

- Arnold LE, Aman MG, Hollway J, et al. Placebo-controlled pilot trial of mecamylamine for treatment of autism spectrum disorders. Journal of Child & Adolescent Psychopharmacology. 2012;22(3):198–205.
- NCT00773812, Autism S, Ohio State U. Placebo-Controlled Pilot Trial of Mecamylamine for Treatment of Autism Spectrum Disorders. 2008.

1. Arnold_2019^21, 22^

- Arnold LE, Luna R.A, Williams K, et al. Probiotics for Gastrointestinal Symptoms and Quality of Life in Autism: a placebo-controlled pilot trial. J Child Adolesc Psychopharmacol. 2019.
- NCT02903030, Autism Treatment N, Autism S, Ohio State U. Probiotics for Quality of Life in Autism Spectrum Disorders. 2016

1. August 1987^23^ *(crossover; no relevant data before the crossover)*

- August GJ, Raz N, Baird TD. Fenfluramine response in high and low functioning autistic children. Journal of the American Academy of Child and Adolescent Psychiatry. 1987;26(3):342–346.

1. Ballester 2015^24–27^ *(crossover and sleep outcomes; no relevant data before the crossover)*

- Ballester P, Martinez MJ, Javaloyes A, Hern, ez L, Peiro AM. Agomelatine effectiveness in sleep disturbances in autism spectrum disorder. Clinical therapeutics. 2015;37(8):e132-e133.
- Ballester P, Martinez MJ, Inda M-D-M, et al. Evaluation of agomelatine for the treatment of sleep problems in adults with autism spectrum disorder and co-morbid intellectual disability. Journal of psychopharmacology (oxford, england). 2019:269881119864968. doi:10.1177/0269881119864968.
- EUCTR-2011-003313-42. Efficacy of agomelatine on sleep disturbance in Autism Spectrum Disorder (ASD). EU clinical trials register [www.clinicaltrialsregister.eu]. 2011.
- EUCTR2011-003313-42-ES, Hospital General Universitario de, Alicante. Agomelatine efficacy of the drug to improve sleep problems in autistic people. 2011.

1. Barthelemy 1989^28^ *(crossover; no relevant data before the crossover)*

- Barthelemy C, Bruneau N, Jouve J, Martineau J, Muh JP, Lelord G. Urinary dopamine metabolites as indicators of the responsiveness to fenfluramine treatment in children with autistic behavior. Journal of autism and developmental disorders. 1989;19(2):241–254.

1. Belsito 2001^29, 30^

- Belsito KM, Kirk KS, L, a RJ, Law PA, Zimmerman AW. Lamotrigine therapy for childhood autism: a randomised, double-blind, placebo-controlled trial. Neurology. 1998;50(4):A85.
- Belsito KM, Law PA, Kirk KS, L, a RJ, Zimmerman AW. Lamotrigine therapy for autistic disorder: a randomized, double-blind, placebo-controlled trial. Journal of autism and developmental disorders. 2001;31(2):175–181.

1. Bent 2011^31, 32^

- Bent S, Bertoglio K, Ashwood P, Bostrom A, Hendren RL. A pilot randomized controlled trial of omega-3 fatty acids for autism spectrum disorder. Journal of autism and developmental disorders. 2011;41(5):545–554.
- NCT00786799, Autism S, University of California, San Francisco. Omega-3 Fatty Acids for Autism Treatment. 2008.

1. Bent 2014^33, 34^

- Bent S, Hendren RL, Z, et al. Internet-based, randomized, controlled trial of omega-3 fatty acids for hyperactivity in autism. Journal of the American Academy of Child and Adolescent Psychiatry. 2014;53(6):658–666.
- NCT01694667, University of California, San Francisco, Hugo W. Moser Research Institute at Kennedy Krieger, Inc. Omega-3 Fatty Acids for Hyperactivity Treatment in Autism Spectrum Disorder. 2012.

1. Bernaets 2017^35, 36,266^ *(unpublished protocol, available abstract but not useful data)*

- Bernaerts S, Dillen C, Steyaert J, Alaerts K. The effects of four weeks of intranasal oxytocin on social responsiveness and repetitive and restricted behaviors in autism spectrum disorders: a randomized controlled trial. Biological psychiatry. Conference: 72nd annual scientific convention and meeting of the society of biological psychiatry, SOBP 2017. United states. 2017;81(10):S349-s350.
- EUCTR2014-000586-45-BE, Leuven KU. The use of Oxytocin for Autism Spectrum Disorders: Investigating the effect on behavior and at the level of the brain. 2014.
- NCT02940574. Neural and Behavioral Effects of Oxytocin in Autism Spectrum Disorders. [https://ClinicalTrials.gov/show/NCT02940574. 2016](https://ClinicalTrials.gov/show/NCT02940574.%202016).

1. Bertoglio 2010^37, 38^ *(crossover; no relevant data before the crossover)*

- Bertoglio K, Jill James S, Deprey L, Brule N, Hendren RL. Pilot study of the effect of methyl B12 treatment on behavioral and biomarker measures in children with autism. Journal of alternative and complementary medicine (new york, N.Y.). 2010;16(5):555–560.
- NCT00273650, University of California D. Efficacy Study of Subcutaneous Methyl-B12 in Children With Autism. 2006.

1. Bolman 1999^39^ *(crossover; no relevant data before the crossover)*

- Bolman WM, Richmond JA. A double-blind, placebo-controlled, crossover pilot trial of low dose dimethylglycine in patients with autistic disorder. Journal of autism and developmental disorders. 1999;29(3):191–194.

1. Bolognani_2019^40, 41^

- Bolognani F, Del Valle Rubido M, Squassante L, et al. A phase 2 clinical trial of a vasopressin V1a receptor antagonist shows improved adaptive behaviors in men with autism spectrum disorder. Sci Transl Med. 2019;11(491).
- NCT01793441, Hoffmann-La R. A Study of RG7314 to Investigate Efficacy and Safety in Individuals With Autism Spectrum Disorders (ASD). 2013.

1. Bouvard 1995^42^ *(crossover; no relevant data before the crossover)*

- Bouvard MP, Leboyer M, Launay JM, et al. Low-dose naltrexone effects on plasma chemistries and clinical symptoms in autism: a double-blind, placebo-controlled study. Psychiatry research. 1995;58(3):191–201.

1. Buitelaar 1990^43–45^ *(crossover; no relevant data before the crossover)*

- Buitelaar JK, Engel, H., Ree JM, Wied D. Behavioral effects of Org 2766, a synthetic analog of the adrenocorticotrophic hormone (4-9), in 14 outpatient autistic children. Journal of autism and developmental disorders. 1990;20(4):467–478.
- Buitelaar JK, Engel, H., et al. The use of adrenocorticotrophic hormone (4-9) analog ORG 2766 in autistic children: effects on the organization of behavior. Biological Psychiatry. 1992;31(11):1119–1129.
- Buitelaar JK, van Engel, H., et al. Deficits in social behavior in autism and their modification by a synthetic adrenocorticotrophic hormone (4-9) analog. Experientia. 1992;48(4):391–394.

1. Buitelaar 1992^46^ *(crossover; no relevant data before the crossover)*

- Buitelaar JK, Engel, H., et al. The adrenocorticotrophic hormone (4-9) analog ORG 2766 benefits autistic children: report on a second controlled clinical trial. Journal of the American Academy of Child and Adolescent Psychiatry. 1992;31(6):1149–1156.

1. Campbell 1987^47, 48^

- Campbell M, Adams P, Small AM, et al. Efficacy and safety of fenfluramine in autistic children. J. AM. ACAD. Child adolesc. PSYCHIATRY. 1988;27(4):434–439.
- Campbell M, Small AM, Palij M, et al. The efficacy and safety of fenfluramine in autistic children: preliminary analysis of a double-blind study. Psychopharmacology Bulletin. 1987;23(1):123–127.

1. Campbell 1993^49–52^

- Campbell M, Anderson LT, Small AM, Locascio JJ, Lynch NS, Choroco MC. Naltexone in autistic children: a double-blind and placebo-controlled study. Psychopharmacology Bulletin. 1990;26(1):130–135.
- Campbell M, Anderson LT, Small AM, Locascio JJ, Lynch NS, Choroco MC. Naltrexone in autistic children: a double-blind and placebo-controlled study. Psychopharmacology Bulletin. 1990;26(1):130–135.
- Campbell M, Anderson LT, Small AM, Adams P, Gonzalez NM, Ernst M. Naltrexone in autistic children: behavioral symptoms and attentional learning. Journal of the American Academy of Child and Adolescent Psychiatry. 1993;32(6):1283–1291.
- Gonzalez NM, Campbell M, Small AM, et al. Naltrexone plasma levels, clinical response and effect on weight in autistic children. Psychopharmacology Bulletin. 1994;30(2):203–208.

1. Chez 2017^53–55^ *(crossover; no relevant data before the crossover)*

- Chez M, Kile S. A Randomized, placebo-controlled, blinded, crossover, single-center study of the effects of nuedexta in the treatment of neurobehavioral symptoms of adults with Autism spectrum disorder. Neurology. Conference: 70th Annual Meeting of the American Academy of Neurology, AAN. 2017;88(16).
- Chez M, Kile S, Lepage C, Parise C, Benabides B, Hankins A. A Randomized, Placebo-Controlled, Blinded, Crossover, Pilot Study of the Effects of Dextromethorphan/Quinidine for the Treatment of Neurobehavioral Symptoms in Adults with Autism. Journal of Autism & Developmental Disorders. 2018.
- NCT01630811, Sutter H. Nuedexta for the Treatment of Adults With Autism. 2012.

1. Chugani 2016^57, 58^

- Chugani DC, Chugani HT, Wiznitzer M, et al. Efficacy of Low-Dose Buspirone for Restricted and Repetitive Behavior in Young Children with Autism Spectrum Disorder: a Randomized Trial. Journal of pediatrics. 2016;170:45-53.e1-4.
- NCT00873509, National Institute of Neurological, Disorders, Stroke, Chugani DC. Buspirone in the Treatment of 2-6 Year Old Children With Autistic Disorder. 2009.

1. Cohen 1980^59^ *(crossover; no relevant data before the crossover)*

- Cohen IL, Campbell M, Posner D. Behavioral effects of haloperidol in young autistic children. An objective analysis using a within-subjects reversal design. J am acad child psychiatr. 1980;19(4):665–677.

1. Cortesi 2012^60^ *(sleep outcmes; no relevant data)*

- Cortesi F, Giannotti F, Sebastiani T, Panunzi S, Valente D. Controlled-release melatonin, singly and combined with cognitive behavioural therapy, for persistent insomnia in children with autism spectrum disorders: a randomized placebo-controlled trial. Journal of sleep research. 2012;21(6):700–709.

1. Danfors 2005^61^ *(crossover; no relevant data before the crossover)*

- Danfors T, Knorring AL, Hartvig P, et al. Tetrahydrobiopterin in the treatment of children with autistic disorder: a double-blind placebo-controlled crossover study. Journal of Clinical Psychopharmacology. 2005;25(5):485–489.

1. Dean 2017^62–64^

- ACTRN12610000635066, University University of M. Efficacy Of N-Acetyl Cysteine In Autism: A Double-Blind, Placebo-Controlled Randomised Trial. 2010.
- Dean OM, Gray KM, Villagonzalo KA, et al. A randomised, double blind, placebo-controlled trial of a fixed dose of N-acetyl cysteine in children with autistic disorder. Australian and New Zealand Journal of Psychiatry. 2017;51(3):241–249.
- Dean OM, Gray K, Dodd S, et al. Does n-acetylcysteine improve behaviour in children with autism?: A mixed-methods analysis of the effects of n-acetylcysteine. Journal of Intellectual and Developmental Disability. 2018.

1. Duker 1991^65^ *(crossover; no relevant data before the crossover)*

- Duker PC, Welles K, Seys D, Rensen H. Brief report: effects of fenfluramine on communicative, stereotypic, and inappropriate behaviors of autistic-type mentally handicapped individuals. Journal of autism and developmental disorders. 1991;21(3):355–363.

1. EFFET 2015^66, 67^ *(unpublished protocol)*

- EUCTR2015-000955-25-FR, Centre Hospitalier Régional Universitaire de, Nancy. Evaluation of the efficiency of B9 vitaminon the reduction of autistic spectrum symptoms:a pilot study "EFFET. 2015.
- NCT02551380. Folinic Acid in Children With Autism Spectrum Disorders. https://ClinicalTrials.gov/show/NCT02551380. 2015.

1. Ekman 1989^68^

- Ekman G, Mir, a-Linné F, Gillberg C, Garle M, Wetterberg L. Fenfluramine treatment of twenty children with autism. Journal of autism and developmental disorders. 1989;19(4):511–532.

1. Fahmy 2013^73, 74^

- Fahmy SF, El-Hamamsy M, Zaki O, Badary OA. Effect of l-carnitine on behavioral disorder in autistic children. Value in health. 2013;16(3):A15.
- Fahmy SF, El-Hamamsy MH, Zaki OK, Badary OA. L-Carnitine supplementation improves the behavioral symptoms in autistic children. Research in autism spectrum disorders. 2013;7(1):159–166

1. Findling 1997^75^ *(crossover; no relevant data before the crossover)*

- Findling RL, Maxwell K, Scotese-Wojtila L, Huang J, Yamashita T, Wiznitzer M. High-dose pyridoxine and magnesium administration in children with autistic disorder: an absence of salutary effects in a double-blind, placebo-controlled study. Journal of autism and developmental disorders. 1997;27(4):467–478.

1. Frye 2018^76, 77^

- Frye RE, Slattery J, Delhey L, et al. Folinic acid improves verbal communication in children with autism and language impairment: a randomized double-blind placebo-controlled trial. Molecular Psychiatry. 2018;23(2):247–256.
- NCT01602016, Arkansas Children’s Hospital Research, Institute, University of A. A Folinic Acid Intervention for Autism Spectrum Disorders. 2012.

1. Gabis 2019^78, 79^

- Gabis LV, Ben-Hur R, Shefer S, Jokel A, Shalom DB. Improvement of Language in Children with Autism with Combined Donepezil and Choline Treatment. J Mol Neurosci. 2019.
- NCT01098383, The Israeli Society of Clinical, Pediatrics, Sheba Medical C. Treatment With Acetyl-Choline Esterase Inhibitors in Children With Autism Spectrum Disorders. 2010

1. Geier 2011^80, 81^

- Geier DA, Kern JK, Davis G, et al. A prospective double-blind, randomized clinical trial of levocarnitine to treat autism spectrum disorders. Medical science monitor. 2011;17(6):Pi15-23.
- ISRCTN54273114, Autism Research I. A Clinical Trial of Levocarnitine to Treat Autism Spectrum Disorders. 2010.

1. Ghodsi 2019^82^ *(biochemical outcomes; no relevant outcome)*

- Ghodsi R, Kheirouri S, Nosrati R. Carnosine supplementation does not affect serum concentrations of advanced glycation and precursors of lipoxidation end products in autism: a randomized controlled clinical trial. Ann Clin Biochem. 2019;56(1):148–154.

1. Ghuman 2009^83^ *(crossover; no data before the crossover)*

- Ghuman JK, Aman MG, Lecavalier L, et al. Randomized, placebo-controlled, crossover study of methylphenidate for attention-deficit/hyperactivity disorder symptoms in preschoolers with developmental disorders. Journal of Child & Adolescent Psychopharmacology. 2009;19(4):329–339.

1. Gordon 1993^84, 85^ *(crossover; no data before the crossover)*

- Gordon CT, Rapoport JL, Hamburger SD, State RC, Mannheim GB. Differential response of seven subjects with autistic disorder to clomipramine and desipramine. American journal of psychiatry. 1992;149(3):363–366.
- Gordon CT, State RC, Nelson JE, Hamburger SD, Rapoport JL. A double-blind comparison of clomipramine, desipramine, and placebo in the treatment of autistic disorder. Archives of General Psychiatry. 1993;50(6):441–447

1. Gringras 2017^86–91^ *(sleep outcome, associated symptoms, not eligible scales for this review; no relevant data)*

- Findling RL, Gringras P, Nir T, Zisapel N. Short- and long-term prolonged release melatonin treatment for sleep disorders in children with autism spectrum disorders - Results of a phase III randomized clinical trial. Journal of the american academy of child and adolescent psychiatry. Conference: 64th annual meeting american academy of child and adolescent psychiatry, AACAP 2017. United states. 2017;56(10):S167.
- Gringras P, Findling RL, Nir T, Zisapel N. Short and long term prolonged release melatonin treatment for sleep disorders in children with autism spectrum disorders: results of a phase III randomized clinical trial. Sleep medicine. Conference: 14th world sleep congress. Czech republic. 2017;40:e119.
- Gringras P, Findling R, Nir T, Zisapel N. Short and long term prolonged release melatonin treatment for sleep disorders in children with autism spectrum disorders: results of a phase iii randomized clinical trial. Developmental medicine and child neurology. Conference: 44th annual conference of the british paediatric neurology association, BPNA 2018. United kingdom. 2017;59:27.
- Gringras P, Nir T, Breddy J, Frydman-Marom A, Findling RL. Efficacy and Safety of Pediatric Prolonged-Release Melatonin for Insomnia in Children With Autism Spectrum Disorder. Journal of the American Academy of Child and Adolescent Psychiatry. 2017;56(11):948-957.e4.
- NCT01906866. Efficacy and Safety of Circadin® in the Treatment of Sleep Disturbances in Children With Neurodevelopment Disabilities. https://ClinicalTrials.gov/show/NCT01906866.
- Schroder CM, Malow BA, Maras A, et al. Pediatric Prolonged-Release Melatonin for Sleep in Children with Autism Spectrum Disorder: Impact on Child Behavior and Caregiver's Quality of Life. Journal of Autism & Developmental Disorders. 2019

1. Guastella 2015^92–94^

- ACTRN12609000513213, University University of S. A course of oxytocin nasal spray (OT) to treat social problems in youth with autism spectrum disorders. 2009.
- Guastella AJ. A randomized controlled trial of oxytocin nasal spray to treat youth diagnosed with autism spectrum disorders. Biological Psychiatry. 2012;71(8):234s.
- Guastella AJ, Gray KM, Rinehart NJ, et al. The effects of a course of intranasal oxytocin on social behaviors in youth diagnosed with autism spectrum disorders: a randomized controlled trial. Journal of child psychology and psychiatry, and allied disciplines. 2015;56(4):444–452

1. Handen 2000^97^ *(crossover; no relevata data before the crossover)*

- Handen BL, Johnson CR, Lubetsky M. Efficacy of methylphenidate among children with autism and symptoms of attention-deficit hyperactivity disorder. Journal of autism and developmental disorders. 2000;30(3):245–255.

1. Handen 2009^98, 99^

- Handen BL, Melmed RD, Hansen RL, et al. A double-blind, placebo-controlled trial of oral human immunoglobulin for gastrointestinal dysfunction in children with autistic disorder. Journal of autism and developmental disorders. 2009;39(5):796–805.
- NCT00110708, PediaMed P. Safety and Efficacy Study in the Treatment of Intestinal Problems Associated With Autism. 2005

1. Handen 2012^100–103^

- Handen, Benjamin L., Department of Psychiatry, Merck Program, Western Psychiatric Institute and Clinic, 3811 O’Hara St., Pittsburgh, PA, US, 15213, Johnson CR, McAuliffe-Bellin S, Hardan A. Safety and efficacy of Donepezil in children and adolescents with autism: Behavioral measures. Merrick, Joav [Ed]. 2012.
- Handen, Benjamin L., Department of Psychiatry, Merck Program, Western Psychiatric Institute and Clinic, 3811 O’Hara St., Pittsburgh, PA, US, 15213, Johnson CR, McAuliffe-Bellin S, Hardan A. Safety and efficacy of donepezil in children and adolescents with autism: Behavioral measures. Zachor, Ditza A [Ed]. 2013.
- Handen BL, Johnson CR, McAuliffe-Bellin S, Murray PJ, Hardan AY. Safety and efficacy of donepezil in children and adolescents with autism: neuropsychological measures. Journal of Child & Adolescent Psychopharmacology. 2011;21(1):43–50.
- NCT00047697, National Institute of Mental, Health, University of P. Donepezil HCl & Cognitive Deficits in Autism. 2002

1. Handen 2015^104–110^

- Arnold LE, Ober N, Aman MG, et al. A 1.5-Year Follow-Up of Parent Training and Atomoxetine for Attention-Deficit/Hyperactivity Disorder Symptoms and Noncompliant/Disruptive Behavior in Autism. Journal of Child and Adolescent Psychopharmacology;28(5):322–330.
- Handen BL, Aman MG, Arnold LE, et al. Atomoxetine, Parent Training, and Their Combination in Children With Autism Spectrum Disorder and Attention-Deficit/Hyperactivity Disorder. Journal of the American Academy of Child and Adolescent Psychiatry. 2015;54(11):905–915.
- Hollway JA, Aman MG, Mendoza-Burcham MI, et al. Caregiver Satisfaction with a Multisite Trial of Atomoxetine and Parent Training for Attention-Deficit/Hyperactivity Disorder and Behavioral Noncompliance in Children with Autism Spectrum Disorder. Journal of Child and Adolescent Psychopharmacology. 2016;26(9):807–814.
- Hollway JA, Mendoza-Burcham M, Andridge R, et al. Atomoxetine, Parent Training, and Their Effects on Sleep in Youth with Autism Spectrum Disorder and Attention-Deficit/Hyperactivity Disorder. Journal of Child and Adolescent Psychopharmacology. 2018;28(2):130–135.
- Lecavalier L, Pan X, Smith T, et al. Parent Stress in a Randomized Clinical Trial of Atomoxetine and Parent Training for Children with Autism Spectrum Disorder. Journal of autism and developmental disorders;48(4):980–987.
- NCT00844753, University of P, Ohio State U, University of R. Atomoxetine, Placebo and Parent Management Training in Autism. 2008.
- Tumuluru RV, Corbett-Dick P, Aman MG, et al. Adverse Events of Atomoxetine in a Double-Blind Placebo-Controlled Study in Children with Autism. Journal of Child and Adolescent Psychopharmacology. 2017;27(8):708–714.

1. Hardan 2012^111, 112^

- Hardan AY, Fung LK, Libove RA, et al. A randomized controlled pilot trial of oral N-acetylcysteine in children with autism. Biological Psychiatry. 2012;71(11):956–961.
- NCT00627705, Stanford U. A Study of N-Acetyl Cysteine in Children With Autism. 2008

1. Harfterkamp 2013^113–119^

- Harfterkamp M, Buitelaar JK, Minderaa RB, Loo-Neus G, Gaag RJ, Hoekstra PJ. Long-term treatment with atomoxetine for attention-deficit/hyperactivity disorder symptoms in children and adolescents with autism spectrum disorder: an open-label extension study. Journal of Child & Adolescent Psychopharmacology. 2013;23(3):194–199.
- Harfterkamp M, Buitelaar JK, Minderaa RB, Loo-Neus G, Gaag RJ, Hoekstra PJ. Atomoxetine in autism spectrum disorder: no effects on social functioning; some beneficial effects on stereotyped behaviors, inappropriate speech, and fear of change. Journal of Child & Adolescent Psychopharmacology. 2014;24(9):481–485.
- Harfterkamp M, Loo-Neus G, Minderaa RB, et al. A randomized double-blind study of atomoxetine versus placebo for attention-deficit/hyperactivity disorder symptoms in children with autism spectrum disorder. Journal of the American Academy of Child and Adolescent Psychiatry. 2012;51(7):733–741.
- Harfterkamp M, van der Meer J. A Randomized double-blind study of atomoxetine vs. placebo followed by an open label extension period of treatment with atomoxetine for ADHD symptoms in children with ASD. European Child and Adolescent Psychiatry;1:S216-S217.
- Harfterkamp M, van der Meer D, van der Loo-Neus G, Buitelaar JK, Minderaa RB, Hoekstra PJ. No evidence for predictors of response to atomoxetine treatment of attention-deficit/hyperactivity disorder symptoms in children and adolescents with autism spectrum disorder. Journal of Child and Adolescent Psychopharmacology. 2015;25(4):372–375.
- Meer JM, Harfterkamp M, Loo-Neus G, et al. A randomized, double-blind comparison of atomoxetine and placebo on response inhibition and interference control in children and adolescents with autism spectrum disorder and comorbid attention-deficit/hyperactivity disorder symptoms. Journal of Clinical Psychopharmacology. 2013;33(6):824–827.
- NCT00380692, Company, Eli L. Atomoxetine Versus Placebo for Symptoms of Attention-Deficit/Hyperactivity Disorder (ADHD) in Children and Adolescents With Autism Spectrum Disorder. 2006

1. Hellings 2005^120, 121^

- Hellings JA, Weckbaugh M, Nickel EJ, et al. A double-blind, placebo-controlled study of valproate for aggression in youth with pervasive developmental disorders. Journal of Child & Adolescent Psychopharmacology. 2005;15(4):682–692.
- NCT00065884, Human D, National Institute of Mental, Health, Eunice Kennedy Shriver National Institute of Child, Health. Valproate Response in Aggressive Autistic Adolescents. 2003

1. Hellings 2006^122–124^ *(crossover; no relevant data before the crossover)*

- Hellings JA, Cardona AM, Schroeder SR. Long-term safety and adverse events of risperidone in children, adolescents, and adults with pervasive developmental disorders. Journal of Mental Health Research in Intellectual Disabilities. 2010;3(3):132–144.
- Hellings JA, Zarcone JR, Cr, all K, Wallace D, Schroeder SR. Weight gain in a controlled study of risperidone in children, adolescents and adults with mental retardation and autism. Journal of Child & Adolescent Psychopharmacology. 2001;11(3):229–238.
- Hellings JA, Zarcone JR, Reese RM, et al. A crossover study of risperidone in children, adolescents and adults with mental retardation. Journal of autism and developmental disorders. 2006;36(3):401–411

1. Hendren 2016^125–127^

- Hendren RL, James SJ, Widjaja F, Lawton B, Rosenblatt A, Bent S. Randomized, Placebo-Controlled Trial of Methyl B12 for Children with Autism. Journal of Child and Adolescent Psychopharmacology. 2016;26(9):774–783.
- NCT01039792, University of California D, Arkansas Children’s Hospital Research, Institute, University of California, San Francisco. Trial of Methyl B12 on Behavioral and Metabolic Measures in Children With Autism. 2009.
- Widjaja F, James SJ, Hendren RL. Double-blind placebo controlled trial of methyl B12 on behavioral and metabolic measures in children with autism. Neuropsychiatrie de l’Enfance et de l’Adolescence. 2012;60(5):S221

1. Herscu 2019^128, 129^

- Herscu P, Handen BL, Arnold LE, et al. The SOFIA Study: Negative Multi-center Study of Low Dose Fluoxetine on Repetitive Behaviors in Children and Adolescents with Autistic Disorder. Journal of Autism & Developmental Disorders. 2019.
- NCT00515320, Autism S, Neuropharm. Study of Fluoxetine in Autism. 2007

1. Hollander 2005^128–132^

- Hollander E, Phillips A, Chaplin W, et al. A placebo controlled crossover trial of liquid fluoxetine on repetitive behaviors in childhood and adolescent autism. Neuropsychopharmacology. 2005;30(3):582–589.
- Hollander E, Swanson E, Anagnostou E, Phillips A, Chaplin W, Wasserman S. Liquid fluoxetine versus placebo for repetitive behaviors in childhood autism. Cummings, Jeffrey L [Ed]. 2006.
- NCT00004486, Mount Sinai School of, Medicine. Randomized Study of Fluoxetine in Children and Adolescents With Autism. 1999

1. Hollander 2006^133, 134^

- Anagnostou E, Esposito K, Soorya L, et al. Divalproex versus placebo for the prevention of irritability associated with fluoxetine treatment in autism spectrum disorder [11]. Journal of Clinical Psychopharmacology;26(4):444–446.
- Hollander E, Soorya L, Wasserman S, Esposito K, Chaplin W, Anagnostou E. Divalproex sodium vs. placebo in the treatment of repetitive behaviours in autism spectrum disorder. The international journal of neuropsychopharmacology. 2006;9(2):209–213

1. Hollander 2006b^135^

- Hollander E, Wasserman S, Swanson EN, et al. A double-blind placebo-controlled pilot study of olanzapine in childhood/adolescent pervasive developmental disorder. Journal of Child & Adolescent Psychopharmacology. 2006;16(5):541–548

1. Hollander 2010^136, 137^

- Hollander E, Chaplin W, Soorya L, et al. Divalproex sodium vs placebo for the treatment of irritability in children and adolescents with autism spectrum disorders. Neuropsychopharmacology. 2010;35(4):990–998.
- NCT00211757, National Institute of Neurological, Disorders, Stroke, Montefiore Medical C. Divalproex Sodium vs. Placebo in Childhood/Adolescent Autism. 2005

1. Hollander 2012^138–141^

- Hollander E. A double-blind placebo-controlled trial of fluoxetine for repetitive behaviors and global severity in adult autism spectrum disorders (American Journal of Psychiatry (2012) 169 (292-299)). American journal of psychiatry. 2012;169(5):540.
- Hollander E, Soorya L, Chaplin W, et al. A double-blind placebo-controlled trial of fluoxetine for repetitive behaviors and global severity in adult autism spectrum disorders. American journal of psychiatry. 2012;169(3):292–299.
- Hollander E, Soorya L, Chaplin W, et al. A double-blind placebo-controlled trial of fluoxetine for repetitive behaviors and global severity in adult spectrum disorders" Correction. The American Journal of Psychiatry. 2012;169(5):540.
- NCT00027404, Mount Sinai School of, Medicine. Study of Fluoxetine in Adults With Autistic Disorder. 2001

1. Ichikawa 2017^142–145^

- Ichikawa H, Mikami K, Okada T, et al. Aripiprazole in the Treatment of Irritability in Children and Adolescents with Autism Spectrum Disorder in Japan: A Randomized, Double-blind, Placebo-controlled Study. Child psychiatry and human development. 2017;48(5):796–806.
- JPRN-JapicCTI-121862, Otsuka Pharmaceutical Co L. A short treatment study of aripiprazole in pediatric patients with Autistic Disorder. 2012.
- JPRN-JapicCTI-121863, Otsuka Pharmaceutical Co L. A Long-term, Extended Treatment Study of Aripiprazole in Pediatric Patients With Autistic Disorder. 2012.
- NCT01617447, Otsuka Pharmaceutical Co L. A Short Treatment Study of Aripiprazole in Pediatric Patients With Autistic Disorder. 2012

1. IRCT20131013014994N5^146^ *(unpublished protocol)*

- IRCT20131013014994N5, Kermanshah University of Medical, Sciences. effect of vitamin D onAutism Spectrum Disorders. 2018.

1. ISRCTN04516575^147^ *(unpublished protocol)*

- ISRCTN04516575, University of R. Investigation of WCFS1 on the gut microbiota of autistic spectrum disorder (ASD) children. 2011.

1. ISRCTN20233876^148^ *(unpublished protocol)*

- ISRCTN20233876, Indywidualna Specjalistyczna Praktyka Lekarska w Miejscu, Wezwania. Benefits of polyunsaturated fatty acid (PUFA) supplementation in therapy of children and teenagers with Asperger?s Syndrome. 2012

1. Kent 2013^149–152^

- EUCTR2015-001220-31-Outside-EU/EEA, amp, Johnson Pharmaceutical R, Development LLC, Johnson. A Study of the Effectiveness and Safety of Two Doses of Risperidone in the Treatment of Children and Adolescents With Autistic Disorder. 2015.
- Kent JM, Hough D, Singh J, Karcher K, P, ina G. An open-label extension study of the safety and efficacy of risperidone in children and adolescents with autistic disorder. Journal of Child & Adolescent Psychopharmacology. 2013;23(10):676–686.
- Kent JM, Kushner S, Ning X, et al. Risperidone dosing in children and adolescents with autistic disorder: a double-blind, placebo-controlled study. Journal of autism and developmental disorders. 2013;43(8):1773–1783.
- NCT00576732, amp, Johnson Pharmaceutical R, Development LLC, Johnson. A Study of the Effectiveness and Safety of Two Doses of Risperidone in the Treatment of Children and Adolescents With Autistic Disorder. 2007

1. Kerley 2017^153, 154^

- Kerley CP, Power C, Gallagher L, Coghlan D. Lack of effect of Vitamin D 3 supplementation in autism: a 20-week, placebo-controlled RCT. Archives of disease in childhood. 2017;102(11):1030–1036.
- NCT02508922, The National Children’s Hospital, Tallaght, University of Dublin, Trinity College. Trial of Vitamin D3 Supplementation in Paediatric Autism. 2015

1. Kern 2001^155^

- Kern JK, Miller VS, Cauller PL, Kendall PR, Mehta PJ, Dodd M. Effectiveness of N,N-dimethylglycine in autism and pervasive developmental disorder. Journal of child neurology. 2001;16(3):169–173

1. King 2001^156^

- King BH, Wright DM, H, et al. Double-blind, placebo-controlled study of amantadine hydrochloride in the treatment of children with autistic disorder. Journal of the American Academy of Child and Adolescent Psychiatry. 2001;40(6):658–665

1. King 2009^157–163^

- Anonymous. Citalopram ineffective for reducing repetitive behavior in autism spectrum disorders. Journal of the National Medical Association;101(9):976.
- King BH, Dukes K, Donnelly CL, et al. Baseline factors predicting placebo response to treatment in children and adolescents with autism spectrum disorders: a multisite randomized clinical trial. JAMA pediatrics. 2013;167(11):1045–1052.
- King BH, Holl, er E, et al. Lack of efficacy of citalopram in children with autism spectrum disorders and high levels of repetitive behavior: citalopram ineffective in children with autism. Archives of General Psychiatry. 2009;66(6):583–590.
- King BH, Holl, er E, et al. Factors influencing placebo response in the STAART citalopram trial. Annals of Neurology. 2010;14:S118.
- NCT00086645, National Institute of Mental, Health, Boston U. Citalopram for Children With Autism and Repetitive Behavior (STAART Study 1). 2004.
- Scahill L, McCracken JT, Bearss K, et al. Design and subject characteristics in the federally-funded citalopram trial in children with pervasive developmental disorders. Journal of autism and developmental disorders. 2012;42(3):432–440.
- Volkmar FR. Citalopram treatment in children with autism spectrum disorders and high levels of repetitive behavior. Archives of General Psychiatry. 2009;66(6):581–582

1. Klaiman 2013^164, 165^

- Klaiman C, Huffman L, Masaki L, Elliott GR. Tetrahydrobiopterin as a treatment for autism spectrum disorders: a double-blind, placebo-controlled trial. Journal of Child & Adolescent Psychopharmacology. 2013;23(5):320–328.
- NCT00850070, BioMarin P, The Children’s Health C. Sapropterin as a Treatment for Autistic Disorder. 2009

1. Kolmen 1997^166–168^ *(crossover; no relevant data before the crossover)*

- Feldman HM, Kolmen BK, Gonzaga AM. Naltrexone and communication skills in young children with autism. Journal of the American Academy of Child and Adolescent Psychiatry. 1999;38(5):587–593.
- Kolmen BK, Feldman HM, H, en BL, Janosky JE. Naltrexone in young autistic children: a double-blind, placebo-controlled crossover study. Journal of the American Academy of Child and Adolescent Psychiatry. 1995;34(2):223–231.
- Kolmen BK, Feldman HM, H, en BL, Janosky JE. Naltrexone in young autistic children: replication study and learning measures. Journal of the American Academy of Child and Adolescent Psychiatry. 1997;36(11):1570–157

1. Kosaka 2016^169, 170^

- JPRN-UMIN000005211, Department of Neuropsychiatry, Faculty of Medical Sciences University of Fukui. A research of therapy evaluation to prosocial behavior after intranasal oxytocin administration. 2011.
- Kosaka H, Okamoto Y, Munesue T, et al. Oxytocin efficacy is modulated by dosage and oxytocin receptor genotype in young adults with high-functioning autism: a 24-week randomized clinical trial. Translational psychiatry. 2016;6(8):e872

1. Lemonnier 2012^171–175^

- EUCTR2009-010393-38-FR, BREST, C. H. U. de. ETUDE DE L’EFFICACITE D’UN TRAITEMENT PAR BUMETANIDE DANS UNE POPULATION D’ENFANTS AUTISTES. 2009.
- Hadjikhani N, Åsberg Johnels J, Lassalle A, et al. Bumetanide for autism: more eye contact, less amygdala activation. Scientific reports. 2018;8(1):3602. doi:10.1038/s41598-018-21958-x.
- Hadjikhani N, Zurcher NR, Rogier O, et al. Improving emotional face perception in autism with diuretic bumetanide: a proof-of-concept behavioral and functional brain imaging pilot study. Autism. 2015;19(2):149–157. doi:10.1177/1362361313514141.
- Lemonnier E, Degrez C, Phelep M, et al. A randomised controlled trial of bumetanide in the treatment of autism in children. Translational psychiatry. 2012;2:e202.
- NCT01078714, University Hospital B. Efficiency of Bumetanide in Autistic Children. 2010

1. Lemonnier 2017^176, 177^

- EUCTR2013-003259-39-ES, Neurochlore. Study in children and adolescents with autism. 2014.
- Lemonnier E, Villeneuve N, Sonie S, et al. Effects of bumetanide on neurobehavioral function in children and adolescents with autism spectrum disorders.[Erratum appears in Transl Psychiatry. 2017 May 9;7(5):e1124; PMID: 28485727]. Transl Psychiatry Psychiatry. 2017;7(3):e1056

1. Leventhal 1993^178^ *(crossover; no relevant data before the crossover)*

- Leventhal BL, Cook EH, Morford M, Ravitz AJ, Heller W, Freedman DX. Clinical and neurochemical effects of fenfluramine in children with autism. Journal of neuropsychiatry and clinical neurosciences. 1993;5(3):307–315.

1. Levine 1997^179^

- Levine J, Aviram A, Holan A, Ring A, Barak Y, Belmaker RH. Inositol treatment of autism. Journal of neural transmission. 1997;104(2):307–310.

1. Liu 2019^180, 181^

- Liu Y-W, Liong MT, Chung Y-CE, et al. Effects of Lactobacillus plantarum PS128 on Children with Autism Spectrum Disorder in Taiwan: A Randomized, Double-Blind, Placebo-Controlled Trial. Nutrients. 2019;11(4). doi:10.3390/nu11040820.
- ACTRN12616001002471, Yu-Yu W, Yen-Wenn L. Lactobacillus plantarum PS128 on behavior activity of children with autism. 2016

1. Loebel 2016^182, 183^

- Loebel A, Brams M, Goldman RS, et al. Lurasidone for the Treatment of Irritability Associated with Autistic Disorder. Journal of autism and developmental disorders. 2016;46(4):1153–1163.
- NCT01911442, Sunovion. Lurasidone Pediatric Autism Study. 2013.

1. Malone 2010^184, 185^ *(abstact only available, metabolic and cardiac outcomes; no relevant data)*

- Ghaffari M, West SH, Malone RP, et al. The effects of olanzapine on QTc in children with autistic disorder. Journal of Child and Adolescent Psychopharmacology;20:532.
- Malone RP, West SH, Ghaffari M, et al. Metabolic effects of olanzapine in children with autistic disorder. Journal of Child and Adolescent Psychopharmacology;20:531–532

1. Mankad 2015^186, 187^

- Mankad D, Dupuis A, Smile S, et al. A randomized, placebo controlled trial of omega-3 fatty acids in the treatment of young children with autism. Mol Autism. 2015;6:18.
- NCT01248728, Holl, Bloorview Kids Rehabilitation H, The Hospital for Sick, Children, Evdokia A. Omega-3 Fatty Acids For Treatment Of Young Children With Autism (OMG). 2010

1. Marcus 2009^188–193^

- Benton TD. Aripiprazole to treat irritability associated with autism: a placebo-controlled, fixed-dose trial. Current psychiatry reports. 2011;13(2):77–79.
- Mankoski R, Stockton G, Manos G, et al. Aripiprazole treatment of irritability associated with autistic disorder and the relationship between prior antipsychotic exposure, adverse events, and weight change. Journal of Child & Adolescent Psychopharmacology. 2013;23(8):572–576.
- Marcus RN, Owen R, Kamen L, et al. A placebo-controlled, fixed-dose study of aripiprazole in children and adolescents with irritability associated with autistic disorder. Journal of the American Academy of Child and Adolescent Psychiatry. 2009;48(11):1110–1119.
- Marcus RN, Owen R, Manos G, Mankoski R, Kamen L, McQuade RD. Aripiprazole in the treatment of irritability in pediatric patients (Aged 6-17 Years) with autistic disorder: results from a 52-week, open-label study. Journal of Child & Adolescent Psychopharmacology. 2011;21(3):229–236.
- Marcus RN, Owen R, Manos G, et al. Safety and tolerability of aripiprazole for irritability in pediatric patients with autistic disorder: a 52-week, open-label, multicenter study. Journal of clinical psychiatry. 2011;72(9):1270–1276.
- NCT00337571, amp, Commercialization I, Otsuka America P, Otsuka Pharmaceutical D. Study of Aripiprazole in the Treatment of Children and Adolescents With Autistic Disorder (AD). 2006

1. Martineuau 1985^194–196^ *(crossover; no relevant data before the crossover)*

- Barthelemy C, Garreau B, Leddet I, et al. Biological and clinical effects of oral magnesium and associated magnesium-vitamin B6 administration on certain disorders observed in infantile autism. [French] Effets Cliniques Et Biologiques De L’administration Orale Du Magnesium Seul Ou Du Magnesium Associe a La Vitamine B6 Sur Certains Troubles Observes Dans L’autisme Infantile. Therapie. 1980;35(5):627–632.
- Martineau J, Barthelemy C, Garreau B, Lelord G. Vitamin B6, magnesium, and combined B6-Mg: therapeutic effects in childhood autism. Biological Psychiatry. 1985;20(5):467–478.
- Martineau J, Barthelemy C, Roux S, Garreau B, Lelord G. Electrophysiological effects of fenfluramine or combined vitamin B6 and magnesium on children with autistic behaviour. Developmental Medicine & Child Neurology. 1989;31(6):721–727

1. Martsenkovska 2015^197, 198^ *(abstact only available; no relevant data)*

- Martsenkovska I. Risperidone and atomoxetine in the treatment of severe and challenging behaviours in children with pervasive developmental disorders. European neuropsychopharmacology. 2015;25:S649.
- Martsenkovsky I, Martsenkovska I, Martsenkovskyi D. Risperidon and atomoxetine in the treatment of several and challending behaviors in children with PDD. European Psychiatry. 2015;30:195

1. Martsenkovsky 2016^199^ *(abstract only available; no relevant data)*

- Martsenkovsky I, Martsenkovska I. The safety and efficacy of memantine hydrochloride versus placebo for children under 3 years old with autism spectrum disorders. European neuropsychopharmacology. Conference: 29th european college of neuropsychopharmacology congress, ECNP 2016. Austria. Conference start: 20160917. Conference end: 20160920. 2016;26:S729

1. Mazahery 2016^200–203^

- ACTRN12615000144516, Waitemata District Health B, Massey U. Omega-3, vitamin D and Autism in children. 2015.
- Mazahery H, Conlon C, Beck KL, et al. Vitamin D and omega-3 fatty acid supplements in children with autism spectrum disorder: a study protocol for a factorial randomised, double-blind, placebo-controlled trial. Trials. 2016;17(1):295.
- Mazahery H, Conlon CA, Beck KL, et al. A randomised controlled trial of vitamin D and omega-3 long chain polyunsaturated fatty acids in the treatment of irritability and hyperactivity among children with autism spectrum disorder. J Steroid Biochem Mol Biol. 2019;187:9–16.
- Mazahery H, Conlon CA, Beck KL, et al. A Randomised-Controlled Trial of Vitamin D and Omega-3 Long Chain Polyunsaturated Fatty Acids in the Treatment of Core Symptoms of Autism Spectrum Disorder in Children. Journal of Autism & Developmental Disorders. 2019;49(5):1778–1794

1. McDougle 1996^204–206^

- Huffman GB. Fluvoxamine for the treatment of autistic disorders in adults. American Family Physician. 1997;55(4):1375–1376.
- McDougle CJ, Naylor ST, Cohen DJ, Volkmar FR, Heninger GR, Price LH. A double-blind, placebo-controlled study of fluvoxamine in adults with autistic disorder. Archives of General Psychiatry. 1996;53(11):1001–1008.
- Vegso SJ. A double-blind placebo-controlled study of fluvoxamine in treating the symptoms of autism. 1995

1. McDougle 1998^207^

- McDougle CJ, Holmes JP, Carlson DC, Pelton GH, Cohen DJ, Price LH. A double-blind, placebo-controlled study of risperidone in adults with autistic disorder and other pervasive developmental disorders. Archives of General Psychiatry. 1998;55(7):633–641

1. McDougle 2000 *(unpublished pediatric fluvoxamine trial)*^208^

- McDougle C, Le Kresch, Posey DJ. Repetitive Thoughts and Behavior in Pervasive Developmental Disorders: Treatment with Serotonin Reuptake Inhibitors. J Autism Dev Disord. 2000;30(5):427–435 *(review that refers to this unpublished trial)*

1. Mehrazad-Saber 2018^209–211^

- IRCT2016061711689N4, Technology Of Tabriz University Of Medical Science, Nutritional Science Resea, Deputy of r. Effects Of Carnosine On Autism. 2016.
- IRCT2016061711689N5, technology of Tabriz university of medical, science, Deputy of r. Effects of carnosine supplementation on autism disorder. 2016.
- Mehrazad-Saber Z, Kheirouri S, Noorazar SG. Effects of l-Carnosine Supplementation on Sleep Disorders and Disease Severity in Autistic Children: A Randomized, Controlled Clinical Trial. Basic Clin Pharmacol Toxicol. 2018;123(1):72–77

1. Moradi 2018^212, 213^ *(biochemical outcomes; no relevant data)*

- IRCT20150519022323N2, Ferdowsi University Of Mashhad, Mashhad. The effect of Perceptual motor Exercises along with music and Vitamin D3 Supplementation in children with autism spectrum disorder. 2017.
- Moradi H, Sohrabi M, Taheri H, Khodashenas E, Movahedi A. The effects of different combinations of perceptual-motor exercises, music, and vitamin D supplementation on the nerve growth factor in children with high-functioning autism. Complementary Therapies in Clinical Practice. 2018;31:139–145

1. Munasinghe 2010^214^ *(crossover and not eligible scales; no relevant data before the crososver)*

- Munasinghe SA, Oliff C, Finn J, Wray JA. Digestive enzyme supplementation for autism spectrum disorders: a double-blind randomized controlled trial. Journal of autism and developmental disorders. 2010;40(9):1131–1138.

1. Munesue 2016^215–217^

- Higashida H, Munesue T, Kosaka H, Yamasue H, Yokoyama S, Kikuchi M. Social Interaction Improved by Oxytocin in the Subclass of Autism with Comorbid Intellectual Disabilities. Diseases. 2019;7(1).
- JPRN-UMIN000007250, Research Center for Child Mental Development, Kanazawa University. A randomized, double-blind, placebo-controlled, cross-over trial of oxytocin in patients with autism spectrum disorder. 2012.
- Munesue T, Nakamura H, Kikuchi M, et al. Oxytocin for male subjects with autism spectrum disorder and comorbid intellectual disabilities: a randomized pilot study. Frontiers in psychiatry. 2016;7.

1. Nagaraj 2006^218^

- Nagaraj R, Singhi P, Malhi P. Risperidone in children with autism: randomized, placebo-controlled, double-blind study. Journal of child neurology. 2006;21(6):450–455

1. NCT00057408^219^ *(unpublished protocol)*

- NCT00057408, National Institute of Mental, Health, Development, F. D. A. Office of Orphan Products. A Controlled Study of Olanzapine in Children With Autism. 2003

1. NCT00166621^220^ *(unpublished protocol)*

- NCT00166621, Chugani DC. Early Pharmacotherapy Aimed at Neuroplasticity in Autism : Safety and Efficacy. 2005

1. NCT00183339^221^ *(not eligible scales in clinicaltrials.gov)*

- NCT00183339, National Institute of Mental, Health, University of North Carolina, Chapel Hill. Early Intervention With Fluoxetine in Autism. 2005

1. NCT00183404^222^ *(unpublished protocol)*

- NCT00183404, National Institute of Mental, Health, Drexel U. Long-Term Olanzapine Treatment in Children With Autism. 2005.

1. NCT00198107^223^

- NCT00198107, National Institute of Mental, Health, Indiana U. Evaluating the Effectiveness of Aripiprazole and D-Cycloserine to Treat Symptoms Associated With Autism. 2005.

1. NCT00198120^224^ *(unpublished protocol)*

- NCT00198120, National Institute of Mental, Health, National Alliance for Research on, Schizophrenia, Depression, Indiana University School of, Medicine, Indiana U. Safety and Effectiveness of D-Cycloserine in Children With Autism. 2005.

1. NCT00252603^225^ *(unpublished protocol)*

- NCT00252603, Dentistry of New J, National Alliance for Autism, Research, University of M. Galantamine Versus Placebo in Childhood Autism. 2005

1. NCT00467818^226^ *(unpublished protocol)*

- NCT00467818, Dentistry of New J, National Center for C, Integrative H, University of M. Omega 3 Fatty Acids in the Treatment of Children With Autism Spectrum Disorders. 2007

1. NCT00468130^227^ *(unpublished protocol)*

- NCT00468130, Dentistry of New J, University of M. Efficacy of Aripiprazole Versus Placebo in the Reduction of Aggressive and Aberrant Behavior in Autistic Children. 2007

1. NCT00498173^228^

- NCT00498173, National Institute of Mental, Health, Massachusetts General H. Effectiveness of Atomoxetine in Treating ADHD Symptoms in Children and Adolescents With Autism. 2007

1. NCT00572741^229^ *(unpublished protocol)*

- NCT00572741, Arkansas Children’s Hospital Research, Institute. Treating Oxidative Stress and the Metabolic Pathology of Autism. 2007

1. NCT00609531^230^

- NCT00609531, National Institute of Mental, Health, University of North Carolina, Chapel Hill. Functional MRI Evaluation of the Effect of Citalopram in Autism Spectrum Disorders. 2008

1. NCT00655174^231^ *(unpublished protocol)*

- NCT00655174, The Hospital for Sick, Children. Fluvoxamine and Sertraline in Childhood Autism - Does SSRI Therapy Improve Behaviour and/or Mood? 2008.

1. NCT00672360^232^ *(unpublished protocol)*

- NCT00672360, Baylor College of M. Folate Rechallenge. 2008.

1. NCT00870727^233^

- NCT00870727, National Institute of Mental, Health, Bristol-Myers S, Indiana U. Study of Aripiprazole in the Treatment of Pervasive Developmental Disorders. 2009.

1. NCT00881452^234^ *(unpublished protocol)*

- NCT00881452, Curemark. A Trial of CM-AT in Children With Autism. 2009

1. NCT01171937^236^ *(unpublished protocol)*

- NCT01171937, Eunice Kennedy Shriver National Institute of Child, Health, Human D, University of California, Los Angeles. Risperidone Treatment In Children With Autism Spectrum Disorder And High Levels Of Repetitive Behavior. 2010.

1. NCT01230359^237^ *(unpublished protocol)*

- NCT01230359, Qatar U, Heidelberg U, Hamad Medical C. Early Nutritional Intervention in Patients With Autism Spectrum Disorders. 2010.

1. NCT01302964^239^

- NCT01302964, Autism S, Massachusetts General H. Mirtazapine Treatment of Anxiety in Children and Adolescents With Pervasive Developmental Disorders. 2010.

1. NCT01366859^240^ *(unpublished protocol)*

- NCT01366859, Immunotec I, Nova Southeastern U. Nutritional Intervention in Children With Autism Using Whey Protein (Immunocal): Impact on Core Areas of Behavior. 2011.

1. NCT01372449^241^ *(unpublished protocol)*

- NCT01372449, Icahn School of Medicine at Mount, Sinai, Rush University Medical C, Nationwide Children’s H, Evdokia A. A Multi-site Double-blind Placebo-controlled Trial of Memantine Versus Placebo in Children With Autism (MEM). 2011

1. NCT01661855^242^

- NCT01661855, Holl, Bloorview Kids Rehabilitation H, et al. A Pilot Study of Riluzole Versus Placebo in the Treatment of Children and Adolescents With ASD. 2012.

1. NCT01745497^243^ *(unpublished protocol)*

- NCT01745497, Autism Treatment N, Massachusetts General H, et al. Iron Treatment of Sleep Disorders in Children With Autism Spectrum Disorder. 2012.

1. NCT01788072^244^ *(unpublished protocol)*

- NCT01788072, Holl, Bloorview Kids Rehabilitation H, McMaster U, St. Michael’s Hospital T, Evdokia A. INtranasal OXyTocin for the Treatment of Autism Spectrum Disorders. 2013

1. NCT01908205^246^ *(unpublished protocol)*

- NCT01908205, United States Department of, Defense, Evdokia A. Intranasal Oxytocin for the Treatment of Children and Adolescents With Autism Spectrum Disorders (ASD). 2013.

1. NCT01944046^247^ *(unpublished protocol)*

- NCT01944046, Eunice Kennedy Shriver National Institute of Child, Health, Human D, Linmarie S. Study of Oxytocin in Autism to Improve Reciprocal Social Behaviors. 2013.

1. NCT01966679^248^ *(unpublished protocol)*

- NCT01966679, University of California, Los Angeles. Targeting GABA-A for the Treatment of Social Disability in Young Adults With Autism Spectrum Disorders: A Phase II Proof of Mechanism Trial. 2013.

1. NCT01972074^250^ *(unpublished protocol)*

- NCT01972074, McLean H, Massachusetts General H. Behavioral and Neural Response to Memantine in Adolescents With Autism Spectrum Disorder. 2013.

1. NCT02222285^251^ *(unpublished protocol)*

- NCT02222285, Enzymotec. An Exploratory, Double-Blind, Placebo-Controlled Study of the Medical Food Vayarin in Children With Autism Spectrum Disorder (ASD). 2014.

1. NCT02385799^252^

- NCT02385799, Health R, Services A, R, i J. Hagerman, M. D. A Trial of Sertraline in Young Children With Autism Spectrum Disorder. 2015.

1. NCT02410902^253^ *(unpublished protocol)*

- NCT02410902, Curemark. A Trial of CM-AT in Children With Autism With All Levels of FCT (The Blum Study). 2015.

1. NCT02550912^255^ *(unpublished protocol)*

- NCT02550912, Ain Shams U. A Study Evaluating the Effect of Vitamin D on Clinical Outcome in Autistic Children. 2015.

1. NCT02586935^256^ *(unpublished protocol)*

- NCT02586935, Holl, Bloorview Kids Rehabilitation H, et al. Tideglusib vs. Placebo in the Treatment of Adolescents With Autism Spectrum Disorders. 2015.

1. NCT02757066^260^ *(unpublished protocol)*

- NCT02757066, Nobelpharma. Verification of the Efficacy of NPC-15 for Sleep Disorders of Children With Autism Spectrum Disorders. 2016.

1. NCT02909959^265^ *(unpublished protocol)*

- NCT02909959, North Carolina T, Clinical Sciences I, University of North Carolina, Chapel Hill. Sulforaphane for the Treatment of Young Men With Autism Spectrum Disorder. 2016.

1. NCT02947048^267^ *(unpublished protocol)*

- NCT02947048, Yamo Pharmaceuticals, L. L. C., Halas FP. Safety of L1-79 in Autism. 2016.

1. NCT02956226^268^ *(unpublished protocol)*

- NCT02956226, Shaare Zedek Medical C. Cannabinoids for Behavioral Problems in Children With ASD. 2016.

1. NCT03553875^281^ *(unpublished protocol)*

- NCT03553875, Massachusetts General H. Memantine for the Treatment of Social Deficits in Youth With Disorders of Impaired Social Interactions. 2018

1. Niederhofer 2002^282, 283^ *(crossover; no relevant data before the crossover)*

- Niederhofer H, Staffen W, Mair A. Galantamine may be effective in treating autistic disorder [5]. British Medical Journal. 14;325(7377):1422.
- Niederhofer H, Staffen W, Mair A. Galantamine may be effective in treating autistic disorder. BMJ (clinical research ed.). 2002;325(7377):1422.

1. Niederhofer 2002b^284, 285^ *(crossover; no relevant data before the crossover)*

- Niederhofer H, Staffen W, Mair A. Lofexidine in hyperactive and impulsive children with autistic disorder. Journal of the American Academy of Child and Adolescent Psychiatry. 2002;41(12):1396–1397.
- Niederhofer H, Staffen W, Mair A. Lofexidine in hyperactive impulsive children with autistic disorder. Journal of the American Academy of Child and Adolescent Psychiatry. 2002;41(12):1396–1397.

1. Niederhofer 2003^286^

- Niederhofer H, Staffen W, Mair A. Tianeptine: a novel strategy of psychopharmacological treatment of children with autistic disorder. Human Psychopharmacology. 2003;18(5):389–393.

1. Niederhofer 2004^287^ *(crossover; no relevant data before the crossover)*

- Niederhofer H. Venlafaxine has modest effects in autistic children. Therapy. 2004;1(1):87–90

1. Noone 2014^288–290^ *(unpublished protocol, abstract only available; no relevant data)*

- NCT01337700, Forest L, Montefiore Medical C. Milnacipran in Autism and the Functional Locus Coeruleus and Noradrenergic Model of Autism. 2010.
- Noone R, Ferretti C, Taylor B, Racine E, Holl, er E. Milnacipran vs. Placebo in adult autism spectrum disorder: impact on hyperactivity/ impulsivity domain. Neuropsychopharmacology. 2014;39:S363-s364.
- Noone RH, Ferretti CJ, Taylor BP, et al. Modulation of the locus coeruleus-noradrenergic system with milnacipran vs placebo in autism spectrum disorder. Biological Psychiatry. 2014;75(9):324s

1. Owen 2008^291–293^

- Lewis DW, Couch DM, Marcus RN, Manos G, Mankoski R, Carson WH. Efficacy and safety of flexibly-dosed aripiprazole for the treatment of irritability associated with autistic disorder in children and adolescents (6?17 years). Annals of Neurology. 2009;66:S110-111, Abstract no: 43.
- NCT00332241, amp, Commercialization I, Otsuka America P, Otsuka Pharmaceutical D. Study of Aripiprazole in the Treatment of Children and Adolescents With Autistic Disorder (AD). 2006.
- Owen R, Sikich L, Marcus RN, et al. Aripiprazole in the treatment of irritability in children and adolescents with autistic disorder. Pediatrics. 2009;124(6):1533–1540

1. Parellada 2017^294–297^

- EUCTR2007-006444-21-ES, Fundación para la Investigación Biomédica Hospital Gregorio, Marañón. EFFECT OF 8-WEEK FATTY ACIDS OMEGA-3 TREATMENT ON OXIDATIVE METABOLISM IN PATIENTS WITH AUTISM SPECTRUM DISORDER: A RANDOMISED DOUBLE-BLIND CROSSOVER PLACEBO-CONTROLLED TRIAL. - Omega-3 tr. 2010.
- Moreno C, Calvo-Escalona R, Gutierrez S, et al. Effect of omega-3 polyunsaturated fatty acids on oxidative stress in children and adolescents with autism spectrum disorders. European neuropsychopharmacology. 2014;24:S725.
- Parellada M, Llorente C, Calvo R, et al. Double-blind crossed-over randomized controlled-trial with omega-3 fatty acids for autism spectrum disorders. European neuropsychopharmacology. 2015;25:S138.
- Parellada M, Llorente C, Calvo R, et al. Randomized trial of omega-3 for autism spectrum disorders: Effect on cell membrane composition and behavior. European neuropsychopharmacology. 2017;27(12):1319–1330

1. Parker 2017^298, 299^

- NCT01624194, Stanford U. Intranasal Oxytocin Treatment for Social Deficits in Children With Autism. 2012.
- Parker KJ, Oztan O, Libove RA, et al. Intranasal oxytocin treatment for social deficits and biomarkers of response in children with autism. Proceedings of the national academy of sciences of the united states of america. 2017;114(30):8119–812

1. Pearson 2013^300, 301^ *(crossover; no relevant data before the crossover)*

- NCT00178503, National Institute of Mental, Health, The University of Texas Health Science Center, Houston. Methylphenidate for Attention Deficit Hyperactivity Disorder and Autism in Children. 2005.
- Pearson DA, Santos CW, Aman MG, et al. Effects of extended release methylphenidate treatment on ratings of attention-deficit/hyperactivity disorder (ADHD) and associated behavior in children with autism spectrum disorders and ADHD symptoms. Journal of Child & Adolescent Psychopharmacology. 2013;23(5):337–351

1. Pusponegoro 2015^302^

- Pusponegoro HD, Ismael S, Firmansyah A, Sastroasmoro S, V, enplas Y. Gluten and casein supplementation does not increase symptoms in children with autism spectrum disorder. Acta paediatrica. 2015;104(11):e500-5.

1. Quintana 1995^303, 304^ *(crossover; no relevant data before the crossover)*

- Quintana H, Birmaher B, Stedge D, et al. Use of methylphenidate in the treatment of children with autistic disorder. Journal of autism and developmental disorders. 1995;25(3):283–294.
- Quintana H, Birmaher B, Stedge D, Lennon S, al. e. Use of methylphenidate in the treatment of children with autistic disorder. Annual Progress in Child Psychiatry & Child Development. 1996:295–307.

1. Ratliff 2005^305^ *(crossover; no relevant data before the crossover)*

- Ratliff-Schaub K, Carey T, Reeves GD, Rogers MA. Randomized controlled trial of transdermal secretin on behavior of children with autism. Autism. 2005;9(3):256–265

1. Realmuto 1986^306^ *(crossover; no relevant data before the crossover)*

- Realmuto GM, Jensen J, Klykylo W, et al. Untoward effects of fenfluramine in autistic children. Journal of Clinical Psychopharmacology. 1986;6(6):350–355.

1. Reddihough 2019^307–309^

- ACTRN12608000173392, None, Victorian Medical Insurance A. Fluoxetine for the treatment of repetitive behaviours in children and adolescents with autism: A randomised double-blind placebo-controlled trial. 2008.
- Mouti A, Reddihough D, Marraffa C, et al. Fluoxetine for Autistic Behaviors (FAB trial): study protocol for a randomized controlled trial in children and adolescents with autism. Trials. 2014;15:230.
- Reddihough D, Marraffa C, Mouti A, et al. A randomised placebo-controlled trial to determine if fluoxetine is effective for improving autistic behaviours. preprint-Lancet. 2019

1. Remington 2001^310–313^ *(crossover; no relevant data before the crossover)*

- King R, Fay G, Wheildon H. Re: Clomipramine vs. haloperidol in the treatment of autistic disorder: A double-blind, placebo, crossover study. Journal of Clinical Psychopharmacology. 2002;22(5):525–526.
- Remington G, Sloman L, Konstantareas M, Parker K, Gow R. Clomipramine versus haloperidol in the treatment of autistic disorder: a double-blind, placebo-controlled, crossover study. Journal of Clinical Psychopharmacology. 2001;21(4):440–444.
- Sloman L, Konstantareas M, Remington G. Re: Clomipramine vs. haloperidol in the treatment of autistic disorder: A double-blind, placebo, crossover study." Reply to Dr. King and associates. Journal of Clinical Psychopharmacology. 2002;22(5):526.
- Sloman L, Remington G, Konstantareas M, Parker K. Haloperidol versus clomipramine in autistic disorder. 151st annual meeting of the american psychiatric association; 1998 may 30 - jun 4; toronto. 1998(17)

1. RISAUT-JPN^314, 315^ (NCT01624675)

- EUCTR2015-001320-31-Outside-EU/EEA, Janssen Pharmaceutical KK. A Study to Evaluate the Efficacy and Safety of Risperidone (R064766) in Children and Adolescents with Irritability Associated with Autistic Disorder. 2015.
- NCT01624675, Janssen Pharmaceutical KK. A Study to Evaluate the Efficacy and Safety of Risperidone (R064766) in Children and Adolescents With Irritability Associated With Autistic Disorder. 2012

1. RUPP 2002^316–335^

- Adetunji B FMathews, Maju, Mathews M FOsinowo, Thomas, Osinowo T FWilliams, Adedapo, Williams A. Risperidone for the core symptom domains of autism.
- Aman MG, Arnold LE, McDougle CJ, et al. Acute and long-term safety and tolerability of risperidone in children with autism. Journal of Child & Adolescent Psychopharmacology. 2005;15(6):869–884.
- Aman MG, Hollway JA, McDougle CJ, et al. Cognitive effects of risperidone in children with autism and irritable behavior. Journal of Child & Adolescent Psychopharmacology. 2008;18(3):227–236.
- Aman M, Rettiganti M, Nagaraja HN, et al. Tolerability, Safety, and Benefits of Risperidone in Children and Adolescents with Autism: 21-Month Follow-up After 8-Week Placebo-Controlled Trial. Journal of Child & Adolescent Psychopharmacology. 2015;25(6):482–493.
- Anderson GM, Scahill L, McCracken JT, et al. Effects of short- and long-term risperidone treatment on prolactin levels in children with autism. Biological Psychiatry. 2007;61(4):545–550.
- Arnold LE, Farmer C, Kraemer HC, et al. Moderators, mediators, and other predictors of risperidone response in children with autistic disorder and irritability. Journal of Child and Adolescent Psychopharmacology. 1;20(2):83–93.
- Arnold LE, Vitiello B, McDougle C, et al. Parent-defined target symptoms respond to risperidone in RUPP autism study: customer approach to clinical trials. Journal of the American Academy of Child and Adolescent Psychiatry. 2003;42(12):1443–1450.
- Carroll D, Hallett V, McDougle CJ, et al. Examination of aggression and self-injury in children with autism spectrum disorders and serious behavioral problems. Child and Adolescent Psychiatric Clinics of North America. 2014;23(1):57–72.
- Levine SZ, Kodesh A, Goldberg Y, et al. Initial severity and efficacy of risperidone in autism: results from the RUPP trial. European Psychiatry. 2016;32:16–20.
- Lindsay RL, Eugene Arnold L, Aman MG, et al. Dietary status and impact of risperidone on nutritional balance in children with autism: a pilot study. Journal of Intellectual and Developmental Disability. 2006;31(4):204–209.
- McDougle CJ, Scahill L, Aman MG, et al. Risperidone for the core symptom domains of autism: results from the study by the autism network of the research units on pediatric psychopharmacology. American journal of psychiatry. 2005;162(6):1142–1148.
- NCT00005014, National Institute of Mental, Health. Treatment of Autism in Children and Adolescents. 2000.
- Nurmi EL, Spilman SL, Whelan F, et al. Moderation of antipsychotic-induced weight gain by energy balance gene variants in the RUPP autism network risperidone studies. Translational psychiatry;3:e274.
- Research Units on Pediatric Psychopharmacology Autism Network. Risperidone in children with autism and serious behavioral problems. New England Journal of Medicine. 2002;347(5):314–321.
- Risperidone treatment of autistic disorder: longer-term benefits and blinded discontinuation after 6 months. American journal of psychiatry. 2005;162(7):1361–1369.
- Scahill L, Hallett V, Aman MG, et al. Brief Report: social disability in autism spectrum disorder: results from Research Units on Pediatric Psychopharmacology (RUPP) Autism Network trials. Journal of autism and developmental disorders. 2013;43(3):739–746.
- Scahill L, McCracken J, McDougle CJ, et al. Methodological issues in designing a multisite trial of risperidone in children and adolescents with autism. Journal of Child & Adolescent Psychopharmacology. 2001;11(4):377–388.
- Vitiello B, Aman MG, Scahill L, et al. Research knowledge among parents of children participating in a randomized clinical trial. Journal of the American Academy of Child and Adolescent Psychiatry;44(2):145–149.
- Vitiello B, Davies M, Arnold LE, et al. Assessment of the integrity of study blindness in a pediatric clinical trial of risperidone. Journal of Clinical Psychopharmacology. 2005;25(6):565–569.
- Vo LC, Snyder C, McCracken C, et al. No Apparent Cardiac Conduction Effects of Acute Treatment with Risperidone in Children with Autism Spectrum Disorder. Journal of Child and Adolescent Psychopharmacology. 2016;26(10):900–908

1. RUPP 2005^336–342^ *(crossover; no relevant data before the crossover)*

- Jahromi LB, Kasari CL, McCracken JT, et al. Positive effects of methylphenidate on social communication and self-regulation in children with pervasive developmental disorders and hyperactivity. Journal of autism and developmental disorders. 2009;39(3):395–404.
- McCracken JT, Badashova KK, Posey DJ, et al. Positive effects of methylphenidate on hyperactivity are moderated by monoaminergic gene variants in children with autism spectrum disorders. Pharmacogenomics journal. 2014;14(3):295–302.
- NCT00025779, National Institute of Mental, Health. Methylphenidate in Children and Adolescents With Pervasive Developmental Disorders. 2001.
- Posey DJ, Aman MG, McCracken JT, et al. Positive effects of methylphenidate on inattention and hyperactivity in pervasive developmental disorders: an analysis of secondary measures. Biological Psychiatry. 2007;61(4):538–544.
- Research Units on Pediatric Psychopharmacology (RUPP) Autism Network. Randomized, controlled, crossover trial of methylphenidate in pervasive developmental disorders with hyperactivity. Archives of General Psychiatry. 2005;62(11):1266–1274.
- Scahill L, Aman MG, McDougle CJ, et al. A prospective open trial of guanfacine in children with pervasive developmental disorders. Journal of Child and Adolescent Psychopharmacology;16(5):589–598.
- Scahill L, Bearss K, Sarhangian R, et al. Using a Patient-Centered Outcome Measure to Test Methylphenidate Versus Placebo in Children with Autism Spectrum Disorder. Journal of Child and Adolescent Psychopharmacology. 2017;27(2):125–131

1. Saad 2015^343^

- Saad K, Eltayeb AA, Mohamad IL, et al. A Randomized, Placebo-controlled Trial of Digestive Enzymes in Children with Autism Spectrum Disorders. Clinical Psychopharmacology and Neuroscience. 2015;13(2):188–193.

1. Saad 2018^344–346^ *(Retracted, due to reliability on the data)*

- Saad K. Response to letters: Randomized controlled trial of vitamin d supplementation in children with autism spectrum disorder - correction and additional information. Journal of Child Psychology and Psychiatry. 2018;59(1):e3-e5.
- Saad K, Abdel-Rahman AA, Elserogy YM, et al. Randomized controlled trial of vitamin d supplementation in children with autism spectrum disorder. Journal of Child Psychology and Psychiatry. 2018;59(1):20–29.
- Saad K, Abdel-Rahman A, Elserogy Y, et al. Retraction: Randomized controlled trial of vitamin D supplementation in children with autism spectrum disorder. Journal of Child Psychology and Psychiatry. 2019;60(6):711

1. Santocchi 2016^347, 348^ *(unpublished protocol)*

- NCT02708901, Ministry of Health I, Istituto di Fisiologia Clinica, C. N. R., Maris IFS. Gut to Brain Interaction in Autism. Role of Probiotics on Clinical, Biochemical and Neurophysiological Parameters. 2016.
- Santocchi E, Guiducci L, Fulceri F, et al. Gut to brain interaction in Autism Spectrum Disorders: a randomized controlled trial on the role of probiotics on clinical, biochemical and neurophysiological parameters. BMC Psychiatry. 2016;16:183

1. Scahill 2015^349–351^

- NCT01238575, Emory U, Massachusetts General H, et al. Guanfacine for the Treatment of Hyperactivity in Pervasive Developmental Disorder. 2010.
- Politte LC, Scahill L, Figueroa J, McCracken JT, King B, McDougle CJ. A randomized, placebo-controlled trial of extended-release guanfacine in children with autism spectrum disorder and ADHD symptoms: an analysis of secondary outcome measures. Neuropsychopharmacology;43(8):1772–1778.
- Scahill L, McCracken JT, King BH, et al. Extended-Release Guanfacine for Hyperactivity in Children With Autism Spectrum Disorder. American journal of psychiatry. 2015;172(12):1197–1206

1. Scifo 1991^352–354^ *(crossover; no relevant data before the crossosver)*

- Marchetti B, Scifo R, Batticane N, Scapagnini U. Immunological significance of opioid peptide dysfunction in infantile autism. Brain Dysfunction. 1990;3(5):346–354.
- Scifo R, Batticane N, Quattropani MC, Spoto G, Marchetti B. A double-blind trial with naltrexone in autism. Brain Dysfunction. 1991;4(6):301–307.
- Scifo R, Cioni M, Nicolosi A, et al. Opioid-immune interactions in autism: behavioural and immunological assessment during a double-blind treatment with naltrexone. Annali dell’istituto superiore di sanita. 1996;32(3):351–359

1. Shea 2004^355–359^

- Kastner TA. Use of Risperidone in Developmentally Disabled Children. Pediatrics. 2005;115(5):1447. doi:10.1542/peds.2005-0156.
- Light M, Dunbar F, Shea SE. Efficacy and safety of risperidone in the treatment of children with autistic and other pervasive developmental disorders (PDD): a randomized, double-blind, placebo controlled trial (P2.104). European neuropsychopharmacology. 2004;14:S278.
- NCT00261508, Janssen-Ortho Inc C. A Study of the Effectiveness and Safety of Risperidone Versus Placebo in the Treatment of Children With Autistic Disorder and Other Pervasive Developmental Disorders (PDD). 2005.
- Pandina GJ, Bossie CA, Youssef E, Zhu Y, Dunbar F. Risperidone improves behavioral symptoms in children with autism in a randomized, double-blind, placebo-controlled trial. Journal of autism and developmental disorders. 2007;37(2):367–373.
- Shea S, Turgay A, Carroll A, et al. Risperidone in the treatment of disruptive behavioral symptoms in children with autistic and other pervasive developmental disorders. Pediatrics. 2004;114(5):e634-41

1. Sherman 1989^360^ *(crossover; no relevant data before the crossover)*

- Sherman J, Factor DC, Swinson R, Darjes RW. The effects of fenfluramine (hydrochloride) on the behaviors of fifteen autistic children. Journal of autism and developmental disorders. 1989;19(4):533–543.

1. Sikich 2013^361, 362^ (NCT01308749)

- NCT01308749, Autism S, University of North Carolina, Chapel Hill. A Study of Oxytocin in Children and Adolescents With Autistic Disorder. 2011.
- Sikich L, Alderman C, Hazzard L, Bethea TC, Gregory S, Johnson J. Pilot study of sustained oxytocin treatment in children and adolescents with autistic disorder. Biological Psychiatry. 2013;73(9):145s

1. Singh 2014^363, 364^

- NCT01474993, Johns Hopkins U, Andrew Z. Sulforaphane-rich Broccoli Sprout Extract for Autism. 2011.
- 364. Singh K, Connors SL, Macklin EA, et al. Sulforaphane treatment of autism spectrum disorder (ASD). Proceedings of the national academy of sciences of the united states of america. 2014;111(43):15550–15555

1. SOMELIA 2015^365, 366^ *(unpublished protocol)*

- EUCTR2013-001230-17-FR, Hospices Civils de L. La mélatonine restaure-t-elle l’architecture du sommeil chez les enfants avec autisme ? Etude de phase II. 2015.
- NCT01993251. Does Melatonin Restore Sleep Architecture in Autistic Children. https://ClinicalTrials.gov/show/NCT01993251. 2013

1. Stern 1990^367, 368^ *(crossover; no relevant data before the crossover)*

- Oades RD, Stern LM, Walker MK, Clark CR, Kapoor V. Event-related potentials and monoamines in autistic children on a clinical trial of fenfluramine. International journal of psychophysiology. 1990;8(3):197–212.
- Stern LM, Walker MK, Sawyer MG, Oades RD, Badcock NR, Spence JG. A controlled crossover trial of fenfluramine in autism. Journal of child psychology and psychiatry, and allied disciplines. 1990;31(4):569–585

1. Stivaros 2018^369^

- Stivaros S, Garg S, Tziraki M, et al. Randomised controlled trial of simvastatin treatment for autism in young children with neurofibromatosis type 1 (SANTA). Molecular Autism. 22;9(190).

1. Sugie 2005^370–373^ *(crossover; no relevant data before the crossover)*

- Fukuda T, Sugie H, Ito M, Sugie Y. [Clinical evaluation of treatment with fluvoxamine, a selective serotonin reuptake inhibitor in children with autistic disorder]. No to Hattatsu [Brain & Development]. 2001;33(4):314–318.
- Fukuda T, Sugie H, Ito M, Sugie Y. Clinical evaluation of treatment with fluvoxamine, a selective serotonin reuptake inhibitor, in children with autistic disorder. [Japanese]. No to Hattatsu [Brain & Development]. 2001;33(4):314–318.
- Sugie Y, Sugie H, Fukuda T, Ito M, Ohzeki T. Serotonin 2A receptor gene polymorphism and clinical efficacy of fluvoxamine in children with autistic disorder. No to hattatsu = brain and development. 2003;35(1):23–28.
- Sugie Y, Sugie H, Fukuda T, et al. Clinical efficacy of fluvoxamine and functional polymorphism in a serotonin transporter gene on childhood autism. Journal of autism and developmental disorders. 2005;35(3):377–385

1. Sugiyama 1998^374, 375^ *(crossover; no relevant data before the crossover)*

- Sugiyama N, Sugie H, Igarashi Y, Ito M, Fukuda T. Low dose levodopa therapy of autistic disorder: Evaluation of clinical effectiveness. [Japanese]. No to Hattatsu [Brain & Development]. 1998;30(1):51–55.
- Sugiyama N, Sugie H, Igarashi Y, Ito M, Fukuda T. Low-dose levodopa therapy of autistic disorder: evaluation of clinical effectiveness. No to hattatsu = brain and development. 1998;30(1):51–55

1. Tordjman 2013^376–378^ *(unpublished protocol)*

- NCT01780883, Centre Hospitalier Guillaume Régnier, Rennes, Rennes University H. Melatonin Dose-effect Relation in Childhood Autism. 2013.
- Tordjman S, Kermarrec S, Cohen D, et al. MELADOSE: Study of the dose-response relationship for melatonin in childhood autism. [French] Meladose: Etude de la relation dose-effet de la melatonine dans l’autisme infantile. Neuropsychiatrie de l’Enfance et de l’Adolescence. 2013;61(7):415–416

1. UMIN000002650^379^ *(unpublished protocol)*

- JPRN-UMIN000002650, Department of Pediatrics Tohoku University School of Medicine National Institute of Mental Health: National Center of, Neurology, Psychiatry Yasuhara Children C, Department of Disaster Public, Health. Effects of vitamin B6 in children with autism: a randomized controlled trial. 2009.

1. UMIN000009075^380^ *(unpublished protocol)*

- JPRN-UMIN000009075, Osaka University H. Effects of long-term administration of intranasal oxytocin on autism spectrum disorders. 2012.

1. UMIN000017876^381^ *(unpublished protocol)*

- JPRN-UMIN000017876, United Graduate School of Child Development, Osaka University. Effects of long-term administration of intranasal oxytocin in children with autism spectrum disorder. 2015

1. Vasconcelos 2014^382, 383^ *(unpublished protocol, abstract only available; not usable data: medians presented without range of 20 participants, Hozo et al 2005)*

- RBR-7nq8m7, Brazil H. Steroid Therapy for Autistic Children. 2011.
- Vasconcelos MM, Brito AR, Vairo GT, et al. c(corticosteroids for autism - A scientific trial) study. Annals of Neurology. 2014;76:S189-s190

1. Veenstra-Vanderweele 2017^384–387^

- Kaufmann WE, Walton-Bowen KL, Kuriyama N, et al. Randomized, controlled, phase2 trial of STX209 (arbaclofen) for social function in autism spectrum disorder. Annals of Neurology. 2013;74:S129-s130.
- NCT01288716, Seaside Therapeutics I. Study of Arbaclofen for the Treatment of Social Withdrawal in Subjects With Autism Spectrum Disorders. 2011.
- NCT01706523, Seaside Therapeutics I. Open Label Extension Study of STX209 (Arbaclofen) in Autism Spectrum Disorders. 2012.
- Veenstra-V, erweele J, Cook EH, et al. Arbaclofen in Children and Adolescents with Autism Spectrum Disorder: a Randomized, Controlled, Phase 2 Trial. Neuropsychopharmacology. 2017;42(7):1390–1398

1. Voigt 2014^388, 389^

- NCT00577447, Dsm Nutritional Products I, Mayo C. Docosahexaenoic Acid in the Treatment of Autism. 2007.
- Voigt RG, Mellon MW, Katusic SK, et al. Dietary docosahexaenoic acid supplementation in children with autism. Journal of pediatric gastroenterology and nutrition. 2014;58(6):715–722

1. Wasserman 2006^390^ *(not relevant data; test statistics are reported without means)*

- Wasserman S, Iyengar R, Chaplin WF, et al. Levetiracetam versus placebo in childhood and adolescent autism: a double-blind placebo-controlled study. International Clinical Psychopharmacology. 2006;21(6):363–367.

1. Watanabe 2015^391–394^

- Benner S, Aoki Y, Watanabe T, et al. Neurochemical evidence for differential effects of acute and repeated oxytocin administration. Mol Psychiatry. 2018.
- JPRN-UMIN000007122, Showa University School of, Medicine, Department of Neuropsychiatry, Graduate School of Medicine University of Tokyo. A randomized, double-blind and cross-over trial to examine effects of continuous administration of intranasal oxytocin on social dysfunction in subjects with autism spectrum disorders. 2012.
- Owada K, Watanabe T, Kuroda M, et al. Development of quantitative facial expressions as a surrogate marker for autism spectrum disorder and oxytocin’s effect on it. Biological Psychiatry;1:124S-125S.
- Watanabe T, Kuroda M, Kuwabara H, et al. Clinical and neural effects of six-week administration of oxytocin on core symptoms of autism. Brain. 2015;138:3400–3412.

1. Willemsen-Swinkels 1996^395, 396^ *(crossover; no relevant data before the crossover)*

- Willemsen-Swinkels SH, Buitelaar JK, Weijnen FG, Engel, H. Placebo-controlled acute dosage naltrexone study in young autistic children. Psychiatry research. 1995;58(3):203–215.
- Willemsen-Swinkels SH, Buitelaar JK, Engel, H. The effects of chronic naltrexone treatment in young autistic children: a double-blind placebo-controlled crossover study. Biological Psychiatry. 1996;39(12):1023–1031

1. Wink 2016^397, 398^

- NCT00453180, National Alliance for Autism, Research, Indiana University School of, Medicine. A Study of Oral N-Acetylcysteine in Children With Autism Spectrum Disorders. 2007.
- Wink LK, Adams R, Wang Z, et al. A randomized placebo-controlled pilot study of N-acetylcysteine in youth with autism spectrum disorder. Molecular Autism. 2016;7:26.

1. Wright 2011^399, 400^ *(crossover; not relevant data before the crossover)*

- ISRCTN77884120, York Primary Care T, North Y. Melatonin treatment for sleep problems in children with autism: a randomised controlled crossover trial. 2008.
- Wright B, Sims D, Smart S, et al. Melatonin versus placebo in children with autism spectrum conditions and severe sleep problems not amenable to behaviour management strategies: a randomised controlled crossover trial. Journal of autism and developmental disorders. 2011;41(2):175–184.

1. Yamasue 2018^401, 402^

- JPRN-UMIN000015264, Kanazawa University Hospital, Nagoya University Hospital University of Fukui Hospital, Department of Neuropsychiatry, The University of Tokyo Hospital. A multicenter, parallel group, placebo-controlled, double blind, confirmatory trial of intranasal oxytocin in participants with autism spectrum disorders. 2014.
- Yamasue H, Okada T, Munesue T, et al. Effect of intranasal oxytocin on the core social symptoms of autism spectrum disorder: a randomized clinical trial. Molecular Psychiatry. 2018;29:1–10.

1. Yamasue 2018b^403, 404^ *(unpublished protocol)*

- JPRN-UMIN000031412, Hamamatsu University School of Medicine, Department of Psychiatry. An early phase II trial for efficacy and safety of TTA-121 on autism spectrum disorder. 2018.
- NCT03466671, Japan Agency for Medical, Research, Development, Hamamatsu U. A Trial of TTA-121 on Autism Spectrum Disorder. 2018

1. Yarbrough 1987^405^ *(crossover; not relevant data before the crossover)*

- Yarbrough E, Santat U, Perel I, Webster C, Lombardi R. Effects of fenfluramine on autistic individuals residing in a state developmental center. Journal of autism and developmental disorders. 1987;17(3):303–314.

1. Yatawara 2017^406, 407^

- ACTRN12611000061932, University University of S. Effects of Oxytocin on Social Behavior and Repetitive Behavior in Children with Autism. 2011.
- Yatawara CJ, Einfeld SL, Hickie IB, Davenport TA, Guastella AJ. The effect of oxytocin nasal spray on social interaction deficits observed in young children with autism: a randomized clinical crossover trial. Molecular Psychiatry. 2016;21(9):1225–1231.

1. Yui 2013^408–413^

- JPRN-JMA-IIA00041, Kunio Y. Therapeutic Effect of Dietary Omega-6 and Omega-3 Fatty Acids in Improving Social Impairment in Youth With Autism Spectrum Disorders: A Double-Blind, Randomized, Placebo-Controlled Trial. 2010.
- NCT01154894, Ashiya U. Dietary Fatty Acids Improve Social Impairment in Autism Spectrum Disorders. 2010.
- Yui K, Koshiba M, Nakamura S, Onishi M. [Therapeutic effects of larger doses of arachidonic acid added to DHA on social impairment and its relation to alterations of polyunsaturated fatty acids in individuals with autism spectrum disorders]. Nihon Shinkei Seishin Yakurigaku Zasshi. 2011;31(3):117–124.
- Yui K, Koshiba M, Nakamura S, Kobayashi Y. Effects of large doses of arachidonic acid added to docosahexaenoic acid on social impairment in individuals with autism spectrum disorders: a double-blind, placebo-controlled, randomized trial. Journal of Clinical Psychopharmacology. 2012;32(2):200–206.
- Yui K, Koshiba K, Nakamura S. Effects of adding large doses of arachidonic acid to docosahexaenoic acid on social impairment in individuals with autism spectrum disorders. Current psychopharmacology. 2013;2(1):84–90.
- Yui K, Murphy D, Hamakawa H. [Effects of arachidonic acids on social behavior in patients with autism spectrum disorders]. Seishin Shinkeigaku Zasshi - Psychiatria et Neurologia Japonica. 2009;111(11):1387–1396.

1. Zimmerman 2018^414, 415^ *(unpublished protocol)*

- NCT02561481, Congressionally Directed Medical Research, Programs, Johns Hopkins U, University of Massachusetts W. Sulforaphane Treatment of Children With Autism Spectrum Disorder (ASD). 2015.
- Zimmerman A, Diggins E, Connors S, Singh K. Sulforaphane treatment of children with autism spectrum disorder (ASD) - A progress report. Neurology. Conference: 70th annual meeting of the american academy of neurology, AAN 2018. United states. 2018;90(15)

### 2.2. Ongoing trials

1. EUCTR2010-018740-13-NL

- EUCTR2010-018740-13-NL, University Medical Center G. Short- and long-term effects of oxytocin on empathy and social behaviour in autistic and antisocial male adults. - Oxytocin effects in autistic and antisocial male adults. 2010.

1. EUCTR2014-001560-35-NL

- EUCTR2014-001560-35-NL, Brain Center Rudolf Magnus, University Medical Center Utrecht Department of Psychiatry Utrecht the Netherl, S. Bumetanide for Autism Treatment Study. 2016.

1. EUCTR2017-004419-38-NL

- EUCTR2017-004419-38-NL, Institut de Recherches Internationales, Servier. Efficacy and safety of bumetanide oral liquid formulation in children and adolescents aged from 7 to less than 18 years old with Autism Spectrum Disorder. 2018.

1. EUCTR2017-004420-30-NL

- EUCTR2017-004420-30-NL, Institut de Recherches Internationales, Servier. Efficacy and safety of bumetanide oral liquid formulation in children aged from 2 to less than 7 years old with Autism Spectrum Disorder. 2018

1. Häge 2016

- EUCTR2014-003080-38-DE, Behaviour, Radboud University Nijmegen Medical Centre, Donders Institute for Brain Cognition. Glutamatergic medication in the treatment of Obsessive Compulsive Disorder (OCD) and Autism Spectrum Disorder (ASD). 2014.
- Häge A, Banaschewski T, Buitelaar JK, et al. Glutamatergic medication in the treatment of obsessive compulsive disorder (OCD) and autism spectrum disorder (ASD) - study protocol for a randomised controlled trial. Trials. 2016;17(1):141.

6. NCT01260961

- NCT01260961, Rutgers, The State University of New Jersey. Developing Treatment, Treatment Validation and Treatment Scope in the Setting of an Autism Clinical Trial. 2010

7. NCT01813318

- NCT01813318, Autism S, Children’s Hospital Medical Center, Cincinnati. Study of Acamprosate in Autism. 2013.

8. NCT01970345

- NCT01970345, Autism Science F, Icahn School of Medicine at Mount, Sinai. A Pilot Treatment Study of Insulin-Like Growth Factor-1 (IGF-1) in Autism Spectrum Disorder. 2013.

9. NCT02487082

- NCT02487082, Korea Institute of S, Technology, Stony Brook U. Pilot Study of Sleep Therapy and Biomarkers in Children With Autism Spectrum Disorders. 2015.

10. NCT02627508

- NCT02627508, Simons F, Stanford U. Pilot Trial of Pregnenolone in Autism. 2015

11. NCT02674984

- NCT02674984, Texas Higher Education Control, Board, The University of Texas Health Science Center, Houston. Road to Discovery for Combination Probiotic BB-12 With LGG in Treating Autism Spectrum Disorder. 2015

12. NCT02677051

- NCT02677051, Rowan U, Rutgers, The State University of New Jersey. Sulforaphane in a New Jersey (NJ) Population of Individuals With Autism. 2016.

13. NCT02839915

- NCT02839915, Phoenix Children’s H, Harvard U, Emory U. Folinic Acid and Language Impairment in Autism Spectrum Disorder. 2016.

1. NCT02871349

- NCT02871349, University of M-C. Trial of Propranolol in Children and Youth With Autism Spectrum Disorder and Predictors of Response. 2016.

15. NCT02879110

- NCT02879110, Davis family f, University of C, University of Illinois at, Chicago, Central South U. A 12-weeks Study to Evaluate Sulforaphane in Treatment of Autism Spectrum Disorder. 2016.

16. NCT02901431

- NCT02901431, Hoffmann-La R. A Study to Investigate the Efficacy and Safety of RO5285119 in Participants With Autism Spectrum Disorder (ASD). 2016.

17. NCT03008889

- NCT03008889, Emory U. A Feasibility Study of N-acetylcysteine for Self-injurious Behavior in Children With Autism Spectrum Disorder. 2016.

18. NCT0315615356

- NCT03156153, Shanghai Jiao Tong University School of, Medicine, Xinhua Hospital, Shanghai Jiao Tong University School of Medicine. A Study of Bumetanide for the Treatment of Autism Spectrum Disorders. 2017.
- ChiCTR-IPR-16009627, Xinhua Hospital Affiliated to Shanghai Jiaotong University School of, Medicine. A randomized double-blind placebo-controlled trial of the efficiency and mechanism of bumetanide on children with Autism Spectrum Disorder. 2016.

19. NCT03202303271

- NCT03202303, United States Department of, Defense, Eric H, er. Cannabidivarin (CBDV) vs. Placebo in Children With Autism Spectrum Disorder (ASD). 2017.

20. NCT03204786

- NCT03204786, Eunice Kennedy Shriver National Institute of Child, Health, Human D, Stanford U. Intranasal Vasopressin Treatment in Children With Autism. 2017

21. NCT03279471

- NCT03279471, University of California D. Specifying and Treating Anxiety in Autism Research. 2017.

22. NCT03337035

- NCT03337035, Massachusetts General H. Probiotics and Oxytocin Nasal Spray on Social Behaviors of Autism Spectrum Disorder (ASD) Children. 2017.

23. NCT03369431

- NCT03369431, University College L. Efficacy of Vivomixx on Behaviour and Gut Function in Autism Spectrum Disorder. 2017

24. NCT03434366

- NCT03434366, Children’s Medical C, Guangzhou W. Intranasal Ketamine With Dexmedetomidine for the Treatment of Children With Autism Spectrum Disorder. 2018.

25. NCT03487770

- NCT03487770, Otsuka Beijing Research I. Aripiprazole Oral Solution in the Treatment of Children and Adolescents With Autistic Disorder. 2018.

26. NCT03504917

- NCT03504917, Hoffmann-La R. A Study of Balovaptan in Adults With Autism Spectrum Disorder With a 2-Year Open-Label Extension. 2018.

27. NCT03514784

- NCT03514784, The University of Texas Health Science Center, Houston. Combination Probiotic: BB-12 With LGG (Different Doses) in Treating Children With Autism Spectrum Disorder. 2018.

28. NCT03550209

- NCT03550209, National Center for C, Integrative H, Sarah K. Fatty Acid Supplementation in Children With ASD. 2018.

## 3. Contacting corresponding authors for additional data/clarifications

We tried to contact all corresponding authors of eligible or unclear studies (k=211 eligible and ongoing, eight excluded and 11 remained unclear). We did not contact old trials published up to 1990 (k=13) and protocols with an ongoing status (k=28). From the rest of the studies (k=170), authors of 144 could be contacted, with a reminder e-mail in case of no response, while for the 26 a working e-mail of the corresponding author could not be found. Of the 144, 51% of the authors replied with 17% providing additional data or clarifications.

|  | **Study name** | **In the review** | **Contacted** | **Replied** | **Provided Data** | **Comment (up to 10.10.2019)** |
| --- | --- | --- | --- | --- | --- | --- |
| 1 | ACTRN12613000334707 | unpublished protocol/abstract | yes | no | no |  |
| 2 | ACTRN12617000441314 | unpublished protocol/abstract | yes | no | no |  |
| 3 | Akkok 1995 | included in the analysis | yes | yes | no |  |
| 4 | Aliyev_2018a | included in the analysis | yes | yes | yes |  |
| 5 | Aliyev_2018b | included in the analysis | yes | yes | yes |  |
| 6 | Aman 2017 | included in the analysis | yes | yes | no |  |
| 7 | Amminger 2008 | included in the analysis | yes | no | no |  |
| 8 | Anagnostou 2012 | included in the analysis | yes | yes | no |  |
| 9 | Anderson 1984 | crossover aggregated data | no | no | no | Up to 1989 |
| 10 | Anderson 1989 | included in the analysis | no | no | no | Up to 1989 |
| 11 | Arnold 2006 | included in the analysis | yes | yes | yes |  |
| 12 | Arnold 2012 | included in the analysis | yes | yes | yes |  |
| 13 | Arnold_2019 | included in the analysis | yes | yes | yes |  |
| 14 | August 1987 | crossover aggregated data | no | no | no | Up to 1989 |
| 15 | Ballester 2015 | crossover aggregated data | yes | no | no |  |
| 16 | Barthelemy 1989 | crossover aggregated data | no | no | no | Up to 1989 |
| 17 | Belsito 2001 | included in the analysis | yes | yes | yes |  |
| 18 | Bent 2011 | included in the analysis | yes | yes | yes |  |
| 19 | Bent 2014 | included in the analysis | yes | yes | yes |  |
| 20 | Bernaets 2017 | unpublished protocol/abstract | yes | yes | no | Submitted for publication |
| 21 | Bertoglio 2010 | crossover aggregated data | yes | yes | yes |  |
| 22 | Bolman 1999 | crossover aggregated data | no | no | no | Contact information of corresponding author could not be found |
| 23 | Bolognani_2019 | included in the analysis | yes | yes | yes |  |
| 24 | Bouvard 1995 | crossover aggregated data | no | no | no | E-mail of corresponding author was not in use |
| 25 | Buitelaar 1990 | crossover aggregated data | yes | yes | no |  |
| 26 | Buitelaar 1992 | crossover aggregated data | yes | yes | no |  |
| 27 | Campbell 1987 | included in the analysis | no | no | no | Up to 1989 |
| 28 | Campbell 1990 | included in the analysis | no | no | no | Contact information of corresponding author could not be found |
| 29 | Chez 2003 | excluded | yes | yes | no |  |
| 30 | Chez 2017 | crossover aggregated data | yes | yes | yes |  |
| 31 | Chugani 2016 | included in the analysis | yes | no | no |  |
| 32 | Cohen 1980 | crossover aggregated data | no | no | no | Up to 1989 |
| 33 | Cortesi 2012 | not usable outcomes | yes | no | no |  |
| 34 | Danfors 2005 | crossover aggregated data | yes | yes | no |  |
| 35 | Dean 2017 | included in the analysis | yes | no | no |  |
| 36 | Duker 1991 | crossover aggregated data | no | no | no | Contact information of corresponding author could not be found |
| 37 | EFFET 2015 | unpublished protocol/abstract | yes | yes | no | Ongoing study |
| 38 | Ekman 1989 | included in the analysis | no | no | no | Up to 1989 |
| 39 | EUCTR2006-006126-25-FR | unclear | yes | yes | no |  |
| 40 | EUCTR2009-009475-35-NL | unclear | no | no | no | Contact information of corresponding author could not be found |
| 41 | EUCTR2010-018740-13-NL | unpublished protocol/abstract | no | no | no | Ongoing study |
| 42 | EUCTR2014-001560-35-NL | unpublished protocol/abstract | no | no | no | Ongoing study |
| 43 | EUCTR2017-004419-38-NL | unpublished protocol/abstract | no | no | no | Ongoing study |
| 44 | EUCTR2017-004420-30-NL | unpublished protocol/abstract | no | no | no | Ongoing study |
| 45 | Fahmy 2013 | included in the analysis | yes | yes | no |  |
| 46 | Findling 1997 | crossover aggregated data | yes | no | no |  |
| 47 | Frye 2018 | included in the analysis | no | no | no | E-mail of corresponding author was not in use |
| 48 | Gabis 2019 | included in the analysis | yes | yes | no |  |
| 49 | Garstang 2006 | excluded | yes | no | no |  |
| 50 | Geier 2011 | included in the analysis | yes | no | no |  |
| 51 | Ghodsi 2018 | not usable outcomes | yes | yes | no |  |
| 52 | Ghuman 2009 | crossover aggregated data | yes | no | no |  |
| 53 | Gordon 1993 | crossover aggregated data | no | no | no | E-mail of corresponding author was not in use |
| 54 | Grimaldi 2018 | excluded | yes | yes | no |  |
| 55 | Gringras 2017 | not usable outcomes | yes | yes | yes |  |
| 56 | Guastella 2015 | included in the analysis | yes | no | no |  |
| 57 | Häge 2016 | unpublished protocol/abstract | yes | yes | no | Analysis ongoing |
| 58 | Handen 2000 | crossover aggregated data | yes | yes | no |  |
| 59 | Handen 2009 | included in the analysis | yes | yes | no |  |
| 60 | Handen 2012 | included in the analysis | yes | yes | no |  |
| 61 | Handen 2015 | included in the analysis | yes | yes | no |  |
| 62 | Hardan 2012 | included in the analysis | yes | yes | no |  |
| 63 | Harfterkamp 2013 | included in the analysis | yes | no | no |  |
| 64 | Hellings 2005 | included in the analysis | no | no | no | E-mail of corresponding author was not in use |
| 65 | Hellings 2006 | crossover aggregated data | no | no | no | E-mail of corresponding author was not in use |
| 66 | Hendren 2016 | included in the analysis | yes | yes | no |  |
| 67 | Herscu 2019 | included in the analysis | yes | yes | no |  |
| 68 | Hollander 2005 | included in the analysis | yes | no | no |  |
| 69 | Hollander 2005b | included in the analysis | yes | no | no |  |
| 70 | Hollander 2006b | included in the analysis | yes | no | no |  |
| 71 | Hollander 2010 | included in the analysis | yes | no | no |  |
| 72 | Hollander 2012 | included in the analysis | yes | no | no |  |
| 73 | Ichikawa 2017 | included in the analysis | yes | no | no |  |
| 74 | IRCT2012111011421N1 | unclear | yes | no | no |  |
| 75 | IRCT20131013014994N5 | unpublished protocol/abstract | yes | no | no |  |
| 76 | IRCT2015122625699N1 | unclear | yes | yes | no |  |
| 77 | ISRCTN04516575 | unpublished protocol/abstract | yes | no | no |  |
| 78 | ISRCTN20233876 | unpublished protocol/abstract | no | no | no | Contact information of corresponding author could not be found |
| 79 | Kanmani 2018 | unclear | yes | no | no |  |
| 80 | Kent 2013 | included in the analysis | no | no | no | E-mail of corresponding author was not in use |
| 81 | Kerley 2017 | included in the analysis | yes | yes | no |  |
| 82 | Kern 2001 | included in the analysis | yes | yes | no |  |
| 83 | King 2001 | included in the analysis | yes | no | no |  |
| 84 | King 2009 | included in the analysis | yes | no | no |  |
| 85 | Klaiman 2013 | included in the analysis | yes | yes | no |  |
| 86 | Kolmen 1997 | crossover aggregated data | no | no | no | Contact information of corresponding author could not be found |
| 87 | Kosaka 2016 | included in the analysis | yes | no | no |  |
| 88 | Lemonnier 2012 | included in the analysis | yes | yes | yes |  |
| 89 | Lemonnier 2017 | included in the analysis | yes | yes | yes |  |
| 90 | Leventhal 1993 | crossover aggregated data | yes | yes | no |  |
| 91 | Levine 1997 | included in the analysis | yes | yes | yes |  |
| 92 | Liu_2019 | included in the analysis | yes | yes | yes |  |
| 93 | Loebel 2016 | included in the analysis | no | no | no | E-mail of corresponding author was not in use |
| 94 | Malone 2010 | unpublished protocol/abstract | no | no | no | Corresponding author no longer available |
| 95 | Mankad 2015 | included in the analysis | yes | yes | no |  |
| 96 | Marcus 2009 | included in the analysis | yes | no | no |  |
| 97 | Martineuau 1985 | crossover aggregated data | no | no | no | Up to 1989 |
| 98 | Martsenkovska 2015 | unpublished protocol/abstract | yes | no | no | Difficult to identify e-mail, authors contacted also via researchgate |
| 99 | Martsenkovsky 2016 | unpublished protocol/abstract | yes | no | no | Difficult to identify e-mail, authors contacted also via researchgate |
| 100 | Mazahery 2016 | included in the analysis | yes | no | no |  |
| 101 | McDougle 1996 | included in the analysis | yes | no | no |  |
| 102 | McDougle 1998 | included in the analysis | yes | no | no |  |
| 103 | McDougle_2000unpublished fluvoxamine | unpublished protocol/abstract | yes | no | no |  |
| 104 | Mehrazad-Saber 2018 | included in the analysis | yes | yes | no |  |
| 105 | Moradi 2018 | not usable outcomes | yes | no | no |  |
| 106 | Munasinghe 2010 | crossover aggregated data | no | no | no | E-mail of corresponding author was not in use |
| 107 | Munesue 2016 | included in the analysis | no | no | no | No missing information |
| 108 | Nagaraj 2006 | included in the analysis | no | no | no | E-mail of corresponding author was not in use |
| 109 | NCT00036231 | unclear | yes | no | no |  |
| 110 | NCT00036244 | unclear | yes | no | no |  |
| 111 | NCT00036244 | unclear | yes | no | no |  |
| 112 | NCT00054730 | unclear | yes | yes | no |  |
| 113 | NCT00057408 | unpublished protocol/abstract | no | no | no | Corresponding author no longer available |
| 114 | NCT00166621 | unpublished protocol/abstract | yes | no | no |  |
| 115 | NCT00183339 | not usable outcomes | yes | yes | no |  |
| 116 | NCT00183404 | unpublished protocol/abstract | no | no | no | Corresponding author no longer available |
| 117 | NCT00198107 | included in the analysis | yes | no | no |  |
| 118 | NCT00198120 | unpublished protocol/abstract | yes | no | no |  |
| 119 | NCT00252603 | unpublished protocol/abstract | yes | no | no |  |
| 120 | NCT00318162 | excluded | yes | yes | no | The study was not conducted |
| 121 | NCT00325572 | excluded | yes | yes | no | RCT was not conducted |
| 122 | NCT00467818 | unpublished protocol/abstract | yes | no | no |  |
| 123 | NCT00468130 | unpublished protocol/abstract | yes | no | no |  |
| 124 | NCT00498173 | included in the analysis | yes | no | no |  |
| 125 | NCT00572741 | unpublished protocol/abstract | no | no | no | E-mail of corresponding author was not in use |
| 126 | NCT00609531 | included in the analysis | yes | no | no |  |
| 127 | NCT00655174 | unpublished protocol/abstract | yes | yes | no | Data difficult to be found |
| 128 | NCT00672360 | unpublished protocol/abstract | no | no | no | No contact information |
| 129 | NCT00870727 | included in the analysis | yes | yes | no |  |
| 130 | NCT00881452 | unpublished protocol/abstract | yes | no | no | LE Arnold suggested to contact Curemark; Contacted but no reply |
| 131 | NCT00927030 | excluded | yes | yes | no |  |
| 132 | NCT00936182 | excluded | yes | yes | no | The study was not conducted |
| 133 | NCT01171937 | unpublished protocol/abstract | yes | yes | no | Submitted for publication |
| 134 | NCT01230359 | unpublished protocol/abstract | no | no | no | E-mail of corresponding author could not be found |
| 135 | NCT01260961 | unpublished protocol/abstract | yes | no | no | Ongoing; not recruiting |
| 136 | NCT01302964 | included in the analysis | yes | no | no |  |
| 137 | NCT01366859 | unpublished protocol/abstract | yes | yes | no | Submitted for publication |
| 138 | NCT01372449 | unpublished protocol/abstract | yes | yes | no | Submitted for publication |
| 139 | NCT01661855 | included in the analysis | yes | yes | no | Expected in three months |
| 140 | NCT01745497 | unpublished protocol/abstract | yes | no | no |  |
| 141 | NCT01788072 | unpublished protocol/abstract | yes | yes | no | Expected in three months |
| 142 | NCT01813318 | unpublished protocol/abstract | no | no | no | Ongoing study |
| 143 | NCT01908205 | unpublished protocol/abstract | yes | yes | no | Expected in three months |
| 144 | NCT01944046 | unpublished protocol/abstract | yes | yes | no |  |
| 145 | NCT01966679 | unpublished protocol/abstract | yes | yes | no | Submitted for publication |
| 146 | NCT01970345 | unpublished protocol/abstract | no | no | no | Recruiting |
| 147 | NCT01972074 | unpublished protocol/abstract | yes | no | no | Results published in September 11, 2019 |
| 148 | NCT02007447 | excluded | yes | yes | no |  |
| 149 | NCT02222285 | unpublished protocol/abstract | yes | no | no |  |
| 150 | NCT02385799 | included in the analysis | yes | no | no |  |
| 151 | NCT02410902 | unpublished protocol/abstract | yes | no | no |  |
| 152 | NCT02487082 | unpublished protocol/abstract | no | no | no | Recruiting |
| 153 | NCT02550912 | unpublished protocol/abstract | yes | yes | no |  |
| 154 | NCT02586935 | unpublished protocol/abstract | yes | yes | no | Expected in three months |
| 155 | NCT02627508 | unpublished protocol/abstract | no | no | no | Recruiting |
| 156 | NCT02674984 | unpublished protocol/abstract | no | no | no | Recruiting |
| 157 | NCT02677051 | unpublished protocol/abstract | no | no | no | Recruiting |
| 158 | NCT02757066 | unpublished protocol/abstract | no | no | no | Recently completed 16.04.2019; e-mail not found |
| 159 | NCT02839915 | unpublished protocol/abstract | no | no | no | Not yet recruiting |
| 160 | NCT02871349 | unpublished protocol/abstract | no | no | no | Recruiting |
| 161 | NCT02879110 | unpublished protocol/abstract | no | no | no | Recently completed 30.07.2019 |
| 162 | NCT02901431 | unpublished protocol/abstract | no | no | no | Recruiting |
| 163 | NCT02909959 | unpublished protocol/abstract | yes | yes | no | Results will be published in six months |
| 164 | NCT02947048 | unpublished protocol/abstract | yes | no | no |  |
| 165 | NCT02956226 | unpublished protocol/abstract | yes | yes | no | Not accepted for publication yet |
| 166 | NCT03008889 | unpublished protocol/abstract | yes | yes | no | Not yet completed |
| 167 | NCT03156153 | unpublished protocol/abstract | yes | yes | no | Not yet unblinded |
| 168 | NCT03202303 | unpublished protocol/abstract | no | no | no | Recruiting |
| 169 | NCT03204786 | unpublished protocol/abstract | no | no | no | Recruiting |
| 170 | NCT03279471 | unpublished protocol/abstract | no | no | no | Recruiting |
| 171 | NCT03337035 | unpublished protocol/abstract | no | no | no | Ongoing study |
| 172 | NCT03369431 | unpublished protocol/abstract | no | no | no | Ongoing study |
| 173 | NCT03434366 | unpublished protocol/abstract | no | no | no | Ongoing study |
| 174 | NCT03487770 | unpublished protocol/abstract | no | no | no | Recruiting |
| 175 | NCT03504917 | unpublished protocol/abstract | no | no | no | Recruiting |
| 176 | NCT03514784 | unpublished protocol/abstract | no | no | no | Not yet recruiting |
| 177 | NCT03550209 | unpublished protocol/abstract | no | no | no | Enrollling participants |
| 178 | NCT03553875 | unpublished protocol/abstract | yes | no | no |  |
| 179 | Niederhofer 2002 | crossover aggregated data | yes | no | no | Difficult to identify a working e-mail |
| 180 | Niederhofer 2002b | crossover aggregated data | yes | no | no | Difficult to identify a working e-mail |
| 181 | Niederhofer 2003 | included in the analysis | yes | no | no | Difficult to identify a working e-mail |
| 182 | Niederhofer 2004 | crossover aggregated data | yes | no | no | Difficult to identify a working e-mail |
| 183 | Noone 2014 | unpublished protocol/abstract | yes | no | no |  |
| 184 | NTR6325 | unclear | yes | no | no |  |
| 185 | Owen 2008 | included in the analysis | yes | no | no |  |
| 186 | Parellada 2017 | included in the analysis | yes | yes | yes |  |
| 187 | Parker 2017 | included in the analysis | yes | no | no |  |
| 188 | Pearson 2013 | crossover aggregated data | yes | yes | no |  |
| 189 | Pusponegoro 2015 | included in the analysis | yes | no | no |  |
| 190 | Quintana 1995 | crossover aggregated data | no | no | no | E-mail of corresponding author was not in use |
| 191 | Ratliff 2005 | crossover aggregated data | no | no | no | E-mail of corresponding author was not in use |
| 192 | Realmuto 1986 | crossover aggregated data | no | no | no |  |
| 193 | Reddihough 2019 | included in the analysis | yes | yes | no | Under publication |
| 194 | Remington 2001 | crossover aggregated data | yes | yes | no |  |
| 195 | RISAUT-JPN | included in the analysis | yes | yes | no | CSR of the study was found; provided by YODAS after conducted, no further data |
| 196 | RUPP 2002 | included in the analysis | yes | yes | yes |  |
| 197 | RUPP 2005 | crossover aggregated data | yes | no | no |  |
| 198 | Saad 2015 | included in the analysis | yes | no | no |  |
| 199 | Saad 2018 | retraction due to data reliability issues | yes | no | no |  |
| 200 | Santocchi 2016 | unpublished protocol/abstract | yes | yes | no | Under publication |
| 201 | Scahill 2015 | included in the analysis | yes | yes | yes |  |
| 202 | Scifo 1991 | crossover aggregated data | yes | yes | yes |  |
| 203 | Shea 2004 | included in the analysis | yes | yes | yes |  |
| 204 | Sherman 1989 | crossover aggregated data | no | no | no | Up to 1989 |
| 205 | Sikich 2013 | included in the analysis | yes | yes | no |  |
| 206 | Singh 2014 | included in the analysis | yes | yes | no | A Zimmerman cc'ed Singh; no reply |
| 207 | SOMELIA 2015 | unpublished protocol/abstract | yes | no | no |  |
| 208 | Stern 1990 | crossover aggregated data | no | no | no | E-mail of corresponding author was not in use |
| 209 | Stigler et al | unclear | no | no | no | E-mail of corresponding author was not in use |
| 210 | Stivaros 2018 | included in the analysis | yes | yes | no |  |
| 211 | Sugie 2005 | crossover aggregated data | yes | no | no |  |
| 212 | Sugiyama 1998 | crossover aggregated data | yes | no | no | E-mail of the first author was not provided, Sugie was conducted no reply |
| 213 | Tordjman 2013 | unpublished protocol/abstract | yes | no | no |  |
| 214 | UMIN000002650 | unpublished protocol/abstract | no | no | no | E-mail of corresponding author was not in use |
| 215 | UMIN000009075 | unpublished protocol/abstract | yes | no | no |  |
| 216 | UMIN000017876 | unpublished protocol/abstract | yes | no | no |  |
| 217 | Vasconcelos 2014 | unpublished protocol/abstract | yes | yes | no | Submitted for publication |
| 218 | Veenstra-Vanderweele 2017 | included in the analysis | yes | yes | yes |  |
| 219 | Voigt 2014 | included in the analysis | yes | no | no |  |
| 220 | Wasserman 2006 | not usable outcomes | no | no | no | E-mail of corresponding author was not in use |
| 221 | Watamabe 2015 | included in the analysis | yes | yes | yes |  |
| 222 | Willemsen-Swinkels 1996 | crossover aggregated data | yes | yes | no |  |
| 223 | Wink 2016 | included in the analysis | yes | no | no |  |
| 224 | Wright 2011 | crossover aggregated data | no | no | no | E-mail of corresponding author was not in use |
| 225 | Yamasue 2018 | included in the analysis | yes | yes | yes |  |
| 226 | Yamasue 2018b | crossover aggregated data | yes | yes | no | Ongoing study |
| 227 | Yarbrough 1987 | crossover aggregated data | no | no | no |  |
| 228 | Yatawara 2017 | included in the analysis | yes | no | no |  |
| 229 | Yui 2013 | included in the analysis | yes | no | no |  |
| 230 | Zimmerman 2018 | unpublished protocol/abstract | yes | yes | no |  |

# eAppendix-5 Study chracteristics

1. Tables of study characteristics 120

1.1. Summary of characteristics of the sample 120

1.2. Characteristics of individual studies 121

2. Risk of bias 122

2.1 Risk of bias of included studies 122

2.2. Risk of bias summary of included studies 125

3. Eligible scales 126

3.1 General strategy 126

3.2. Table of eligible scales 126

4. Covariate data 128

4.1. Missing covariate data in included studies 128

4.2. Correlation matrix with the bivariate Spearman’ correlations between covariates 129

5. References 131

## 1. Tables of study characteristics

### 1.1. Summary of characteristics of the sample

|  |  | 86 studies, overall sample size 5365 with 2360 participants on placebo |
| --- | --- | --- |
| Study design | Publication year | 2014 [2009-2017], min/max = 1988-2019 |
|  | Design | Parallel (k=73), crossover (k=13)*  Double blind (k=85), single-blind (k=1) |
|  | Counties | Australia (k=4), Austria (k=2), Azerbaijan (k=2), Canada (k=3), Egypt (k=2), France (k=2), France (k=1), India (k=1), Indonesia (k=1), Iran (k=1), Ireland (k=1), Israel (k=1), Japan (k=7), Netherlands (k=1), New Zealand (k=1), Spain (k=1), Sweden (k=1), Taiwan (k=1), Turkey (k=1), UK (k=1), USA (k=51) |
|  | Sites | Single site (k=47)  Number of sites: 1 [1-4], min/max 1-50, n.i. or unclear (k=8)  Only academic sites (k=59), for the rest of the studies the percentage of academic sites ranged between 0%-66.7%, n.i. or unclear (k=15) |
|  | Duration of treatment | 10 weeks [8-12], min/max 1-52 weeks  Longer-term studies (12 or more weeks) (k=41) |
|  | Washout and placebo lead-in | Use of washout period from psychotropic drugs (k=41), n.i. (k=11); there was inconsistency in the definition, duration and allowance of stable medication.  Use of a placebo lead-in period with exclusion of placebo responders (k=5). Definition of response and duration varied. |
|  | Number of arms and medications | Two arms (k=76), three arms (k=4), four arms (k=6),  All of the studies investigated two medications (including placebo and different doses) apart from one study which investigated four medications (placebo, PUFA, vitamin-D, vitamin-D + PUFA) |
|  | Sample size, percentage on participants on placebo | 45 [30-91], min/max 10-223,  The percentage of participants treated with placebo was 48.7% [44-50.5%], min/max 23.9-66.7% |
| Intervention | Experimental intervention | Pharmacological (k=61):  amantadine (k=1), arbaclofen (k=1), aripiprazole (k=5), atomoxetine (k=4, including one study with atomoxetine + parental training and parental training arms), balovaptan (k=1), bumetanide (k=2), buspirone (k=1), citalopram (k=2), divalproex (k=2), donepezil (k=2, including one study with donepezil + choline), fenfluremine (k=2), fluoxetine (k=4), fluvoxamine (k=1), guanfacine (k=1), haloperidol (k=1), IGOH (k=1), lamotrigine (k=1), levetiracetam (k=1), lurasidone (k=1), mecamylamine (k=1), memantine (k=1), mirtazapine (k=1), naltrexone (k=2), olanzapine (k=1), oxytocin (k=9), riluzole (k=1), risperidone (k=6), sertraline (k=1), simvastatin (k=1), tianeptine (k=1), valproate (k=2)  Dietary supplements (k=25):  carnitine/carnosine (k=3), digestive enzymes (k=1), dimethylglycine (k=1), folinic acid (k=1), gluten-casein supplement (k=1), inositol (k=1), n-acetylcysteine (k=2), pre/probiotics (k=2), PUFA (k=8, including one study that investigated vitamin D and a combination of PUFA and vitamin D), saproptertin (k=1), sulforaphane (k=1), vitamin-B12 (k=1), vitamin-D (k=2, including one study that investigated PUFA and a combination of PUFA and vitamin D) |
|  | Dose administration | Flexible schedule (k=41), fixed (k=43), n.i. (k=2) |
|  | Route of administration | Oral (k=76), intranasal (k=9), subcutaneous (k=1) |
| Participants | Diagnosis | Standardized diagnostic criteria ((k=81):  DSM-III (k=7), DSM-IV (k=60), DSM-5 (k=9), ICD-10 (k=5), DSM-version n.i. (k=2)  Studies that used only diagnostic tools (k=5):  ADI-R + ADOS (k=2), CARS (k=2), SCQ + clinical diagnosis (k=1, validation of the method was reported**) |
|  | Age | Children/adolescents (k=75), adults (k=8), mixed (k=3)  8.63 years [6.55-10.16], min/max 3.5-38 years, n.i. (k=4) |
|  | Sex | Percentage of female participants was 17.2% [10-20.9%], min/max 0-75%, n.i. (k=6) |
|  | Ethnicity | Percentage of Caucasian or Hispanic participants was 66.8% [55.1-81.5%], min/max 0-100%, n.i. (k=39) |
|  | Intellectual disability | Percentage of participants with intellectual impairment was 54.2% [0-75%], min/max 0-100%, n.i. (k=56).  The IQ was 76.10 [62.62-93.14], min/max 37.5-118, n.i. (k= 53). Different scales and versions were used within and between studies. |
|  | Associated symptoms | Associated symptoms as inclusion criteria (k=29): Irritability (k=14), ADHD symptoms (k=6), irritability and hyperactivity (k=1), other subgroup of conditions or symptoms (k=8).  Genetic syndrome as inclusion criteria (k=1, neurofibromatosis type 1) |
|  | BMI | 18.96 [18.25-19.84], min/max 16.27-23.7, n.i. (k=72) |
|  | Baseline severity | Baseline CGI-Severity 4.73[4.39-5], min/max 3.88-6.06, n.i. (k=48)  Baseline ABC-Irritability 17.18 [13.71-22.70], min/max 10.2-30.2, n.i, (k=50)  Minimum threshold of ASD core symptoms for inclusion (k=9): SRS (k=1), ABC-L/SW (k=2), CARS (k=1), C-YBOCS, C-YBOCS-PDD or Y-BOCS (k=5)*** |

The median of values are presented (of summary data), [] = interquartile ranges, min/max =minimum and maximum values are presented, k= number of studies, n.i. = not indicated or unclear, IGOH= oral human immunoglobulin

*only crossover studies with available data before the crossover

**Lee H, Marvin AR, Watson T, et al. Accuracy of phenotyping of autistic children based on Internet implemented parent report. Am J Med Genet B Neuropsychiatr Genet. 2010;153B(6):1119-1126.

***When scales used only for the confirmation of a diagnosis (such as SRS T-score of 60, CARS score of 30) were not considered as a minimum threshold of symptoms.

### 1.2. Characteristics of individual studies

Detailed characteristics of study characteristics are reported in [Table-S](file:///\\nas.ads.mwn.de\ge73tul\AIMS-2\analysis\placebo_response\manuscript_placebo_response\manuscript_v2\Table_S_25092019.xlsx).

## 2. Risk of bias

### 2.1 Risk of bias of included studies

| **Study** | **Sequence generation** | **Allocation concealment** | **Blinding of participants and personnel** | **Blinding of outcome assessment** | **Missing outcome data** | **Selective reporting** | **Other biases** | **Overall** |
| --- | --- | --- | --- | --- | --- | --- | --- | --- |
| Akkok_1995 | Unclear | Unclear | High | Unclear | Low | Low | Unclear | Moderate |
| Aliyev_2018 | Low | Low | Low | Low | Unclear | High | Unclear | Moderate |
| Aliyev_2018b | Low | Low | Low | Low | Unclear | High | Unclear | Moderate |
| Aman_2017 | Low | Low | Unclear | Unclear | Low | Low | Unclear | Low |
| Amminger_2007 | Unclear | Unclear | Low | Low | High | Low | High | High |
| Anagnostou_2012 | Low | Low | Unclear | Unclear | Low | Low | Low | Low |
| Anderson_1989 | Unclear | Unclear | Low | Low | Unclear | Unclear | Unclear | Moderate |
| Arnold_2006 | Unclear | Unclear | Low | Low | Low | High | Unclear | Moderate |
| Arnold_2012 | Low | Low | Unclear | Unclear | Low | Low | High | Moderate |
| Arnold_2019 | Low | Low | Low | Low | High | Low | Low | Moderate |
| Belsito_2001 | Low | Low | Low | Low | High | High | Unclear | High |
| Bent_2011 | Low | Low | Low | Low | Low | Low | Low | Low |
| Bent_2014 | Low | Low | Low | Low | Low | Low | Unclear | Low |
| Bolognani_2019 | Low | Low | Low | Low | Low | Low | Low | Low |
| Campbell_1987 | Unclear | Unclear | Low | Low | Unclear | High | High | High |
| Campbell_1993 | Unclear | Unclear | Unclear | Unclear | Unclear | High | Unclear | Moderate |
| Chugani_2016 | Low | Unclear | Unclear | Unclear | Low | Low | High | Moderate |
| Dean_2017 | Low | Low | Low | Low | Low | Low | Low | Low |
| Ekman_1989 | Unclear | Unclear | Unclear | Unclear | High | Unclear | Unclear | Moderate |
| Fahmy_2013 | Low | Unclear | Low | Low | Unclear | Low | High | Moderate |
| Frye_2018 | Low | Low | Low | Low | Low | Low | High | Moderate |
| Gabis_2019 | Unclear | Low | Low | Low | High | High | Unclear | High |
| Geier_2011 | Low | Unclear | Low | Low | High | Low | Low | Moderate |
| Guastella_2015 | Low | Low | Low | Low | Low | Low | Low | Low |
| Handen_2009 | Low | Unclear | Low | Low | Low | High | Unclear | Moderate |
| Handen_2012 | Unclear | Low | Unclear | Unclear | Low | Low | Low | Low |
| Handen_2015 | Low | Low | Low | Low | Low | Low | Low | Low |
| Hardan_2012 | Low | Low | Low | Low | Low | Low | Low | Low |
| Harfterkamp_2013 | Low | Low | Low | Low | Low | Low | High | Moderate |
| Hellings_2005 | Unclear | Low | Unclear | Unclear | Unclear | High | Low | Moderate |
| Hendren_2016 | Low | Low | Unclear | Unclear | Low | Low | Low | Low |
| Herscu_2019 | Low | Low | Low | Low | Low | Low | Low | Low |
| Hollander_2005 | Unclear | Unclear | Unclear | Unclear | Unclear | Unclear | Low | Moderate |
| Hollander_2006 | Unclear | Unclear | Low | Low | Low | Unclear | Low | Low |
| Hollander_2006b | Unclear | Unclear | Low | Low | Unclear | High | Unclear | Moderate |
| Hollander_2010 | Unclear | Unclear | Low | Low | Low | High | Unclear | Moderate |
| Hollander_2012 | Unclear | Unclear | Unclear | Low | Low | Unclear | Low | Moderate |
| Ichikawa_2017 | Unclear | Low | Unclear | Unclear | Low | Low | Low | Low |
| Kent_2013 | Low | Unclear | Low | Low | Low | High | Low | Moderate |
| Kerley_2017 | Unclear | Low | Low | Low | High | Low | Low | Moderate |
| Kern_2001 | Unclear | Low | Low | Low | High | High | High | High |
| King_2001 | Unclear | Unclear | Low | Low | Low | High | Low | Moderate |
| King_2009 | Low | Unclear | Low | Low | Low | Low | Low | Low |
| Klaiman_2013 | Low | Low | Low | Low | Low | Low | High | Moderate |
| Kosaka_2016 | Low | Low | Unclear | Unclear | Low | Low | Low | Low |
| Lemonnier_2012 | Low | Low | Low | Low | High | Low | Low | Moderate |
| Lemonnier_2017 | Low | Low | Low | Low | Low | Low | Low | Low |
| Levine_1997 | Low | Low | Low | Low | Low | Low | Low | Low |
| Liu_2019 | Low | Low | Low | Low | High | Low | Low | Moderate |
| Loebel_2016 | Low | Unclear | Low | Low | Low | Low | Low | Low |
| Mankad_2015 | Low | Low | Low | Low | Low | Low | Low | Low |
| Marcus_2009 | Low | Low | Unclear | Unclear | Low | Low | Low | Low |
| Mazahery_2019 | Low | Low | Low | Low | High | Low | High | High |
| McDougle_1996 | Unclear | Unclear | Low | Low | Low | High | Low | Moderate |
| McDougle_1998 | Low | Unclear | Low | Low | Low | Low | Low | Low |
| Mehrazad_2018 | Low | Unclear | Low | Low | High | Low | Low | Moderate |
| Munesue_2016 | Low | Low | Low | Low | Low | Low | Low | Low |
| Nagaraj_2006 | Low | Low | Low | Low | Low | Low | Low | Low |
| NCT00198107 | Unclear | Unclear | Unclear | Unclear | Low | Low | Unclear | Moderate |
| NCT00498173 | Unclear | Unclear | Low | Low | Unclear | Low | Unclear | Moderate |
| NCT00609531 | Unclear | Low | Unclear | Unclear | Unclear | Unclear | Unclear | Moderate |
| NCT00870727 | Unclear | Unclear | Low | Low | Low | Low | Unclear | Low |
| NCT01302964 | Unclear | Unclear | Low | Low | Low | High | Unclear | Moderate |
| NCT01308749 | Unclear | Unclear | Unclear | Unclear | Low | Low | Unclear | Moderate |
| NCT01624675 | Unclear | Low | Low | Low | Low | High | Low | Moderate |
| NCT01661855 | Unclear | Unclear | Unclear | Unclear | Unclear | Unclear | Unclear | Moderate |
| NCT02385799 | Unclear | Low | Unclear | Unclear | Low | High | Unclear | Moderate |
| Niederhofer_2003 | Unclear | Unclear | Low | Low | Unclear | High | Unclear | Moderate |
| Owen_2009 | Low | Low | Unclear | Unclear | Low | Low | Low | Low |
| Parellada_2017 | Low | Low | Low | Low | High | Low | Low | Moderate |
| Parker_2017 | Low | Low | Low | Low | High | Low | Low | Moderate |
| Pusponegoro_2015 | Unclear | Low | Low | Low | High | Low | Low | Moderate |
| Reddihough_2019 | Low | Low | Low | Low | Low | Low | High | Moderate |
| RUPP_2002 | Low | Low | Low | Low | Low | Low | Low | Low |
| Saad_2015 | Low | Unclear | Unclear | Unclear | High | Low | Low | Moderate |
| Scahill_2015 | Low | Low | Low | Low | Low | Low | Low | Low |
| Shea_2004 | Low | Low | Unclear | Unclear | Low | Low | Low | Low |
| Singh_2014 | Low | Low | Low | Low | Low | Low | Low | Low |
| Stivaros_2018 | Low | Low | Unclear | Unclear | Low | Low | Low | Low |
| VeenstraVanderWeele_2017 | Low | Low | Low | Low | Low | Low | Low | Low |
| Voigt_2014 | Low | Low | Low | Low | High | High | Unclear | High |
| Watanabe_2015 | Low | Low | Low | Low | High | Low | Low | Moderate |
| Wink_2016 | Low | Low | Low | Low | High | Low | Low | Moderate |
| Yamasue_2018 | Low | Low | Low | Low | Low | Low | Low | Low |
| Yatawara_2016 | Low | Low | Low | Low | High | Low | Low | Moderate |
| Yui_2013 | Unclear | Low | Low | Low | Unclear | Low | Low | Low |

### 2.2. Risk of bias summary of included studies


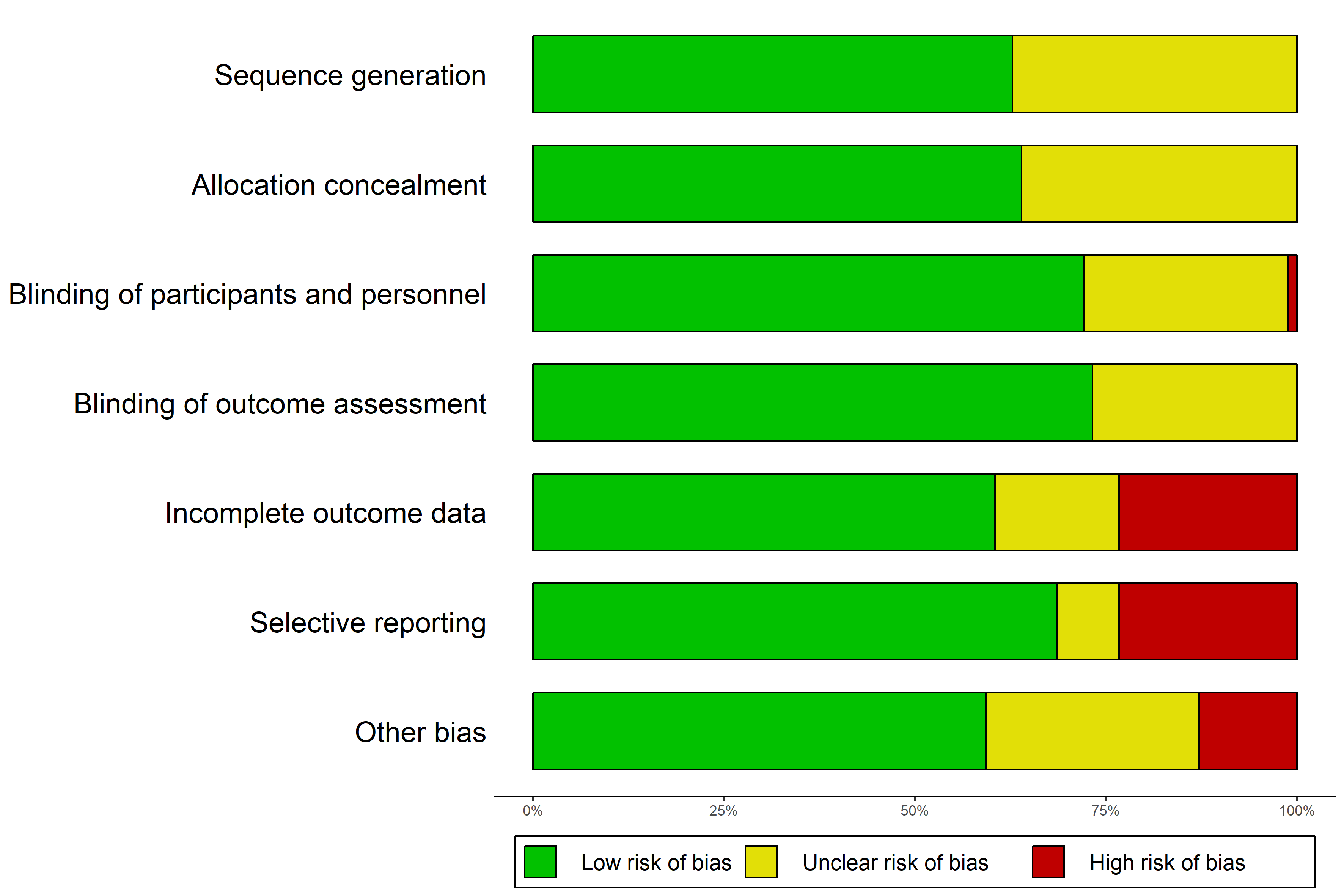


## 3. Eligible scales

### 3.1 General strategy

Data from clinicians’ (observations or interviews), caregivers’, teachers’ rating scales were extracted separately. Usually one scale per type of informant was reported, but

- Regarding caregivers’ and teachers’ ratings, we preferred the frequently used subscales of ABC-Lethargy/Social Withdrawal [1], ABC-Stereotypic behavior [2], and SRS total score [3]. SRS subscales were also eligible when data from other scales were not available [4, 5].
- Regarding clinicians’ ratings, we preferred the frequently used Vineland-Socialization domain (semi-structured interview) [1], CYBOCS-PDD (or C-YBOCS-Compulsion subscale) [2] and CARS [3].

The five original subscales of SRS were based on expert consensus rather appropriate factorial analyses. However, a more recent confirmatory factor analysis suggested that a two-factor structure consisting of social-communication difficulties and repetitive behaviors/restricted interests (RRBI) could be acceptable [6]. Therefore, we decided that SRS subscales would be eligible when data from other scales were not available for social-communication deficits or RRBI. We followed the two-factor structure whenever possible, and when the five original SRS subscales were reported, we used as a) measure of RRBI the subscale ‘Autistic Mannerism’ and as b) measure of social-communication difficulties, we calculated a total score (or average for t-standardized scores) using the four subscales Social Awareness, Social cognition, Social Communication, Social Motivation [5]. To calculate the standard deviation of a total or average score, we assumed a correlation of 0.5 between the subscales [7, 8].

### 3.2. Table of eligible scales

| Social communication and interaction difficulties | Repetitive behaviors and restricted interests | Overall core symptoms |
| --- | --- | --- |
| Preferred:   - ABC-Lethargy/Social Withdrawal - ADOS-Social - AIM-Social reciprocity/ AIM-Peer interaction - ASQ-Social - ATEC-Sociability - BASC-Social skills/BASC-Withdrawal - BOSCC-Social communication - CBCL-Social Problems - GARS-Social - PDD-BI-Social approach behaviors/PDD-BI-Social Pragmatic Problems - VABS-Socialization   Also eligible, when the former not available:   - ADOS-Communication - AIM-Communication - ASQ-Communication - BASC-Functional communication - GARS communication - PDD-BI-Receptive/expressive social communication abilities - VABS-Communication - CCC-2 Social interaction deviance index - SRS-Social communication composite score | - ABC-Stereotypic behavior - ADOS-Repetitive behaviors - AIM-Repetitive behaviors - ASQ-Stereotyped behavior - BOSCC-Repetitive behaviors - CYBOCS-PDD - CYBOCS/YBOCS-Compulsion subscale (total score also eligible) - GARS-Stereotyped behavior - PDD-BI-Sensory perceptual approach behaviors/PDDBI-Stereotyped restricted behavior - RBQ - RBS-R total score - SRS-Autistic Mannerisms (when another scale was not available) | - ADOS-CSS (total score also eligible, if the calibrated severity score not available) - BOSCC total score - AIM-Frequency/AIM-Impact - ASQ total score - AUBC total score - BSE-Autism factor - CARS total score - CBCL-PDD scale - CPRS-Autism factor - CBSQ - GARS total score - PDD-BI-Autism composite score/ PDD-BI-Approach/Withdrawal problems - RF total score - SRS total score (standardized scores preferred to raw) |

Abbreviations:

ABC, Aberrant Behavior Checklist; ADOS, Autism Diagnostic Observation Scale; AIM, Autism Impact Measure; ASQ, Autism Symptoms Questionnaires; ATEC, Autism Treatment Evaluation Checklist; AUBC, Krug’s Autism Behavior Checklist; BASC, Behavior Assessment System for Children; BSE, Behavior Summarized Evaluation; BOSCC, Brief Observation of Social Communication Change; CARS, Childhood Autism Rating Scale; CBCL, Child Behavior Checklist; CBSQ,  Children’s Social Behavior Questionnaire; CCC-2, Children Communication Checklist; CPRS, Children's Psychiatric Rating Scale; (C)YBOCS-PDD, (Children) Yale Obsessive Compulsive Scale-Pervasive Developmental Disorders; GARS, Gilliam Autism Rating Scale; PDD-BI, Pervasive Developmental Disorders Behavioral Inventory; RBQ, Repetitive Behaviour Questionnaire; RBS-R, Repetitive Behavior Scale - Revised; RF, Ritvo-Freeman Real Life Rating Scale; SRS, Social Responsiveness Scale; VABS, Vineland Adaptive Behavior Scale.

## 4. Covariate data

### 4.1. Missing covariate data in included studies


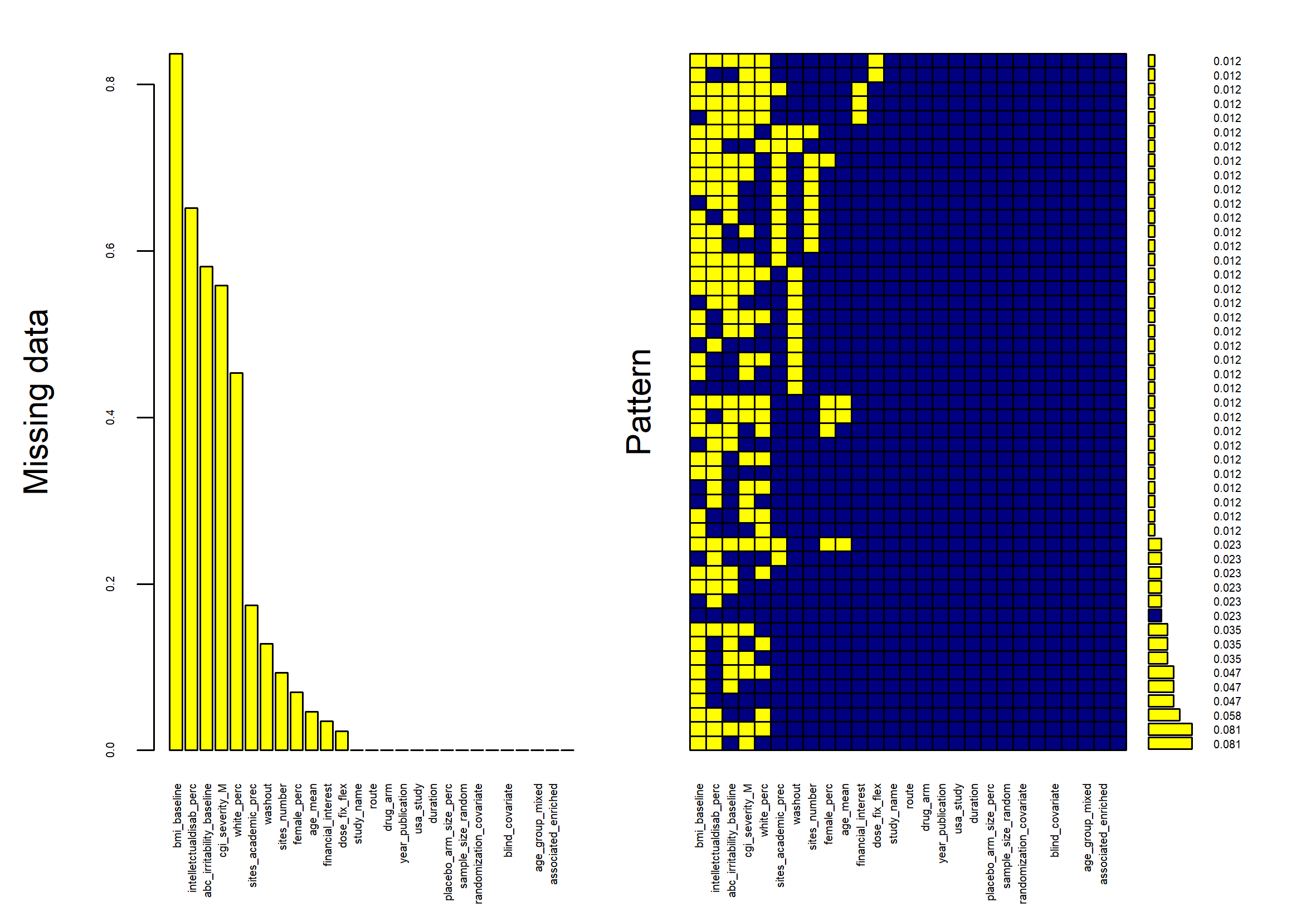


The left graph shows the percentage of studies with missing data for the covariates. Data for some covariates were very limited, especially for participant-related factors e.g. baseline BMI (missing in more than 80% of the studies), percentage of participants with intellectual disability, baseline ABC-Irritability of CGI-Severity. The right graph shows the patterns of missing covariate data (yellow for missing data, blue fore reported). Only a small portion of studies had data for all covariates (2.3%).

### Correlation matrix with the bivariate Spearman’ correlations between covariates


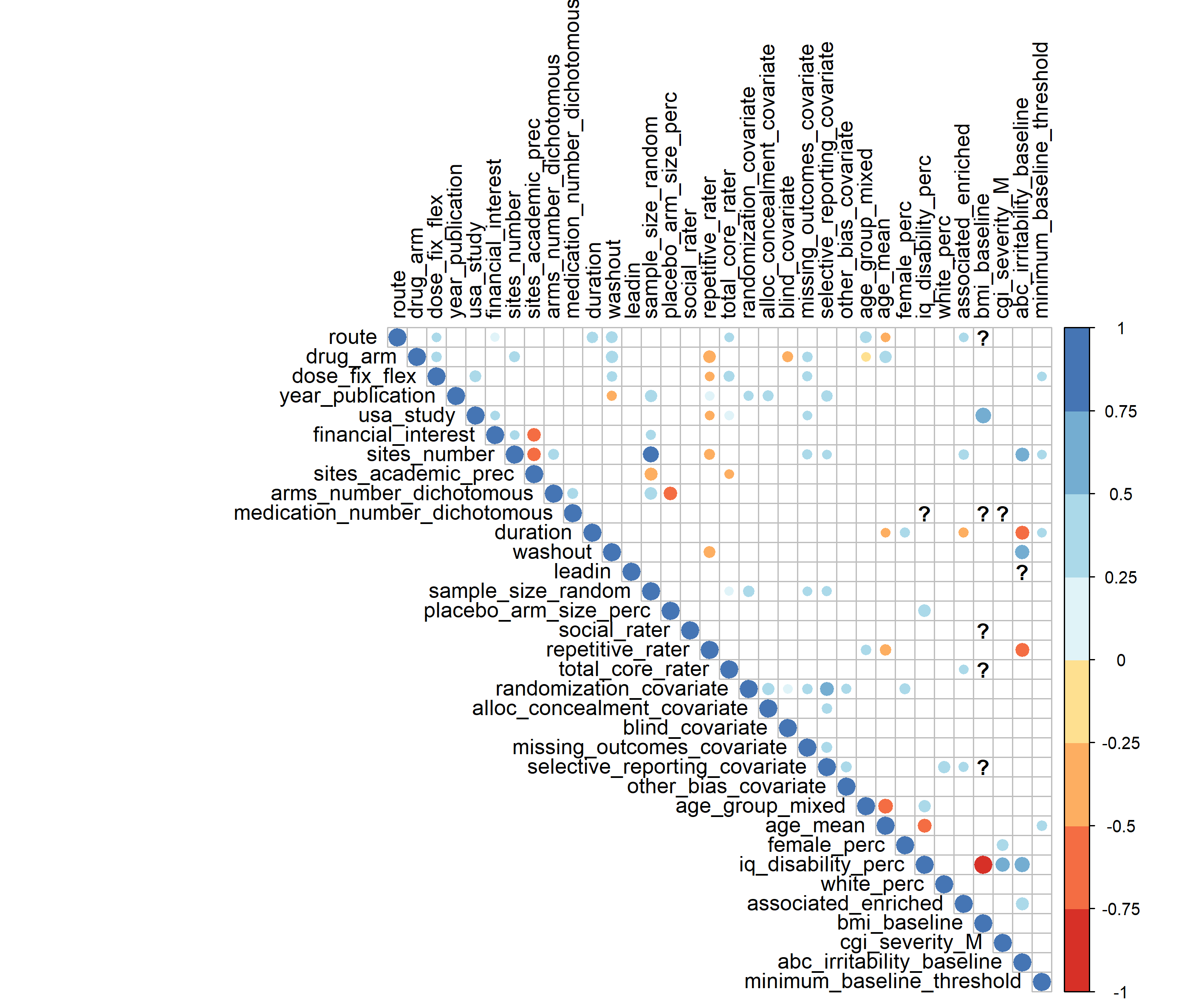


Post-hoc, we conducted bivariate Spearman’ ρ correlation between covariates. Only significant correlations (p<0.05) are presented. Significant correlations with a |ρ|>0.5 were present between sample size and number of sites (ρ =0.77, p<0.001), baseline BMI and percentage of participants with intellectual disability (ρ=-1, p<0.001), country of origin and baseline BMI (ρ=0.7, p=0.01), mean age and age group (ρ=-0.62, p<0.001), baseline ABC-Irritability and the following covariates percentage of participants with intellectual disability (ρ=0.67, p=0.03), washout period (ρ=0.59, p<0.001), type of rater of repetitive behaviors (ρ=-0.57, p<0.001), number of sites (ρ=0.55, p<0.001), as well as duration of treatment (ρ=-0.54, p<0.001) between CGI-S and percentage of participants with intellectual disability (ρ=0.58, p=0.04), between sequence generation and selective reporting (ρ=0.57, p<0.001), number of arms and percentage of participants on placebo (ρ=-0.54, p<0.001), mean age and percentage of participants with intellectual disability (ρ=-0.54, p<0.001), between percentage of academic sites and industry-funding (ρ=-0.52, p<0.001) and number of sites (ρ=-0.51, p<0.001).

It should be noted that some of these covariates had a large proportion of missing data, i.e. baseline BMI, percentage of participants with intellectual disability, baseline ABC-Irritability, baseline CGI-Severity. ? = correlations could not be calculated due to missing data.

## 5. References

1. Anagnostou E, Jones N, Huerta M, Halladay AK, Wang P, Scahill L, Horrigan JP, Kasari C, Lord C, Choi D *et al*: **Measuring social communication behaviors as a treatment endpoint in individuals with autism spectrum disorder**. *Autism* 2014, **19**(5):622-636.

2. Scahill L, Aman MG, Lecavalier L, Halladay AK, Bishop SL, Bodfish JW, Grondhuis S, Jones N, Horrigan JP, Cook EH *et al*: **Measuring repetitive behaviors as a treatment endpoint in youth with autism spectrum disorder**. *Autism* 2013, **19**(1):38-52.

3. European Medicines Agency: **Guideline on the clinical development of medicinal products for the treatment of Autism Spectrum Disorder (ASD)**. In*.* Edited by (CHMP) CfMPfHU; 2017.

4. Constantino JN, Gruber CP: **Social Responsiveness Scale: SRS-2 Software Kit**: Western Psychological Services; 2012.

5. Cheon KA, Park JI, Koh YJ, Song J, Hong HJ, Kim YK, Lim EC, Kwon H, Ha M, Lim MH *et al*: **The social responsiveness scale in relation to DSM IV and DSM5 ASD in Korean children**. *Autism research : official journal of the International Society for Autism Research* 2016, **9**(9):970-980.

6. Frazier TW, Ratliff KR, Gruber C, Zhang Y, Law PA, Constantino JN: **Confirmatory factor analytic structure and measurement invariance of quantitative autistic traits measured by the Social Responsiveness Scale-2**. *Autism* 2013, **18**(1):31-44.

7. Borenstein M, Hedges LV, Higgins JPT, Rothstein HR: **Introduction to meta-analysis**: John Wiley & Sons; 2011.

8. Balk EM, Earley A, Patel K, Trikalinos TA, Dahabreh IJ: **Empirical assessment of within-arm correlation imputation in trials of continuous outcomes**. 2012.

# eAppendix-6 Results

1. Sensitivity analyses 133

1.1. Social communication and interaction difficulties 133

1.2. Repetitive behaviors and restricted interests 133

1.3. Overall core symptoms 134

1.4. Responder rates 134

2. Publication bias and small study effects 135

2.1. Egger’s test and trim-and-fill analysis 135

a. Funnel plots 135

2.2.1. Social communication and interaction difficulties 135

2.2.2. Repetitive behavior and restricted interests 136

2.2.3. Overall core symptoms 137

3. Meta-regression analyses 138

3.1. Univariate meta-regression analyses 138

3.1.1. Meta-analytic scatter plots 138

3.2. Multivariate meta-regression models 138

3.2.1. Social communication and interaction difficulties 138

3.2.1.a Initial factors: publication year, type of rater, ABC-Irritability, other bias 138

3.2.1.b. Initial factors: publication year, type of rater, other bias 138

3.2.2. Repetitive behaviors and restricted interests 138

3.2.2.a. Initial factors: dose administration schedule, sample size, minimum threshold of core symptoms at inclusion 138

3.2.3. Overall core symptoms 139

3.2.3.a. Initial factors: number of sites, allocation concealment 139

4. Placebo response by scales filled by clinicians, caregivers and teachers 140

4.1. Social communication and interaction difficulties 140

4.1.1. Table of separate analysis of scales filled by different raters in social communication difficulties 140

4.1.2. Forest plot of social communication and interaction difficulties rated by caregivers 140

4.1.3. Forest plot for social communication and interaction difficulties rated by clinicians 141

4.1.4. Forest plot for social communication and interaction difficulties rated by teachers 142

4.2. Repetitive behaviors and restricted interests 142

4.2.1. Table of separate analysis of scales filled by different raters in repetitive behaviors 142

4.2.2. Forest plot of repetitive behaviors and restricted interests rated by caregivers 143

4.2.3. Forest plot of repetitive behaviors and restricted interests rated by clinicians 144

4.2.4. Forest plot of repetitive behaviors and restricted interests rated by teachers 145

4.3. Overall core symptoms 145

4.3.1. Table of separate analysis of scales filled by different raters in overall core symptoms 145

4.3.2. Forest plot of overall core symptoms rated by caregivers 146

4.3.3. Forest plot of overall core symptoms rated by clinicians 147

5. Correlation between standardized mean changes of placebo and experimental intervention 148

5.1. Social-communication difficulties 148

5.2. Repetitive behaviors 149

5.3. Overall core symptoms 150

6. References 151

## 1. Sensitivity analyses

There was no study in the analysis with implied randomization (as defined by our protocol, eAppendix-2, when randomization was not indicated in a double-blind study). Therefore, sensitivity analyses with exclusion of studies with implied randomization were not performed.

### 1.1. Social communication and interaction difficulties

| **Analysis** | **k** | **SMC [95% CI]** | **χ^2^ , p-value** | **I^2^ (%)** |
| --- | --- | --- | --- | --- |
| Primary (pre-post correlation 0.5) | 52 | -0.32 [-0.39, -0.25] | 74.87, 0.0164 | 31.88 |
| Fixed effects | 52 | -0.31 [-0.37, -0.26] | - | - |
| Using a pre-post correlation 0.25 | 52 | -0.32 [-0.38, -0.25] | 52.82, 0.4035 | 3.45 |
| Using a pre-post correlation 0.75 | 52 | -0.31 [-0.38, -0.25] | 131.71, <0.001 | 61.28 |
| Exclusion of implied randomization | - | - | - | - |
| Exclusion of the one single blind study [1] | - | - | - | - |
| Exclusion of the single study with genetic syndrome at baseline [2] | 51 | -0.32 [-0.39, -0.25] | 74.73, 0.0133 | 33.09 |
| Exclusion of studies used only diagnostic tools | 48 | -0.32 [-0.40, -0.25] | 72.75, 0.0094 | 35.40 |
| Exclusion of shorter than 4 weeks | - | - | - | - |
| Exclusion of studies presenting only completers data | 37 | -0.35 [-0.42, -0.27] | 51.46, 0.0457 | 30.04 |
| Exclusion of studies with an overall moderate or high risk of bias | 27 | -0.40 [-0.49, -0.31] | 43.48, 0.0172 | 40.20 |
| Exclusion of studies with estimated SD (imputed, from median/range, pooling subscales) | 48 | -0.34 [-0.41, -0.27] | 65.97, 0.0353 | 28.75 |
| Exclusion of studies that did not report baseline SD, and change or follow-up SDs were used for the calculation of SMC | 46 | -0.33 [-0.40, -0.26] | 69.14, 0.0119 | 34.91 |

### 1.2. Repetitive behaviors and restricted interests

| **Analysis** | **k** | **SMC [95% CI]** | **χ^2^ , p-value** | **I^2^ (%)** |
| --- | --- | --- | --- | --- |
| Primary (pre-post correlation 0.5) | 52 | -0.23 [-0.32, -0.15] | 113.32, <0.001 | 55.00 |
| Fixed effects | 52 | -0.27 [-0.32, -0.21] | - | - |
| Using a pre-post correlation 0.25 | 52 | -0.25 [-0.33, -0.16] | 81.03, 0.0047 | 37.06 |
| Using a pre-post correlation 0.75 | 52 | -0.22 [-0.30, -0.14] | 191.37, <0.001 | 73.35 |
| Exclusion of implied randomization | - | - | - | - |
| Exclusion of the one single blind study [1] | - | - | - | - |
| Exclusion of the single study with genetic syndrome at baseline [2] | 51 | -0.23 [-0.32, -0.15] | 113.32, <0.001 | 55.88 |
| Exclusion of studies used only diagnostic tools | 49 | -0.24 [-0.33, -0.15] | 112.18, <0.001 | 57.21 |
| Exclusion of shorter than 4 weeks | - | - | - | - |
| Exclusion of studies presenting only completers data | 39 | -0.23 [-0.33, -0.13] | 108.14, <0.001 | 64.86 |
| Exclusion of studies with an overall moderate or high risk of bias | 29 | -0.27 [-0.39, -0.15] | 78.66, <0.001 | 64.41 |
| Exclusion of studies with estimated SD (imputed, from median/range, pooling subscales) | 51 | -0.22 [-0.31, -0.14] | 107.76, <0.001 | 53.60 |
| Exclusion of studies that did not report baseline SD, and change or follow-up SDs were used for the calculation of SMC | 48 | -0.23 [-0.32, 0.15] | 104.85, <0.001 | 55.18 |

### 1.3. Overall core symptoms

| **Analysis** | **k** | **SMC [95% CI]** | **χ^2^ , p-value** | **I^2^ (%)** |
| --- | --- | --- | --- | --- |
| Primary (pre-post correlation 0.5) | 45 | -0.36 [-0.46, -0.26] | 98.94, <0.001 | 55.53 |
| Fixed effects | 45 | -0.35 [-0.41, -0.28] | - | - |
| Using a pre-post correlation 0.25 | 45 | -0.36 [-0.47, -0.26] | 73.24, <0.001 | 39.92 |
| Using a pre-post correlation 0.75 | 45 | -0.35 [-0.45, -0.26] | 157.11, <0.001 | 71.99 |
| Exclusion of implied randomization | - | - | - | - |
| Exclusion of the one single blind study [1] | 44 | -0.35 [-0.45, -0.25] | 93.88, <0.001 | 54.20 |
| Exclusion of the single study with genetic syndrome at baseline [2] | - | - | - | - |
| Exclusion of studies used only diagnostic tools | 41 | -0.37 [-0.48, -0.26] | 95.47, <0.001 | 58.10 |
| Exclusion of shorter than 4 weeks | 43 | -0.33 [-0.42, -0.23] | 77.62, <0.001 | 45.89 |
| Exclusion of studies presenting only completers data | 28 | -0.38 [-0.48, -0.28] | 45.62, 0.0140 | 40.82 |
| Exclusion of studies with an overall moderate or high risk of bias | 20 | -0.46 [-0.59, -0.33] | 32.73, 0.0258 | 41.94 |
| Exclusion of studies with estimated SD (imputed, from median/range, pooling subscales) | 42 | -0.38 [-0.48, -0.27] | 97.47, <0.001 | 57.94 |
| Exclusion of studies that did not report baseline SD, and change or follow-up SDs were used for the calculation of SMC | 41 | -0.36 [-0.47, -0.25] | 94.99, <0.001 | 57.89 |

### 1.4. Responder rates

| **Analysis** | **k** | **Responder rate [95% CI]** | **χ^2^ , p-value** | **I^2^ (%)** |
| --- | --- | --- | --- | --- |
| Primary | 57 | 18.90 [15.93-22.27] | 119.7, <0.001 | 53.2 |
| Exclusion of studies with imputed responder rates | 46 | 19.13 [15.73-23.06] | 106.6, <0.001 | 57.8 |

## 2. Publication bias and small study effects

### 2.1. Egger’s test and trim-and-fill analysis

| **Analysis** | **z-test** | **p-value** | **Trim-and-fill SMC** |
| --- | --- | --- | --- |
| Social communication and interaction difficulties | -0.3816 | 0.7028 | No missing study |
| Repetitive behavior and restricted interests | 1.7057 | 0.0881 | Missing 14 studies, -0.33 [-0.41, -0.25] |
| Overall core symptoms | -1.8194 | 0.0689 | No missing study |

### Funnel plots

#### 2.2.1. Social communication and interaction difficulties


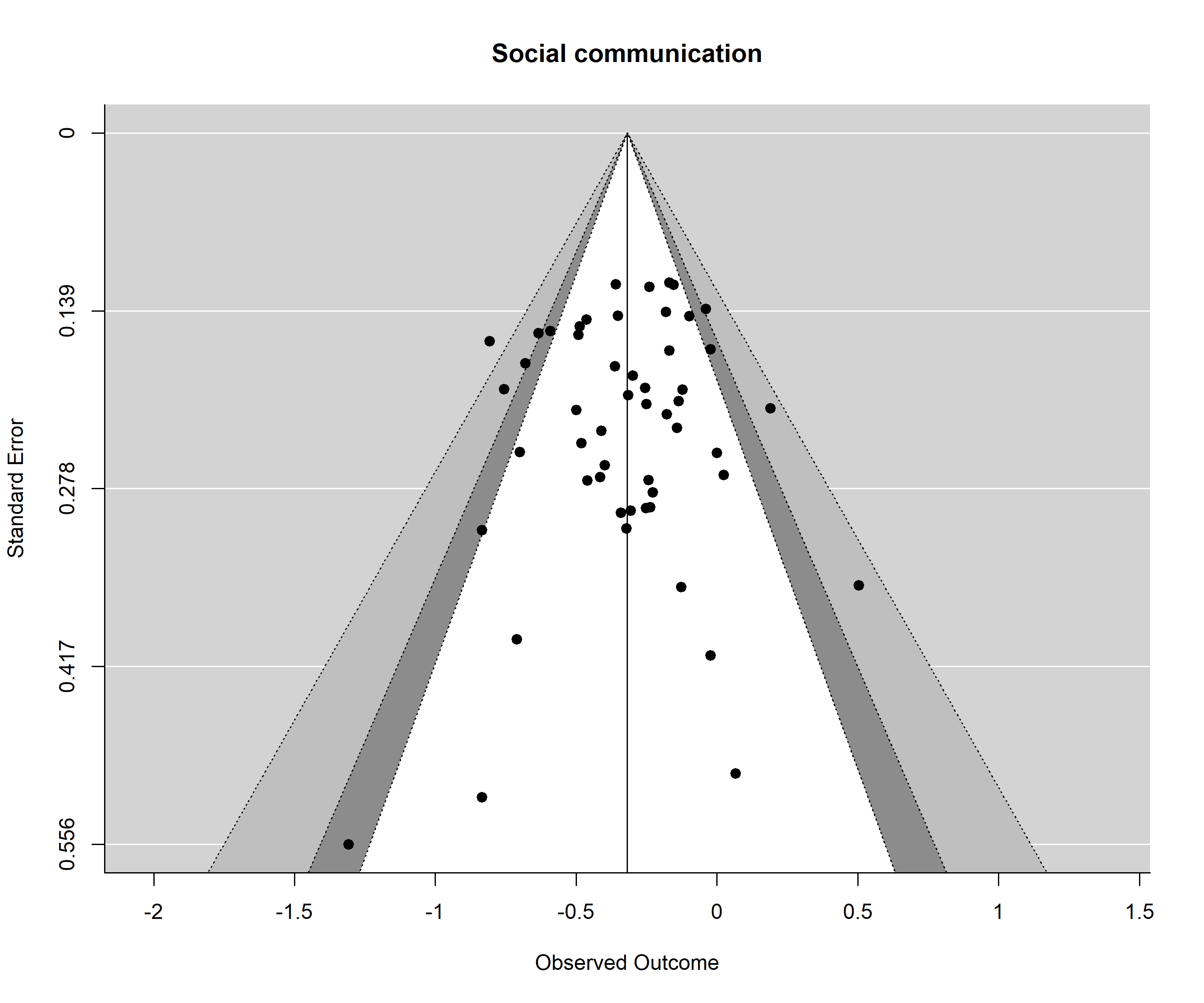
In the trim-and-fill analysis of social communication difficulties, no studies were estimated to be missing.

#### 2.2.2. Repetitive behavior and restricted interests


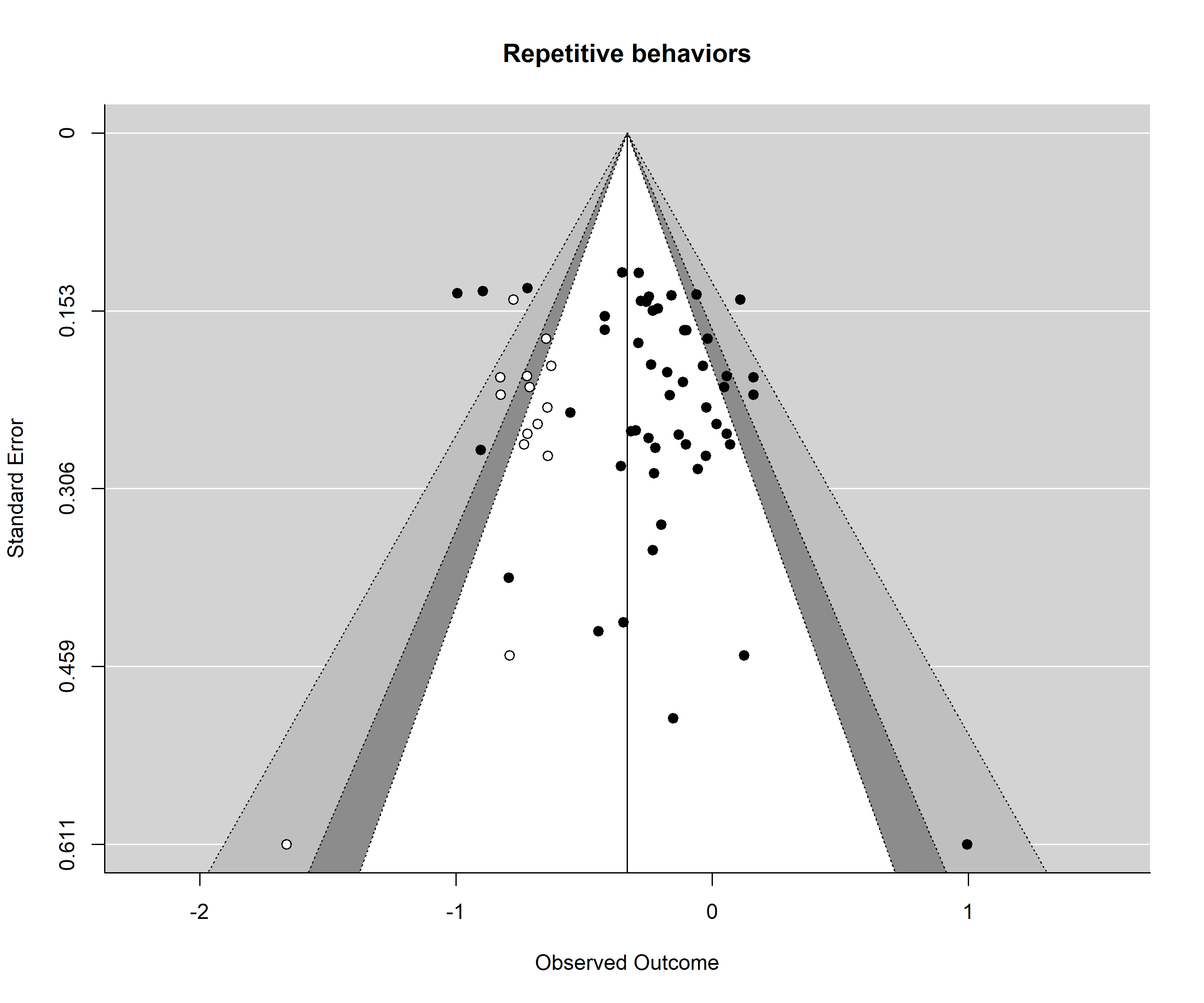
In the trim-and-fill analysis of repetitive behaviors, 14 studies were estimated to be missing from the left side.

#### 2.2.3. Overall core symptoms


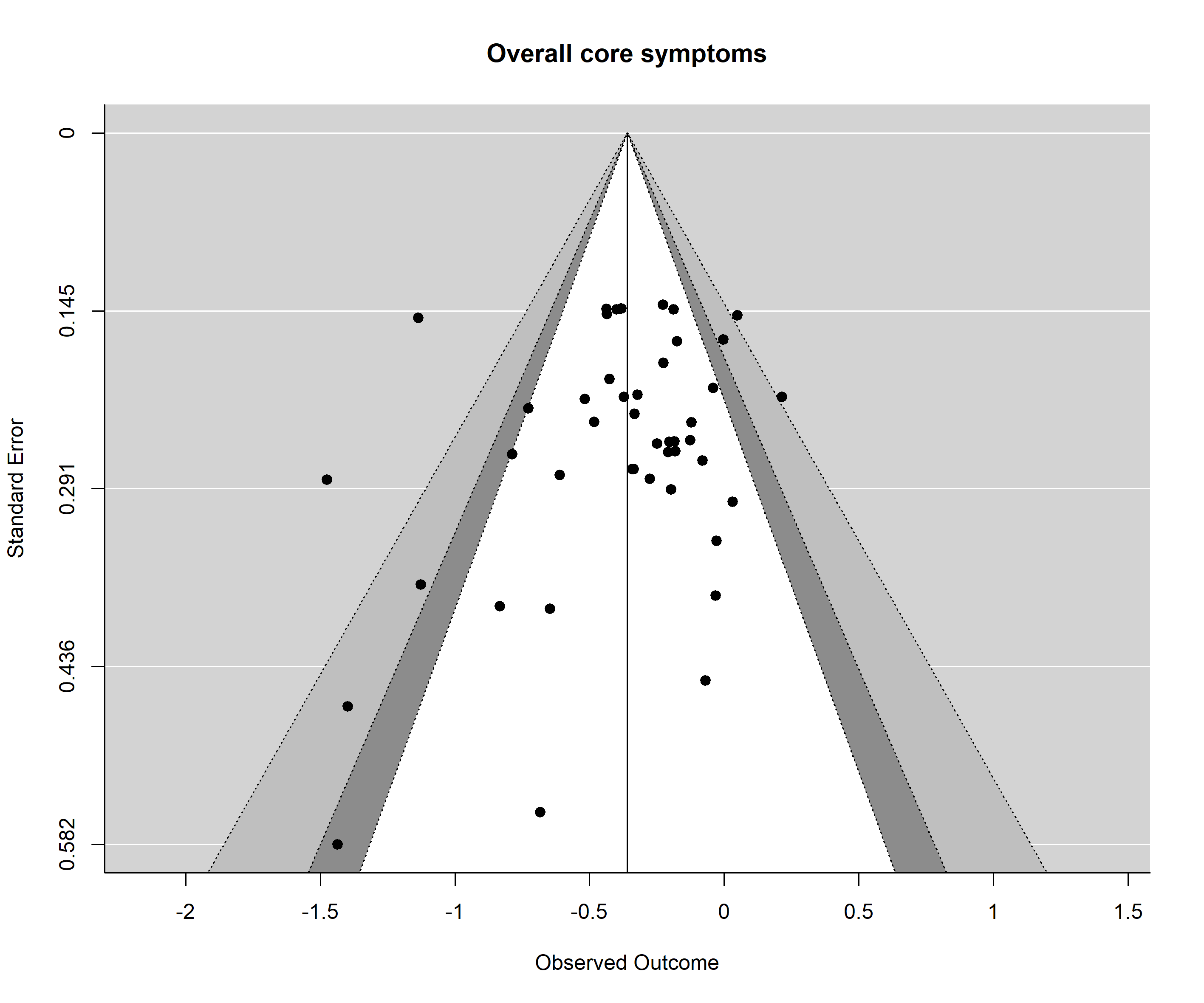


In the trim-and-fill analysis of overall core symptoms, no studies were estimated to be missing.

## 3. Meta-regression analyses

### 3.1. Univariate meta-regression analyses

#### 3.1.1. Meta-analytic scatter plots

Meta-analytic scatter plots are presented in [Figure-S](file:///\\nas.ads.mwn.de\ge73tul\AIMS-2\analysis\placebo_response\manuscript_placebo_response\manuscript_v2\Figure_S_27092019.pdf). Each point represents the point estimate of standardized mean change (SMC) of placebo response of the study (negative for improvement), and the size of the point is inverse proportional of the variance of SMC. For continuous covariates, the meta-regression line and its confidence interval is presented (negative slope for increased placebo response). For categorical covariates, simple boxplots are presented instead. The coefficient β (negative for increased placebo response) and its 95% confidence interval and p-value are presented.

### 3.2. Multivariate meta-regression models

As in our previous analyses in schizophrenia [3], multivariate meta-regression models were conducted using factors that were significant in the univariate analysis and then a formal backward stepwise algorithm with a removal criterion of p=0.15. Studies without missing values in any of the initial factors were included in the multivariate models. For the intercept, continuous covariates were centered around the median of the total sample and a reference was used for dichotomous covariates.

#### 3.2.1. Social communication and interaction difficulties

##### 3.2.1.a Initial factors: publication year, type of rater, ABC-Irritability, other bias

| **Covariates** | **k** | **β [95% CI]** | **p** | **R^2^ (%)** |
| --- | --- | --- | --- | --- |
| Intercept | 31 | -0.0652[-0.2869, 0.1565] | 0.5644 | 100 |
| Rater (ref. clinician) |  | -0.1711[-0.3688, 0.0267] | 0.0899 |  |
| Baseline ABC-Irritability (median 17.185) |  | -0.0103[-0.0222, 0.0016] | 0.0905 |  |
| Other bias (ref. unclear/high) |  | -0.2069[-0.3677, -0.0461] | 0.0117 |  |

##### 3.2.1.b. Initial factors: publication year, type of rater, other bias

| **Covariates** | **k** | **β [95% CI]** | **p** | **R^2^ (%)** |
| --- | --- | --- | --- | --- |
| Intercept | 51 | -0.1051[-0.2562, 0.0460] | 0.1727 | 69.65 |
| Rater (ref. clinician) |  | -0.1368[-0.2741, 0.0004] | 0.0506 |  |
| Publication year (median 2014.5) |  | 0.0141[0.0013, 0.0269] | 0.0312 |  |
| Other bias (ref. unclear/high) |  | -0.1545[-0.2821, -0.0270] | 0.0176 |  |

Because ABC-Irritability was available in 31 studies and the multivariate model was conducted in this subset of studies, we also conducted an analysis without baseline ABC-Irritability.

#### 3.2.2. Repetitive behaviors and restricted interests

##### 3.2.2.a. Initial factors: dose administration schedule, sample size, minimum threshold of core symptoms at inclusion

| **Covariates** | **k** | **β [95% CI]** | **p** | **R^2^ (%)** |
| --- | --- | --- | --- | --- |
| Intercept | 51 | -0.0572 [-0.1651, 0.0506] | 0.2983 | 68.64 |
| Dose administration (ref. fixed) |  | -0.1680[-0.3095, -0.0264] | 0.0201 |  |
| Sample size (median 45) |  | -0.0015[-0.0029, -0.0001] | 0.0387 |  |
| Minimum threshold of core symptoms at baseline (ref. not used) |  | -0.1951[-0.3933, 0.0031] | 0.0537 |  |

#### 3.2.3. Overall core symptoms

##### 3.2.3.a. Initial factors: number of sites, allocation concealment

| **Covariates** | **k** | **β [95% CI]** | **p** | **R^2^ (%)** |
| --- | --- | --- | --- | --- |
| Intercept | 41 | -0.1249[-0.3272, 0.0773] | 0.2261 | 44.09 |
| Allocation concealment (ref. unclear) |  | -0.2292[-0.4554, -0.0030] | 0.0470 |  |
| Number of sites (median one site) |  | -0.0242 [-0.0431, -0.0053] | 0.0120 |  |

*Number of sites was found not significant in the univariate analysis by excluding one outlier study.

## 4. Placebo response by scales filled by clinicians, caregivers and teachers

### 4.1. Social communication and interaction difficulties

#### 4.1.1. Table of separate analysis of scales filled by different raters in social communication difficulties

| **Analysis** | **k** | **n** | **SMC [95% CI]** | **χ^2^ , p-value** | **I^2^ (%)** |
| --- | --- | --- | --- | --- | --- |
| Primary (Clinician were preffered to caregiver) | 52 | 1497 | -0.32 [-0.39, -0.25] | 74.87, 0.0164 | 31.88 |
| Caregiver | 46 | 1386 | -0.39 [-0.46, -0.31] | 72.33, 0.006 | 37.78 |
| Clinician | 12 | 371 | -0.20 [-0.30, -0.09] | 7.34, 0.7709 | 0 |
| Teacher | 3 | 72 | 0.03 [-0.57, 0.63] | 7.37, 0.0250 | 72.88 |

*In teacher ratings, there was one outlier study with high risk of selective reporting (see below the forest plot of teacher ratings, eAppendix-4.1.4). One study, Niederhofer 2003, reported an aggregated score of ABC-L/SW as rated by caregivers and teachers. Therefore, this study was not included in the separate analyses or meta-regressions.

#### 4.1.2. Forest plot of social communication and interaction difficulties rated by caregivers


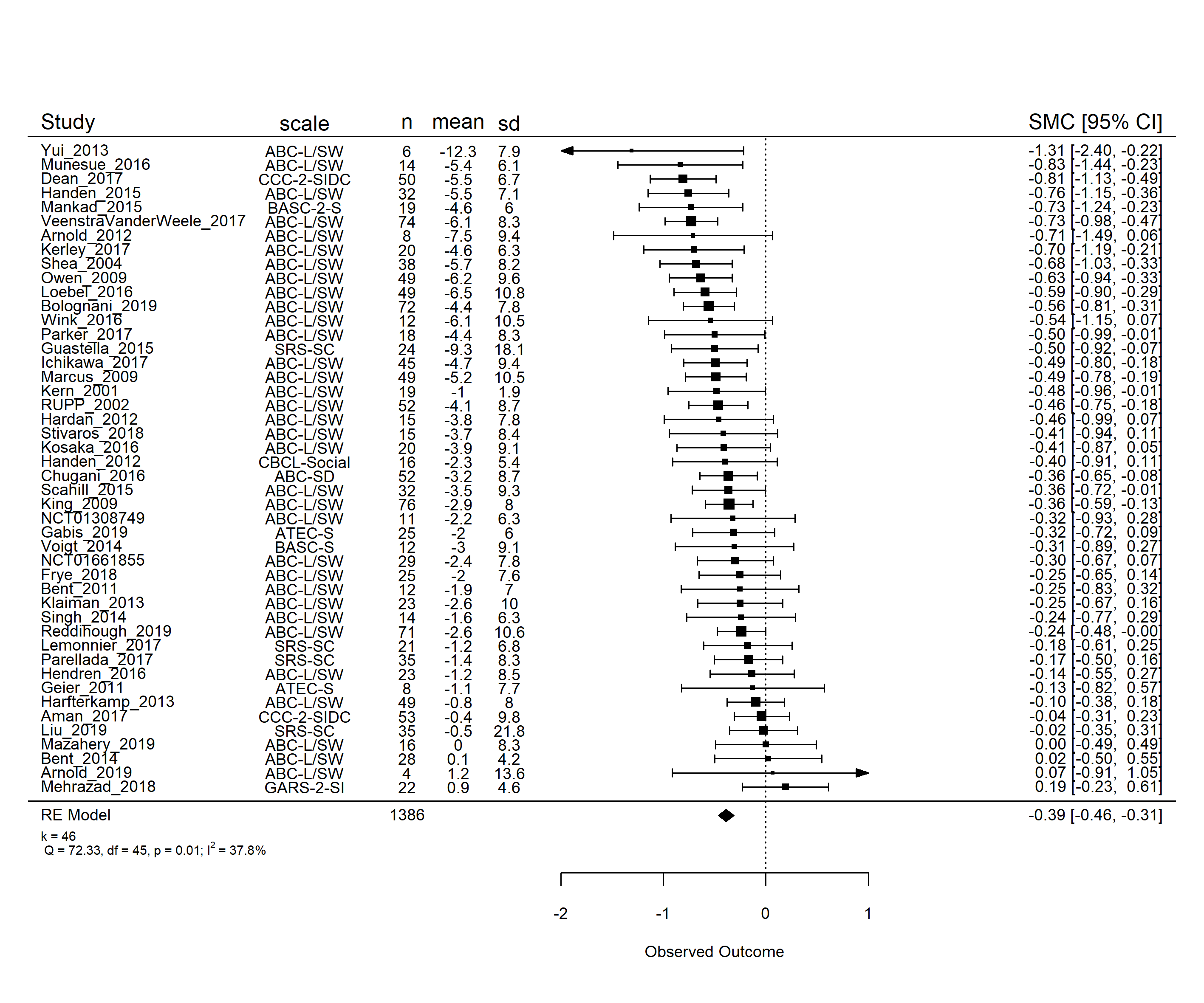


*SEs might have been reported in Chugani_2016 as SDs. Therefore, we calculated SDs from the reported values (no reply from corresponding author).

#### 4.1.3. Forest plot for social communication and interaction difficulties rated by clinicians


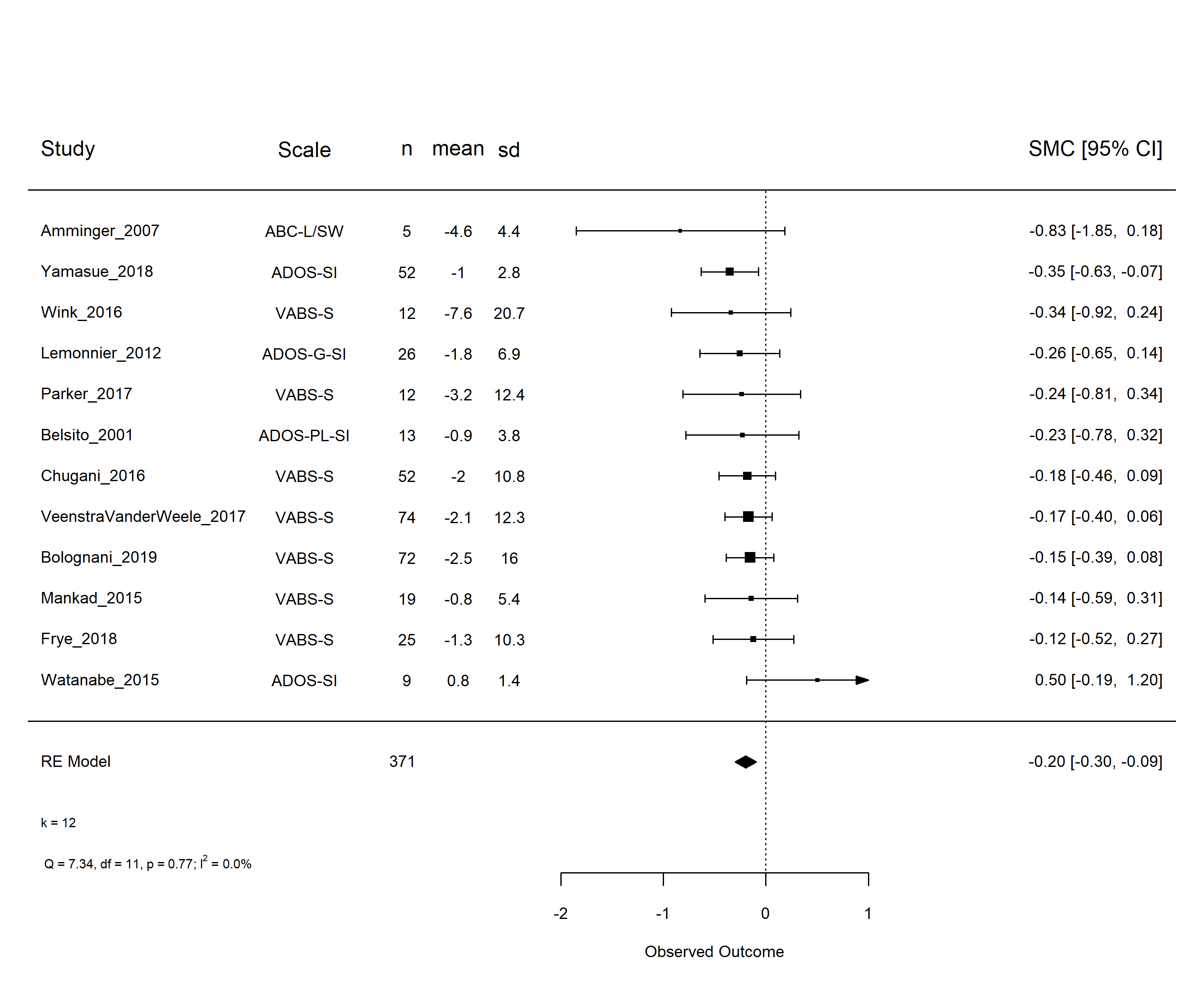
*It should be noted that in Amminger_2007, ABC-L/SW was rated by clinicians of the day care center. SEs might have been reported in Chugani_2016 as SDs. Therefore, we calculated SDs from the reported values (no reply from corresponding author).

#### 4.1.4. Forest plot for social communication and interaction difficulties rated by teachers


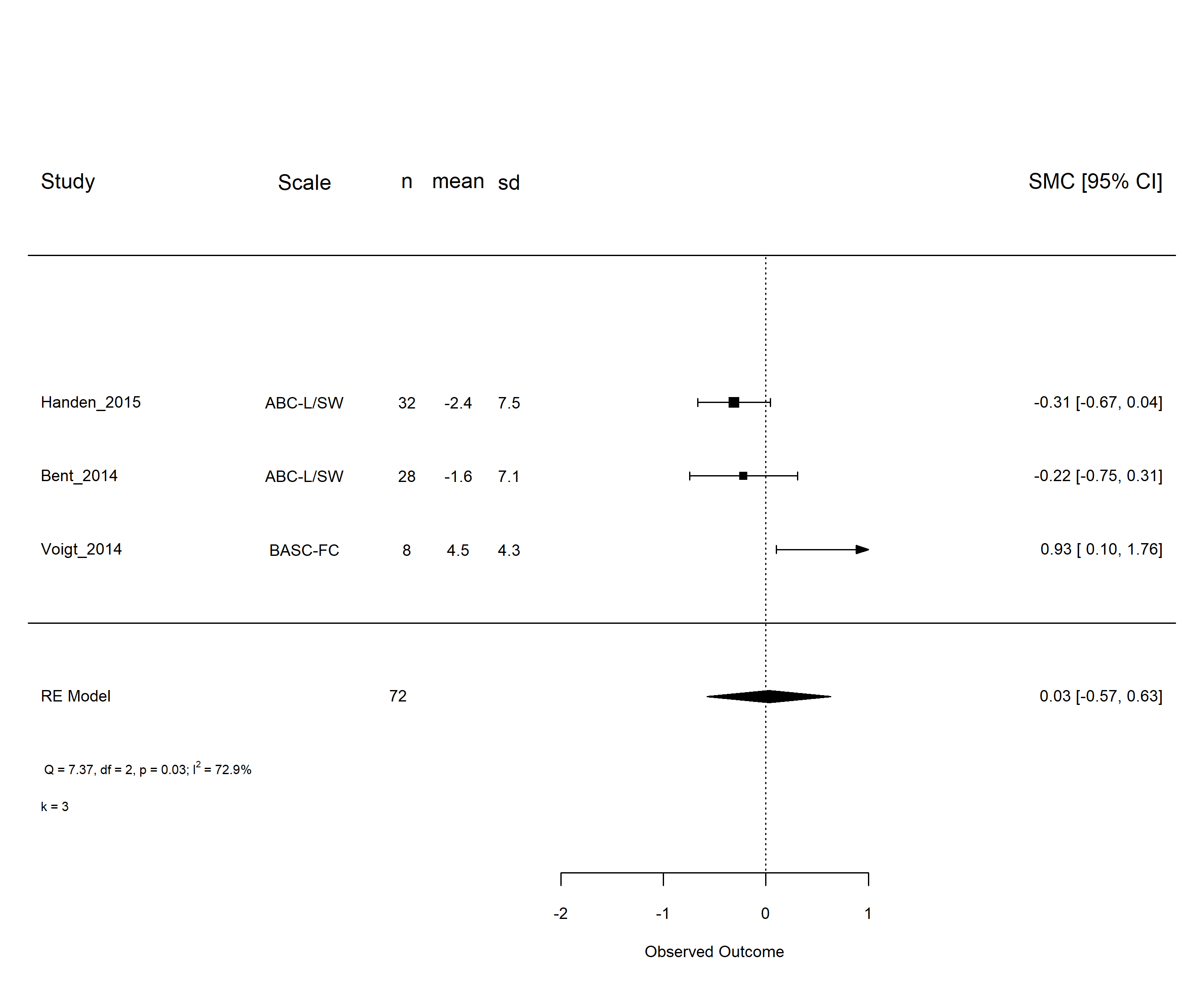
*The study of Voigt 2014 was an outlier and had a high risk of selective reporting bias. The teacher rated BASC-Functional Communication was reported (rather Social Skills or Withdrawal subscales, which were preferred according to our methods, eAppendix-5.3.2). These subscales was selectively reported in the trial because there was a significant difference between placebo and omega-3 fatty acids in this subscale.

### 4.2. Repetitive behaviors and restricted interests

#### 4.2.1. Table of separate analysis of scales filled by different raters in repetitive behaviors

| **Analysis** | **k** | **n** | **SMC [95% CI]** | **χ^2^ , p-value** | **I^2^ (%)** |
| --- | --- | --- | --- | --- | --- |
| Primary (Clinician were preferred to Caregiver) | 52 | 1492 | -0.23 [-0.32, -0.15] | 113.32, <0.001 | 55.00 |
| Caregiver | 41 | 1247 | -0.23 [-0.29, -0.18] | 42.11, 0.42 | 5.01 |
| Clinician | 22 | 732 | -0.30 [-0.45, -0.15] | 72.69, <0.001 | 71.11 |
| Teacher | 2 | 60 | -0.17 [-0.46, 0.12] | 0.0003, 0.99 | 0 |

*In Anagonstou_2012, it was unclear if RBS-R might have been rated by self-reports and not by caregivers. Therefore, it was not included in the meta-regression of type of rater or in the separate analysis.

#### 4.2.2. Forest plot of repetitive behaviors and restricted interests rated by caregivers


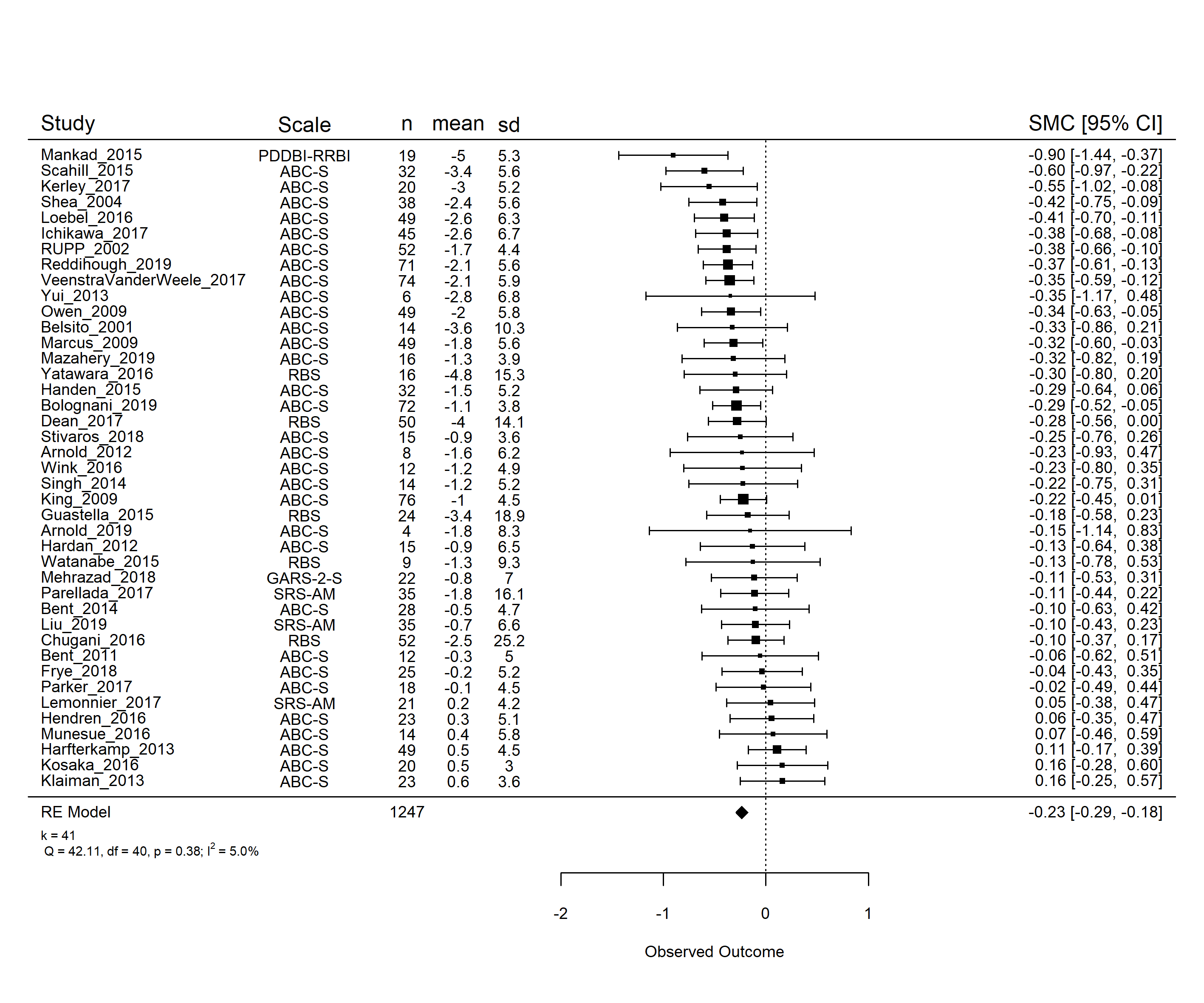


*SEs might have been reported in Chugani_2016 as SDs. Therefore, we calculated SDs from the reported values.

#### 4.2.3. Forest plot of repetitive behaviors and restricted interests rated by clinicians


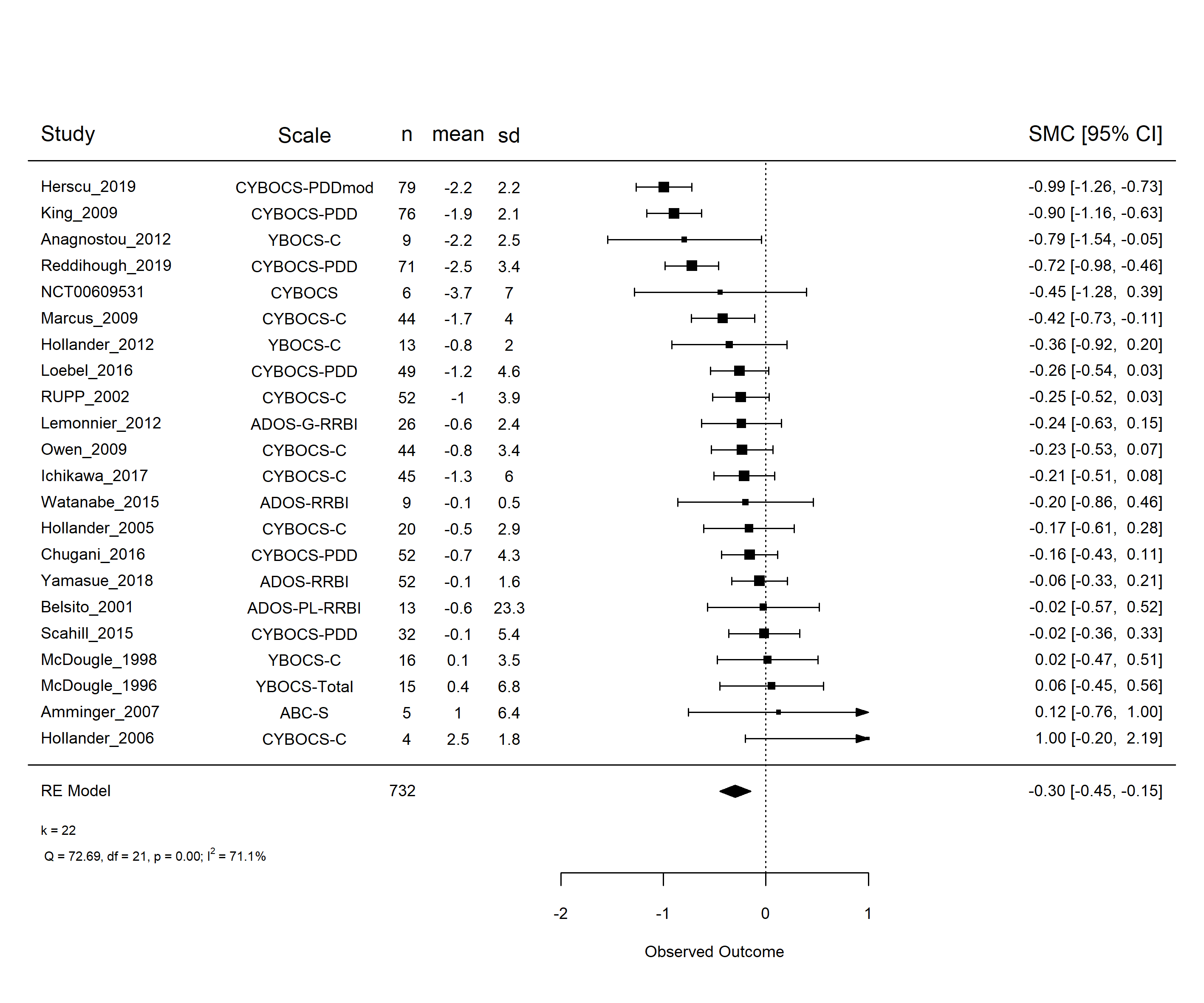
*It should be noted that in Amminger_2007, ABC-S was rated by clinicians of the day care center. SEs might have been reported in Chugani_2016 as SDs. Therefore, we calculated SDs from the reported values.

#### 4.2.4. Forest plot of repetitive behaviors and restricted interests rated by teachers


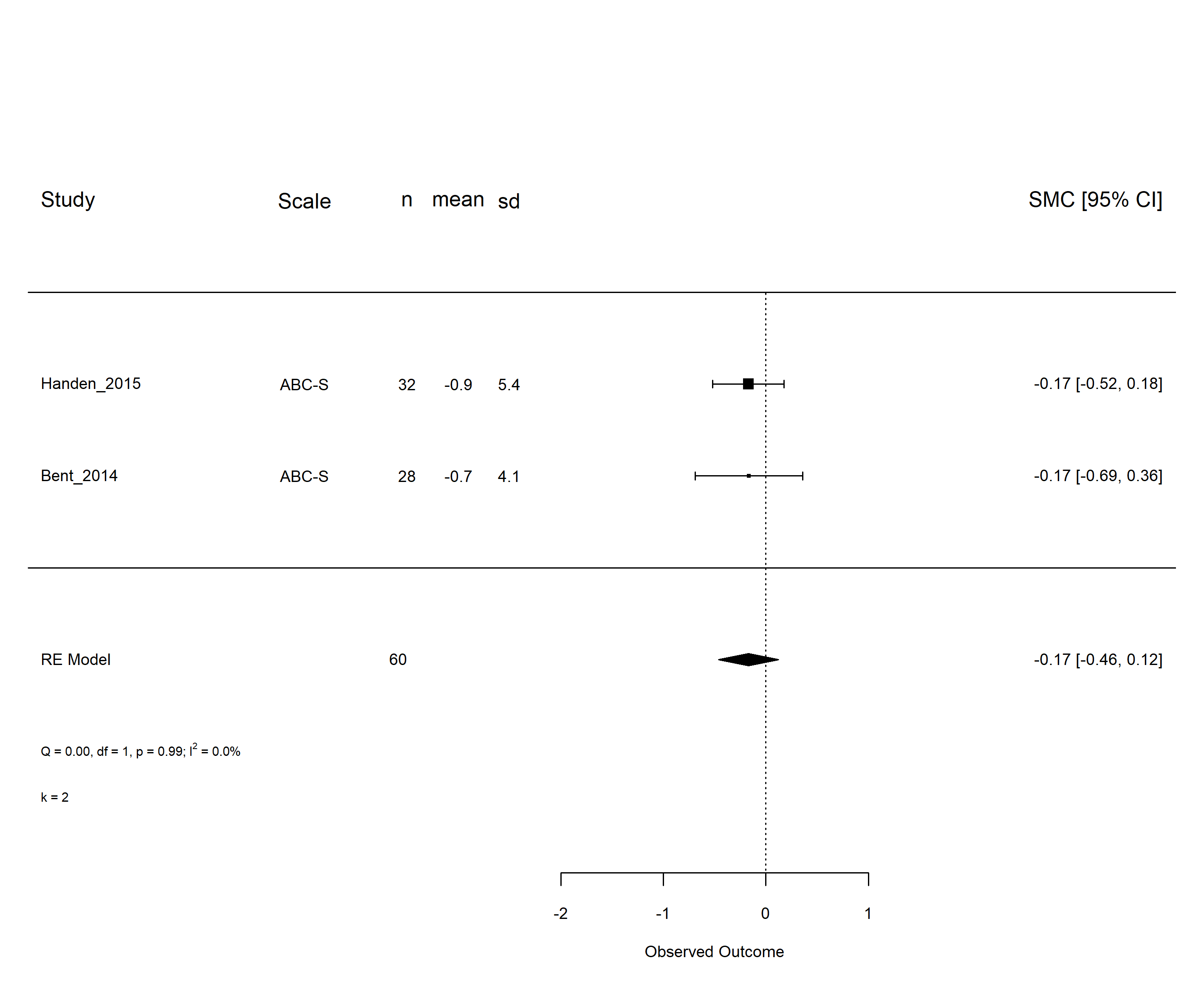


### 4.3. Overall core symptoms

#### 4.3.1. Table of separate analysis of scales filled by different raters in overall core symptoms

| **Analysis** | **k** | **n** | **SMC [95% CI]** | **χ^2^ , p-value** | **I^2^ (%)** |
| --- | --- | --- | --- | --- | --- |
| Primary (Clinician were preferred to caregiver) | 45 | 1063 | -0.36 [-0.46, -0.26] | 98.86, <0.001 | 55.49 |
| Caregiver | 32 | 782 | -0.39 [-0.51, -0.26] | 80.99, <0.001 | 61.73 |
| Clinician | 16 | 302 | -0.28 [-0.39, -0.16] | 10.24, 0.801 | 0 |
| Teacher | - | - | - | - | - |

*In Saad_2015, it was unclear if CARS was rated only by parents or also filled by clinicians (no reply by the corresponding author). In Anagnostou_2012, SRS might have been rated by self-reports and not by caregivers. Therefore, they not included in the meta-regression of type of rater or in the separate analysis.

#### 4.3.2. Forest plot of overall core symptoms rated by caregivers


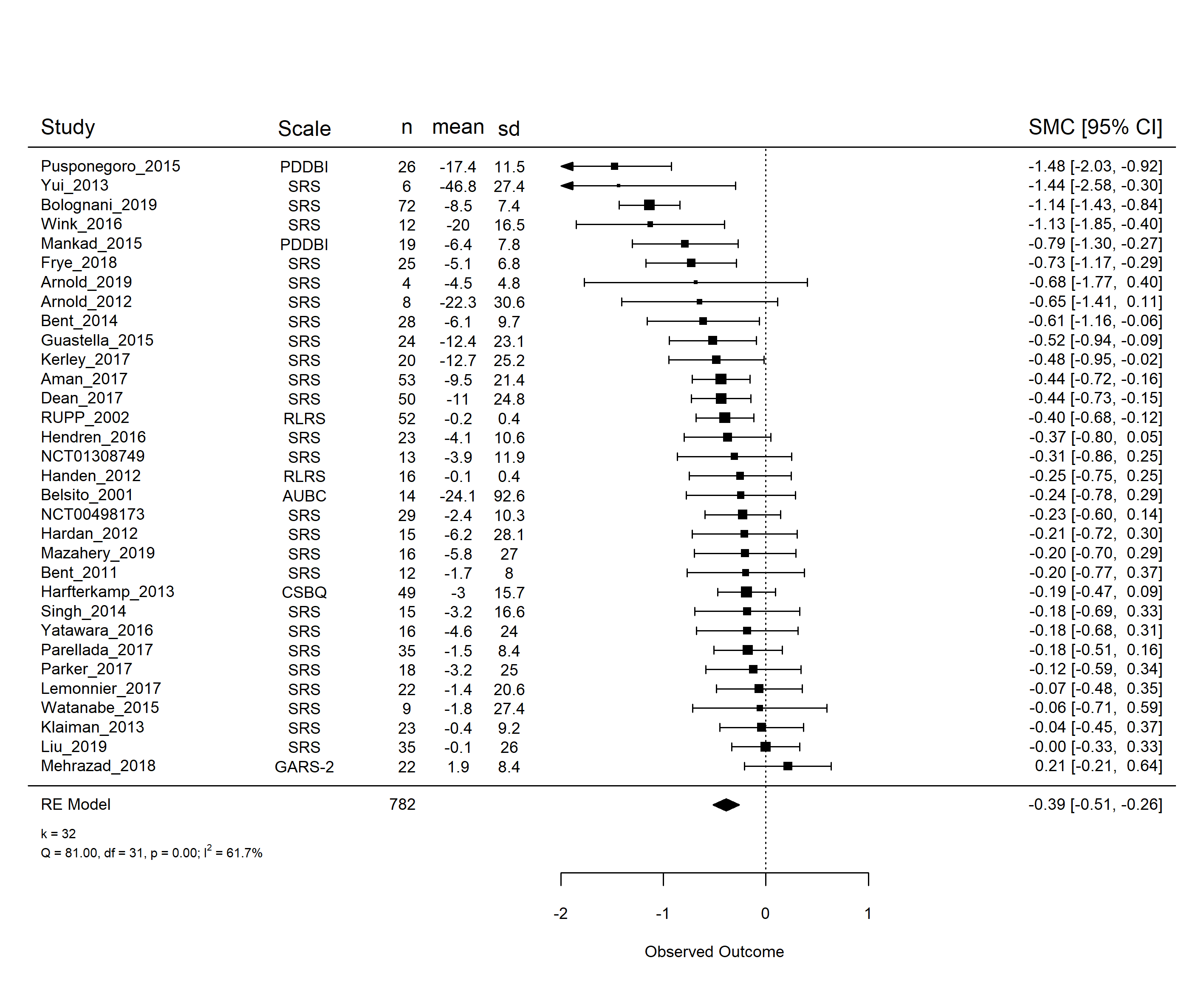


*It should be noted that the Ritvo-Freeman Life Rating Scale was rated by caregivers in RUPP_2002 and Handen_2012.

#### 4.3.3. Forest plot of overall core symptoms rated by clinicians


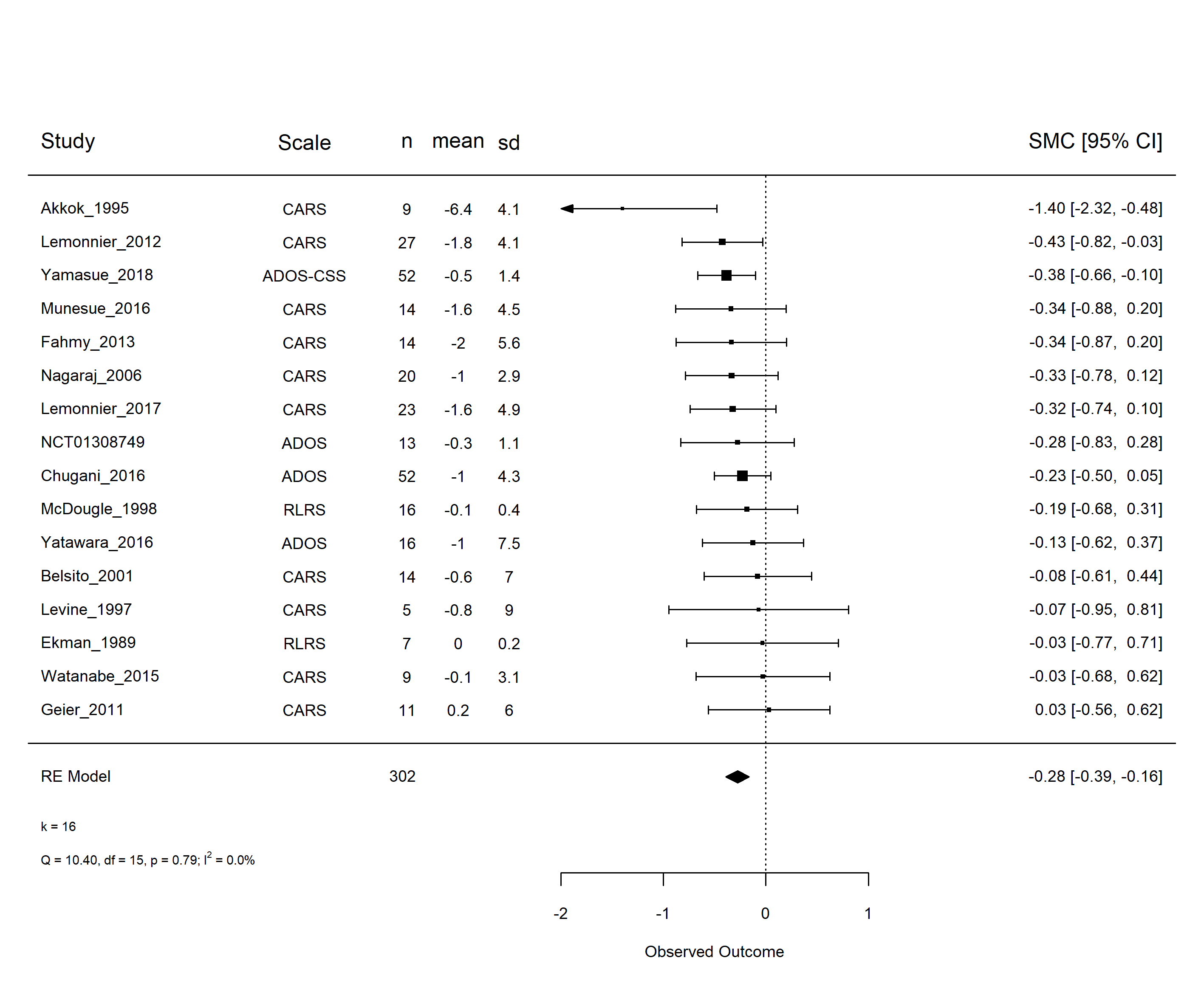


*SEs might have been reported in Chugani_2016 as SDs. Therefore, we calculated SDs from the reported values (no reply from corresponding author).

## 5. Correlation between standardized mean changes of placebo and experimental intervention

In order to investigate whether placebo response was correlated with the response of experimental interventions, Spearman’s rho were calculated between SMCs of placebo and experimental intervention. In case of multi-arm studies, the weighted mean of SMC was calculated for experimental intervention (arms with multimodal or other interventions, e.g. parental training + placebo or parental training + atomoxetine were excluded in Handen_2015). Reference lines of no change (SMC=0) and the equality line (SMC of placebo = SMC of experimental intervention) were added in scatter plots. Points below the equality line have a larger response in experimental intervention (SMC experimental intervention < SMC placebo).

### 5.1. Social-communication difficulties


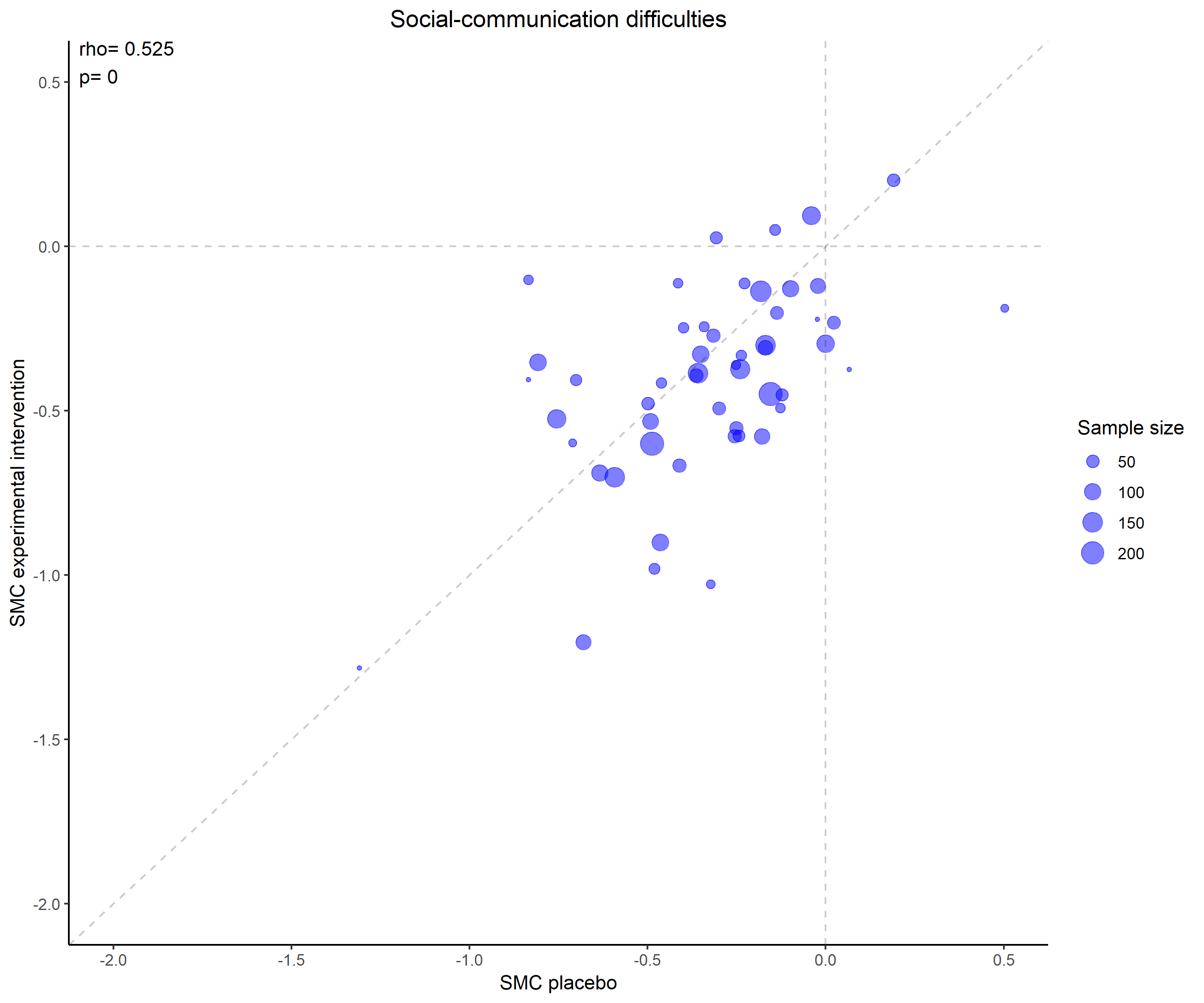


There was a correlation between SMCs for social-communication difficulties between placebo and experimental intervention (Spearman’s rho=0.525, p<0.001).

### 5.2. Repetitive behaviors


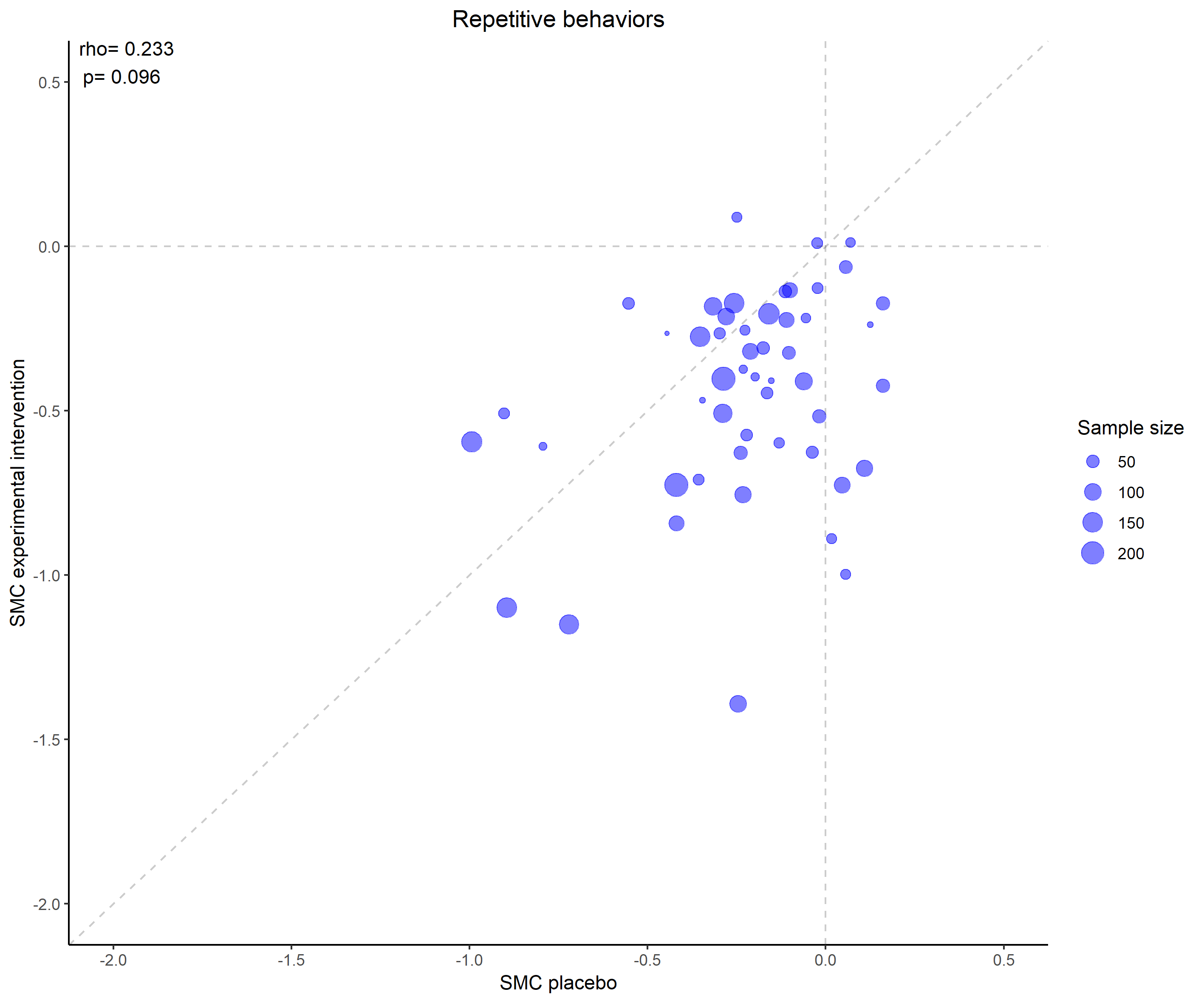


SMCs for repetitive behaviors between placebo and experimental intervention were not correlated (Spearman’s rho=0.233, p=0.096).

### 5.3. Overall core symptoms


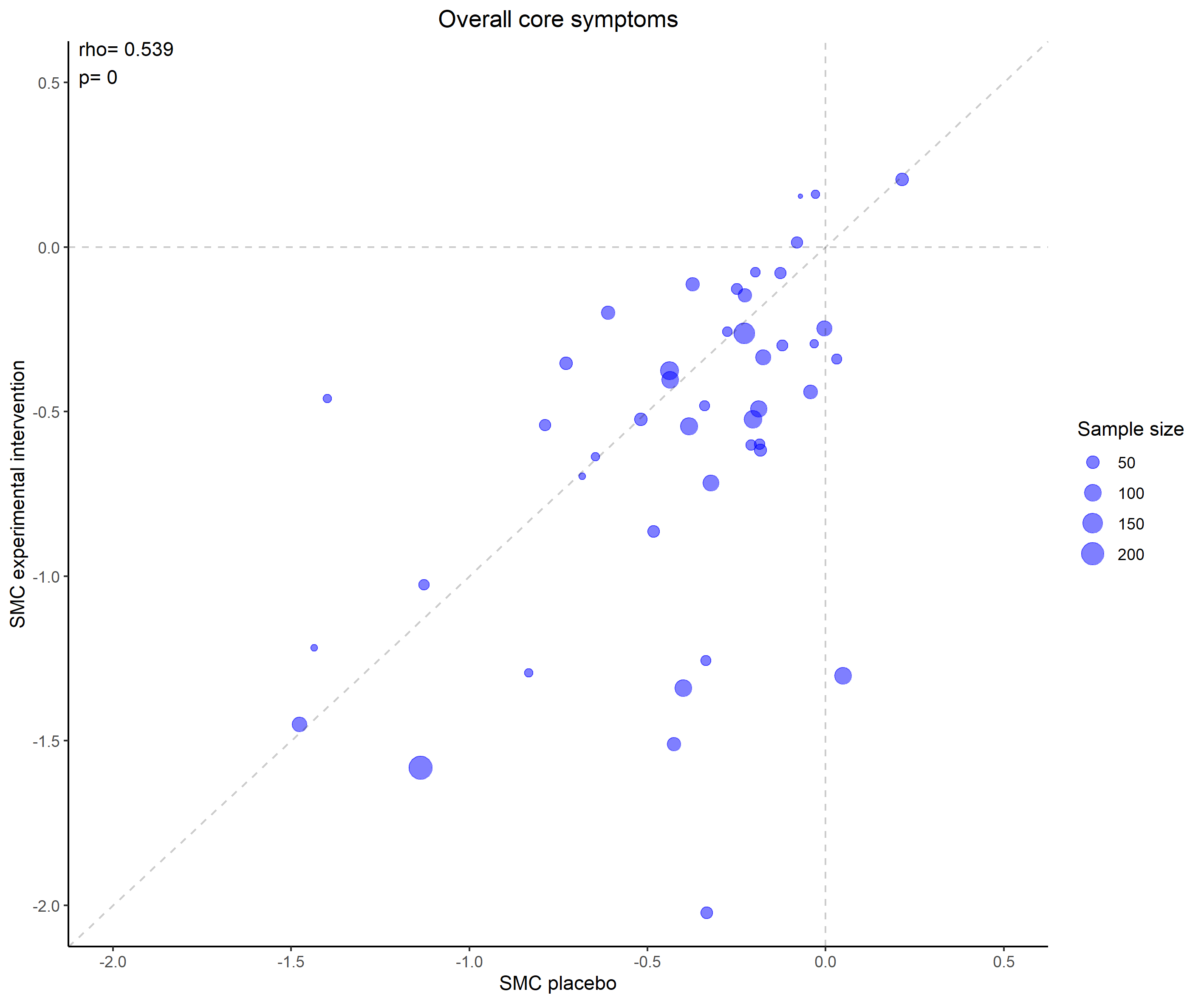


There was a correlation between SMCs for overall core symptoms between placebo and experimental intervention (Spearman’s rho=0.539, p<0.001).

## 6. References

1. Akkok FG, Bahar; Oktem, Ferhunde; Reid, Larry D.; Sucuoglu, Bulbin: **Otizm’de naltrekson sagaltiminin davranissal ve biyokimyasal boyutlari**. *Turk Psikiyatri Dergisi* 1995, **6**(4):251-262.

2. Stivaros S, Garg S, Tziraki M, Cai Y, Thomas O, Mellor J, Morris AA, Jim C, Szumanska-Ryt K, Parkes LM *et al*: **Randomised controlled trial of simvastatin treatment for autism in young children with neurofibromatosis type 1 (SANTA)**. *Mol Autism* 2018, **9**:12.

3. Leucht S, Chaimani A, Mavridis D, Leucht C, Huhn M, Helfer B, Samara M, Cipriani A, Geddes JR, Davis JM: **Disconnection of drug-response and placebo-response in acute-phase antipsychotic drug trials on schizophrenia? Meta-regression analysis**. *Neuropsychopharmacology* 2019.
